# Angiogenesis, Anti-Tumor, and Anti-Metastatic Activity of Novel α-Substituted Hetero-Aromatic Chalcone Hybrids as Inhibitors of Microtubule Polymerization

**DOI:** 10.3389/fchem.2021.766201

**Published:** 2021-11-19

**Authors:** Moran Sun, Yuyang Wang, Minghua Yuan, Qing Zhao, Yixin Zhang, Yongfang Yao, Yongtao Duan

**Affiliations:** ^1^ Henan Provincial Key Laboratory of Children's Genetics and Metabolic Diseases, Children's Hospital Affiliated to Zhengzhou University, Zhengzhou University, Zhengzhou, China; ^2^ School of Pharmaceutical Sciences and Institute of Drug Discovery and Development, Zhengzhou University, Zhengzhou, China

**Keywords:** microtubules, zebrafish, angiogenesis, chalcone analogs, anti-tumor

## Abstract

A library of new heteroaromatic ring-linked chalcone analogs were designed and synthesized of these, compound 7m with α-CH_3_ substitution and bearing a benzofuran ring, displaying the most potent activity, with IC_50_ values of 0.07–0.183 µM against three cancer cells. Its low cytotoxicity toward normal human cells and strong potency on drug-resistant cells revealed the possibility for cancer therapy. It also could moderately inhibit *in vitro* tubulin polymerization with an IC_50_ value of 12.23 µM, and the disruption of cellular architecture in MCF-7 cells was observed by an immunofluorescence assay. Cellular-based mechanism studies elucidated that 7m arrested the cell cycle at the G2/M phase and induced apoptosis by regulating the expression levels of caspases and PARP protein. Importantly, the compound 7 m was found to inhibit HUVEC tube formation, migration, and invasion *in vitro*. *In vivo* assay showed that 7m could effectively destroy angiogenesis of zebrafish embryos. Furthermore, our data suggested that treatment with 7m significantly reduced MCF-7 cell metastasis and proliferation *in vitro* and in zebrafish xenograft. Collectively, this work showed that chalcone hybrid 7m deserves further investigation as dual potential tubulin polymerization and angiogenesis inhibitor.

## 1 Introduction

Microtubules are composed of α- and β-tubulin heterodimers and served as key components of the eukaryotic cytoskeleton. The pipe-like microtubule network plays a pivotal role in several cellular functions including formation and maintenance of cell shape, intracellular vesicle transportation, cell signaling, and secretion. In recent decades, numerous microtubule-binding drugs have been used in therapeutic and palliative cancer chemotherapy regiments (Gigant et al., 2005; Chen et al., 2010; Li, 2012; Duan et al., 2016). Moreover, studies have shown that most microtubule-targeting agents also have anti-angiogenic or vascular-disrupting activities or both (M. R. Sun et al., 2021). Targeting tumor vasculature as a therapeutic approach has a compelling theoretical basis, which is complementary to other existing therapies (Tozer et al., 2005; Mckeage and Baguley, 2010; Greene et al., 2015). These compounds cause selective damage to tumor blood vessel, inducing deleterious morphological and functional changes. As a result, the endothelial cells of immature vasculature shut down, and blood flow within the tumor rapidly and dramatically reduced. Finally, extensive tumor necrosis will occur due to starvation of oxygen and nutrients (Schwartz, 2009).

In recent decades, many compounds had been identified to interfere with the tubulin–microtubule dynamic systems by binding to three well-known sites: the vinca domain, colchicine, and paclitaxel sites. Especially, vascular-disrupting agents binding to tubulin sites received so much attention. Taxol, the tubulin polymerization agent, could prevent melanoma metastases through angiogenesis inhibition (Wang et al., 2003). CA-4 and vinblastine, the tubulin depolymerization agents binding to the vinca domain and colchicine sites, respectively, also could induce an anti-angiogenesis effect and tumor vascular collapse through the rapid depolymerization of microtubules (J. T. Su et al., 2021). Inspired by their potent activity on anti-angiogenesis and vascular-disrupting activity, a series of the tubulin-binding site inhibitors affecting angiogenesis has been in different clinical phases such as CA-4P, CA-1P, and ABT-751 (Liu et al., 2015). However, for either the tubulin polymerization agents or depolymerization agents, the toxicity on normal cell lines and drug-resistance had been the obstacle for their further application in clinic.

**FIGURE 1:**
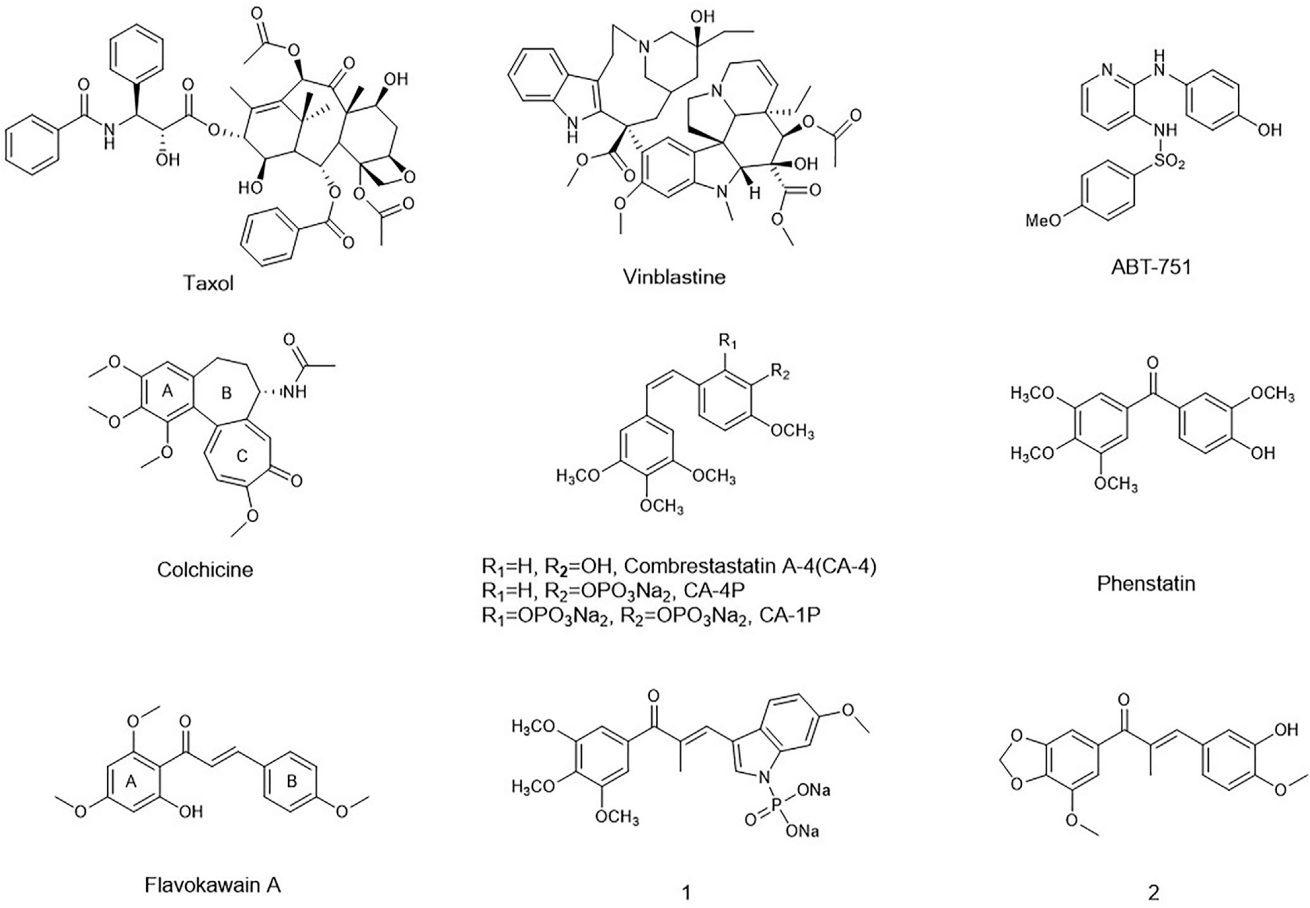
Representative tubulin inhibitors with anti- angiogenesis potency.

Chalcones, an attractive drug scaffold found in many natural products, have been used as a source of microtubule-destabilizing agents binding at the colchicine site (P. Singh et al., 2014; Zhuang et al., 2017). Chalcones bear an α,β-unsaturated carbonyl moiety and act as Michael acceptors to alkylate multiple sulfhydryl residues of many critical proteins. Several synthetic and naturally occurring chalcones have been shown to be with strong anti-tumor and angiogenesis potency. Flavokawain A was found to be a promising apoptosis inducer and arrested cell cycle at G2/M against HER2-overexpressing breast cancer cells (Figure 1) (Jandial et al., 2017). Incorporating the aryl substitution pattern of CA-4 into chalcones produces compound 1. It displayed low cytotoxicity toward normal cells and greater metabolic stability than CA-4, along with potent antiproliferative activity against drug-resistant cells (Yan et al., 2016). Chalcone 2 exhibited vascular disrupting effects and anti-metastatic activity similar to that of CA-4P (Canela et al., 2016).

Following our previous work on tubulin and angiogenesis (Duan et al., 2019; M. R.; Sun et al., 2021), to further discover and novel α-substituted chalcones with low toxicity, overcoming drug resistance and strong anti-angiogenesis property, we explored the incorporation of cyano, methyl functional group in the α-position. Considering the extensive pharmacological activity of heterocyclic analogs, indole, benzofuran, benzothiophene, and dihydrobenzofuran ring were used as benzene surrogates in the B-ring of chalcone. In all cases, as a feature structure of tubulin-binding agents, 3,4,5-trimethoxy substation in the A-ring was retained. We now report full details of the synthesis, characterization, and evaluation of their antiproliferative activities against cancer cells. The lead compound 7m was evaluated for its anti-angiogenesis and anti-metastatic properties *in vitro* and *in vivo*.

## 2 Results and Discussion

### 2.1 Chemistry

Initially, a series of α-position unsubstituted chalcones 6 were prepared in good yields using the Claisen–Schmidt condensation (Figure 12). In brief, propiophenone 3 was prepared by the addition of methylmagnesium bromide to 3,4,5-trimethoxybenzaldehyde, followed by PCC oxidation of the intermediate secondary alcohol. A similar strategy was used in the preparation of chalcone 7 containing a methyl group at the α-position in modest yields as a mixture of geometrical isomers (usually E:Z, 2:1), which were separated by column chromatography. Chalcone 8 with a cyano group at the usually E:Z, 2:1 position was synthesized by base-promoted Knoevenagel condensation from a wide range of heterocyclic aromatic aldehydes with β-ketonitrile 5. Intermediate 5 was obtained by the reaction of ethyl 3,4,5-trimethoxybenzoate with acetonitrile in the presence of sodium hydride.

### 2.2 Biology

#### 2.2.1 Antiproliferative Activity and Structure–Activity Relationship

The synthesized compounds were first evaluated for their cytotoxic effect on MCF-7 (human breast adenocarcinoma), U937 (histiocytic lymphoma cancer), HepG2 (human liver cancer) cells, and MGC-803 (human gastric cancer) using the SRB and CCK-8 assay. As reference compounds CA-4 and colchicine were included.

All the tested chalcones were classified into three categories according to the substitution type of the α-position, namely, α-unsubstituted chalcones (6), α-methyl chalcones (7), and α-nitrile chalcones (8). From the antiproliferative results of α-unsubstituted analogs in Table 1, we mainly investigated the effects of location of α,β-unsaturated ketone on the heterocyclic ring and activity differences of electron-withdrawing or -donating groups on the B-ring. Among compounds 6 b-e and 6g, chalcone 6 with bromide atom on the C6- of indole moiety displayed more potency than compounds 6d and 6e with methyl carboxylate on C4-, C5-, respectively (IC_50_ >20 µM). In contrast, compound 6c containing phenyl at the C2- position of indole displayed the most potent antiproliferative effect toward four cell cancer cell lines. As far as we know, the inhibitory activity of chalcones bearing oxygen or sulfur-containing heterocycle remains mostly unexplored. In the 6h, 6j, and 6l-n series, α,β-unsaturated ketone was incorporated at the ion of ketone, and the cytotoxic activity was C3-(6j) >CC2-, C3-, C5- of benzofuran, respectively. So, locat5-(6m) >C2-(6h). Interestingly, compound 6n containing a benzodihydrofuran fragment has comparable activity, particularly in α-methyl chalcone 7 (Table 2), where 7n displayed most potent activity in all test compounds. Regarding the benzothiofuran (6o–6q) and benzimidazole (6r) ring-B unit, most of the compounds in these two series displayed IC_50_ values below 10 μM, except for the inhibitory effect against U937. Taking Tables 1 and 2 together, we also found that benzodioxole 6s was one of the most potent compounds in this assay.

**TABLE 1:** Evaluation of *in vitro* antiproliferative activity of α-unsubstituted chalcones (6) (IC_50_ ^a^).

Compound	IC_50_ ± SD (µM)			
	U937	MCF-7	HepG2	MGC-803
6b	>20	0.281 ± 0.022	14.110 ± 0.117	0.190 ± 0.002
6c	0.601 ± 0.267	0.313 ± 0.017	1.076 ± 0.092	0.104 ± 0.001
6d	>20	>20	>20	>20
6e	>20	>20	>20	>20
6g	>20	0.380 ± 0.020	18.060 ± 0.295	0.071 ± 0.002
6h	>20	14.590 ± 0.368	>20	8.784 ± 0.309
6j	0.786 ± 0.346	0.988 ± 0.006	4.817 ± 0.102	0.395 ± 0.015
6L	2.082 ± 0.252	0.810 ± 0.068	3.269 ± 0.065	0.326 ± 0.016
6m	6.173 ± 1.645	6.457 ± 0.103	>20	3.181 ± 0.062
6n	>20	3.132 ± 0.415	>20	0.851 ± 0.063
6o	>20	2.068 ± 0.043	8.375 ± 0.585	1.377 ± 0.086
6p	>20	0.283 ± 0.011	1.378 ± 0.136	0.076 ± 0.001
6q	6.384 ± 0.148	5.086 ± 0.569	10.650 ± 0.457	3.219 ± 0.284
6r	1.278 ± 0.071	5.186 ± 0.465	4.673 ± 0.295	2.032 ± 0.225
6s	>20	1.234 ± 0.133	3.322 ± 0.229	0.393 ± 0.017
CA-4	0.0003 ± 0.0001	0.0114 ± 0.0002	0.0071 ± 0.0009	NA

a Note. Each compound was tested in triplicate; the data are presented as the mean ± SD.

U937, histiocytic lymphoma cancer; MCF-7, human breast adenocarcinoma; HepG2, human liver cancer; MGC-803, human gastric cancer.

**TABLE 2:** Evaluation of *in vitro* antiproliferative activity of α-methyl chalcones (7) and α-nitrile chalcones (8) (IC_50_ ^a^).

Compound	IC_50_ ± SD (µM)		
	U937	MCF7	HepG2
7b	3.084 ± 0.433	0.584 ± 0.016	10.780 ± 0.629
7e	>20	>20	>20
7f	1.615 ± 0.190	13.740 ± 0.965	0.193 ± 0.001
7h	0.224 ± 0.007	0.254 ± 0.010	0.116 ± 0.007
7i	1.176 ± 0.037	1.406 ± 0.021	0.879 ± 0.007
7m	0.082 ± 0.002	0.1833 ± 0.032	0.070 ± 0.001
7n	0.011 ± 0.002	0.020 ± 0.002	0.002 ± 0.0001
7o	0.166 ± 0.003	0.187 ± 0.003	0.116 ± 0.001
7s	0.353 ± 0.006	0.2247 ± 0.005	0.259 ± 0.005
8a	0.140 ± 0.041	0.934 ± 0.023	0.938 ± 0.008
8b	>20	>20	>20
8c	4.492 ± 0.433	2.096 ± 0.270	14.08 ± 0.386
8d	8.584 ± 0.178	>20	>20
8e	>20	>20	>20
8f	>20	>20	>20
8g	2.508 ± 0.029	2.161 ± 0.009	1.426 ± 0.049
8h	>20	>20	>20
8i	>20	>20	>20
8j	>20	>20	>20
8k	14.313 ± 0.127	16.43 ± 0.042	18.742 ± 0.035
8l	>20	>20	>20
8m	>20	>20	>20
8n	9.253 ± 0.141	>20	>20
8o	>20	14.74 ± 0.141	1.701 ± 0.013
8p	>20	>20	>20
8q	52.322 ± 3.655	>20	>20
8r	24.917 ± 2.015	>20	>20
8s	>20	>20	>20
8t	>20	>20	>20
CA-4	0.0003 ± 0.0001	0.0114 ± 0.0002	0.0071 ± 0.0009

a Note. Each compound was tested in triplicate; the data are presented as the mean ± SD.

**FIGURE 2:**
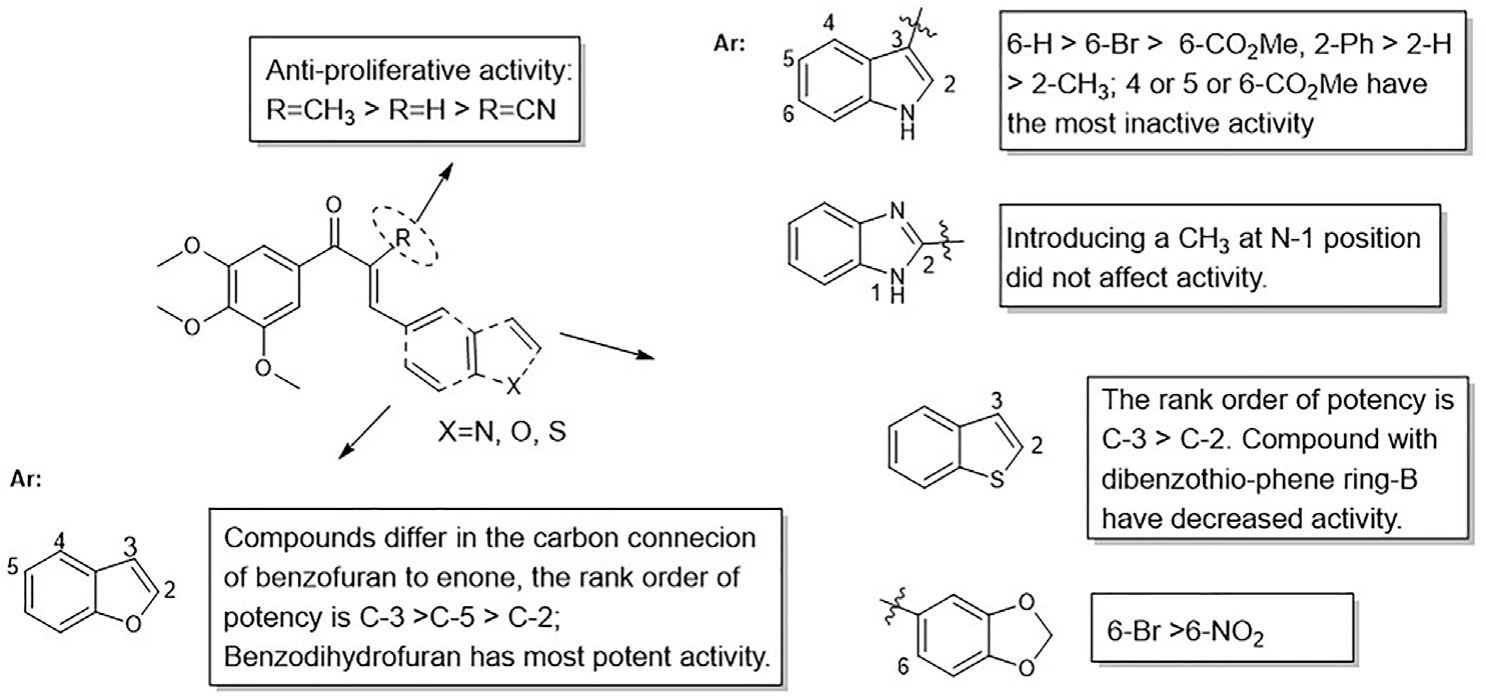
Summarized SARs of synthesized chalcone analogs.

On the other hand, the growth inhibitory activity was highly dependent on the type of α-position substitution. A striking improvement in potency was observed in α-methyl chalcones (7) with the IC_50_ values in sub-micromolar range mostly, whereas the most inactive compounds were observed in α-nitrile chalcones (8) with the majority of IC_50_ values >20 µM. The SARs of all the synthesized compounds are summarized in Figure 2.

#### 2.2.2 Analysis of Tubulin Polymerization *In Vitro*

Among the three series of chalcones above, 7n displayed the best activity toward six cancer cell lines, but with an unfavorable concentration dependence. The compound 7m bears an α-methyl substitution, and the benzofuran ring-B inhibited cell growth with IC_50_ values from 0.07 to 0.183 µM, somewhat less active than 7n. Accordingly, the compound 7m was selected to evaluate the inhibitory effects on tubulin polymerization *in vitro* to determine whether microtubule system could be a potential target.

**FIGURE 3:**
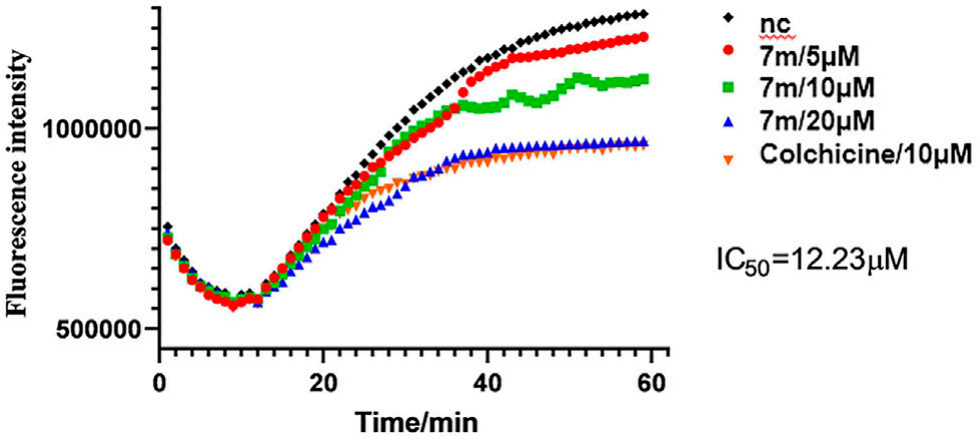
Tubulin polymerization inhibitory activity of 7m *in vitro*. Polymerization of tubulin at 37°C, in the presence of 1% DMSO, compound 7m (5, 10, and 20 µM), and colchicine (10 µM), was measured by monitoring the excitation at 335 nm and emission at 460 nm. Data are presented as the mean ± SD from three independent experiments. **p < 0.01 vs. the vehicle control.

The microtubule-destabilizing agent colchicine was known to inhibit self-polymerization of tubulin. To investigate whether these α-substituted chalcone hybrids target the tubulin–microtubule system, compound 7m was employed at concentrations of 5, 10, and 20 μM for microtubule dynamic assays with colchicine as positive control. In this assay, the increasing fluorescence intensity with time indicated that the purified and unpolymerized tubulin was self-polymerized to microtubules. As shown in Figure 3, after tubulin was incubated with 7m at 5, 10, 20 µM, the increased tendency of the fluorescence intensity was obviously slowed down with increasing concentration, with a similar action to that of colchicine. In addition, 7m produces an obvious concentration-dependent inhibition of tubulin polymerization with an IC_50_ value of 12.23 µM, which was twofold less potent than that of colchicine (IC_50_ = 6.036 µM) (Gao et al., 2019). The results suggested that the mechanism of compound 7m was consistent with previously reported microtubule-destabilizing agents, certifying that compound 7m can act as potential microtubule-destabilizing agents for cancer therapy.

#### 2.2.3 The Selectivity of 7m Toward Normal Cells and Taxol-Resistant Cells

The effect of 7m on normal liver LO2 cells and taxol-resistant MCF7 cells (MCF7/Tax) was further investigated (Table 3). Compound 7m showed a low toxicity toward normal liver LO2 cells with an IC_50_ value of 890 nM. In comparison with paclitaxel and CA-4, 7m was more selective for the tumor MCF7 cells over the normal LO2 cells. For the MCF7/Tax cells, 7m showed nearly equally potent activity (vs. MCF7) with RF (resistant fold) = 1.2, comparable with that of taxol (RF = 13.7). Therefore, 7m had the potential to overcome multidrug resistant with low toxicity.

**TABLE 3:** Growth inhibitory effects of 7m on LO2 and MCF7/Tax cells.

Compound	IC_50_ ± SD (nM)^a^			Selectivity ratio^b^	Resistant fold^c^
	MCF7	MCF7/Tax	LO2		
7m	183 ± 32	213 ± 26	890 ± 14	4.9	1.2
CA-4	11.4 ± 0.2	21.2 ± 0.6	15.2 ± 0.5	1.3	1.9
Taxol	24.6 ± 3.2	336 ± 18	32.4 ± 1.9	1.3	13.7

a Note. Each compound was tested in triplicate; the data are presented as the mean ± SD.

b Selectivity ratio = (IC_50_ human normal cells LO2)/(IC_50_ MCF7).

c Resistant fold = (IC_50_ MCF7/Tax)/(IC_50_ MCF7).

#### 2.2.4 Molecular Modeling Studies

**FIGURE 4:**
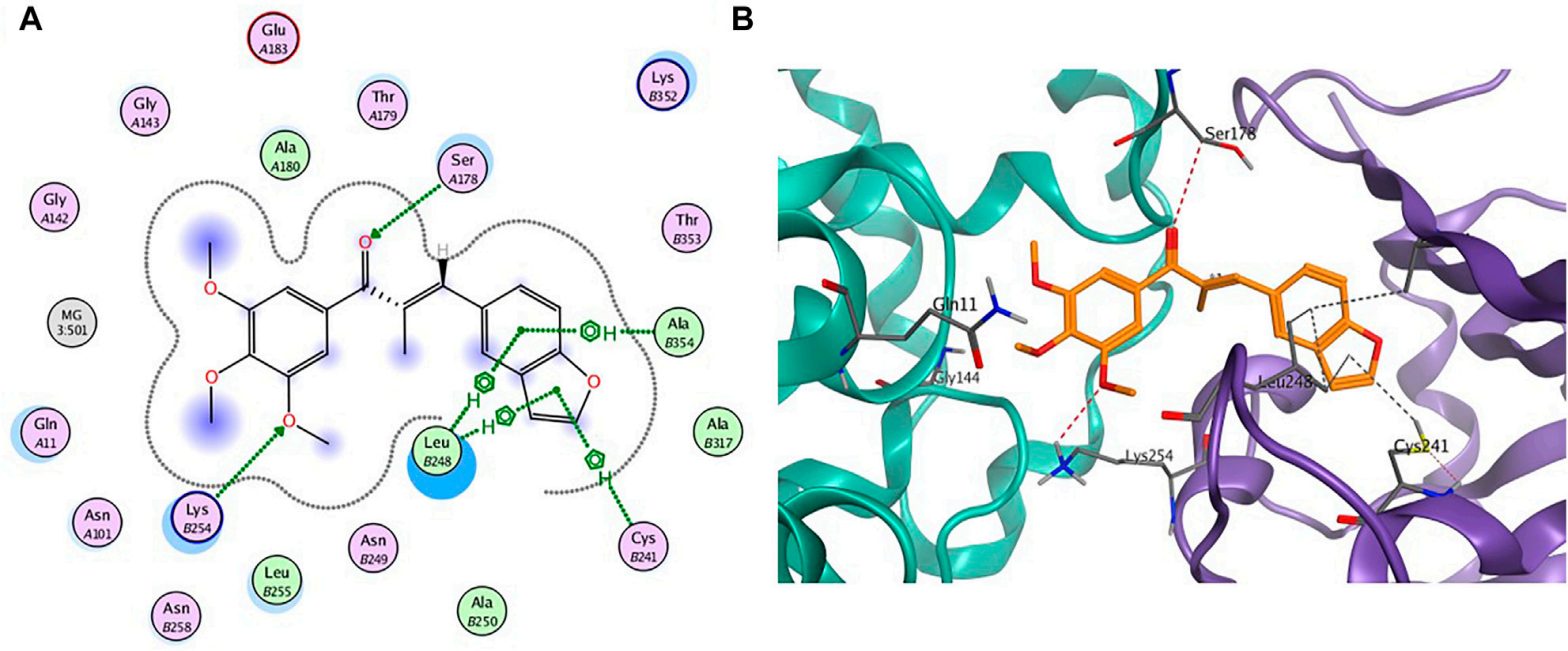
**(A)** The 2D binding model and hydrogen-bond interactions. **(B)** A 3D binging model of 7m in different configurations.

To further gain insight on the potential binding site of compound 7m with tubulin, molecular docking studies were performed using the ligand docking program (PDB code: 1SA0). As shown in Figure 4, 7m is wholly located at the interface of the tubulin heterodimer and inhibits the normal biological function of microtubules by preventing the combination of curved microtubules and straight microtubules. Meanwhile, hydrogen-bonding interactions include the ketone carbonyl moiety of chalcone with α-Ser178 residue and the oxygen atom on the methoxyphenyl group with α-Lys254. The benzene ring of benzofuran in the compound 7m forms CH-π stacking interactions with β-Leu248 and β-Ala354, and furan ring forms CH-π stacking interactions with β-Leu248 and β-Cys241. Through these four nonpolar forces, benzofuran can better interact with the pockets of the tubulin. The docking studies further indicated that compound 7m may be a potential tubulin inhibitor binding to the colchicine site.

#### 2.2.5 Compound 7m Induced Alteration of the Microtubule Network

**FIGURE 5:**
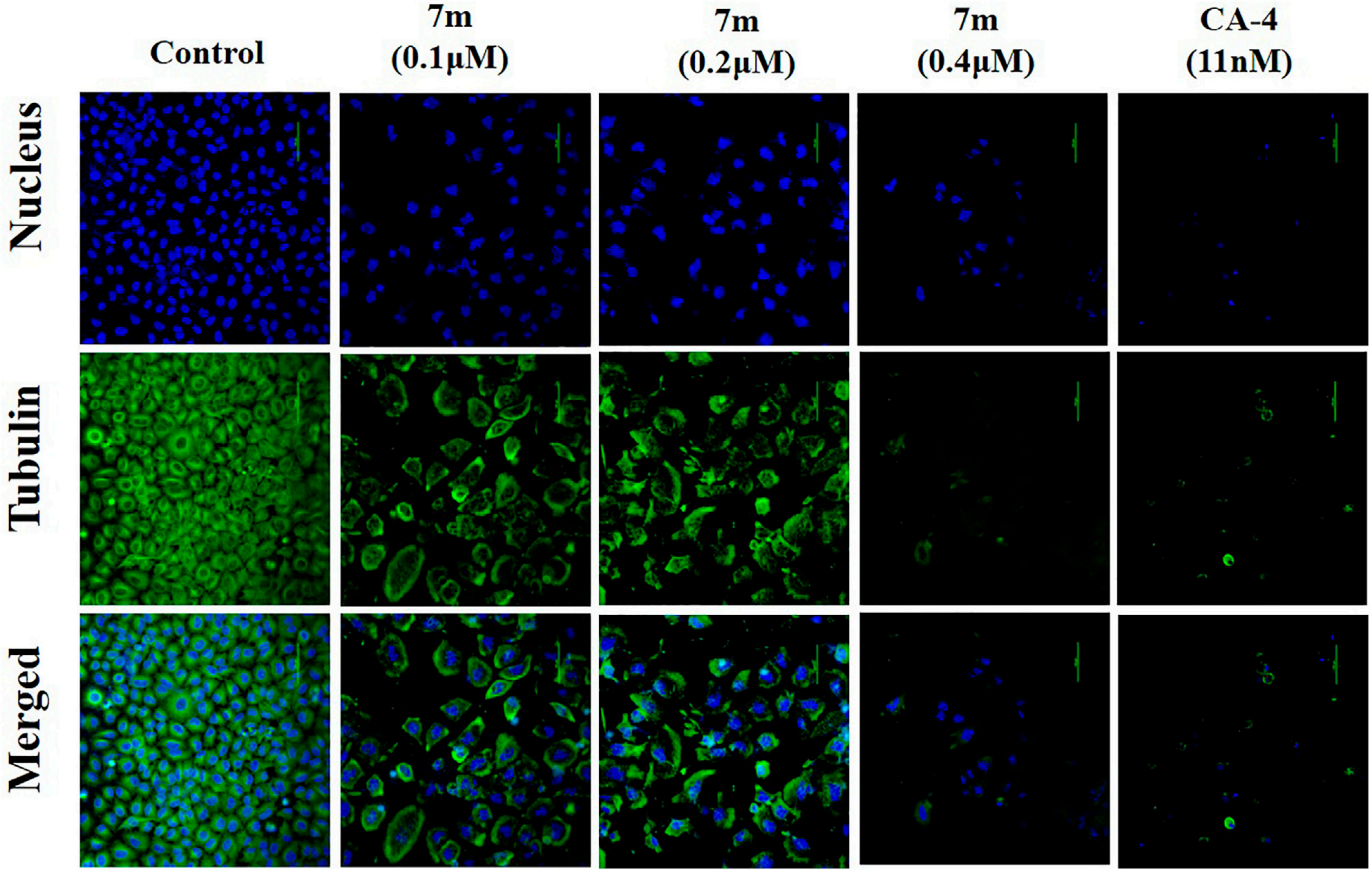
Immunofluorescence assay analysis of the effect of compound 7m on the cellular microtubule network after treatment with DMSO (control) or 7m at the indicated concentrations (0.1, 0.2, and 0.4 µM) for 24 h. Microtubules and unassembled tubulin are shown in green, and DNA is shown in blue.

Microtubule dynamics play an essential role in cancer cell growth. To examine whether compound 7m is able to disrupt microtubule dynamics, an immunofluorescent assay was carried out in MCF-7 cells. As shown in Figure 5, MCF-7 cells in the control group exhibited normal arrangement and intact organization. However, exposure to 0.4 μM of 7m for 24 h led to significant disruption of microtubule organization, which was comparable with the effect of microtubule-destabilizing reference compound CA-4. These results suggest that 7m could destabilize microtubule by inhibiting tubulin polymerization and disturbing microtubule networks.

#### 2.2.6 Cell Cycle Distribution Effect of Compound 7m

**FIGURE 6:**
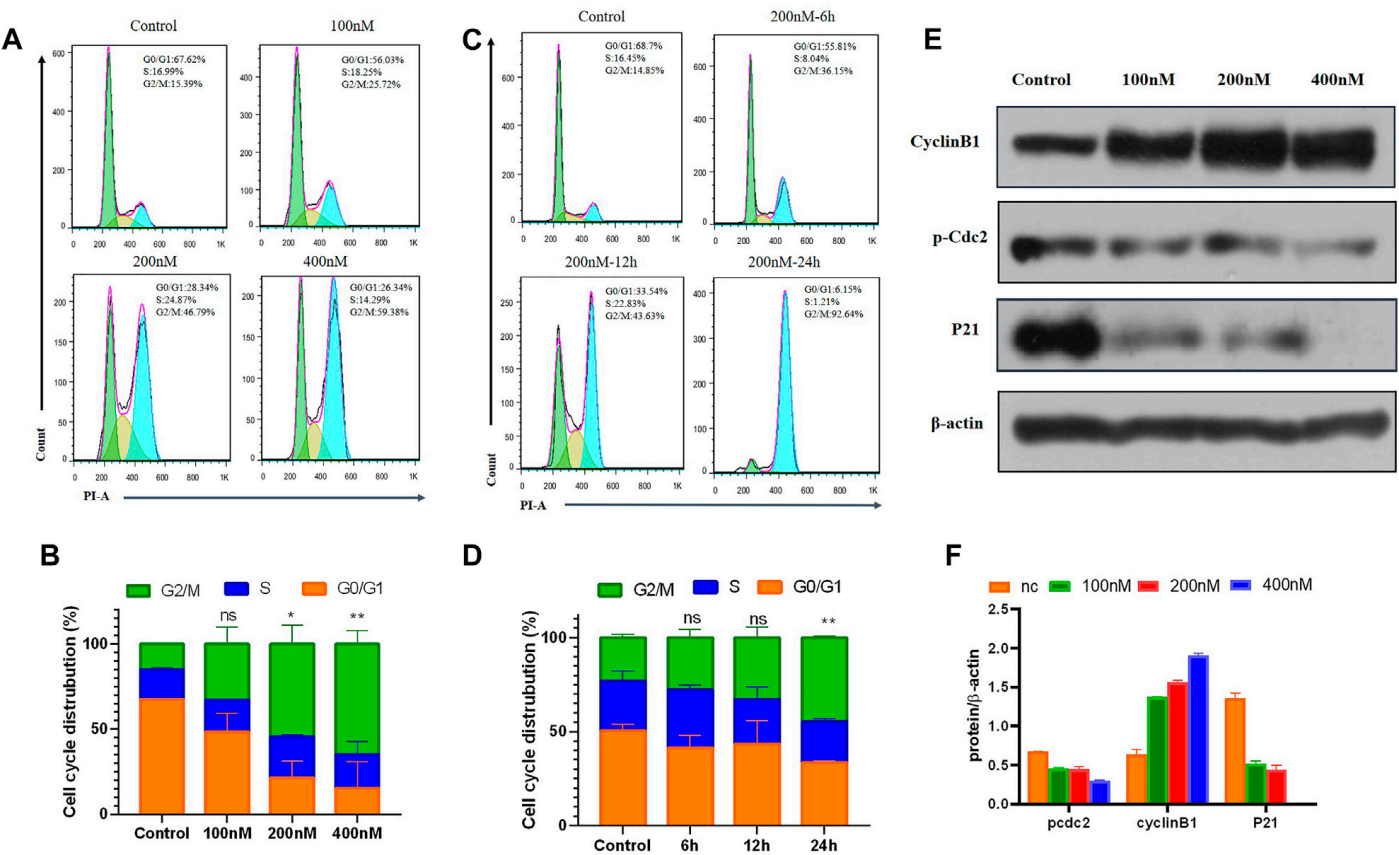
Compound 7m induced cell cycle arrest in G2/M arrest on MCF-7 cells. **(A)** Cells were treated with indicated concentrations of 7m for 24 h, and profiles were obtained by flow cytometry. **(B)** The percentage of cell cycle from **(A)** is illustrated in plots **(C).** Cells were treated with 200 nM 7m for 0, 6, 12, 24 h, respectively, and analyzed by flow cytometry. **(D)** The percentage of cell cycle from **(C)** is illustrated in plots. **(E)** MCF-7 cells were treated with 7m, then the cells were harvested, and the protein expressions of Cyclin B1, *p*-Cdc2, and P21 were detected by Western blotting assay. **(F)** Quantitative analysis of the expression of Cyclin B1, *p*-Cdc2, and P21 from experiments as in **(E)**. Data are presented as mean ± SD of at least three independent experiments.

It is known that tubulin inhibitors block the cell cycle in the G2/M phase because of microtubule depolymerization and cytoskeleton disruption. Therefore, the effects of 7m on cell cycle progression of MCF-7 cells were analyzed by flow cytometry with PI staining. MCF-7 cells were treated with different concentrations (0, 100, 200, and 400 nM) of compound 7m for 24 h and 200 nM 7m at different times (0, 6, 12, and 24 h), respectively. As shown in Figure 6, the number of cells in the G2/M fraction was significantly increased to 26% with 100 nM compound 7m. When the concentration was increased to 200 and 400 nM, cells of 47% and 56% G2/M phase arrest were observed, respectively. Similarly, this phenomenon occurred after treatment with 200 nM 7m from 0 to 24 h. These results suggested that compound 7m induced cell cycle progression arrest in G2/M phase in both dose- and time-dependent manner.

Cell cycle regulatory proteins Cyclin B1 and Cdc2 are key proteins to control the transition from G2 to M phase through increasing the G2/M intracellular fraction in cell cycle distribution. Some agents produce cell cycle arrest by decreasing Cyclin B1 level (Xiao et al., 2018), while others increase the Cyclin B1 level, depending on the cell types (Zhang et al., 2016). The effects of 7m on the cell cycle-related proteins were further tested, and the results are shown in Figure 6. It revealed that 7m dose-dependently decreased *p*-Cdc2 protein level, and meanwhile, Cyclin B1 is overexpressed in MCF-7 cells. It was due to the decreased level of *p*-Cdc2 protein, which inhibited the activity the *p*-Cdc2/Cyclin B1 complex, thus, blocking the ubiquitin degradation of Cyclin B1 and accordingly leading to dramatically upregulated expression of Cyclin B1. These results suggested that the 7m-induced G2/M arrest may be correlated with the changes in *p*-Cdc2/Cyclin B1 complex expression.

#### 2.2.7 Compound 7m Induced Apoptosis in Human Breast Adenocarcinoma Cells

**FIGURE 7:**
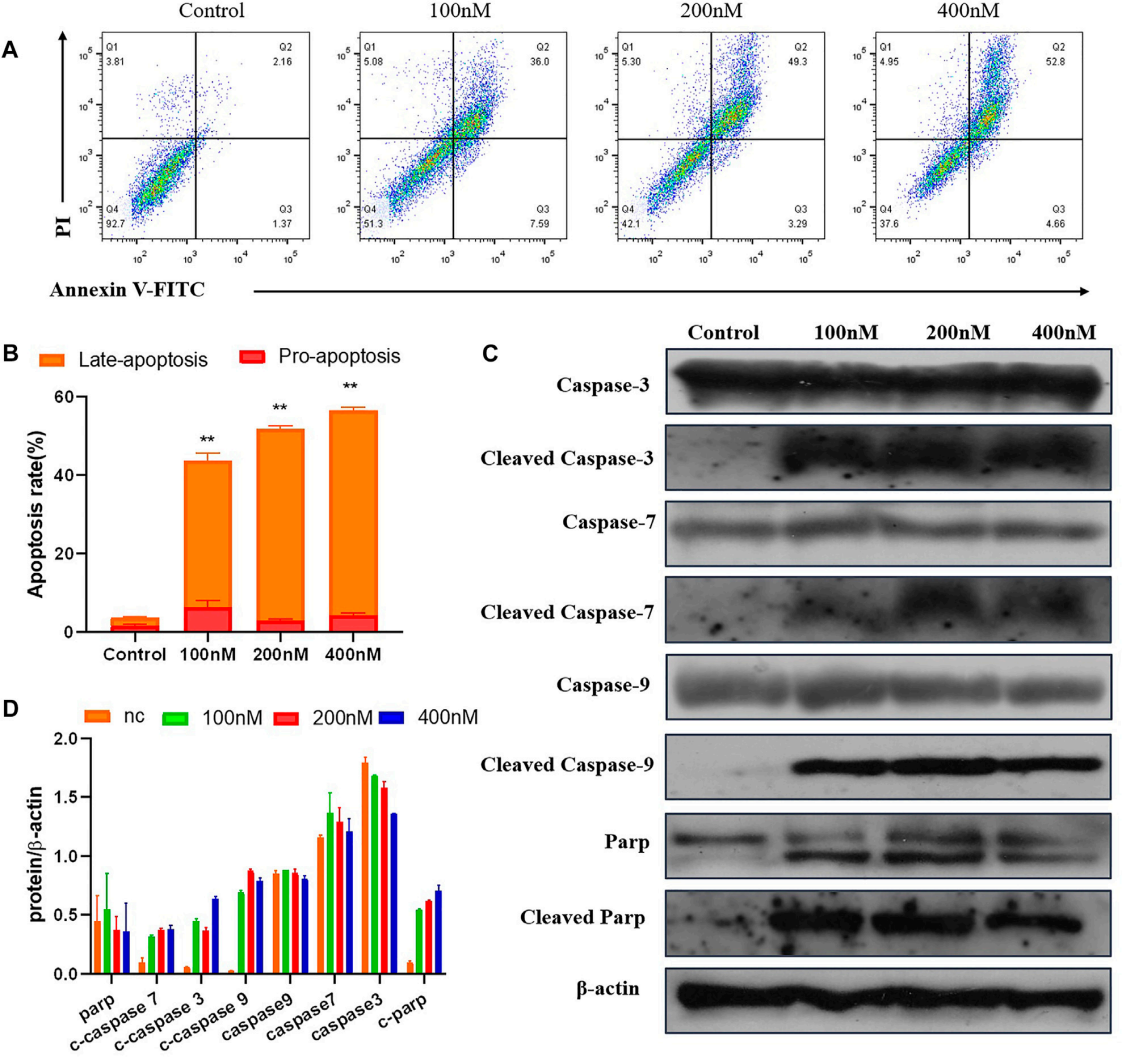
The compound 7m induces MCF-7 cell apoptosis. **(A)** Apoptosis ratio detection by Annexin/PI double staining assay through flow cytometry analysis treated with indicated concentrations of compound 7m for 48 h. **(B)** The quantitative analysis of apoptotic rate at early and advanced stages of MCF-cells. **(C)** Western blot analysis of the apoptosis-related proteins. **(D)** The quantitative analysis of the protein levels. The data are presented as the mean ± SD of three independent tests.

To test whether compound 7m induced reduction in cell viability was responsible for the induction of apoptosis, MCF-7 cells were stained with Annexin-V FITC/PI staining, and the number of apoptotic cells was estimated by flow cytometry. As shown in Figure 7, compared with the control group (4%, early and late apoptosis), the percentage of apoptotic cells was changed gradually from 44% to 53% and 57% after treatment with different concentrations of 7m (100, 200, and 400 nM) for 48 h. These results exhibited that compound 7m can induce apoptosis in MCF-7 cells in a dose-dependent manner.

The Caspase family proteins are considered to be critical regulators of cell apoptosis and facilitate cellular disassembly. Among them, Caspase-9 is termed as a typical caspase and is usually activated first, which in turn activates the “executioner” of apoptosis induction, in particular, Caspase-3. It is known that the activation of poly (ADP-ribose) polymerase (PARP), a downstream substrate of Caspase-3, is a common feature of nuclear fragmentation and chromatin condensation (Hensley and Kyprianou, 2013). Thus, the association between 7m-induced apoptosis and the expression of these proteins was investigated by Western blot analysis. MCF-7 cells were treated with 7m at different concentrations (100, 200, and 400 nM) for 48 h. As shown in Figure 7, 7m in MCF-7 cells produced a concentration-dependent activation of Caspase-3/-7/-9. Furthermore, a significant cleavage of PARP was also observed after incubations with 7m, confirming its proapoptotic activity.

#### 2.2.8 Compound 7m Inhibited Migration, Invasion, and Tube Formation of Vascular Endothelial Cells

**FIGURE 8:**
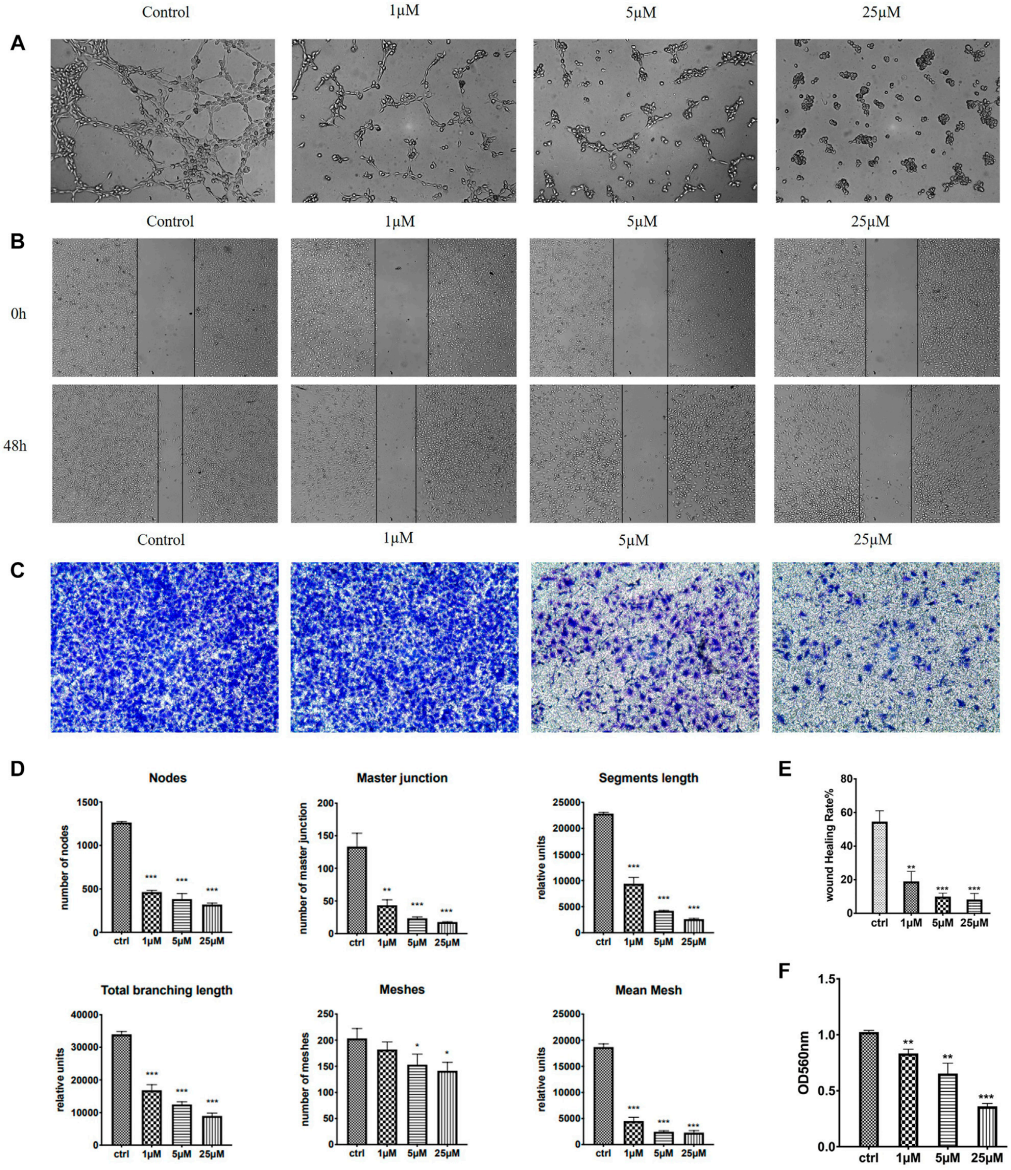
Effect of 7m on the human umbilical vein endothelial cells (HUVECs) migration, invasion, and tube formation. **(A)** Representative images depicting the vascular structure formation of HUVECs by treatments with 7m for 6–8 h. **(B)** Scratches were created and images were captured using phase-contrast microscopy at 0 and 48 h after treatments with 7m at different concentrations (1, 5, and 25 µM) or 1% DMSO. **(C)** The invasion suppressing effects of 7m against HUVECs by Transwell assay. **(D)** Quantitative evaluation of indicated concentrations of 7m on standard parameters of HUVEC tubule formation. Mean ± SEM of three experiments. **p < 0.01; ***p < 0.001 **(E)** Inhibitory rates of 7m on the HUVEC migration. **(F)** Quantitative analysis of the migration ability. *p < 0.05, **p < 0.01; ***p < 0.001, vs. control; *n *= 3.

HUVECs (human umbilical vein endothelial cells) grown on Matrigel were commonly used as a tube formation model and also a widely studied human endothelial cell type in angiogenesis, for their ability to alter their shape to resemble capillaries (Aranda and Owen, 2009). Quite a few anti-tubulin drugs such as CA-4 were found to have both the anti-angiogenic (inhibit the information of new tumor vessels) and vascular-disrupting (destroy existing vessels) effects (Su et al., 2016). We therefore examined the effects of 7m on HUVEC tube formation to evaluate its potential effect on angiogenesis. As shown in Figure 8, after treatment with 1, 5, and 25 μM of 7m, the width and length of “tubule-like” network formation, observed in the untreated HUVECs, were reduced in a concentration-dependent manner. We also performed image analysis in order to obtain a quantitative determination of the segment length, the area, and the number of meshes, the percent of area covered by HUVECs, and the number of branching points after 7m treatment, which statistically suggested the high potential of vascular-disrupting activity of 7m.

Except for targeting tumor vasculature and interfering with new blood vessels formation, the agents binding to the colchicine site have also been reported to inhibit cell migration and motility (Banerjee et al., 2016). A wound healing assay was, thus, performed to evaluate the capacity of 7m to inhibit cell motility. As shown in Figure 8, 7m obviously decreased the closure of wound scratching in a confluent monolayer of HUVEC cells with a dose–response relationship observed, after exposure to 7m at 0, 1, 5, and 25 μΜ for 48 h. Then the invasion inhibitory activities of 7m were further detected using Transwell assay with HUVECs cells treated with 0, 1, 5, and 25 μΜ of 7m. In this assay, MCF-7 cells were seeded into upper chambers which were pre-coated with Matrigel matrix in order to evaluate the cellular invasion. With the increase in the concentration of 7m, as shown in Figure 8, the invasion ability of cells gradually weakened, and the number of migrated endothelial cells gradually decreased. These results collectively indicated that 7m has significant effect on prohibiting HUVEC tubular structure formation, migration, and invasion.

#### 2.2.9 Compound 7m Inhibited Migration and Invasion of Human Breast Adenocarcinoma Cells

**FIGURE 9:**
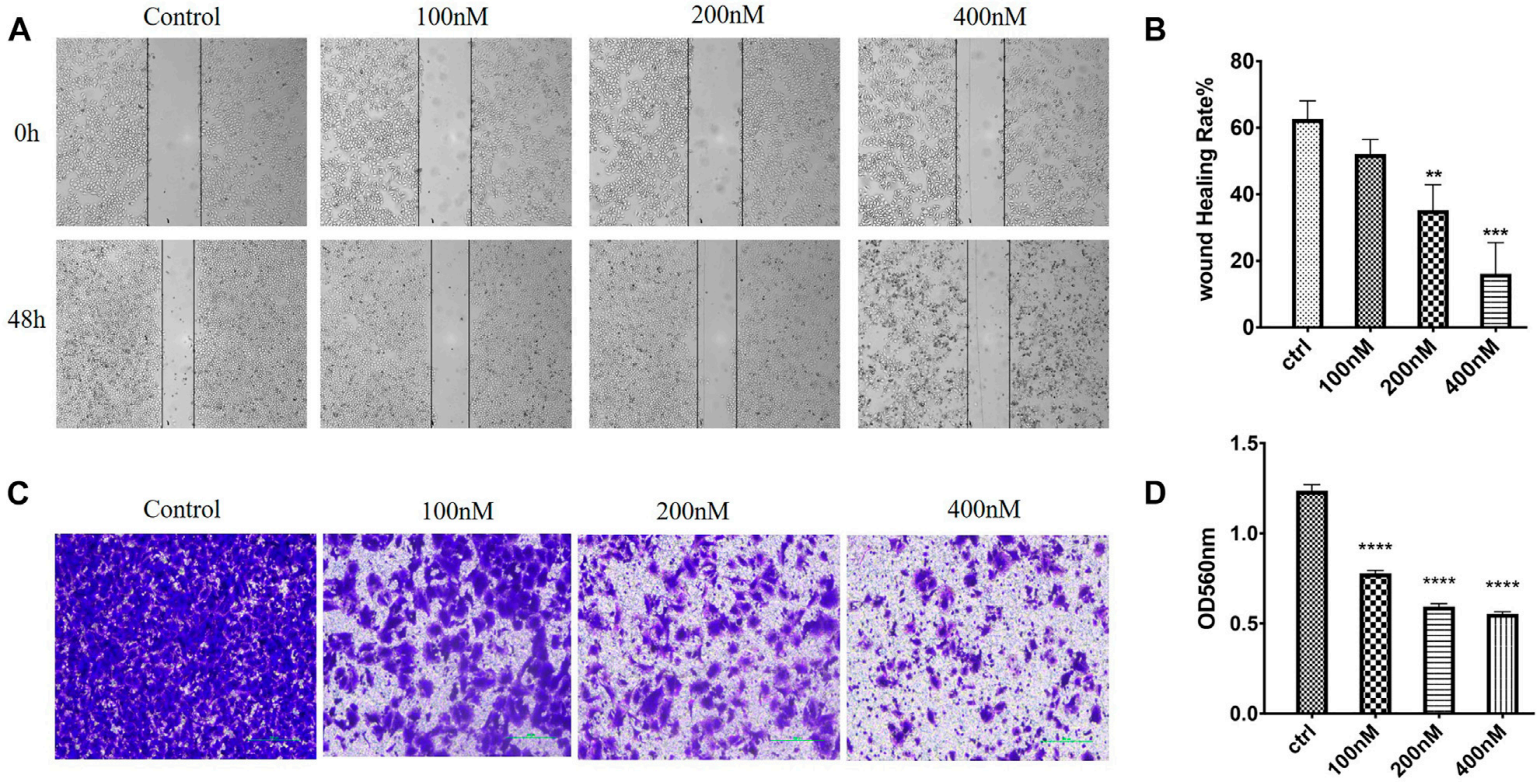
Effects of 7m on human breast adenocarcinoma (MCF-7) cell migration and invasion *in vitro*. **(A)** Images of MCF-7 cell migration inhibited by 7m determined in wound healing assay. **(B)** Inhibitory rates of 7m on MCF-7 cell migration. **(C)** Inhibition of cellular invasion by 7m in Transwell assay. **(D)** Quantitative analysis of the migration ability of MCF-7 cells after the treatment of 7m. The experiments were repeated three times, and results are indicated as means ± SD. *p < 0.05, **p < 0.01; ***p < 0.001 vs. untreated control.

In order to evaluate whether 7m could suppress migration and invasion, the MCF-7 cell culture was performed by wound healing and Transwell Matrigel assay, respectively. As shown in Figure 9, after 48 h, the 7m treatment significantly inhibited the wound healing of MCF-7 cells in a dose-dependent manner, indicating that compound 7m could inhibit tumor cell migration. In addition, Transwell Matrigel assays showed that 7m could hinder the MCF-7 cells invasion in a dose-dependent manner (Figure 9).

#### 2.2.10 Compound 7m Reduced Angiogenesis in Zebrafish Embryos

**FIGURE 10:**
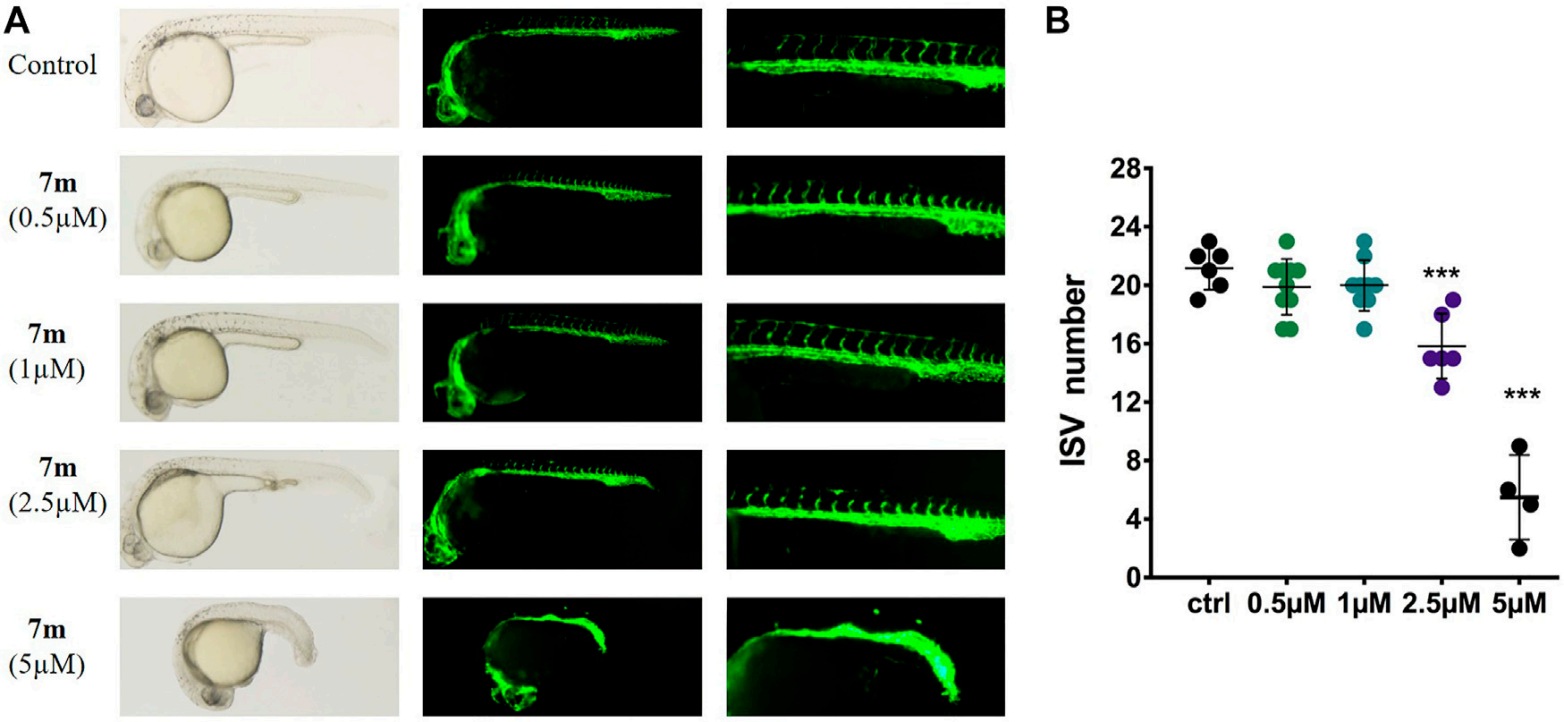
Anti-angiogenic effects of 7m in zebrafish embryos. **(A)** Zebrafish embryos were incubated with the tested compounds at 0.5, 1, 2.5, and 5 µM. The left angiogenic vessels were magnified and shown in the right section. **(B)** Histogram showed the numbers of zebrafish intersegmental vessels (ISVs) per field under confocal microscopy. Results represented the means ± SD. **p < 0.01; ***p < 0.001 compared with untreated control.

Zebrafish embryo is a powerful model to study human diseases, and the transgenic zebrafish labeled with green fluorescent protein (GFP) in endothelial cells are always used to analyze angiogenesis activity *in vivo* (Rocke et al., 2009). Accordingly, we treated the transgenic Tg (flk: EGFP) embryos with different doses of 7m (0.5, 1, 2.5, 5 μΜ), and 30 hpf zebrafish was imaged by fluorescent microscopy to examine the effect on intersegmental vessel (ISV) development. As shown in Figure 10, 7m slightly reduced the number and length of SIVs at 0.5 and 1 μΜ in comparison with the vehicle control group. When the concentration of 7m was elevated to 2.5 μΜ, the angiogenic effect was more pronounced as evidenced by the 3.5- to 4-fold reduction in the number of SIVs. At doses ≥5 μM, ISVs formations were seriously impaired and developmental defects were observed. The result indicated that 7m suppresses the spouts of angiogenesis in zebrafish in a dose-dependent manner.

#### 2.2.11 Anti-Tumor and -Metastasis Efficacy of 7m in Zebrafish Xenograft Assay

**FIGURE 11:**
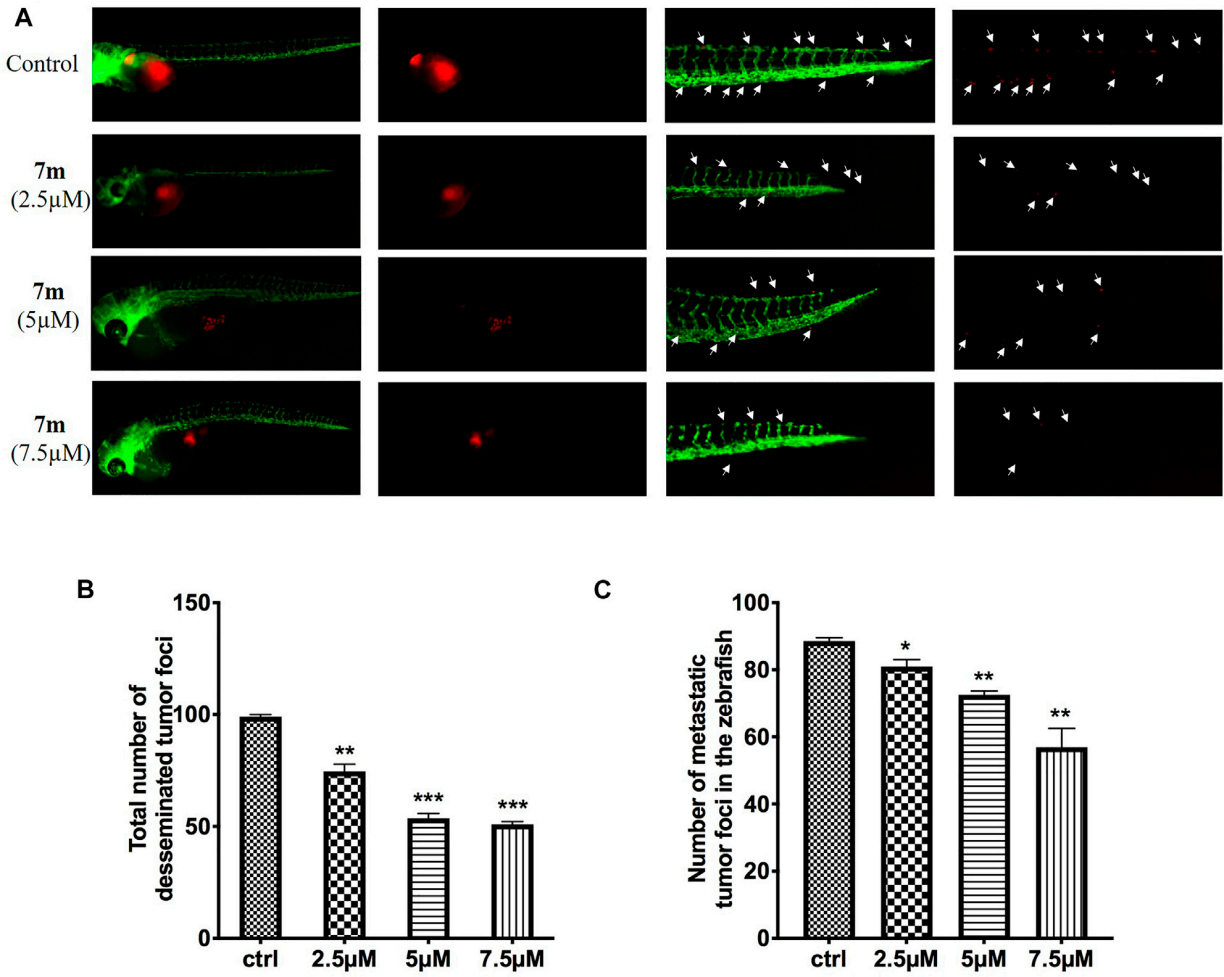
**(A)** Inhibitory effects of 7m on the proliferation and metastasis of MCF-7 cells in zebrafish xenograft models. CMTPX-labeled MCF-7 cells (red) were microinjected into zebrafish embryos, and indicated concentrations of 7m were added. **(B)** Quantification of the fluorescent area of the tumor xenografts, representing total MCF-7 cells in zebrafish. **(C)** Fluorescence intensity of the tumor xenografts in trunk.

To examine the *in vivo* anti-tumor and anti-metastasis potency of 7m, we implanted Cell-Tracker Red CMTPX-labeled MCF-7 cells into the yolk of Tg (flk: EGFP) 48 hpf embryos, and different concentrations of 7m (2.5, 5, 7.5 μΜ) were added. The intensity of the red fluorescence is proportional to the size of the xenograft tumor. After 72 h, the effects of 7m on the tumor mass development and cancer cell metastasis were assessed by fluorescence microscopy. As shown in Figure 11, 7m treatment reduced the fluorescence intensity and tumor size of MCF-7 cells compared with the vehicle group. Without drug treatment, MCF-7 cells disseminated and migrated widely away from the original area, and some tumor cells even migrated into the blood vessels of the zebrafish tail at 72 hpf. However, after treatment with 7m, the migration number of the implanted MCF-7 cells is notably reduced in a dose-dependent manner. On the other hand, the proliferation of MCF-cells is also inhibited by 7m as reflected in the decrease in tumor volume. Together, these results implied that 7m possesses a strong inhibitory effect against the migration and invasion of breast cancer.

**FIGURE 12:**
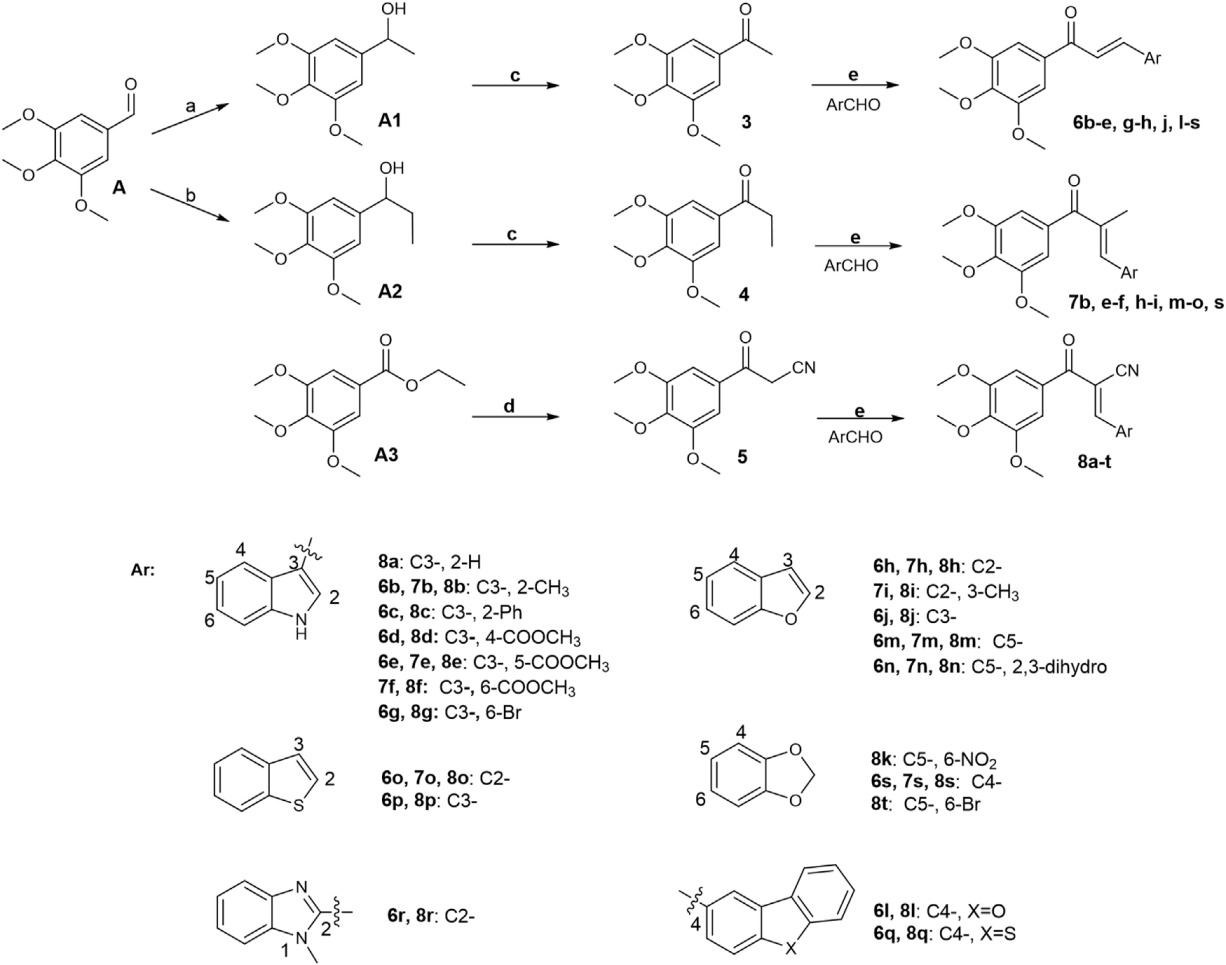
Reagents and conditions: **(A)** CH_3_MgBr, THF, 0°C–25°C; H_2_O, 81%. **(B)** CH_3_CH_2_MgBr, THF, 0°C–25°C; H_2_O, 50%. **(C)** PCC, CH_2_Cl_2_, 0°C–25°C; H_2_O, 74%. **(D)** CH_3_CN-THF (1:1), NaH, reflux, 81%. **(E)** MeOH or ethanol or THF or Dioxane, appropriate base, 0°C–75°C, 45%–95%.

## 3 Conclusion

The results presented in Tables 1 and 2 reveals that incorporation of a methyl group at the α-position leads to a markedly increased cytotoxicity activity in comparison with that of α-CN and α-unsubstituted chalcones. The chalcone hybrid 7m bearing a benzofuran ring in B-ring is most potent in 44 benzoheterocycle analogs. Molecular mechanisms illustrated that compound 7m could affect the expression levels of apoptosis-related proteins against MCF-7 cells. Furthermore, a mechanistic study has shown that 7m arrested the cell cycle of MCF-7 in the G2/M phase, which might derive from the binding to tubulin and interfere with microtubule dynamics. In addition, 7m obviously inhibited MCF-7 cell growth and migration *in vitro* and in a zebrafish xenograft model. More importantly, it reduced angiogenesis in zebrafish xenografts and showed remarkable activity in HUVEC tube formation, Transwell Matrigel, and wound healing assays. Taken together, preliminary observations indicated that compound 7m is a new type of potential tubulin polymerization inhibitor, which is worthy of further research for the treatment of cancer.

## 4 Experimental Procedures

### 4.1 Biological Section

The primary tumor cells were purchased from a professional biomedical company. First, the primary tumor cells were incubated in a cell culture incubator at 37°C and 5% CO_2_. After the cell fusion reached 85%–95%, subculture was performed, and the tumor cells obtained at this time were used for subsequent related experiments. The CCK-8 method was used to inhibit the proliferation of U937 tumor cells, and the SRB method was used to inhibit the proliferation of MCF7 and HepG2 tumor cells.

#### 4.1.1 SRB

Cells were distributed into 96-well plates with a density of 3 × 10^3^–4 × 10^3^ cells per well and incubated for 24 h in a 5% CO_2_ incubator at 37°C, followed by treatment with study compounds at different concentrations at 37°C for 72 h. One hundred microliters of 10% (w/v) trichloroacetic acid was added to each well, fixed at 4°C for 30 min, and dried at 65°C. Then 100 μl of 0.4% SRB was added to each well and stained on a shaker for 20 min at room temperature. The excess dye solution was washed with newly prepared 1% glacial acetic acid and dried at 65°C. One hundred fifty microliters of 10 mM unbuffered Trisbase was added to each well of the plate and shaken for 5 min to dissolve the dye. The microplate reader measured the optical density (OD) at a wavelength of 560 nm. When the inhibition rate was 50%, the concentration of the compound was calculated by the Logit method.

#### 4.1.2 CCK-8

The cells in the logarithmic growth phase were seeded in a 96-well plate at 8,000/well, and the drug-containing medium and cell suspension were added to the well. Three wells were added with the blank medium as the blank group. The 96-well plate was incubated for 72 h in a 37°C, 5% CO_2_ incubator. The 10 μl of CCK-8 dye solution was added to each well, and the incubation continued for 3–4 h. The microplate reader measured the absorbance at 450 nm wavelength, and the calculation formula for the proliferation inhibition rate was inhibition rate (%) = (OD control well − OD dosing well)/OD control well × 100%.

#### 4.1.3 Effects on tubulin Polymerization

An *in vitro* assay was performed to monitor the time-dependent polymerization of tubulin into microtubules. The 96-well cell culture plate was incubated at 37°C for 10 min, and test compounds and reference compounds were prepared with a 10× analyte stock solution. Five microliters of 10× analyte stock solution was added to a 96-well plate, and the microplate reader was heated at 37°C for 1 min. Immediately 85 μl of Tubulin Stock was drawn and mixed with the above mixture to prepare the tubulin reaction solution, and 50 μl of the tubulin reaction solution was to each well. The multifunctional microplate reader measured the absorbance, and the measured fluorescence value represents the level of tubulin in the polymerized state in the reaction solution.

#### 4.1.4 Immunofluorescent Staining

After being cultured in 24-well cell culture plates, MCF-7 cells were treated with 0.1, 0.2, and 0.4 μM compound 7m, CA-4, and vehicle control (DMSO) for 24 h. Then the cells were washed with PBS three times. After permeabilization with 0.25% Triton X-100 for 5 min, the cells were washed with PBS three times and blocked in 5% bovine serum albumin (BSA) for 40 min. The cells were incubated with rabbit anti-α-tubulin primary antibody containing 5% BSA at room temperature for 5–6 h and washed with PBS three times. Cells were finally visualized under a fluorescence microscope.

#### 4.1.5 Cell Cycle Analysis

MCF-7 cells were seeded into six-well plates (5 × 10^5^ cells/well) and incubated at 37°C in a humidified 5% CO_2_ incubator for 24 h, and then treated with or without compound 7m at indicated concentrations (100, 200, 400 nM). The collected cells were fixed by adding 70% of ethanol at −20°C for 12 h. The cells were then washed with PBS, incubated with 50 mg/ml of RNase at 37°C for 30 min, and stained with 12.5 mg/ml of propidium iodide for 15 min, and finally subjected to flow cytometry.

#### 4.1.6 Apoptosis Analysis

The MCF-7 cells in 12-well plates (8 × 10^4^ cells/well) were cultured for 24 h at 37°C in 5% CO_2_ and then incubated with 7m at concentrations of 100, 200, 400 nM, or vehicle to induce cell apoptosis for 48 h. Then the cells floating in the supernatant were combined with the adherent fraction and washed with PBS three times. The cells were incubated with Annexin V-FITC and PI for 15 min at 37°C in the dark. Apoptosis was quantified using a flow cytometer (Becton, Dickinson and Company, USA).

#### 4.1.7 Western Blot Analysis

MCF-7 cells were seeded into 10-mm dishes at a density of 6 × 10^5^/dish and incubated for 24 h. After treating the cells with the compound 7m, the cells were cultured for 48 h and washed with PBS, and then the soluble and insoluble tubulin fractions were collected. Protein quantification was performed by BCA Protein Concentration Determination Kit following centrifugation at 12,000 rpm for 10 min at 4°C. Cell lysates were separated by SDS-PAGE at 80 V constant voltage for 30–40 min. When it was found that the protein was about to enter the lower layer of gel, the voltage was adjusted to 110 V, and the electrophoresis was continued for about 90 min and then transferred to PVDF membranes at 100 V for 90 min. After 5% skim milk was blocked for 1 h, the primary antibody was added and incubated overnight before being washed three times with PBST. The secondary antibody was incubated for 2 h at room temperature the next day and subsequently washed three times with PBST. Then the signal was detected with ECL.

#### 4.1.8 Wound Healing Assays

MCF-7 cells and HUVECs cells were grown in 24-well plates at a density of 1 × 10^6^ cells per well for 24 h. Scratches were made in confluent monolayers by 200-ml pipette tips. The media containing different concentrations of 7m were added to the Petri dishes. Cells which migrated across the wound area were photographed using phase-contrast microscopy at 0 and 24 h. The migration distance of cells migrated to the wound area was measured manually.

#### 4.1.9 Transwell Cell Invasion Experiment

The upper chamber of the Transwell chamber was filled with cells resuspended in serum-free medium, 6 × 10^4^ cells per well, 100 μl per well, and the lower chamber was added with 10% FBS-containing medium prepared with different concentrations of 7m. The cells were allowed to migrate for 48 h at 37°C in an atmosphere of 5% CO_2_. Then the migration of cells to the underside of the filter was determined by crystal violet staining.

#### 4.1.10 Tube Formation Assay

The HUVECs were seeded in 96-well plates and cultured for 24 h, and then incubated with 1, 5, and 25 μM 7m. The EC Matrigel matrix was thawed overnight at 4°C. The HUVECs, suspended in F12K, were seeded in a 96-well culture plate at a cell density of 10,000 cells/well after 45 min of Matrigel polymerization at 37°C. Then they were treated with different concentrations of 7m or carrier at 37°C for 6–8 h. Then the morphological changes of the formed cells and tubes under an inverted microscope were observed, and pictures were taken.

#### 4.1.11 Anti-Angiogenic Effect on Zebrafish Embryos

The transgenic zebrafish Tg (flk: EGFP) embryos were paired normally and collected into a six-well plate (*n* = 30/well), then 1 ml of aquaculture water (0.2 g/L distilled water instant sea salt) was added and were raised at 28.5°C. When the zebrafish embryos developed to 12 hpf, the indicated concentrations of 7m were added, and 30 embryos were selected for each concentration gradient and incubated at 28.5°C to 30 hpf. At 30 hpf, the zebrafish embryos were imaged and photographed under a fluorescence microscope to observe the growth of blood vessels in the body, and the number of intersegmental blood vessels (ISV) was recorded.

#### 4.1.12 Zebrafish Xenograft Assay

The tumor cells MCF-7 were labeled with Red CMTPX, and then the labeled tumor cells were digested and resuspended. A hundred labeled tumor cells per 1 nl were injected into the zebrafish embryos developed for 48 h. The zebrafish injected with tumor cells were incubated in an incubator at 28.5°C for about 1 h under the action of different concentrations of 7m and then were transferred to an incubator at 35°C. The tumor cells were observed with a fluorescence microscope when the zebrafish developed to 72 h, and photos were taken to record them.4.2 Chemistry (the detailed information is in the Supplementary material)

Unless otherwise stated, all chemicals were purchased from commercial suppliers and used without further purification. Solvents and reagents were dried and purified by the methods described in the literature. Column chromatography (CC) was performed on silica gel (200–300 mesh, Qingdao Ocean Chemical Company, China). Thin layer chromatography (TLC) analysis was performed on silica gel GF254 (Qingdao Ocean Chemical Company, China) glass plates. ^1^HNMR and ^13^CNMR were recorded as deuterated chloroform (CDCl_3_) or deuterated dimethyl sulfoxide (DMSO-d_6_) solutions on Bruker AV-400 (400 MHz) nuclear magnetic resonance spectrometer at 400 and 100 MHz, respectively. Chemical shifts were reported in parts per million (ppm) relative to tetramethylsilane as an internal standard. A Q-TOF mini mass spectrometer was used to record high-resolution mass spectra (HRMS).

#### 4.1.13 General Procedure for Synthesis of Compounds 6b-e, g

Compound 3 (1.0 eq) was dissolved by adding anhydrous EtOH, then piperidine (1.2 eq), and the corresponding aromatic aldehyde (0.8 eq) was added. The reaction mixture was allowed to reflux. After the reaction of the raw materials was completed, the reaction mixture was cooled to room temperature, and the solid was precipitated. The solid was directly filtered, washed with cooled ethanol, and dried to obtain the corresponding target compounds 6b-e, g.

(E)-3-(2-methyl-1H-indol-3-yl)-1-(3,4,5-trimethoxyphenyl)prop-2-en-1-one (6b): yield 81%, yellow solid, m. p. 236.7°C–238.2°C;^1^H NMR (400 MHz, DMSO-d_6_) δ 11.81 (s, 1H), 8.06 (d, J = 15.3 Hz, 1H), 8.03–8.01 (m, 1H), 7.50 (d, J = 15.3 Hz, 1H), 7.41–7.39 (m, 1H), 7.35 (s, 2H), 7.23–7.17 (m, 2H), 3.92 (s, 6H), 3.77 (s, 3H), 2.60 (s, 3H);^13^C NMR (101 MHz, DMSO-d_6_) δ 187.89, 152.79, 143.90, 141.18, 137.65, 136.09, 134.31, 122.02, 121.16, 120.04, 114.35, 111.46, 109.15, 105.59, 60.13, 56.08, 11.93; IR (KBr): 3,248, 2,991, 2,831, 1,639, 1,549, 1,459, 1,128, 726 cm^−1^;HR-MS (ESI-TOF) Calcd for C_21_H_21_NO_4_ [M + H]^+^: 352.1549, found: 352.1543.

(E)-3-(2-phenyl-1H-indol-3-yl)-1-(3,4,5-trimethoxyphenyl)prop-2-en-1-one (6c): yield 83%, yellow solid, m. p. 60.2–61.7°C;^1^H NMR (400 MHz, Chloroform-d) δ 9.03 (s, 1H), 8.20 (d, J = 15.5 Hz, 1H), 8.08–8.01 (m, 1H), 7.63 (d, J = 15.5 Hz, 1H), 7.56 (dd, J = 7.6, 1.8 Hz, 2H), 7.49–7.42 (m, 4H), 7.35–7.30 (m, 2H), 7.28 (s, 2H), 3.93 (s, 3H), 3.91 (s, 6H); IR (KBr): 3,368, 2,924, 2,853, 1,644, 1,559, 1,454, 1,127 cm^−1^;HR-MS (ESI-TOF) Calcd for C_26_H_23_NO_4_ [M + H]^+^:414.1705, found: 414.1709.

methyl(E)-3[3-oxo-3-(3,4,5-trimethoxyphenyl)prop-1-en-1-yl]-1H-indole-4-carboxylate (6d): yield 79%, yellow solid, m. p. 221.7°C–223.6°C;^1^H NMR (400 MHz, DMSO-d_6_) δ 12.32 (s, 1H), 8.59–8.49 (m, 2H), 7.75 (d, J = 8.1 Hz, 1H), 7.67–7.59 (m, 2H), 7.40 (s, 2H), 7.28 (t, J = 7.8 Hz, 1H), 3.92 (s, 9H), 3.77 (s, 3H);^13^C NMR (101 MHz, DMSO-d_6_) δ 187.77, 168.16, 152.80, 141.27, 140.28, 138.02, 133.85, 130.30, 123.59, 123.26, 121.29, 116.81, 116.54, 112.58, 105.76, 60.12, 56.08, 51.94; IR (KBr): 3,211, 1712, 1,547, 1,356, 1,100 cm^−1^; HR-MS (ESI-TOF) Calcd for [M + H]^+^ C_22_H_21_NO_6_:396.1447, found: 396.1442.

methyl(E)-3[3-oxo-3-(3,4,5-trimethoxyphenyl)prop-1-en-1-yl]-1H-indole-5-carboxylate (6e): yield 78%, yellow solid, m. p. 218.7°C–220.3°C;^1^H NMR (400 MHz, DMSO-d_6_) δ 12.25 (s, 1H), 8.65–8.62 (m, 1H), 8.29 (s, 1H), 8.05 (d, J = 15.5 Hz, 1H), 7.86 (dd, J = 8.6, 1.5 Hz, 1H), 7.72 (d, J = 15.5 Hz, 1H), 7.59 (d, J = 8.6 Hz, 1H), 7.38 (s, 2H), 3.93 (s, 6H), 3.88 (s, 3H), 3.78 (s, 3H);^13^C NMR (101 MHz, DMSO-d_6_) δ187.57, 166.90, 152.82, 141.35, 139.92, 137.31, 133.68, 133.44, 125.01, 123.44, 122.30, 121.87, 116.83, 113.54, 112.50, 105.50, 60.13, 55.93, 51.84; IR (KBr): 3,370, 1701, 1,568, 1,265, 1,122 cm^−1^;HR-MS (ESI-TOF) Calcd for [M + H]^+^ C_22_H_21_NO_6_: 396.1447, found: 396.1442.

(E)-3-(6-bromo-1H-indol-3-yl)-1-(3,4,5-trimethoxyphenyl)prop-2-en-1-one (6g): yield 85%, orange solid, m.p. 216.4°C–217.6°C;^1^H NMR (400 MHz, DMSO-d_6_) δ 12.01 (s, 1H), 8.19 (s, 1H), 8.07–7.99 (m, 2H), 7.71–7.62 (m, 2H), 7.38 (s, 2H), 7.34 (dd, J = 8.5, 1.7 Hz, 1H), 3.92 (s, 6H), 3.77 (s, 3H);^13^C NMR (101 MHz, DMSO-d_6_) δ 187.84, 152.76, 141.35, 138.16, 137.75, 133.78, 133.04, 124.29, 123.73, 121.86, 116.40, 115.13, 114.90, 112.74, 105.75, 60.08, 56.07; IR (KBr): 3,282, 1,648, 1,556, 1,413, 1,122, 620 cm^−1^;HR-MS (ESI-TOF) Calcd for [M + H]^+^ C_20_H_18_BrNO_4_: 416.0497, found: 416.0492

#### 4.1.14 General Procedure for Synthesis of Compound 6h

Compound 3 (1.0 eq) was dissolved by adding anhydrous THF, and then NaOH (6.0 eq) was added. After the benzofuran-2-carbaldehyde (1.6 eq) was dissolved by adding 1 ml of anhydrous THF, it was added dropwise to the above reaction mixture at 0°C, and then the reaction mixture was stirred at room temperature. After the reaction of the raw materials was completed, the reaction mixture was added an appropriate amount of silica gel to evaporate the solvent, and was purified by silica-gel column chromatography (V_PET_:V_EtOAc_ = 10:1) to obtain the target compound 6h.

(E)-3-(benzofuran-2-yl)-1-(3,4,5-trimethoxyphenyl)prop-2-en-1-one (6h): yield 65%, light yellow solid, m. p. 79.5°C–80.3°C; ^1^H NMR (400 MHz, Chloroform-d) δ 7.75–7.66 (t, J = 15.2, 12.8 Hz, 2H), 7.62 (d, J = 8.6 Hz, 1H), 7.54 (d, J = 8.3 Hz, 1H), 7.42–7.37 (m, 1H), 7.35 (s, 2H), 7.30–7.27 (m, 1H), 7.06 (s, 1H), 3.99 (s, 6H), 3.96 (s, 3H);^13^C NMR (101 MHz, Chloroform-d) δ 188.25, 155.63, 153.22, 153.05, 142.74, 133.27, 130.87, 128.57, 126.79, 123.48, 121.93, 121.50, 112.63, 111.40, 106.15, 61.03, 56.48; IR (KBr): 3,429, 1,654, 1,598, 1,581, 1,335, 1,126, 743 cm^−1^;HR-MS (ESI-TOF) Calcd for [M + H]^+^ C_20_H_18_O_5_: 339.1232, found: 339.1236.

#### 4.1.15 General Procedure for Synthesis of Compounds 6j, 6o-q

Compound 3 (0.8 eq) was dissolved in freshly prepared MeONa solution, and the corresponding aromatic aldehyde (1.0 eq) was added. The reaction mixture was stirred at room temperature. After the reaction of the raw materials was completed, the reaction mixture was added an appropriate amount of silica gel to evaporate the solvent, and was purified by silica-gel column chromatography (V_PET_:V_EtOAc_ = 15:1) to obtain the target compounds 6j, 6o-q.

(E)-3-(benzofuran-3-yl)-1-(3,4,5-trimethoxyphenyl)prop-2-en-1-one (6j): yield 57%, yellow solid, m. p. 117.5°C–120.8°C; ^1^H NMR (400 MHz, Chloroform-d) δ 8.02–7.90 (m, 3H), 7.64–7.56 (m, 2H), 7.44–7.39 (m, 2H), 7.30 (s, 2H), 3.97 (s, 6H), 3.95 (s, 3H); ^13^C NMR (101 MHz, Chloroform-d) δ 189.24, 156.25, 153.24,148.61, 142.66, 134.50, 133.52, 125.53, 124.90, 123.90, 122.15, 120.91, 118.56, 112.21, 106.24, 61.00, 56.48; IR (KBr): 3,430, 1,668, 1,614, 1,583, 1,342, 1,127, 731 cm^−1^; HR-MS (ESI-TOF) Calcd for [M + H]^+^ C_20_H_18_O_5_: 339.1232, found: 339.1236.

(E)-3-(benzo[b]thiophen-2-yl)-1-(3,4,5-trimethoxyphenyl)prop-2-en-1-one (6o): yield 71%, yellow solid, m. p. 115.5°C–116.9°C; ^1^H NMR (400 MHz, Chloroform-d) δ 8.03 (d, J = 15.2 Hz, 1H), 7.83–7.76 (m, 2H), 7.57 (s, 1H), 7.43–7.31 (m, 3H), 7.28 (s, 2H), 3.96 (s, 6H), 3.95 (s, 3H).^13^C NMR (101 MHz, Chloroform-d) δ 183.03,147.90, 137.40, 134.92, 134.40, 132.27, 127.95, 124.58, 121.21, 119.69, 119.27, 117.43, 117.17, 100.81, 55.71, 51.13; IR (KBr): 3,050, 1,654, 1,574, 1,131, 721 cm^−1^;HR-MS (ESI-TOF) Calcd for [M + H]^+^ C_20_H_18_O_4_S: 355.1004, found: 355.1007.

(E)-3-(benzo[b]thiophen-3-yl)-1-(3,4,5-trimethoxyphenyl)prop-2-en-1-one (6p): yield 72%, yellow solid, m. p. 95.5°C–96.4°C;^1^H NMR (400 MHz, Chloroform-d) δ 8.13 (d, J = 15.7 Hz, 1H), 8.09 (d, J = 7.9 Hz, 1H), 7.93 (d, J = 9.7 Hz, 2H), 7.60 (d, J = 15.7 Hz, 1H), 7.54–7.49 (m, 1H), 7.48–7.42 (m, 1H), 7.33 (s, 2H), 3.97 (d, J = 2.5 Hz, 9H);^13^C NMR (101 MHz, Chloroform-d) δ 189.18, 153.19, 142.55, 140.54, 137.39, 136.22, 133.51, 132.35, 128.39, 125.18, 125.08, 123.10, 122.37, 122.10, 106.13, 61.00, 56.42; IR (KBr): 3,421, 3,071, 1,650, 1,571, 1,334, 1,128, 730 cm^−1^;HR-MS (ESI-TOF) Calcd for [M + H]^+^ C_20_H_18_O_4_S: 355.1004, found: 355.1007.

(E)-3[dibenzo (b,d)thiophen-2-yl]-1-(3,4,5-trimethoxyphenyl)prop-2-en-1-one (6q): yield 78%, yellow solid, m. p. 118.7°C–119.2°C;^1^H NMR (400 MHz, Chloroform-d) δ 8.36 (s, 1H), 8.24–8.19 (m, 1H), 8.00 (d, J = 15.6 Hz, 1H), 7.92–7.85 (m, 2H), 7.78 (dd, J = 8.4, 1.5 Hz, 1H), 7.59 (d, J = 15.6 Hz, 1H), 7.53–7.48 (m, 2H), 7.32 (s, 2H), 3.98 (s, 6H), 3.96 (s, 3H);^13^C NMR (101 MHz, Chloroform-d) δ 189.19, 153.21, 144.92, 142.56, 141.92, 139.82, 136.19, 135.02, 133.64, 131.41, 127.34, 125.93, 124.81, 123.24, 123.00, 122.31, 121.76, 121.35, 106.19, 61.02, 56.48; IR (KBr): 2,924, 1,654, 1,597, 1,575, 1,341, 1,126, 758 cm^−1^;HR-MS (ESI-TOF) Calcd for [M + H]^+^ C_24_H_20_O_4_S: 405.1161, found: 405.1166.

#### 4.1.16 General Procedure for Synthesis of Compounds 6l-m

Compound 3 (0.8 eq) and the corresponding aromatic aldehyde (generally 1.0 eq) were placed in a 25-ml round-bottomed flask, and then 10% of KOH methanol solution was added to the reaction mixture and stirred at room temperature. After 8 h, the reaction mixture was quenched with 10% HCl and extracted with EtOAc three times. The organic phase was dried and concentrated with anhydrous MgSO_4_, and the target compounds 6l-m were obtained by purification on silica-gel column chromatography.

(E)-3-[dibenzo (b,d)furan-2-yl]1-(3,4,5-trimethoxyphenyl)prop-2-en-1-one (6l): yield 75%, light yellow solid, m. p. 56.5°C–57.6°C; ^1^H NMR (400 MHz, Chloroform-d) δ 8.23 (s, 1H), 8.04–7.96 (m, 2H), 7.79 (dd, J = 8.6, 1.7 Hz, 1H), 7.61 (dd, J = 8.4, 4.9 Hz, 2H), 7.57–7.47 (m, 2H), 7.39 (t, J = 7.6 Hz, 1H), 7.32 (s, 2H), 3.98 (s, 6H), 3.96 (s, 3H); IR (KBr): 2,935, 1,653, 1,573, 1,124, 814 cm^−1^; HR-MS (ESI-TOF) Calcd for [M + H]^+^ C_24_H_20_O_5_: 389.1389, found: 389.1384.

(E)-3-(benzofuran-5-yl)-1-(3,4,5-trimethoxyphenyl)prop-2-en-1-one (6m): yield 72%, light yellow solid, m. p. 110.5°C–115.3°C; ^1^H NMR (400 MHz, Chloroform-d) δ 7.94 (d, J = 15.6 Hz, 1H), 7.88 (s, 1H), 7.67 (d, J = 2.2 Hz, 1H), 7.64 (dd, J = 8.6, 1.6 Hz, 1H), 7.54 (d, J = 8.6 Hz, 1H), 7.49 (d, J = 15.6 Hz, 1H), 7.30 (s, 2H), 6.82 (dd, J = 2.1, 0.8 Hz, 1H), 3.96 (s, 6H), 3.95 (s, 3H); ^13^C NMR (101 MHz, Chloroform-d) δ 189.21, 156.25, 153.17, 146.19, 145.35, 142.44, 133.70, 130.02, 128.18, 124.60, 122.22, 120.66, 112.04, 106.82, 106.09, 61.00, 56.42; IR (KBr): 3,425, 1,652, 1,573, 1,331, 1,127, 802 cm^−1^; HR-MS (ESI-TOF) Calcd for [M + H]^+^ C_20_H_18_O_5_: 339.1232, found: 339.1237.

#### 4.1.17 General Procedure for Synthesis of Compounds 6n, 6s

The corresponding aromatic aldehyde (1.0 eq) was dissolved in MeOH, then sodium hydroxide (4.0 eq) and compound 3 (1.0 eq) were added. The reaction mixture was heated to reflux and stirred for 5 h and then cooled to room temperature. After the solid precipitated, it was filtered directly, washed with cold methanol, and dried to obtain the target compounds 6n, 6s.

(E)-3-(2,3-dihydrobenzofuran-5-yl)-1-(3,4,5-trimethoxyphenyl)prop-2-en-1-one (6n): yield 75%, light yellow solid, m. p. 107.5°C–110.3°C; ^1^H NMR (400 MHz, Chloroform-d) δ 7.79 (d, J = 15.5 Hz, 1H), 7.53 (s, 1H), 7.44 (d, J = 9.6 Hz, 1H), 7.34 (d, J = 15.5 Hz, 1H), 7.27 (s, 2H), 6.82 (d, J = 8.3 Hz, 1H), 4.65 (t, J = 8.7 Hz, 2H), 3.95 (s, 6H), 3.94 (s, 3H), 3.26 (t, J = 8.7 Hz, 2H); ^13^C NMR (101 MHz, Chloroform-d) δ 189.27, 162.65, 153.11, 145.15, 142.23, 133.93, 130.08, 128.21, 127.74, 125.02, 118.72, 109.84, 105.97, 71.95, 60.98, 56.40, 29.26;

IR (KBr): 3,439, 1,650, 1,593, 1,572, 1,124, 814 cm^−1^;HR-MS (ESI-TOF) Calcd for [M + H]^+^ C_20_H_20_O_5_: 341.1389, found: 341.1385.

(E)-3-(benzo[d][1,3]dioxol-4-yl)-1-(3,4,5-trimethoxyphenyl)prop-2-en-1-one (6s): yield 87%, yellow solid, m. p. 138.4°C–140.3°C;^1^H NMR (400 MHz, Chloroform-d) δ 7.74 (d, J = 15.7 Hz, 1H), 7.68 (d, J = 15.7 Hz, 1H), 7.28 (s, 2H), 7.03 (dd, J = 6.3, 2.8 Hz, 1H), 6.89–6.85 (m, 2H), 6.11 (s, 2H), 3.95 (s, 6H), 3.94 (s, 3H);^13^C NMR (101 MHz, Chloroform-d) δ 189.52, 153.13, 148.01, 146.72, 142.54, 139.08, 133.51, 124.44, 123.20, 121.96, 118.02, 110.01, 106.25, 101.50, 60.99, 56.42; IR (KBr): 3,423, 1,660, 1,604, 1,583, 1,452, 1,131, 768 cm^−1^;HR-MS (ESI-TOF) Calcd for [M + H]^+^ C_19_H_18_O_6_: 343.1182, found: 343.1187.

#### 4.1.18 General Procedure for Synthesis of Compound 6r

Compound 3 (1.0 eq) and 1-methyl-1H-benzo [d]imidazole-2-carbaldehyde (usually 1.2 eq) were placed in a 25-ml round-bottomed flask, anhydrous dioxane was added and BF_3_. Et_2_O (5.0 eq) solution was slowly added dropwise under Ar protection. Then the reaction mixture was heated to 75°C with stirring. After the reaction of the raw materials was completed, the reaction mixture was added with H_2_O, 1 M NaOH, and extracted three times with EtOAc. The organic phase was dried with anhydrous MgSO_4_ and then concentrated. Silica gel column chromatography was used to obtain the target compound 6r.

(E)-3-[1-methyl-1H-benzo (d)imidazol-2-yl]-1-(3,4,5-trimethoxyphenyl)prop-2-en-1-one (6r): yield 57%, yellow solid, m. p. 123.5°C–125.2°C;^1^H NMR (400 MHz, Chloroform-d) δ 8.36 (d, J = 14.9 Hz, 1H), 7.91 (d, J = 14.9 Hz, 1H), 7.82 (dd, J = 6.1, 2.7 Hz, 1H), 7.42 (s, 2H), 7.41–7.32 (m, 3H), 3.98 (s, 6H), 3.97 (s, 3H), 3.96 (s, 3H);^13^C NMR (101 MHz, Chloroform-d) δ 187.76, 153.29, 148.60, 143.14, 136.36, 132.69, 127.87, 127.54, 124.18, 123.60, 120.14, 109.87, 106.33, 61.04, 56.51, 30.06; IR (KBr): 3,411, 1,659, 1,586, 1,345, 1,127, 748 cm^−1^;HR-MS (ESI-TOF) Calcd for [M + H]^+^ C_20_H_20_N_2_O_4_: 353.1501, found: 353.1505.

#### 4.1.19 General Procedure for Synthesis of Compounds 7b, 7e-f

Compound 4 (1.0 eq) was dissolved by adding anhydrous EtOH, then piperidine (1.2 eq), 4A molecular sieve, and the corresponding aromatic aldehyde (0.8 eq) were added. The temperature was increased, and the reaction was stirred under reflux conditions. After the raw material reaction was completed, 1 M HCl was added to the reaction mixture to adjust pH = 6, extracted three times with EtOAc, the anhydrous MgSO_4_ in the organic phase was dried, and then concentrated. The organic phase was purified by silica-gel column chromatography (VPET:VEtOAc = 10:1–2:1) to obtain the target compounds 7b, 7e-f.

(E)-2-methyl-3-(2-methyl-1H-indol-3-yl)-1-(3,4,5-trimethoxyphenyl)prop-2-en-1-one (7b): yield 66%, yellow solid, m. p. 152.7°C–153.2°C; 1H NMR (400 MHz, Chloroform-d) δ 8.24 (s, 1H), 7.47 (d, J = 7.3 Hz, 1H), 7.40 (s, 1H), 7.33 (d, J = 8.3 Hz, 1H), 7.21–7.13 (m, 2H), 7.07 (s, 2H), 3.93 (s, 3H), 3.91 (s, 6H), 2.40 (s, 3H), 2.14 (d, J = 1.1 Hz, 3H);^13^C NMR (101 MHz, Chloroform-d) δ 198.44, 152.79, 141.02, 136.34, 135.48, 135.29, 134.81, 134.48, 127.33, 121.96, 120.33, 119.64, 110.61, 109.79, 107.00, 60.97, 56.27, 15.90, 13.29; IR (KBr): 3,216, 1,589, 1,574, 1,234, 1,131 cm^−1^; HR-MS (ESI-TOF) Calcd for [M + H]^+^ C_22_H_24_O_4_: 343.1705, found: 343.1709.

methyl(E)-3-(2-methyl-3-oxo-3-(3,4,5-trimethoxyphenyl)prop-1-en-1-yl)-1H-indole-5-carboxylate (7e): yield 72%, yellow solid, m. p. 210.4°C–213.7°C;^1^H NMR (400 MHz, Chloroform-d) δ 8.97 (s, 1H), 8.36 (s, 1H), 7.99 (dd, J = 8.6, 1.5 Hz, 1H), 7.67 (d, J = 2.0 Hz, 2H), 7.46 (d, J = 8.9 Hz, 1H), 7.08 (s, 2H), 3.98 (s, 3H), 3.93 (s, 9H), 2.32 (s, 3H);^13^C NMR (101 MHz, Chloroform-d) δ 197.82, 167.68, 152.93, 141.26, 138.12, 133.98, 133.15, 132.90, 127.28, 126.90, 124.67, 123.06, 121.38, 114.35, 111.26, 107.26, 61.01, 56.32, 52.01, 15.68; IR (KBr): 3,432, 3,252, 1707, 1,617, 1,586, 1,253, 1,123, 740 cm^−1^;HR-MS (ESI-TOF) Calcd for [M + H]^+^ C_23_H_23_NO_6_: 410.1604, found: 410.1608.

methyl(E)-3-(2-methyl-3-oxo-3-(3,4,5-trimethoxyphenyl)prop-1-en-1-yl)-1H-indole-6-carboxylate (7f): yield 58%, yellow solid, m. p. 229.3°C–231.7°C;^1^H NMR (400 MHz, DMSO-d_6_) δ 12.27 (s, 1H), 8.13 (dd, J = 8.6, 1.9 Hz, 2H), 7.72 (dd, J = 8.4, 1.5 Hz, 1H), 7.62–7.57 (m, 2H), 6.99 (s, 2H), 3.86 (s, 3H), 3.82 (s, 6H), 3.78 (s, 3H), 2.24 (s, 3H);^13^C NMR (101 MHz, DMSO-d_6_) δ 196.50, 166.84, 152.44, 140.08, 135.10, 134.09, 133.55, 131.38, 130.80, 130.63, 123.44, 120.92, 117.76, 113.92, 111.55, 106.62, 60.16, 56.00, 51.91, 15.14; IR (KBr): 3,433, 1,615, 1,365, 773 cm^−1^;HR-MS (ESI-TOF) Calcd for [M + H]^+^ C_23_H_23_NO_6_: 410.1604, found: 410.1608.

#### 4.1.20 General Procedure for Synthesis of Compound 7h

Benzo [b]furan-2-carboxaldehyde (1.0 eq) was dissolved by adding anhydrous MeOH, then compound 4 (0.7 eq) and NaH solid (10.0 eq) were added in batches at 0°C, then the reaction was stirred at room temperature. After the reaction of the raw materials was completed, the reaction mixture was added an appropriate amount of silica gel to evaporate the solvent, and was purified by silica-gel column chromatography (V_PET_:V_EtOAc_ = 15:1) to obtain the target compound 7h.

The compounds 7m and 7o were prepared from compound 3 following the same procedure except that the temperature as described in the preparation of compound 7h.

(E)-3-(benzofuran-2-yl)-2-methyl-1-(3,4,5-trimethoxyphenyl)prop-2-en-1-one (7h): yield 64%, yellow oily solid m. p. 50.1°C–53.2°C;^1^H NMR (400 MHz, Chloroform-d) δ 7.60 (d, J = 7.7 Hz, 1H), 7.50 (d, J = 8.2 Hz, 1H), 7.37–7.31 (m, 1H), 7.28–7.22 (m, 1H), 7.10 (d, J = 1.08 Hz, 1H), 7.01 (s, 2H), 6.96 (s, 1H), 3.94 (s, 3H), 3.89 (s, 6H), 2.48 (d, J = 0.92 Hz, 3H);^13^C NMR (101 MHz, Chloroform-d) δ 197.60, 155.31, 153.16, 141.48, 136.73, 133.14, 128.47, 128.28, 125.97, 123.35, 121.57, 111.39, 111.09, 106.97, 60.95, 56.25, 15.29; IR (KBr): 3,429, 1,583, 1,330, 1,223, 996, 750 cm^−1^;HR-MS (ESI-TOF) Calcd for [M + H]^+^ C_21_H_20_O_5_: 353.1389, found: 353.13869.

(E: Z = 2:1)3-(benzofuran-6-yl)-2-methyl-1-(3,4,5-trimethoxyphenyl)prop-2-en-1-one (7m): yield 45%, light yellow solid, ^1^H NMR (400 MHz, Chloroform-d) δ 7.64 (d, J = 1.6 Hz, 1H), 7.60 (d, J = 2.2 Hz, 1H), 7.30 (dd, J = 8.6 Hz, 1.7 Hz, 1H), 7.19 (s, 1H), 7.09 (s, 1H), 6.97 (s, 2H), 6.74 (dd, J = 2.2, 0.9 Hz, 1H),3.86 (s, 3H), 3.83 (s, 6H), 3.75 (s, 1H), 3.70 (s, 3H), 2.25 (d, J = 1.3 Hz, 3H), 2.12 (d, J = 1.6 Hz, 1H). HRMS(ESI-TOF) m/z [M + H] + calcd for C_21_H_20_O_5_: 353.1389, found 353.1384.

(E)-3-(benzo[b]thiophen-2-yl)-2-methyl-1-(3,4,5-trimethoxyphenyl)prop-2-en-1-one (7o): yield 49%, white solid, m. p. 81.4°C–83.2°C; ^1^H NMR (400 MHz, Chloroform-d) δ 7.86 (dd, J = 5.8, 3.3 Hz, 1H), 7.80 (dd, J = 5.8, 3.3 Hz, 1H), 7.46 (d, J = 4.4 Hz, 2H), 7.38 (dd, J = 6.0, 3.2 Hz, 2H), 7.00 (s, 2H), 3.95 (s, 3H), 3.90 (s, 6H), 2.43 (d, J = 0.84 Hz, 3H); ^13^C NMR (101 MHz, Chloroform-d) δ 197.78, 152.93, 141.51, 141.42, 138.97, 138.80, 135.60, 134.82, 133.32, 128.61, 125.63, 124.89, 124.19, 122.22, 106.98, 60.98, 56.34, 14.97; IR (KBr): 3,420, 1,581, 1,411, 1,118 cm^−1^;HR-MS (ESI-TOF) Calcd for [M + H]^+^ C_21_H_20_O_4_S: 369.1161, found: 369.1155.

#### 4.1.21 General Procedure for Synthesis of Compounds 7i, 7n, 7s

The corresponding aromatic aldehyde (1.0 eq) was dissolved by adding anhydrous MeOH, then compound 4 (0.7 eq) and Cs_2_CO_3_ (5.0 eq) were added, and then the reaction was stirred at room temperature. After the reaction of the raw materials was completed, the reaction mixture was added an appropriate amount of silica gel to evaporate the solvent, and was purified by silica-gel column chromatography (V_PET_:V_EtOAc_ = 15:1) to obtain the target compounds 7i, 7n, 7s.

(E)-2-methyl-3-(3-methylbenzofuran-2-yl)-1-(3,4,5-trimethoxyphenyl)prop-2-en-1-one (7i): yield 61%, yellow solid, m. p. 113.2°C–115.1°C;^1^H NMR (400 MHz, Chloroform-d) δ 7.52 (dd, J = 11.6, 7.9 Hz, 2H), 7.40–7.35 (m, 1H), 7.30–7.22 (m, 2H), 7.13–7.10 (m, 1H), 7.02 (s, 2H), 3.94 (s, 3H), 3.90 (s, 6H), 2.55 (s, 3H), 2.25 (s, 3H);^13^C NMR (101 MHz, Chloroform-d) δ 197.95, 154.96, 152.90, 149.23, 141.44, 134.93, 133.50, 129.20, 126.19, 125.76, 122.87, 119.86, 111.31, 107.10, 60.97, 56.31, 15.06, 8.69; IR (KBr): 3,441, 1,613, 1,324, 1,128, 747 cm^−1^;HR-MS (ESI-TOF) Calcd for [M + H]^+^ C_22_H_22_O_5_: 367.1545, found: 367.1549.

(E)-3-(2,3-dihydrobenzofuran-5-yl)-2-methyl-1-(3,4,5-trimethoxyphenyl)prop-2-en-1-one (7n): yield 60%, yellow solid, m. p. 99.7°C–100.5°C;^1^H NMR (400 MHz, Chloroform-d) δ 7.34 (s, 1H), 7.25 (s, 1H), 7.17 (s, 1H), 6.98 (s, 2H), 6.83 (d, J = 8.3 Hz, 1H), 4.63 (t, J = 8.7 Hz, 2H), 3.92 (s, 3H), 3.89 (s, 6H), 3.25 (t, J = 8.7 Hz, 2H), 2.28 (d, J = 1.2 Hz, 3H);^13^C NMR (101 MHz, Chloroform-d) δ 198.70, 160.82, 152.81, 142.44, 141.11, 134.01, 130.93, 128.39, 127.64, 126.69, 109.48, 107.02, 71.74, 60.96, 56.32, 29.49, 14.76; IR (KBr): 3,439, 1,599, 1,580, 1,330, 1,130 cm^−1^;HR-MS (ESI-TOF) Calcd for [M + H]^+^ C_21_H_22_O_5_: 355.1545, found: 355.1548.

(E)-3-(benzo[d][1,3]dioxol-4-yl)-2-methyl-1-(3,4,5-trimethoxyphenyl)prop-2-en-1-one (7s): yield 80%, white solid, m. p. 119.7°C–120.9°C;^1^H NMR (400 MHz, Chloroform-d) δ 7.16 (s, 1H), 7.07 (s, 2H), 6.96 (d, J = 7.5 Hz, 1H), 6.88 (t, J = 7.8 Hz, 1H), 6.82 (dd, J = 7.7, 1.2 Hz, 1H), 5.98 (s, 2H), 3.93 (s, 3H), 3.91 (s, 6H), 2.22 (d, J = 1.3 Hz, 3H);^13^C NMR (101 MHz, Chloroform-d) δ 197.99, 152.82, 147.48, 145.84, 141.55, 137.81, 133.85, 133.01, 121.92, 121.52, 118.18, 108.74, 107.29, 100.98, 60.95, 56.25, 14.99; IR (KBr): 3,419, 1,585, 1,450, 1,329, 1,128, 999, 771 cm^−1^;HR-MS (ESI-TOF) Calcd for [M + H]^+^ C_21_H_20_O_6_: 357.1338, found: 357.1333.

#### 4.1.21 General Procedure for Synthesis of Compounds 8a-t

Compound 5 (1.0 eq) was added to 5 ml of anhydrous toluene, then the corresponding aromatic aldehyde (usually 0.7 eq) was added, and piperidine (0.78 eq) and glacial acetic acid (0.82 eq) were quickly added dropwise to the solution. Then the reaction mixture was transferred to 60°C and heated and stirred for 3–12 h. After the reaction of the raw materials was completed, the system was cooled to room temperature, and most of the reaction mixtures had solid precipitation (some reaction mixtures were still clear solutions, then the reaction mixture was transferred to ice water and stirred for 30 min to precipitate the solids). The reaction mixture was directly filtered to obtain the solid and then washed with cooled toluene and dried to obtain the corresponding target compounds 8a-t.

(E)-3-(1H-indol-3-yl)-2-(3,4,5-trimethoxybenzoyl)acrylonitrile (8a): yield 35%, white solid, m. p. 224.2°C–229.3°C; 1H NMR (400 MHz, Chloroform-d) δ 9.24 (s, 1H), 8.79 (d, J = 3.2 Hz, 1H), 8.70 (s, 1H), 7.84 (dd, J = 6.3, 2.2 Hz, 1H), 7.51 (dd, J = 6.3, 2.2 Hz, 1H), 7.40–7.33 (m, 2H), 7.23 (s, 2H), 3.96 (s, 3H), 3.94 (s, 6H); 13C NMR (101 MHz, Chloroform-d) δ 153.01, 147.71, 135.68, 130.93, 127.70, 124.51, 122.93, 118.45, 112.23, 112.05, 106.76, 102.22, 61.04, 56.37;

IR (KBr): 3,307, 2,925, 2,217, 1,334, 1,232, 1,126, 749 cm^−1^; HR-MS (ESI-TOF) Calcd for [M + H]^+^ C_21_H_18_N_2_O_4_: 363.1345, found: 363.1349.

(E)-3-(2-methyl-1H-indol-3-yl)-2-(3,4,5-trimethoxybenzoyl)acrylonitrile (8b): yield 87%, yellow solid, m. p. 216.5°C–218.2°C; 1H NMR (400 MHz, Chloroform-d) δ 8.76 (s, 1H), 8.53 (s, 1H), 8.22 (dd, J = 6.8, 1.5 Hz, 1H), 7.38–7.35 (m, 1H), 7.31 (td, J = 7.0, 1.5 Hz, 2H), 7.25 (s, 2H), 3.95 (s, 3H), 3.95 (s, 6H), 2.68 (s, 3H);

IR (KBr): 3,299, 2,214, 1,561, 1,215, 1,127, 757 cm-1; HR-MS (ESI-TOF) Calcd for [M + H]^+^ C_22_H_20_N_2_O_4_: 377.1501, found: 377.1506.

(E)-2-((l2-azaneylidene)-l3-methyl)-3-(2-phenyl-1H-indol-3-yl)-1-(3,4,5-trimethoxyphenyl)prop-2-en-1-one (8c): yield 88%, yellow solid, m. p. 253.8°C–255.9°C;^1^H NMR (400 MHz, DMSO-d_6_) δ 12.94 (s, 1H), 8.40 (d, J = 7.5 Hz, 1H), 8.18 (s, 1H), 7.62–7.54 (m, 6H), 7.39–7.32 (m, 2H), 7.09 (s, 2H), 3.80 (s, 6H), 3.74 (s, 3H);

IR (KBr): 3,228, 2,206, 1,561, 1,453, 1,223, 1,124, 751 cm^−1^; HR-MS (ESI-TOF) Calcd for [M + H]^+^ C_27_H_22_N_2_O_4_: 439.1658, found: 439.1654.

methyl(E)-3-(2-cyano-3-oxo-3-(3,4,5-trimethoxyphenyl)prop-1-en-1-yl)-1H-indole-4-carboxylate (8d): yield 90%, yellow solid, m. p. 176.7°C–178.2°C;^1^H NMR (400 MHz, Chloroform-d) δ 9.81 (s, 1H), 9.30 (s, 1H), 8.97 (s, 1H), 7.89 (d, J = 7.5 Hz, 1H), 7.71 (d, J = 8.1 Hz, 1H), 7.36 (t, J = 7.8 Hz, 1H), 7.17 (s, 2H), 3.96 (s, 6H), 3.95 (s, 3H), 3.86 (s, 3H);^13^C NMR (101 MHz, Chloroform-d) δ 189.81, 167.95, 152.94, 152.40, 141.77, 137.35, 132.75, 131.93, 126.35, 125.03, 124.28, 123.31, 116.96, 111.82, 106.84, 103.29, 60.99, 56.28, 52.37;

IR (KBr): 3,283, 2,202, 1720, 1,328, 1,216, 1,126, 735 cm^−1^;HR-MS (ESI-TOF) Calcd for [M + H]^+^ C_23_H_20_N_2_O_6_: 421.1400, found: 421.1394.

methyl(E)-3-(2-cyano-3-oxo-3-(3,4,5-trimethoxyphenyl)prop-1-en-1-yl)-1H-indole-5-carboxylate (8e): yield 92%, yellow solid, m. p. >260°C;^1^H NMR (400 MHz, DMSO-d_6_) δ 8.78 (s, 1H), 8.60 (s, 1H), 8.53 (s, 1H), 7.90 (d, J = 8.6 Hz, 1H), 7.69 (d, J = 8.7 Hz, 1H), 7.18 (s, 2H), 3.88 (s, 3H), 3.87 (s, 6H), 3.80 (s, 3H);^13^C NMR (101 MHz, DMSO-d_6_) δ 188.26, 166.69, 152.64, 147.19, 141.21, 135.02, 131.98, 127.10, 124.28, 123.32, 120.76, 113.46, 111.37, 109.31, 106.76, 105.12, 60.19, 56.11, 51.98; IR (KBr): 3,287, 2,203, 1724, 1,323, 1,226, 1,120, 744 cm^−1^;HR-MS (ESI-TOF) Calcd for [M + H]^+^ C_23_H_20_N_2_O_6_: 421.1400, found: 421.1405.

methyl(E)-3-(2-cyano-3-oxo-3-(3,4,5-trimethoxyphenyl)prop-1-en-1-yl)-1H-indole-6-carboxylate (8f): yield 94%, yellow solid, m. p. 239.7 –241.0°C;^1^H NMR (400 MHz, Chloroform-d) δ 9.50 (s, 1H), 8.91 (s, 1H), 8.67 (s, 1H), 8.27 (s, 1H), 8.03 (dd, J = 8.4, 1.3 Hz, 1H), 7.88 (d, J = 8.4 Hz, 1H), 7.24 (s, 2H), 3.98 (s, 3H), 3.97 (s, 3H), 3.95 (s, 6H);^13^C NMR (101 MHz, Chloroform-d) δ 186.33, 167.23, 153.04, 147.07, 135.16, 133.09, 131.66, 131.17, 126.37, 124.31, 123.74, 118.24, 114.46, 111.99, 106.80, 103.29, 61.07, 56.38, 52.37; IR (KBr): 3,274, 2000, 1723, 1,291, 1,230, 1,131, 750 cm^−1^;HR-MS (ESI-TOF) Calcd for [M + H]^+^ C_23_H_20_N_2_O_6_: 421.1400, found: 421.1405.

(E)-3-(6-bromo-1H-indol-3-yl)-2-(3,4,5-trimethoxybenzoyl)acrylonitrile (8g): yield 93%, yellow solid, m. p. 266.5°C–268.2°C;^1^H NMR (400 MHz, DMSO-d_6_) δ 12.49 (s, 1H), 8.70 (s, 1H), 8.51 (s, 1H), 7.90 (d, J = 8.5 Hz, 1H), 7.80 (d, J = 1.6 Hz, 1H), 7.39 (dd, J = 8.5, 1.7 Hz, 1H), 7.15 (s, 2H), 3.84 (s, 6H), 3.79 (s, 3H); IR (KBr): 3,297, 2,199, 1,556, 1,331, 1227, 1125 cm^−1^; HR-MS (ESI-TOF) Calcd for [M + H]^+^ C_21_H_17_BrN_2_O_4_: 442.0450, found: 442.0454.

(E)-3-(benzofuran-2-yl)-2-(3,4,5-trimethoxybenzoyl)acrylonitrile (8h): yield 86%, yellow solid, m. p. 168.3°C–169.5°C;^1^H NMR (400 MHz, Chloroform-d) δ 8.10 (s, 1H), 7.72 (d, J = 9.0 Hz, 2H), 7.62 (d, J = 8.4 Hz, 1H), 7.52 (t, J = 7.8 Hz, 1H), 7.35 (t, J = 7.5 Hz, 1H), 7.26 (s, 2H), 3.97 (s, 3H), 3.95 (s, 6H);^13^C NMR (101 MHz, Chloroform-d) δ 186.24, 156.54, 153.06, 149.95, 142.98, 140.92, 130.55, 129.15, 127.74, 124.34, 122.97, 118.09, 117.13, 112.27, 107.84, 107.04, 61.06, 56.39; IR (KBr): 3,442, 2,215, 1,599, 1,581, 1,337, 1,129, 752 cm^−1^;HR-MS (ESI-TOF) Calcd for [M + H]^+^ C_21_H_17_NO_5_: 364.1185, found: 364.1189.

(E)-3-(3-methylbenzofuran-2-yl)-2-(3,4,5-trimethoxybenzoyl)acrylonitrile (8i): yield 88%, yellow solid, m. p. 136.7°C–138.5°C;^1^H NMR (400 MHz, Chloroform-d) δ 8.13 (s, 1H), 7.64 (d, J = 7.8 Hz, 1H), 7.61 (d, J = 8.4 Hz, 1H), 7.55–7.50 (m, 1H), 7.36–7.31 (m, 1H), 7.29 (s, 2H), 3.96 (s, 3H), 3.95 (s, 6H), 2.52 (s, 3H);^13^C NMR (101 MHz, Chloroform-d) δ 186.84, 156.02, 152.97, 146.69, 142.81, 137.35, 130.85, 129.76, 128.75, 123.80, 121.17, 117.63, 112.40, 107.06, 105.62, 61.04, 56.37, 9.29; IR (KBr): 3,442, 2,212, 1,600, 1,581, 1,336, 1,128, 752 cm^−1^;HR-MS (ESI-TOF) Calcd for [M + H]^+^ C_22_H_19_NO_5_: 378.1341, found: 378.1346.

(E)-3-(benzofuran-3-yl)-2-(3,4,5-trimethoxybenzoyl)acrylonitrile (8j): yield 82%, yellow solid, m. p. 176.5°C–178.4°C;^1^H NMR (400 MHz, Chloroform-d) δ 8.97 (s, 1H), 8.43 (s, 1H), 7.79 (d, J = 8.2 Hz, 1H), 7.62 (d, J = 7.7 Hz, 1H), 7.48–7.39 (m, 2H), 7.26 (s, 2H), 3.97 (s, 3H), 3.95 (s, 6H);^13^C NMR (101 MHz, Chloroform-d) δ 186.06, 155.14, 153.11, 149.85, 144.80, 142.99, 130.71, 126.27, 125.62, 124.46, 119.24, 118.35, 115.92, 112.25, 108.83, 106.99, 61.07, 56.40; IR (KBr): 3,445, 2,204, 1,580, 1,335, 1,128, 749 cm^−1^;HR-MS (ESI-TOF) Calcd for [M + H]^+^ C_21_H_17_NO_5_: 364.1185, found: 364.1189.

(E)-3-[6-nitrobenzo(d)(1,3)dioxol-5-yl]-2-(3,4,5-trimethoxybenzoyl)acrylonitrile (8k): yield 91%, yellow solid, m. p. 160.1°C–163.8°C;^1^H NMR (400 MHz, Chloroform-d) δ 8.33 (s, 1H), 7.75 (s, 1H), 7.26 (s, 2H), 7.17 (d, J = 7.6 Hz, 1H), 6.26 (s, 2H), 3.98 (s, 6H), 3.96 (s, 3H);^13^C NMR (101 MHz, Chloroform-d) δ 187.21, 153.30, 152.83, 152.53, 150.43, 143.27, 142.36, 129.71, 129.04, 128.23, 125.29, 124.98, 115.06, 114.89, 109.16, 107.14, 106.14, 104.10, 61.05, 56.41; IR (KBr): 3,426, 2,228, 1,583, 1,508, 1,129, 1,033 cm^−1^;HR-MS (ESI-TOF) Calcd for [M + H]^+^ C_20_H_16_N_2_O_8_: 413.0985, found: 413.0988.

(E)-3-[dibenzo (b,d)furan-2-yl]-2-(3,4,5-trimethoxybenzoyl)acrylonitrile (8l): yield 87%, yellow solid, m. p. 181.3°C–182.6°C;^1^H NMR (400 MHz, Chloroform-d) δ 8.73 (d, J = 1.8 Hz, 1H), 8.29 (s, 1H), 8.16 (dd, J = 8.7, 1.8 Hz, 1H), 8.03 (d, J = 7.6 Hz, 1H), 7.68 (d, J = 8.7 Hz, 1H), 7.62 (d, J = 8.2 Hz, 1H), 7.54 (td, J = 7.3, 1.2 Hz, 1H), 7.42 (td, J = 7.8, 0.9 Hz, 1H), 7.24 (s, 2H), 3.97 (s, 3H), 3.95 (s, 6H);^13^C NMR (101 MHz, Chloroform-d) δ 187.43, 158.90, 156.92, 155.73, 153.07, 142.85, 131.04, 130.79, 128.48, 126.94, 125.58, 124.11, 123.71, 123.11, 121.22, 117.86, 112.77, 112.06, 108.05, 107.06, 61.06, 56.41; IR (KBr): 3,422, 2,205, 1,664, 1,573, 1,334, 1,135, 749 cm^−1^;HR-MS (ESI-TOF) Calcd for [M + H]^+^ C_25_H_19_NO_5_: 414.1341, found: 414.1346.

(E)-3-(benzofuran-5-yl)-2-(3,4,5-trimethoxybenzoyl)acrylonitrile (8m): yield 85%, yellow solid, m. p. 169.7°C–172.1°C; ^1^H NMR (400 MHz, Chloroform-d) δ 8.45 (s, 1H), 8.26 (s, 1H), 8.01 (dd, J = 8.7, 1.5 Hz, 1H), 7.75 (d, J = 2.1 Hz, 1H), 7.65 (d, J = 8.7 Hz, 1H), 7.23 (s, 2H), 6.91 (d, J = 1.4 Hz, 1H), 3.98 (s, 3H), 3.96 (s, 6H);^13^C NMR (101 MHz, Chloroform-d) δ 187.57, 157.45, 156.24, 153.05, 146.94, 142.79, 130.81, 128.55, 128.20, 127.07, 125.11, 117.81, 112.56, 108.04, 107.16, 107.03, 61.05, 56.38; IR (KBr): 3,421, 2,201, 1,660, 1,570, 1,334, 1,136, 736 cm^−1^; HR-MS (ESI-TOF) Calcd for [M + H]^+^ C_21_H_17_NO_5_: 364.1185, found: 364.1189.

(E)-3-(2,3-dihydrobenzofuran-5-yl)-2-(3,4,5-trimethoxybenzoyl)acrylonitrile (8n): yield 95%, yellow solid, m. p. 173.4°C–175.0°C;^1^H NMR (400 MHz, Chloroform-d) δ 8.15 (s, 1H), 8.07 (s, 1H), 7.77 (dd, J = 8.5, 1.7 Hz, 1H), 7.18 (s, 2H), 6.90 (d, J = 8.4 Hz, 1H), 4.72 (t, J = 8.8 Hz, 2H), 3.95 (s, 3H), 3.93 (s, 6H), 3.31 (t, J = 8.7 Hz, 2H);^13^C NMR (101 MHz, Chloroform-d) δ 187.94, 165.27, 155.76, 152.98, 142.49, 135.15, 131.22, 129.08, 127.59, 125.01, 118.38, 110.35, 106.89, 105.18, 72.68, 61.02, 56.35, 28.96; IR (KBr): 3,443, 2,202, 1,659, 1,493, 1,332, 1,131, 752 cm^−1^;HR-MS (ESI-TOF) Calcd for [M + H]^+^ C_21_H_19_NO_5_: 366.1341, found: 366.1346.

(E)-3-[benzo(b)thiophen-2-yl]-2-(3,4,5-trimethoxybenzoyl)acrylonitrile (8o): yield 92%, yellow solid, m. p. 187.5°C–189.3°C;^1^H NMR (400 MHz, Chloroform-d) δ 8.42 (s, 1H), 8.07 (s, 1H), 7.92 (dd, J = 8.0, 4.0 Hz, 2H), 7.52 (td, J = 7.7, 1.1 Hz, 1H), 7.45 (td, J = 8.1, 0.8 Hz, 1H), 7.25 (s, 2H), 3.97 (s, 3H), 3.95 (s, 6H);^13^C NMR (101 MHz, Chloroform-d) δ 186.40, 153.08, 148.57, 143.21, 142.95, 138.21, 136.24, 135.88, 130.73, 128.36, 125.69, 125.61, 122.80, 117.52, 107.88, 107.01, 61.07, 56.41; IR (KBr): 3,431, 2,202, 1,655, 1,569, 1,333, 1,131, 742 cm^−1^;HR-MS (ESI-TOF) Calcd for [M + H]^+^ C_21_H_17_NO_4_S: 380.0957, found: 380.0952.

(E)-3-[benzo(b)thiophen-3-yl]-2-(3,4,5-trimethoxybenzoyl)acrylonitrile (8p): yield 93%, yellow solid, m. p. 201.8°C–203.1°C;^1^H NMR (400 MHz, Chloroform-d) δ 9.10 (s, 1H), 8.54 (s, 1H), 7.95 (dd, J = 7.4, 4.8 Hz, 2H), 7.57–7.47 (m, 2H), 7.26 (s, 2H), 3.97 (s, 3H), 3.95 (s, 6H); 13C NMR (101 MHz, Chloroform-d) δ 186.88, 153.11, 144.64, 142.92, 139.29, 138.17, 134.72, 130.84, 128.35, 125.94, 125.71, 123.13, 120.91, 118.43, 108.79, 107.02, 61.08, 56.40; IR (KBr): 3,426, 2,199, 1,664, 1,574, 1,332, 1,126, 756 cm-1; HR-MS (ESI-TOF) Calcd for [M + H]^+^ C_21_H_17_NO_4_S:380.0957, found: 380.0952.

(E)-3-[dibenzo (b,d)thiophen-2-yl]-2-(3,4,5-trimethoxybenzoyl)acrylonitrile (8q): yield 92%, yellow solid, m. p. 199.7°C–201.1°C;^1^H NMR (400 MHz, Chloroform-d) δ 8.86 (s, 1H), 8.29 (s, 1H), 8.25–8.20 (m, 1H), 8.12 (dd, J = 8.5, 1.6 Hz, 1H), 7.96 (d, J = 8.4 Hz, 1H), 7.90–7.86 (m, 1H), 7.56–7.50 (m, 2H), 7.25 (s, 2H), 3.97 (s, 3H), 3.95 (s, 6H);^13^C NMR (101 MHz, Chloroform-d) δ 187.34, 155.63, 153.08, 145.17, 142.90, 139.68, 136.32, 134.66, 130.76, 128.69, 128.22, 127.86, 125.23, 124.46, 123.58, 123.00, 122.05, 117.86, 108.52, 107.10; 61.06, 56.41; IR (KBr): 3,440, 2,208, 1,567, 1,335, 1,143, 756 cm^−1^;HR-MS (ESI-TOF) Calcd for [M + H]^+^ C_25_H_19_NO_4_S: 430.1113, found: 430.1116.

(E)-3-[1-methyl-1H-benzo (d)imidazol-2-yl]-2-(3,4,5-trimethoxybenzoyl)acrylonitrile (8r): yield 80%, yellow solid, m. p. 201.8°C–203.1°C;^1^H NMR (400 MHz, Chloroform-d) δ 8.18 (s, 1H), 7.95 (d, J = 8.1 Hz, 1H), 7.46–7.38 (m, 3H), 7.34 (s, 2H), 3.97 (s, 6H), 3.95 (s, 6H);^13^C NMR (101 MHz, Chloroform-d) δ 185.95, 153.03, 144.77, 143.80, 143.38, 137.30, 136.16, 130.00, 126.19, 124.32, 121.96, 116.76, 113.77, 110.13, 107.35, 61.07, 56.44, 30.26; IR (KBr): 3,428, 2,229, 1,666, 1,586, 1,336, 1,128, 754 cm^−1^;HR-MS (ESI-TOF) Calcd for [M + H]^+^ C_21_H_19_N_3_O_4_: 378.1454, found: 378.1458.

(E)-3-[benzo(d)(1,3)dioxol-4-yl]-2-(3,4,5-trimethoxybenzoyl)acrylonitrile (8s): yield 88%, yellow solid, m. p. 176.3°C–177.8°C;^1^H NMR (400 MHz, Chloroform-d) δ 8.24 (s, 1H), 7.92 (dd, J = 7.0, 2.2 Hz, 1H), 7.18 (s, 2H), 6.99–6.96 (m, 2H), 6.08 (s, 2H), 3.96 (s, 3H), 3.93 (s, 6H);^13^C NMR (101 MHz, Chloroform-d) δ 187.30, 153.06, 149.19, 148.00, 147.17, 142.92, 130.47, 122.42, 119.90, 117.09, 114.75, 112.67, 110.06, 107.13, 101.96, 61.05, 56.38; IR (KBr): 3,440, 2,210, 1,581, 1,457, 1,336, 1,127 cm^−1^;HR-MS (ESI-TOF) Calcd for [M + H]^+^ C_20_H_17_NO_6_: 368.1134, found: 368.1138.

(E)-3-[6-bromobenzo(d)(1,3)dioxol-5-yl]-2-(3,4,5-trimethoxybenzoyl)acrylonitrile (8t): yield 89%, yellow solid, m. p. 207.1°C–209.0°C;^1^H NMR (400 MHz, Chloroform-d) δ 8.37 (s, 1H), 7.91 (s, 1H), 7.18 (s, 3H), 6.13 (s, 2H), 3.96 (s, 3H), 3.94 (s, 6H); IR (KBr): 3,424, 2,200, 1,581, 1,486, 1,336, 1,130 cm^−1^;HR-MS (ESI-TOF) Calcd for [M + H]^+^ C_20_H_16_BrNO_6_: 447.0239, found: 447.0234.

## Data Availability

The original contributions presented in the study are included in the article/Supplementary Material. Further inquiries can be directed to the corresponding authors.
