# Supplementary figures and images for: Angiogenesis, Anti-Tumor, and Anti-Metastatic Activity of Novel α-Substituted Hetero-Aromatic Chalcone Hybrids as Inhibitors of Microtubule Polymerization
Source: Front Chem. 2021 Nov 19;9:766201. doi: 10.3389/fchem.2021.766201 (PMC8652888; doi:10.3389/fchem.2021.766201)

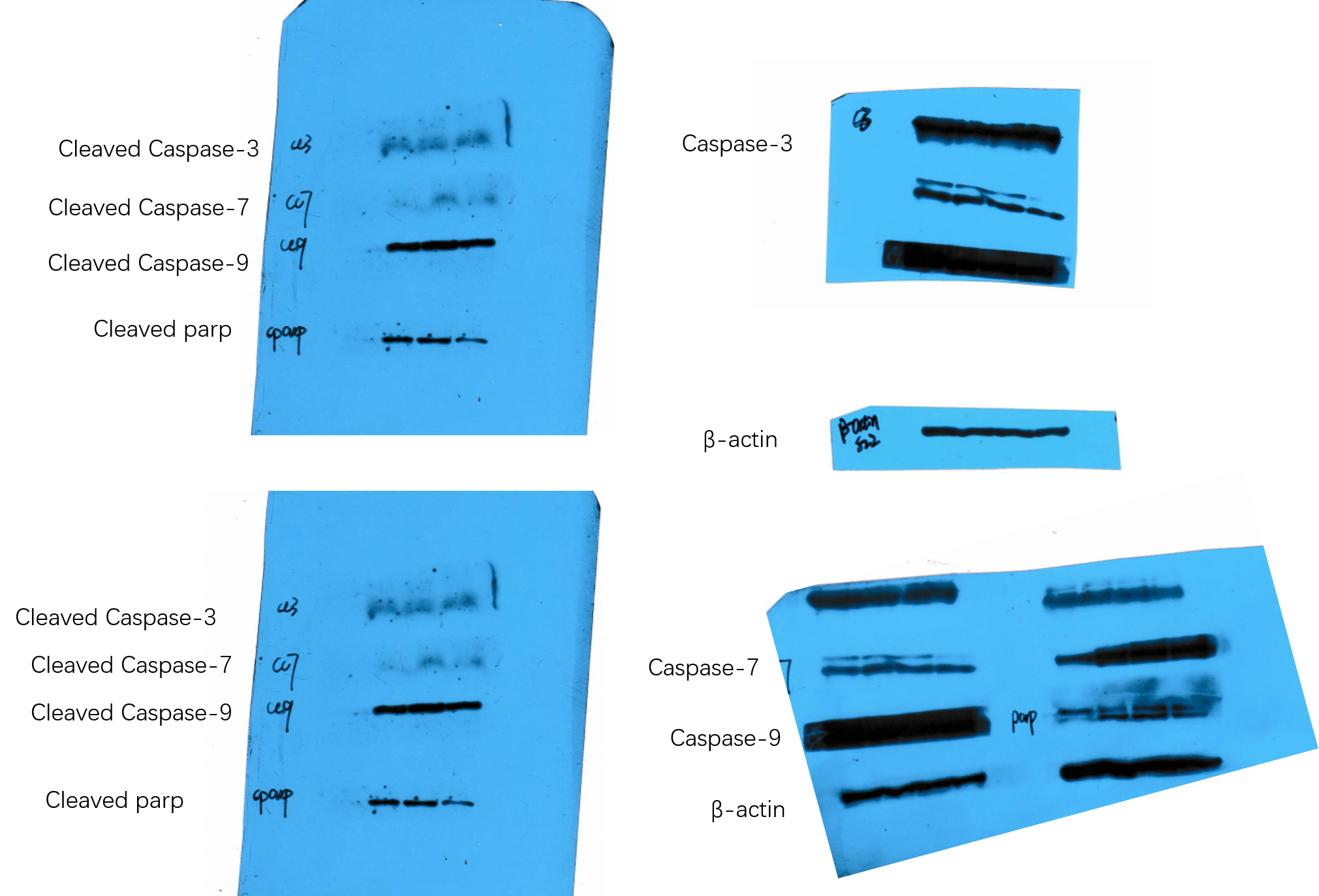

Supplement: Supplementary file 1 [file DataSheet3.ZIP › original data of apotosis/7m-apoptosis -WB.tif]

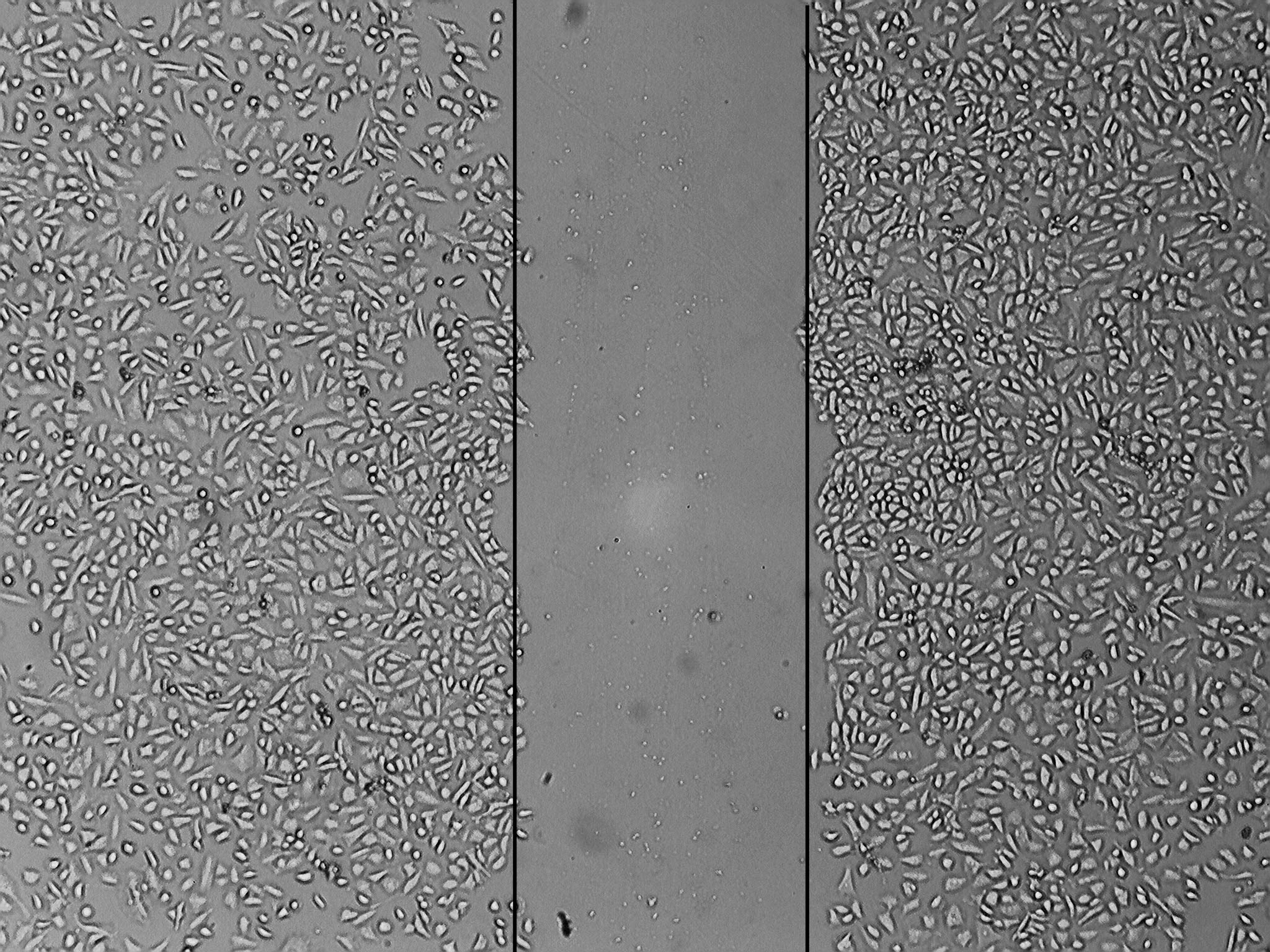

Supplement: Supplementary file 3 [file DataSheet8.ZIP › original data of wound-healing assay/HUVECs/7m-0h/7m-1a╠m.jpg]

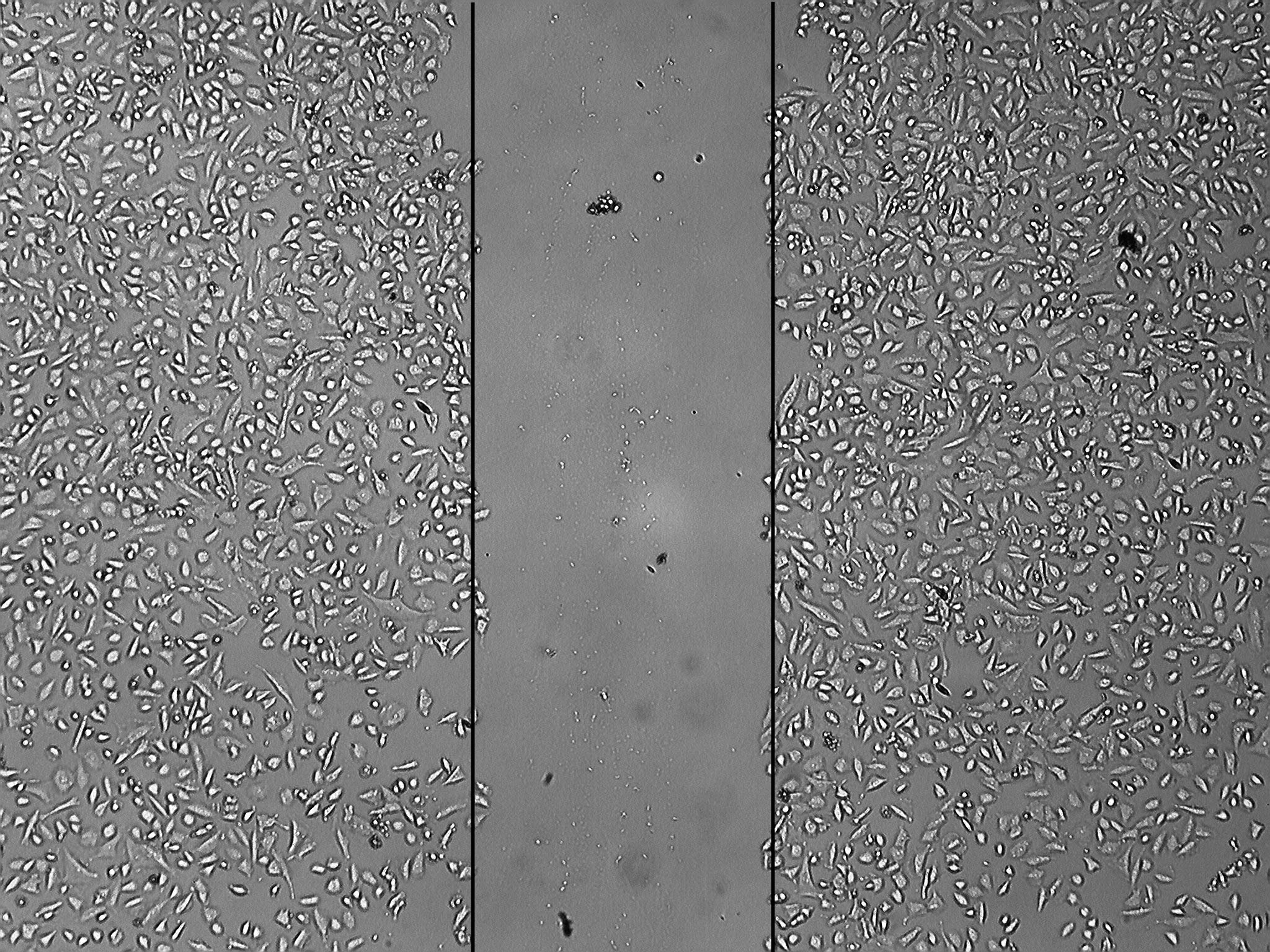

Supplement: Supplementary file 3 [file DataSheet8.ZIP › original data of wound-healing assay/HUVECs/7m-0h/7m-25a╠m.jpg]

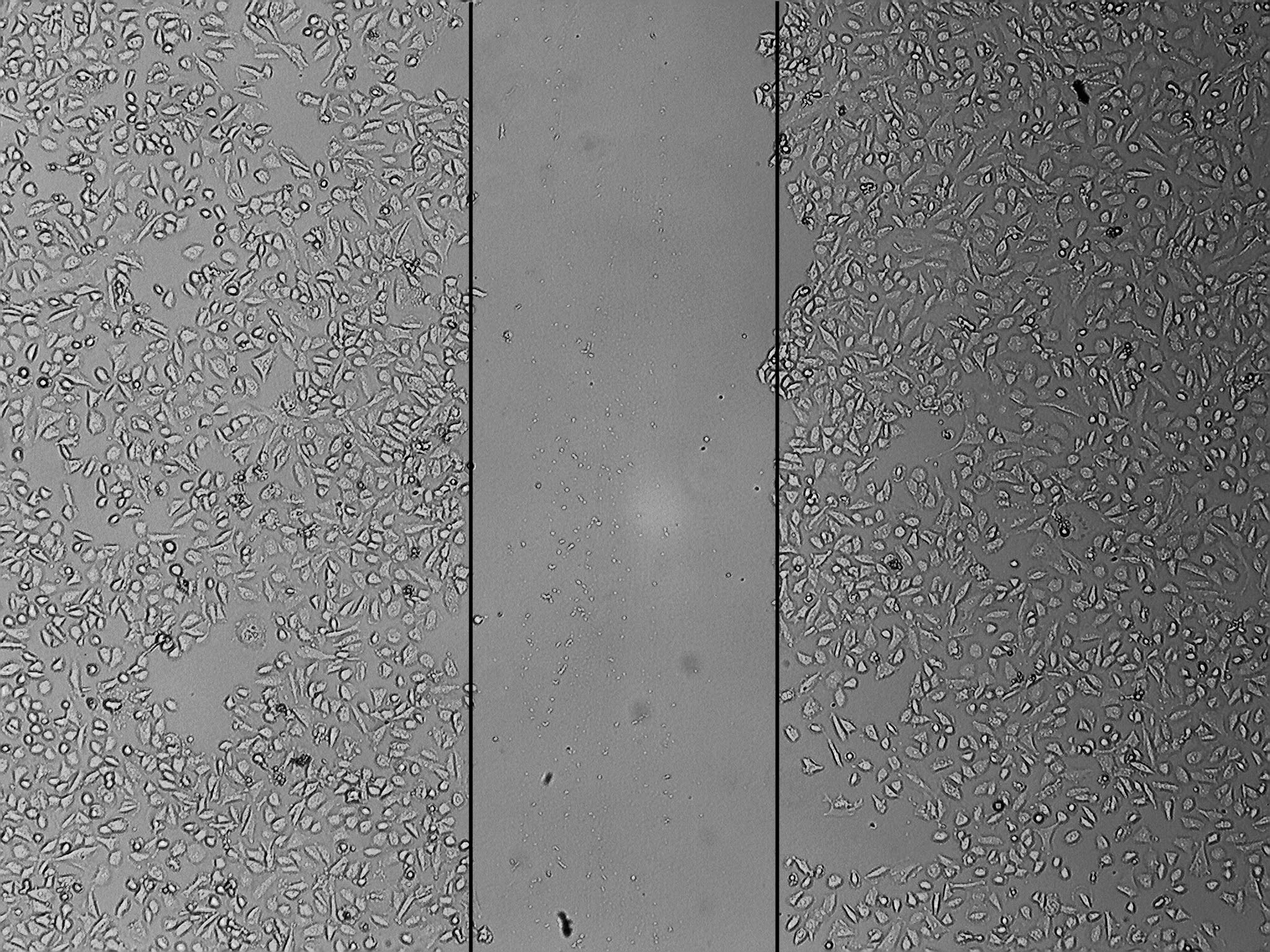

Supplement: Supplementary file 3 [file DataSheet8.ZIP › original data of wound-healing assay/HUVECs/7m-0h/7m-5a╠m.jpg]

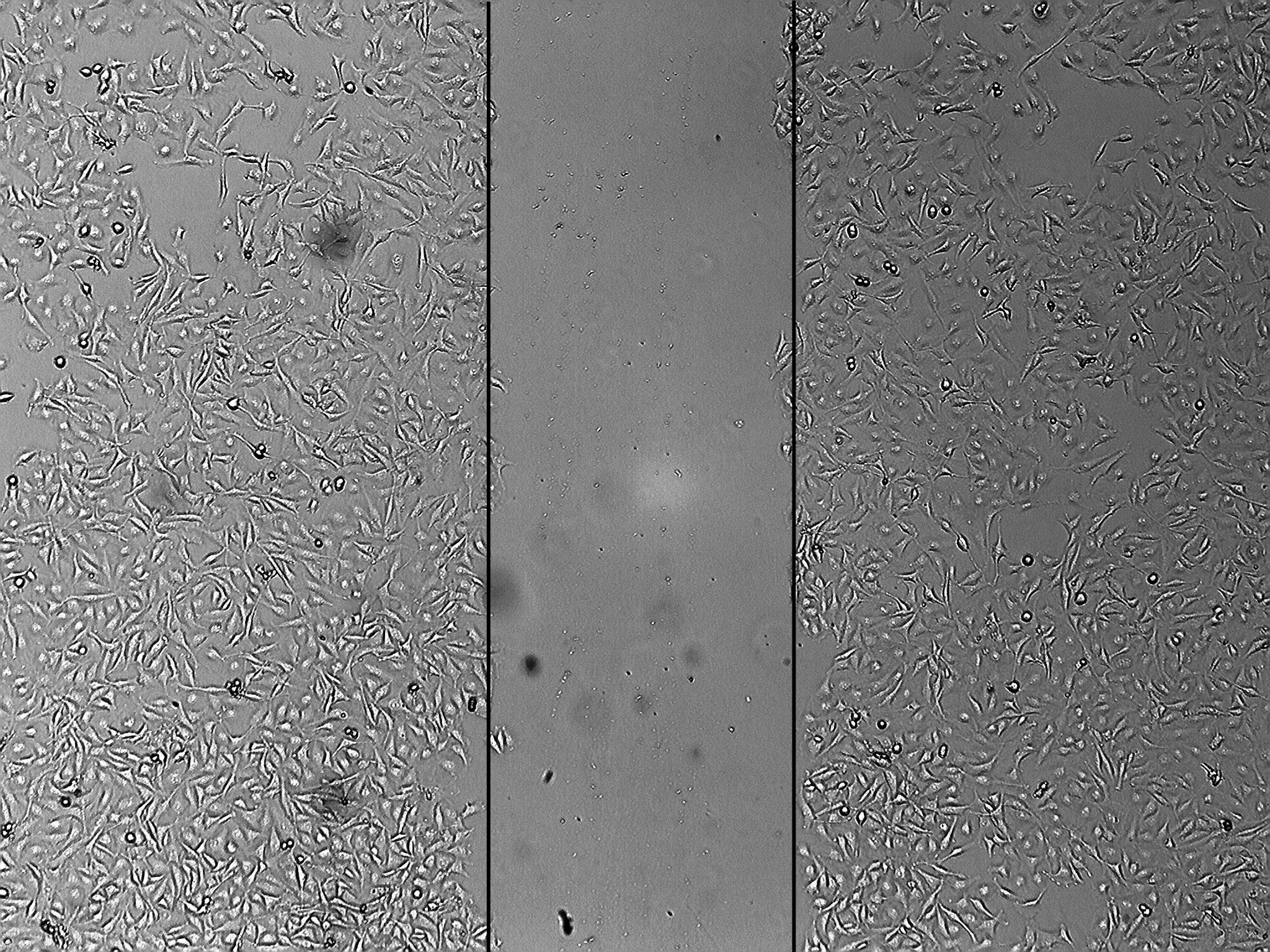

Supplement: Supplementary file 3 [file DataSheet8.ZIP › original data of wound-healing assay/HUVECs/7m-0h/NC.jpg]

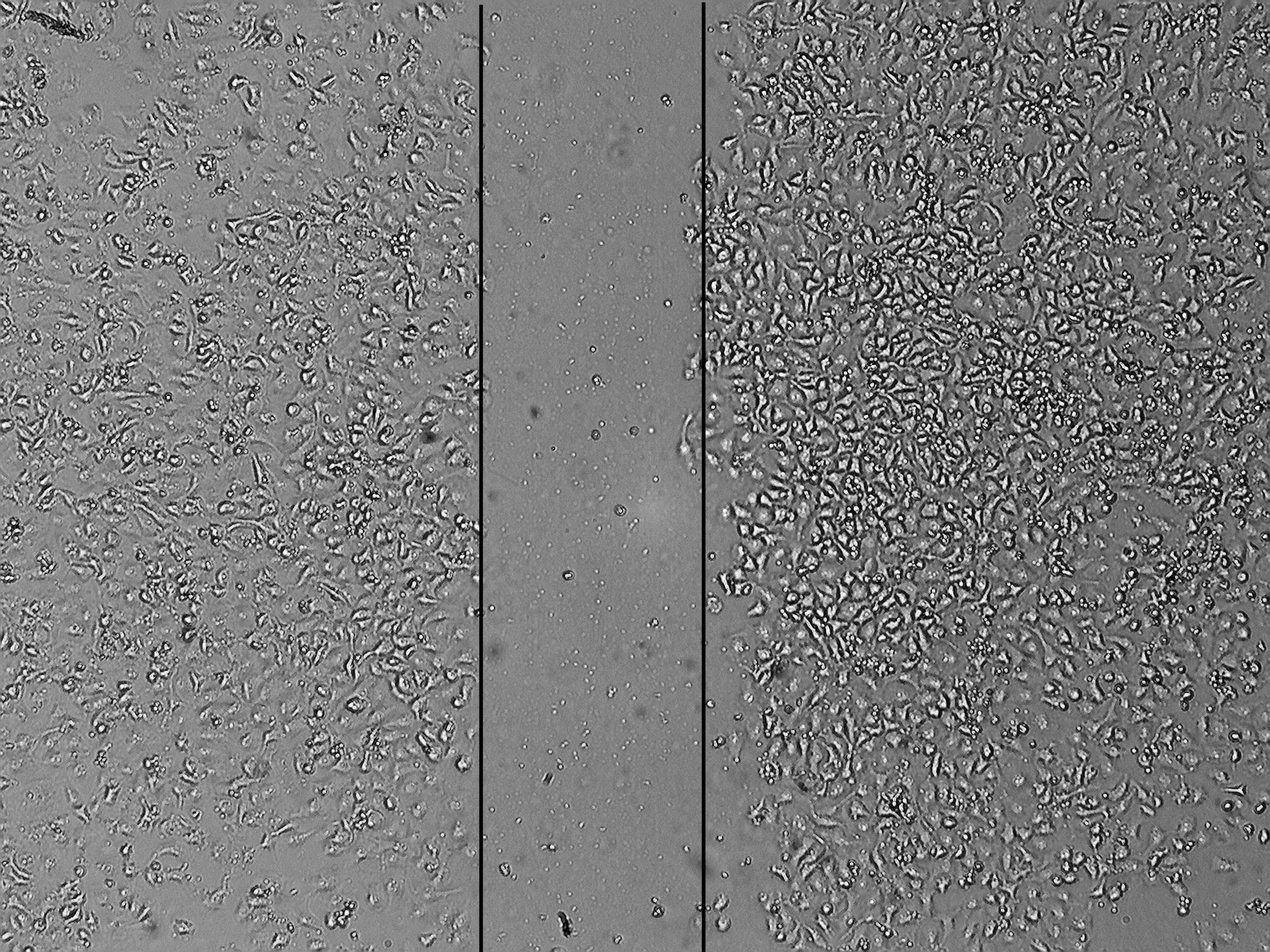

Supplement: Supplementary file 3 [file DataSheet8.ZIP › original data of wound-healing assay/HUVECs/7m-48h/7m-1a╠m.jpg]

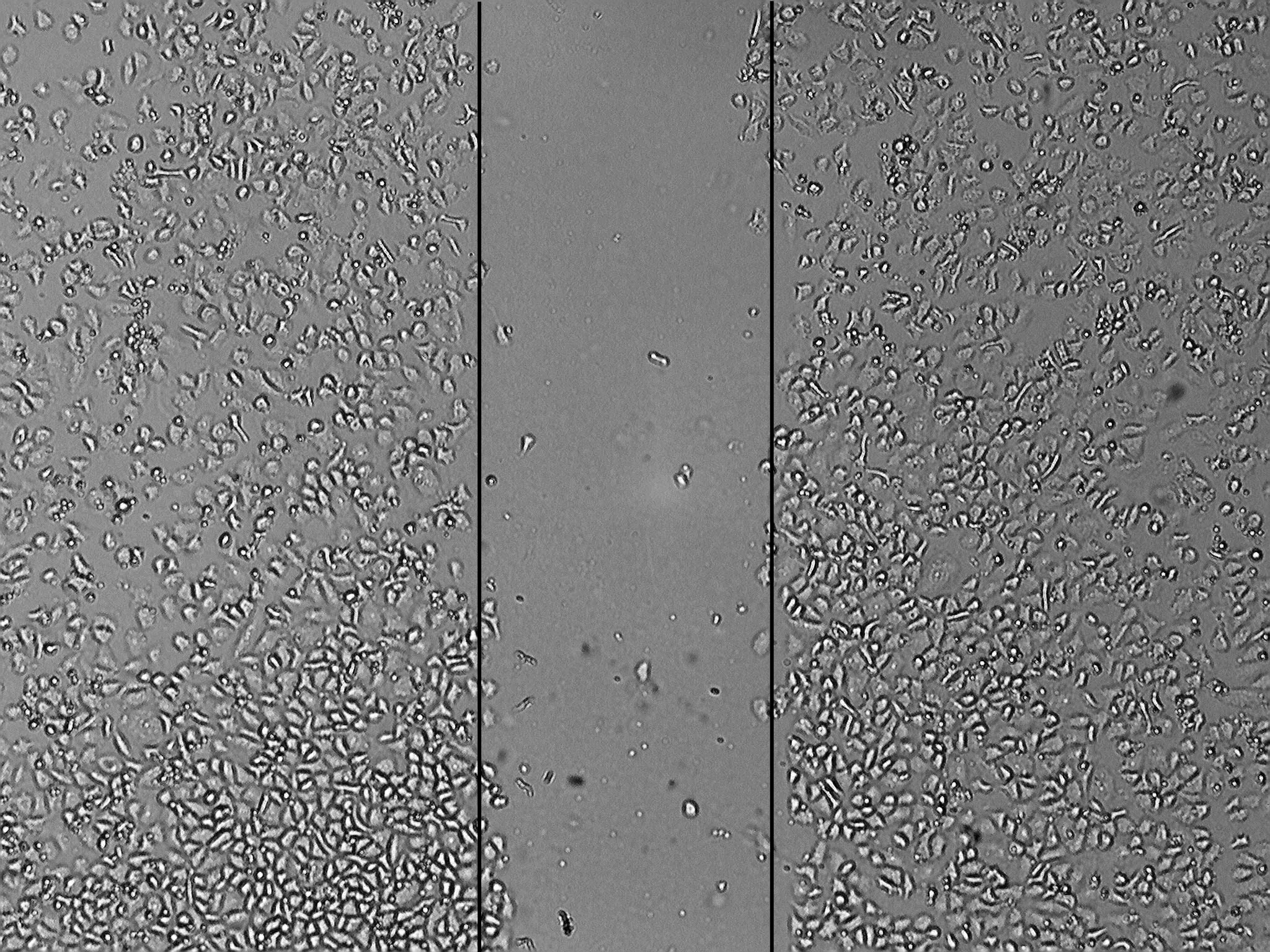

Supplement: Supplementary file 3 [file DataSheet8.ZIP › original data of wound-healing assay/HUVECs/7m-48h/7m-25a╠m.jpg]

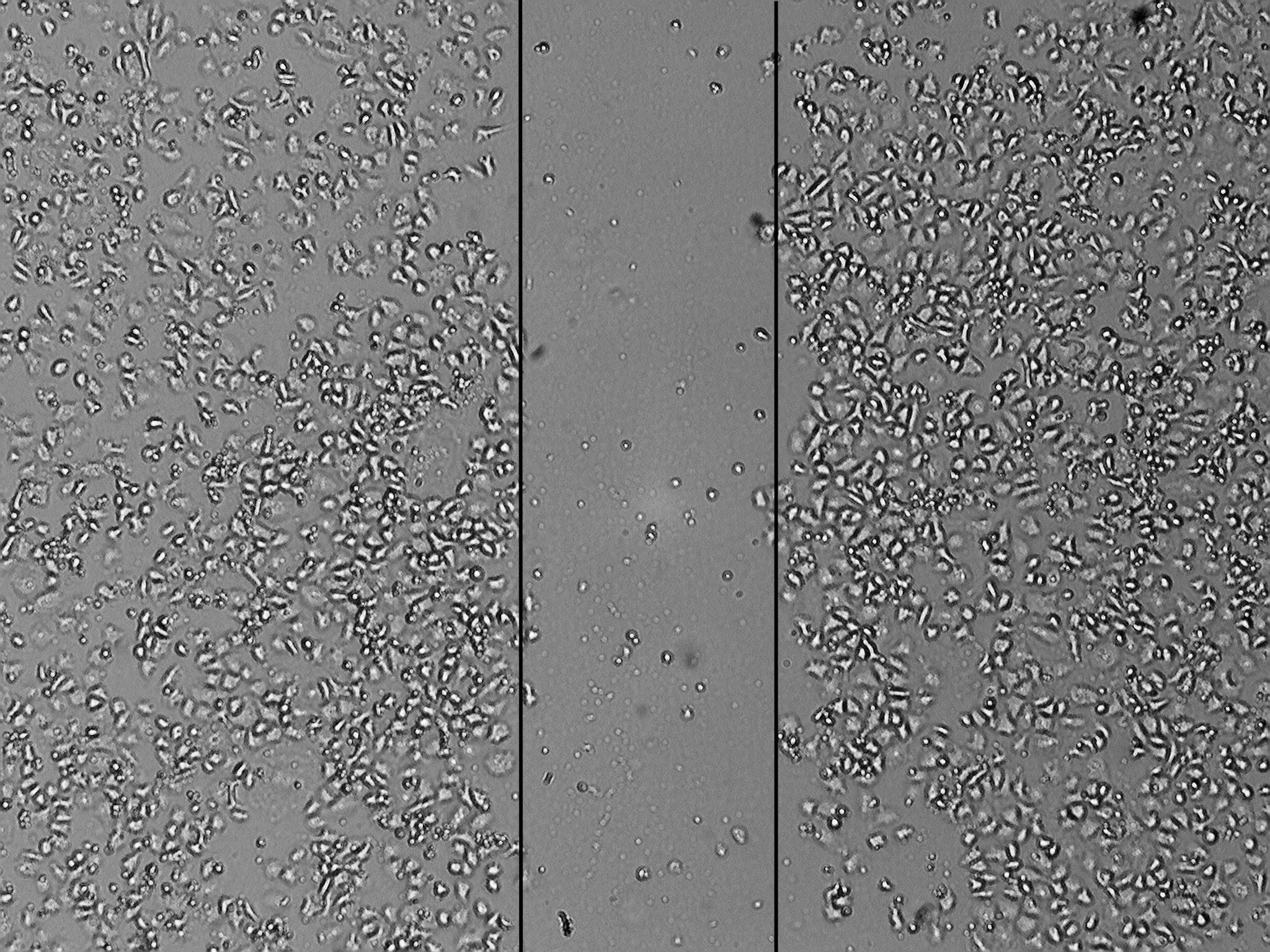

Supplement: Supplementary file 3 [file DataSheet8.ZIP › original data of wound-healing assay/HUVECs/7m-48h/7m-5a╠m.jpg]

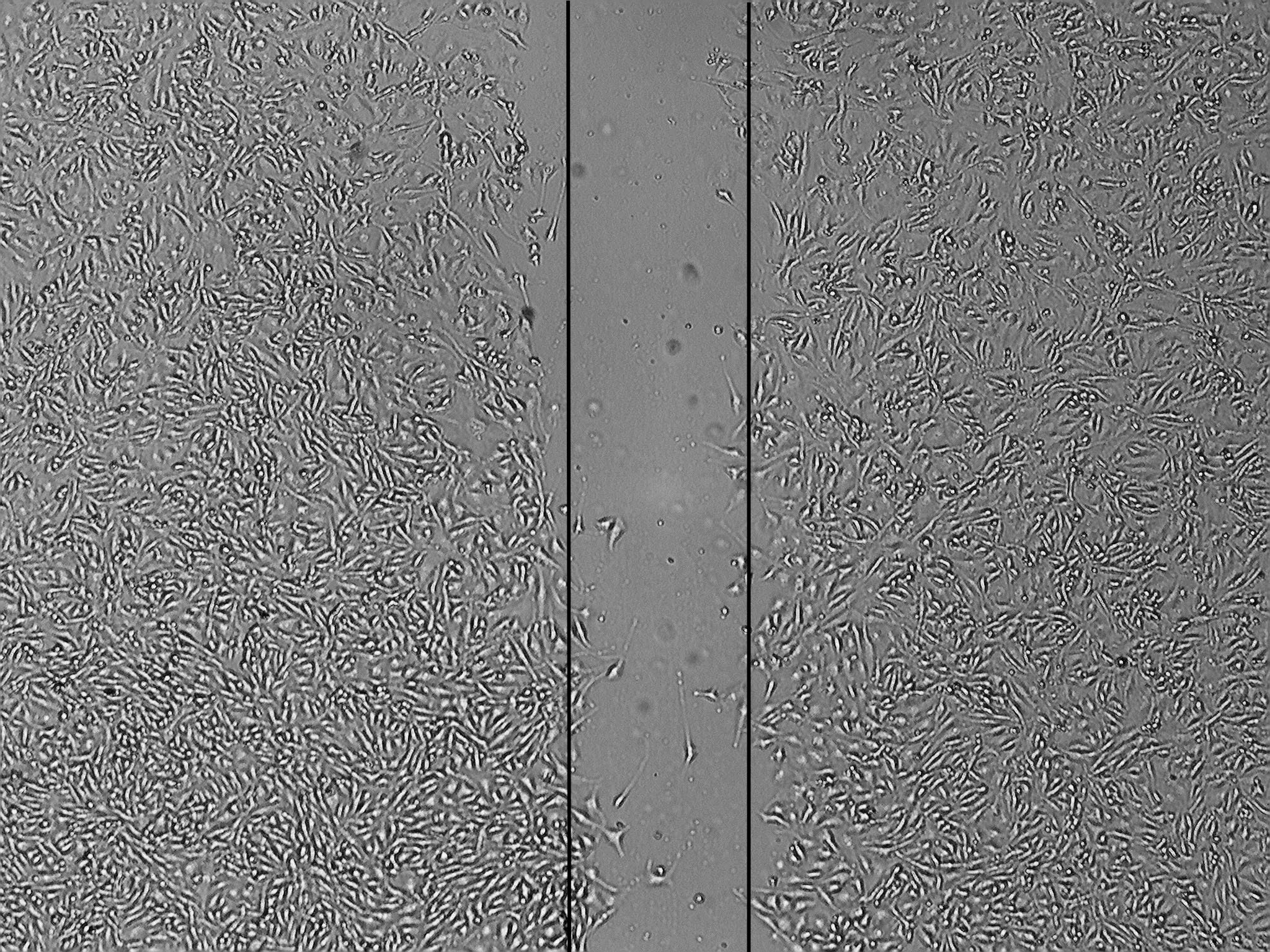

Supplement: Supplementary file 3 [file DataSheet8.ZIP › original data of wound-healing assay/HUVECs/7m-48h/NC.jpg]

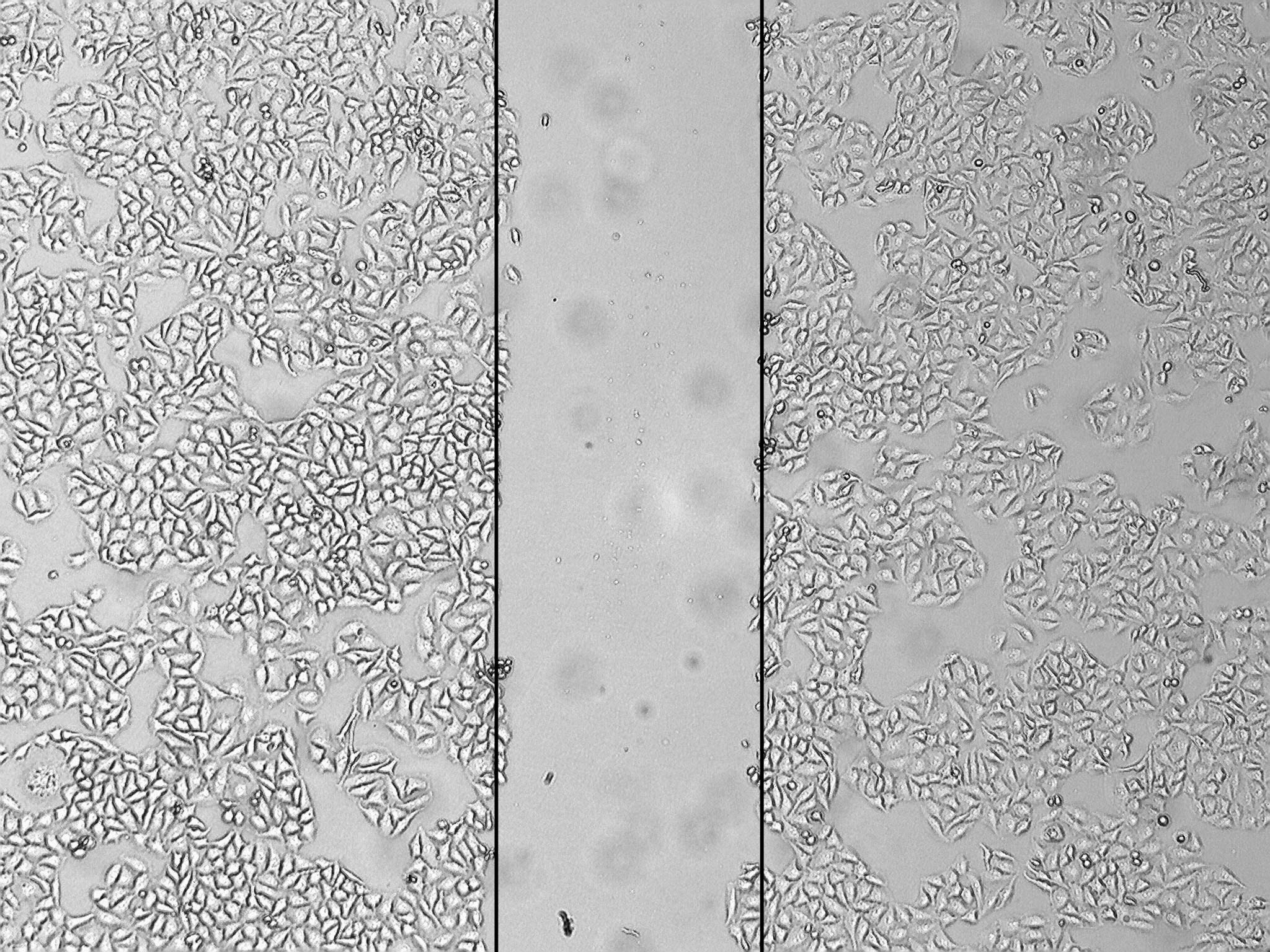

Supplement: Supplementary file 3 [file DataSheet8.ZIP › original data of wound-healing assay/MCF-7 cells/7m-0h/7m-100nm.jpg]

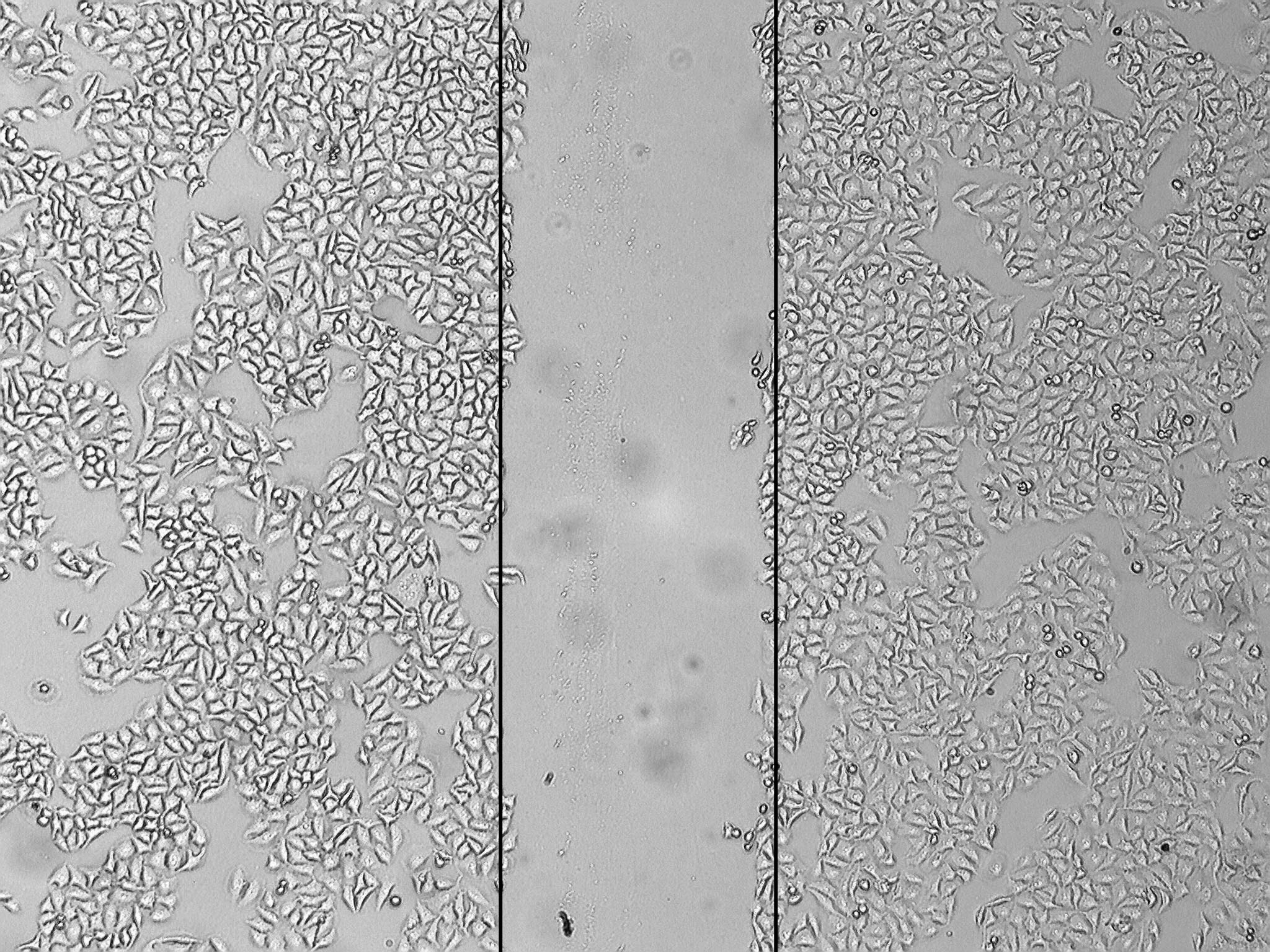

Supplement: Supplementary file 3 [file DataSheet8.ZIP › original data of wound-healing assay/MCF-7 cells/7m-0h/7m-200nm.jpg]

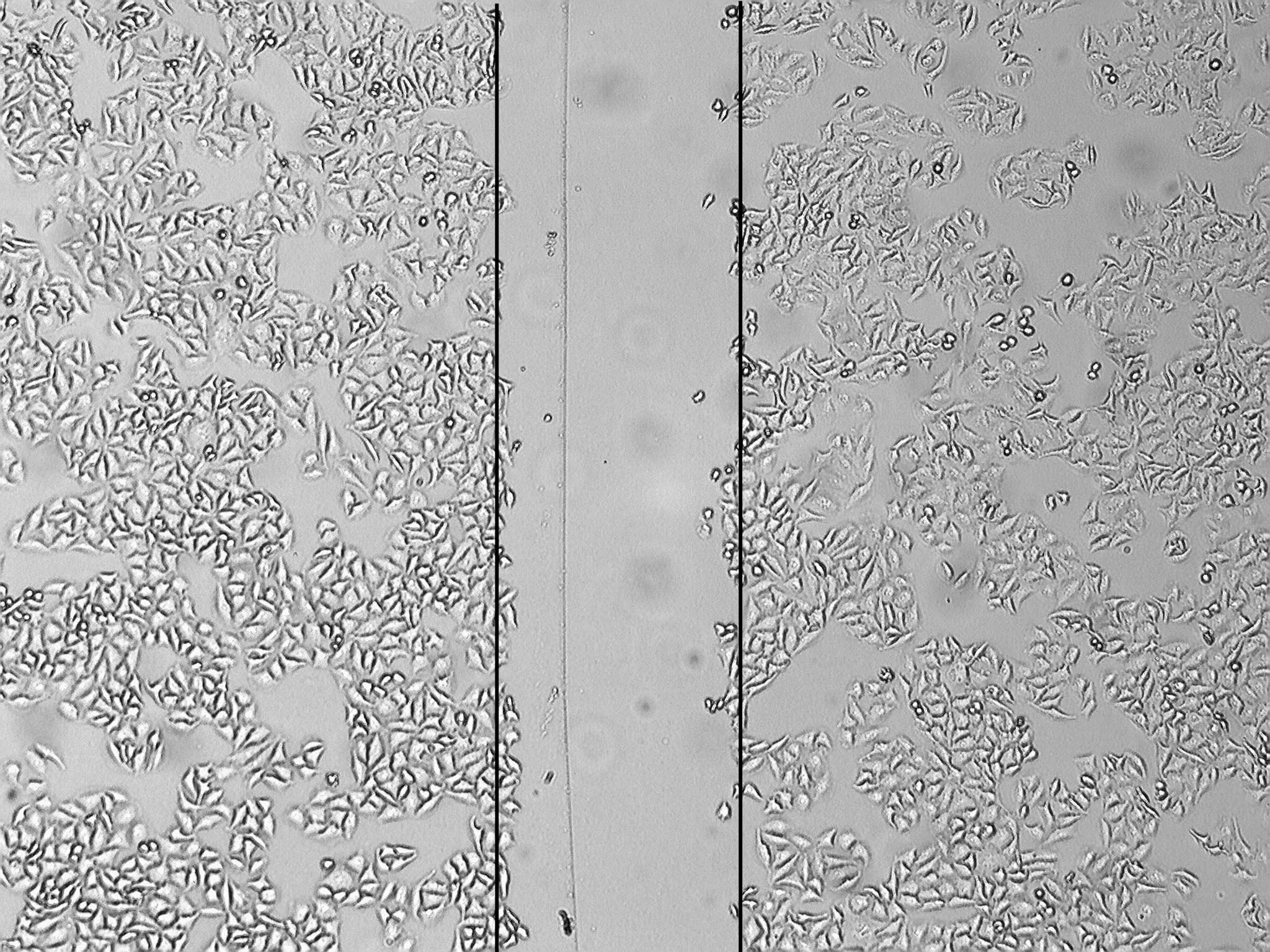

Supplement: Supplementary file 3 [file DataSheet8.ZIP › original data of wound-healing assay/MCF-7 cells/7m-0h/7m-400nm.jpg]

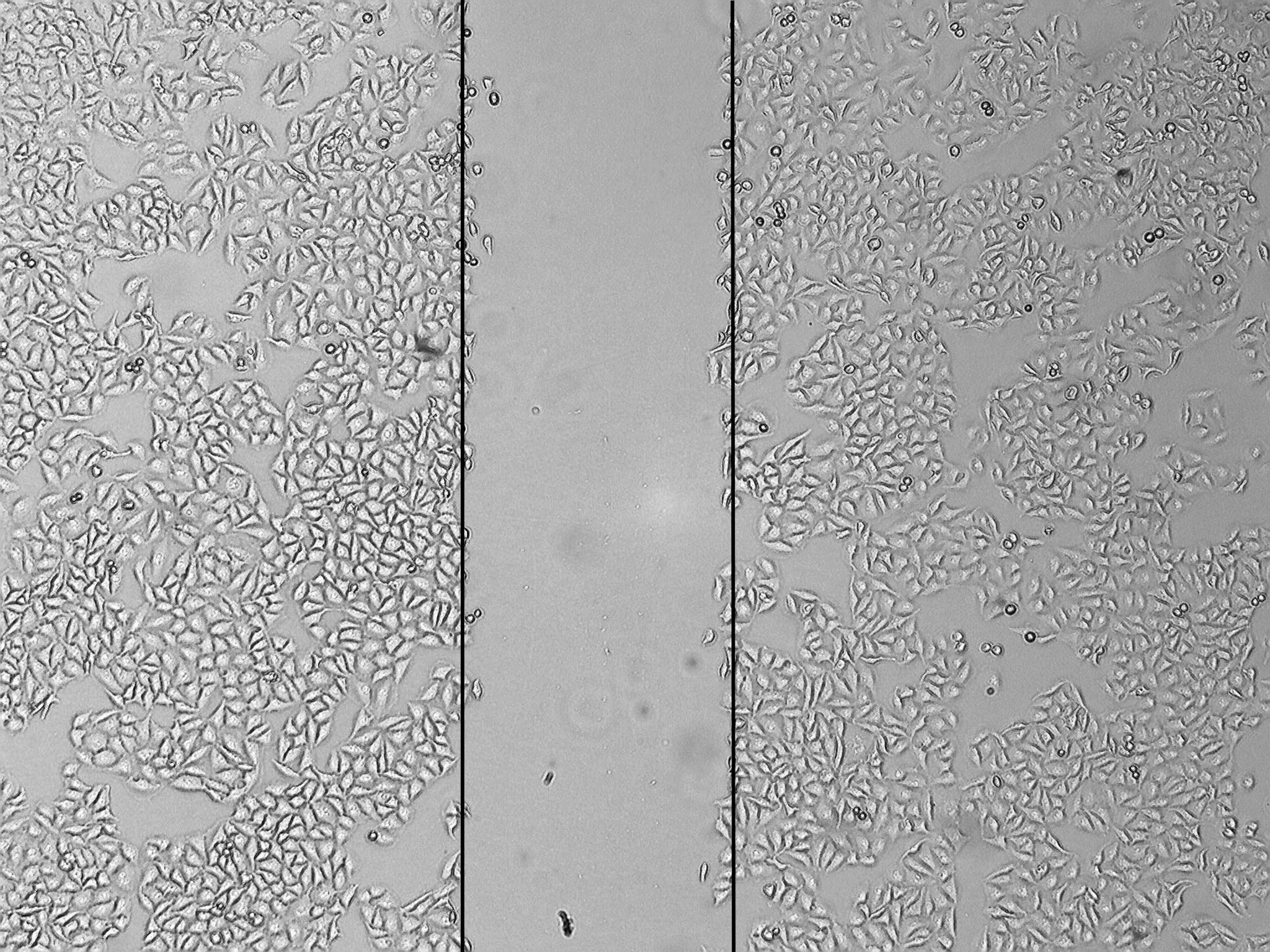

Supplement: Supplementary file 3 [file DataSheet8.ZIP › original data of wound-healing assay/MCF-7 cells/7m-0h/NC.jpg]

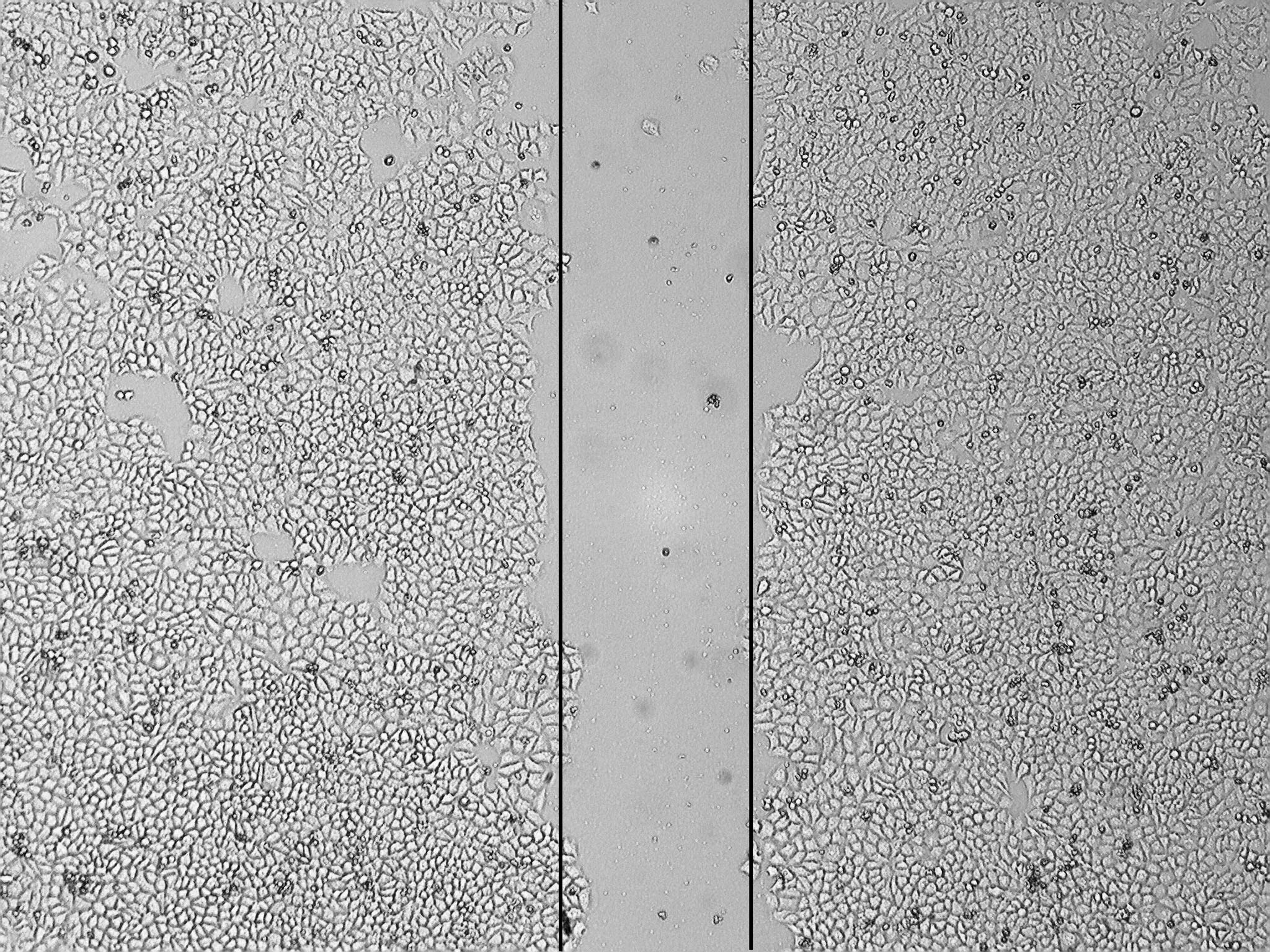

Supplement: Supplementary file 3 [file DataSheet8.ZIP › original data of wound-healing assay/MCF-7 cells/7m-48h/7m-100nm.jpg]

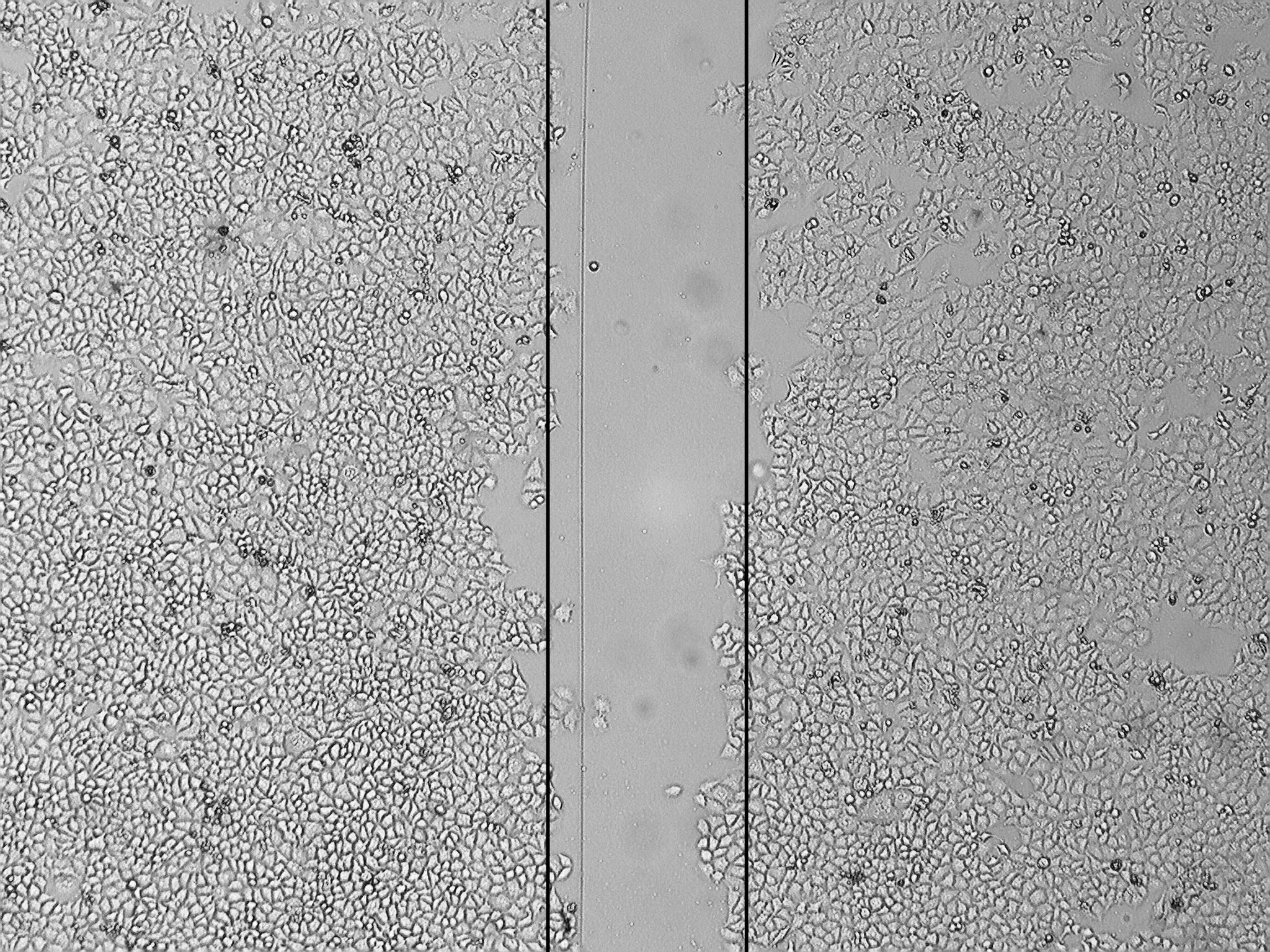

Supplement: Supplementary file 3 [file DataSheet8.ZIP › original data of wound-healing assay/MCF-7 cells/7m-48h/7m-200nm.jpg]

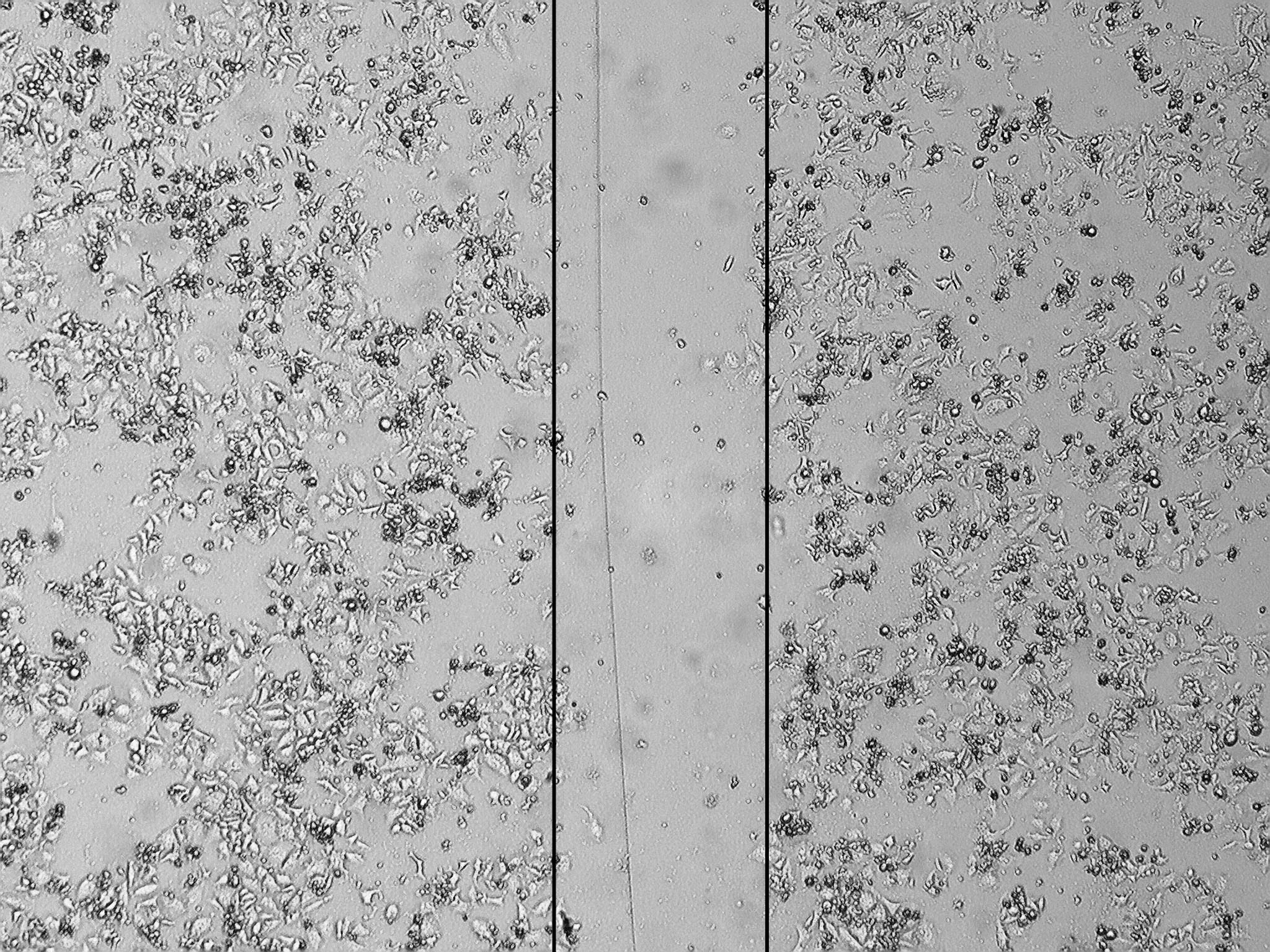

Supplement: Supplementary file 3 [file DataSheet8.ZIP › original data of wound-healing assay/MCF-7 cells/7m-48h/7m-400nm.jpg]

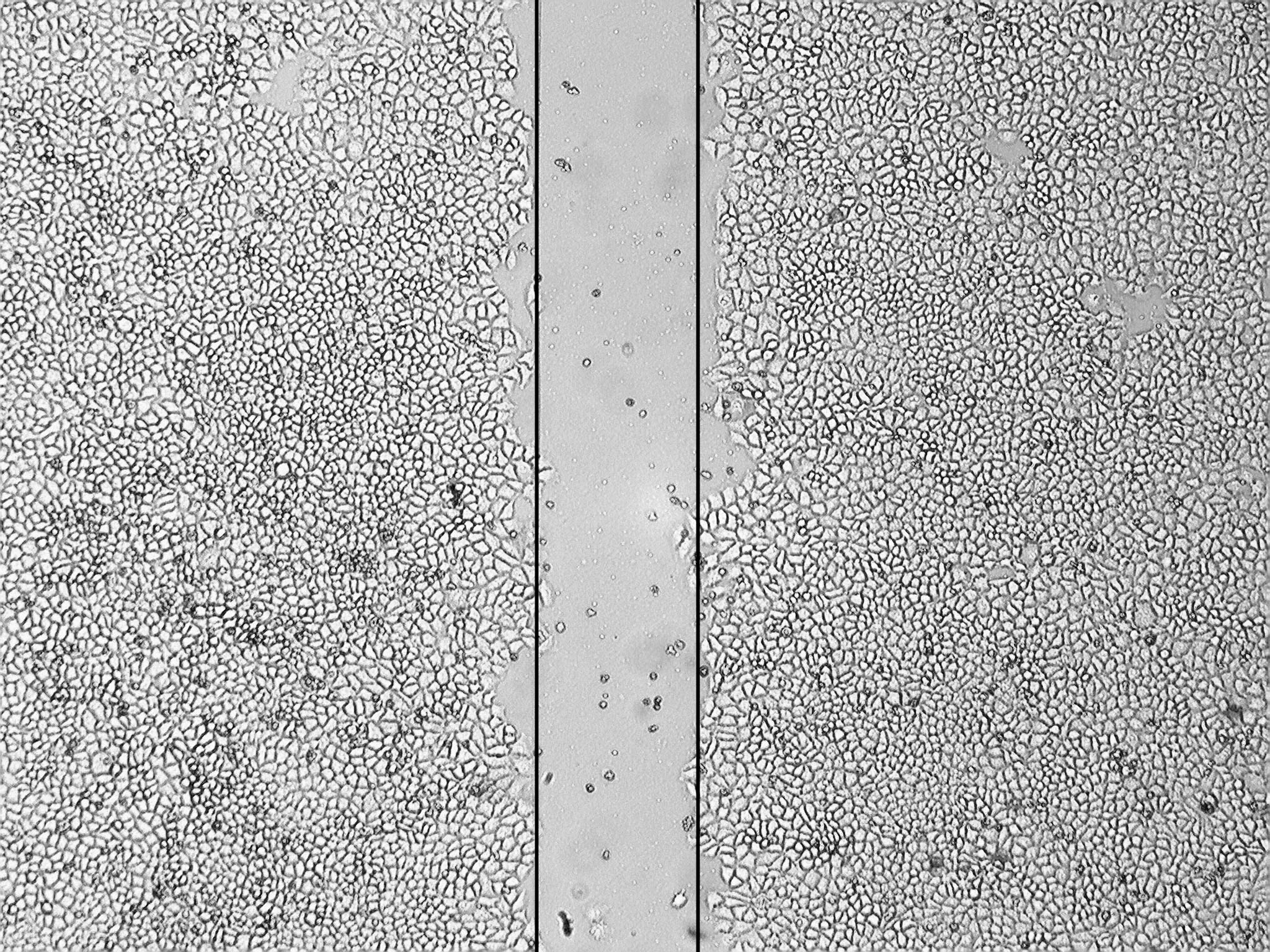

Supplement: Supplementary file 3 [file DataSheet8.ZIP › original data of wound-healing assay/MCF-7 cells/7m-48h/NC.jpg]

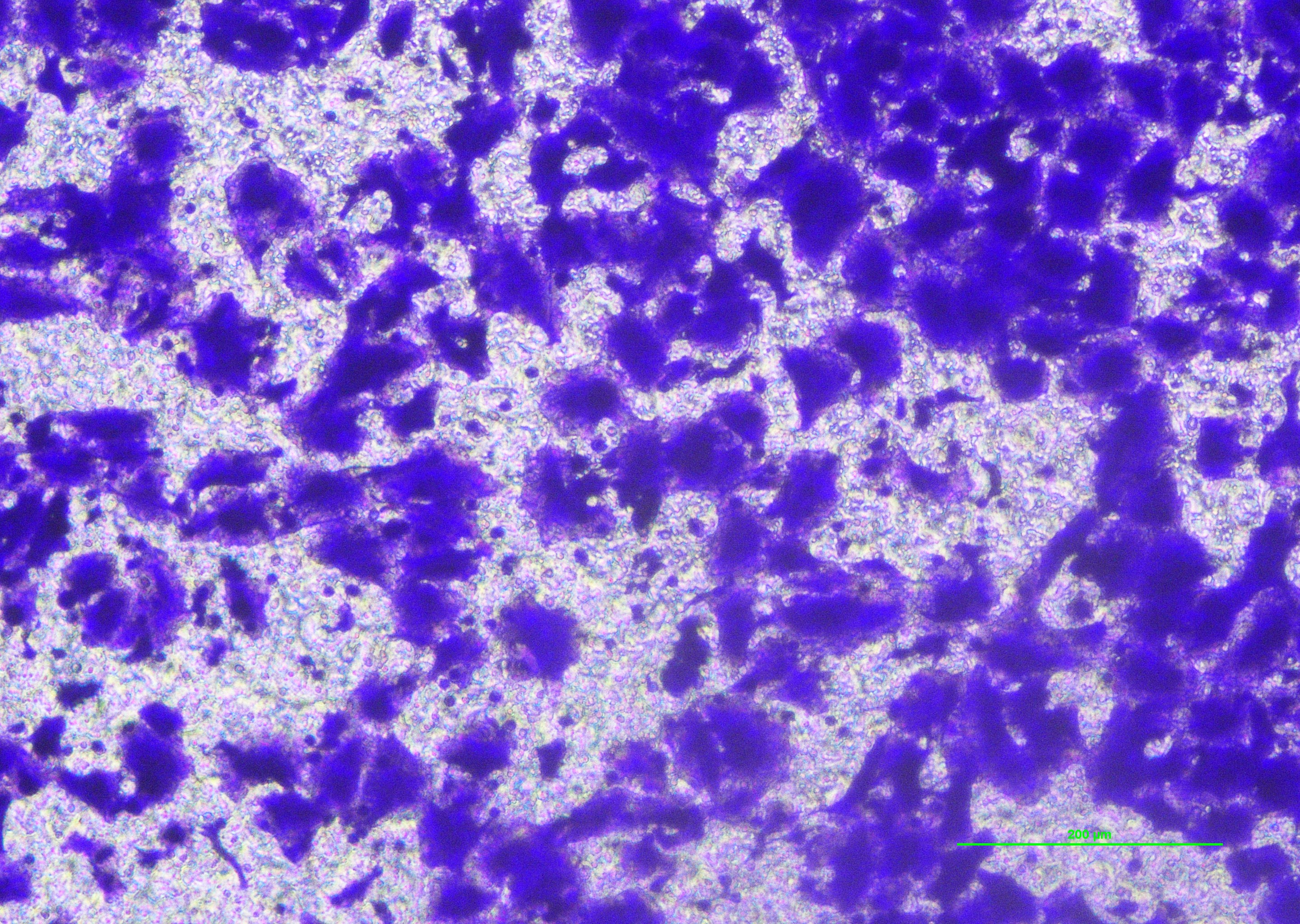

Supplement: Supplementary file 4 [file DataSheet9.ZIP › original data of transwell assay-MCF-7 cells/7m-100nm.jpg]

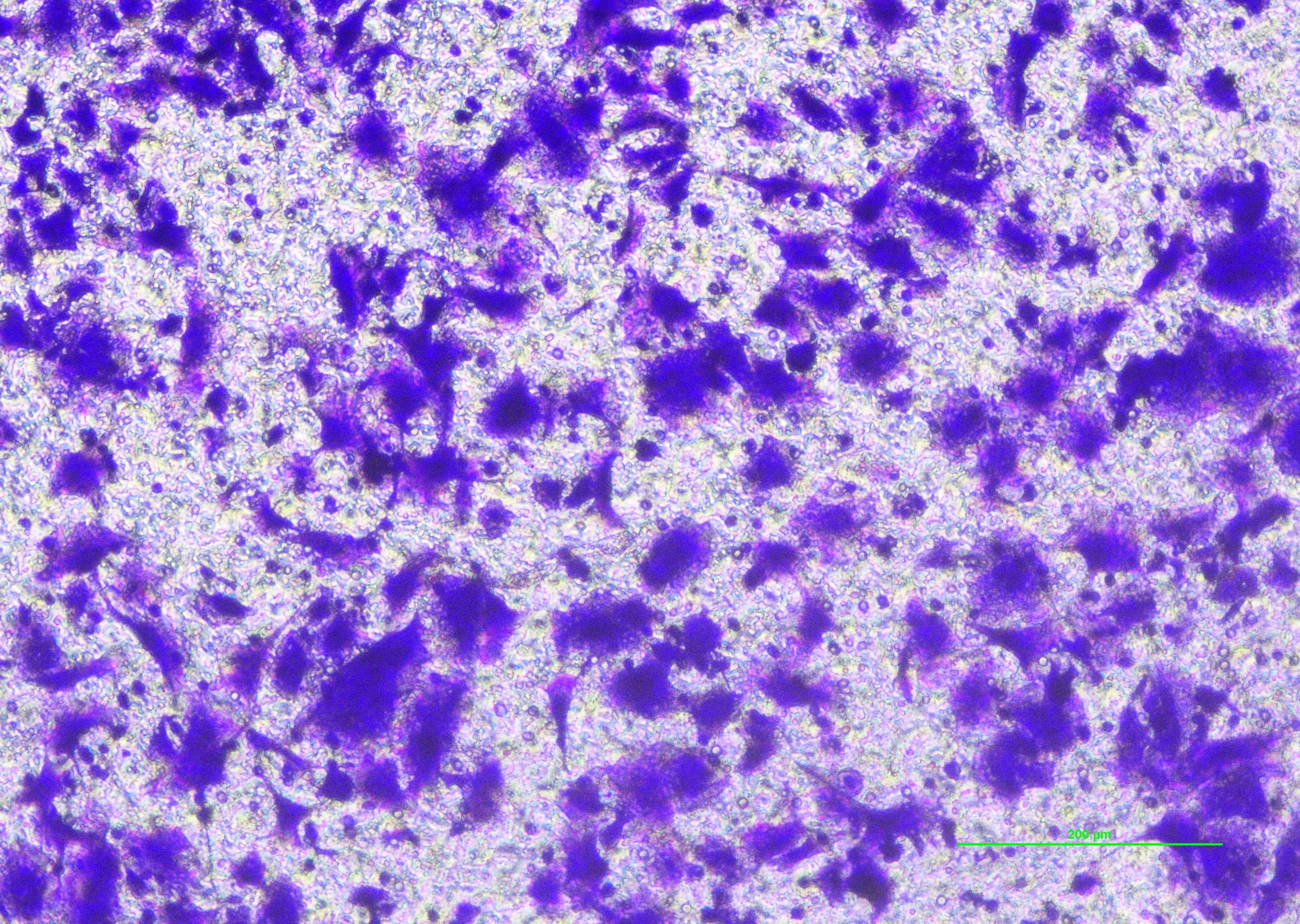

Supplement: Supplementary file 4 [file DataSheet9.ZIP › original data of transwell assay-MCF-7 cells/7m-200nm.jpg]

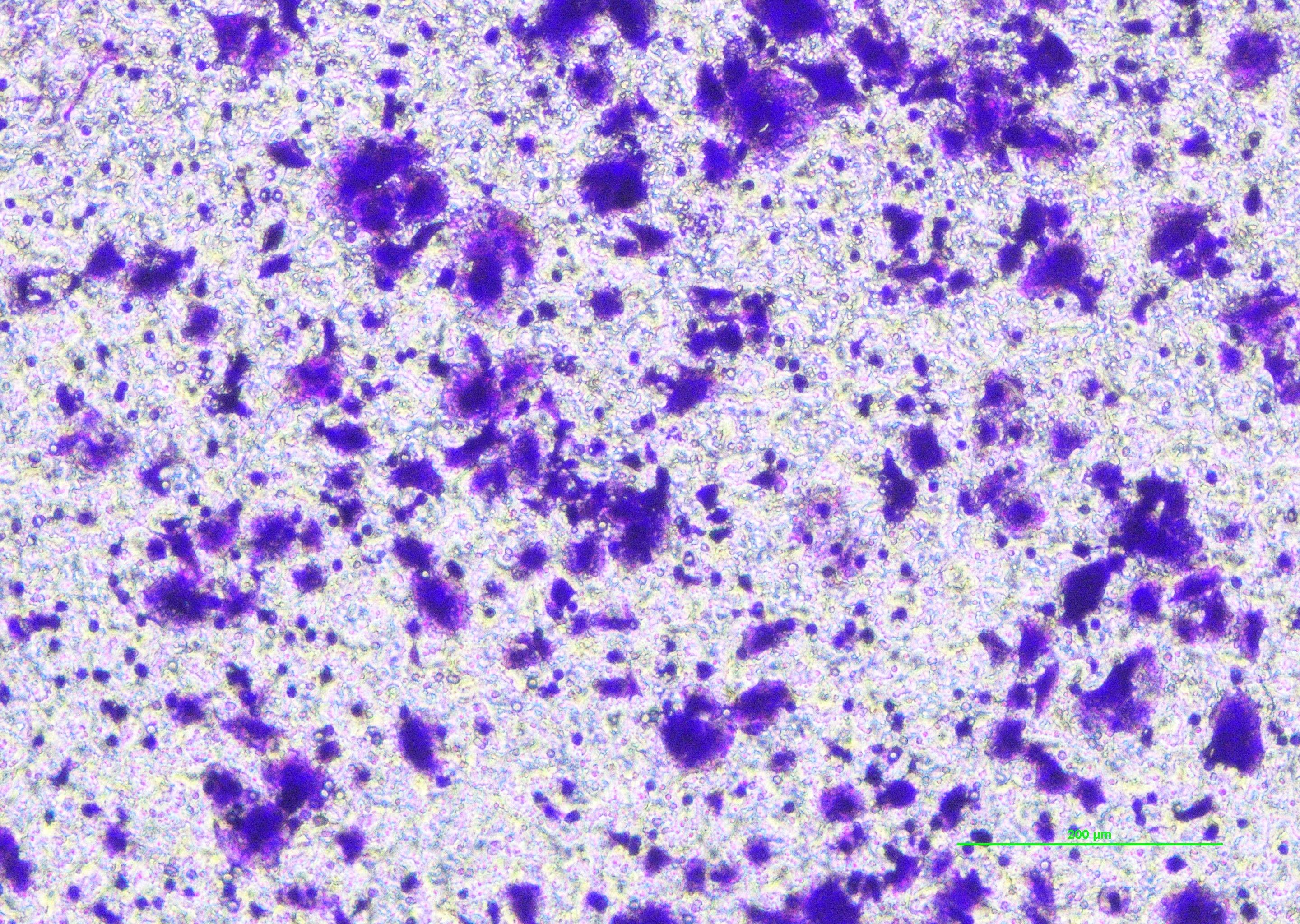

Supplement: Supplementary file 4 [file DataSheet9.ZIP › original data of transwell assay-MCF-7 cells/7m-400nm.jpg]

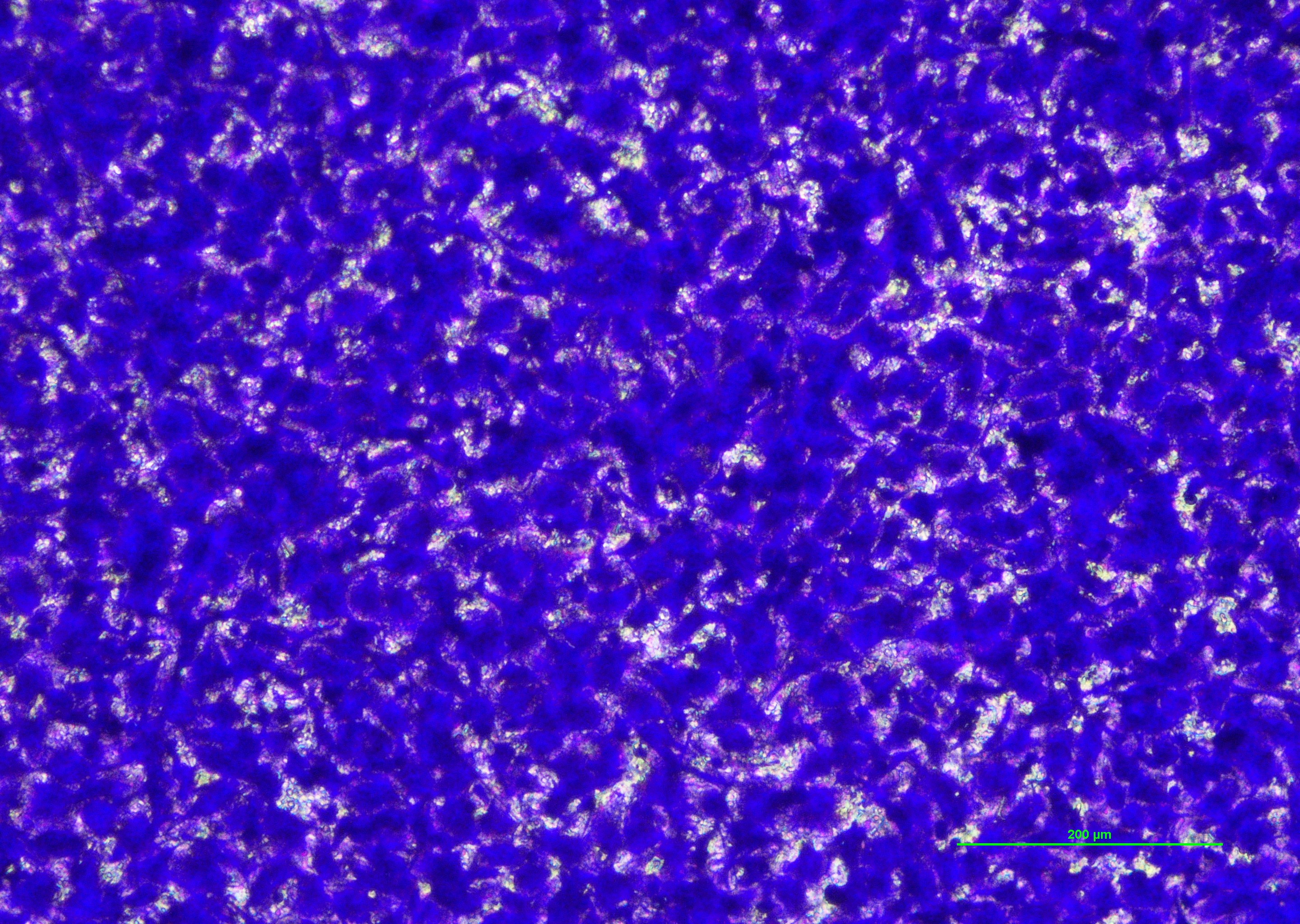

Supplement: Supplementary file 4 [file DataSheet9.ZIP › original data of transwell assay-MCF-7 cells/NC.jpg]

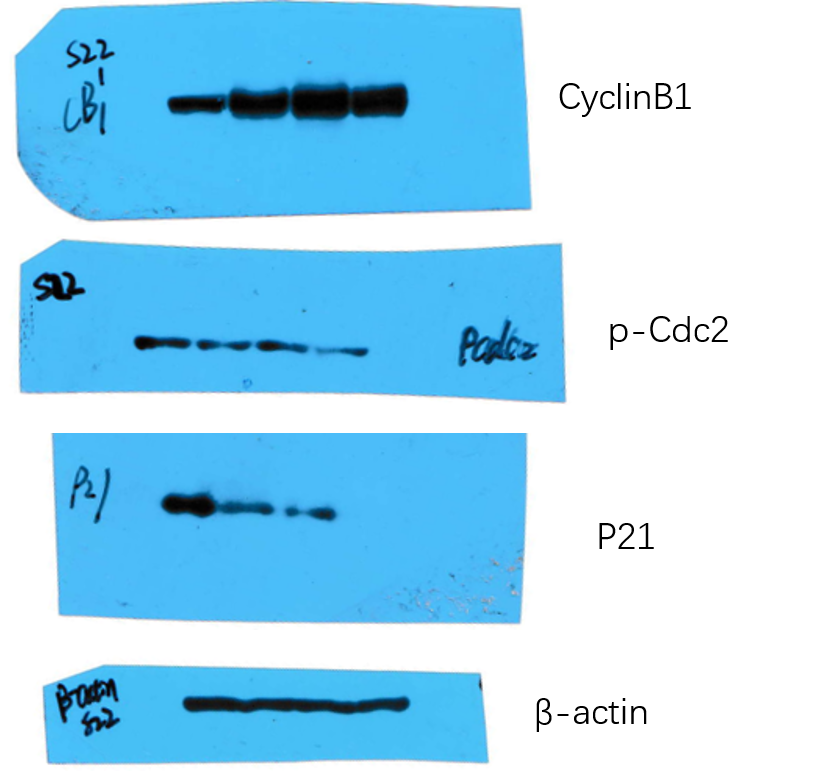

Supplement: Supplementary file 5 [file DataSheet4.ZIP › original data of cell cycle dependent/7m-cell cycle-WB.tif]

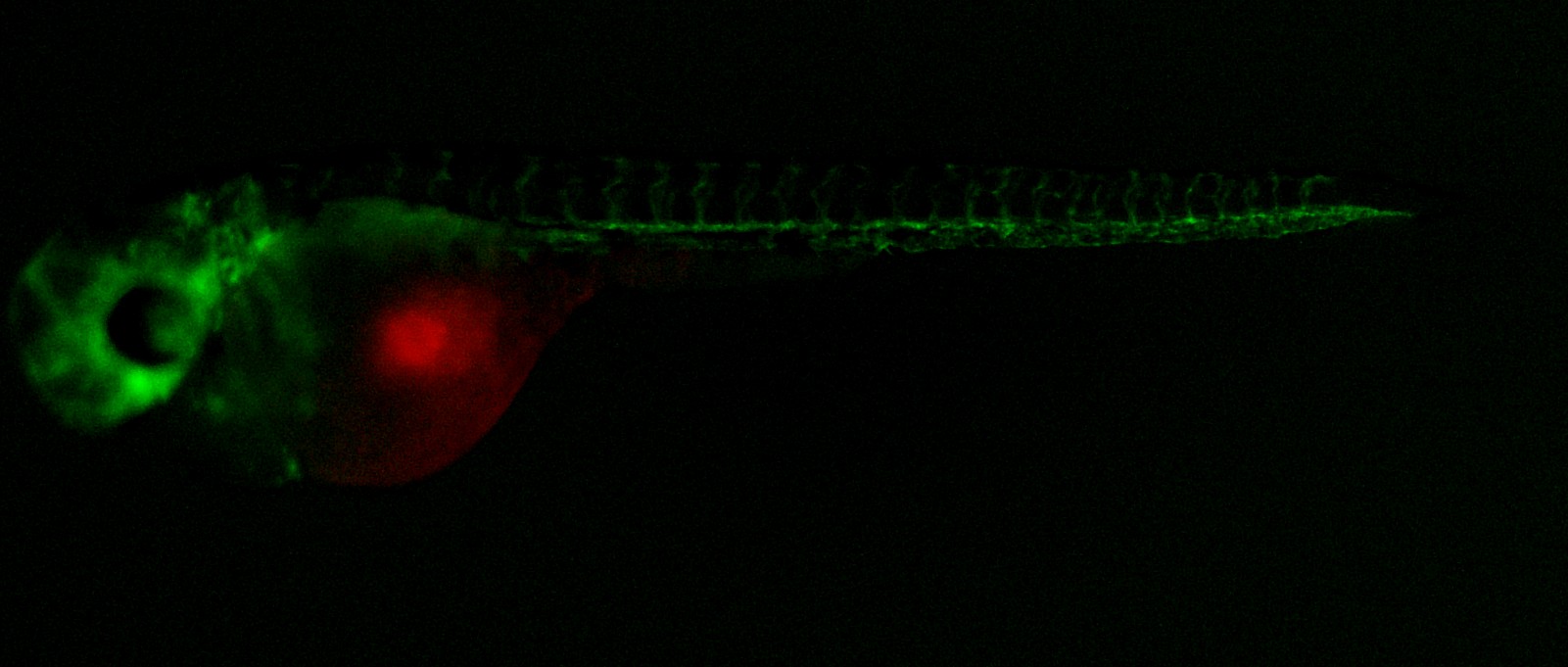

Supplement: Supplementary file 7 [file DataSheet10.ZIP › original data of zebrafish xenograft assay/7m-2.5a╠m-1.jpg]

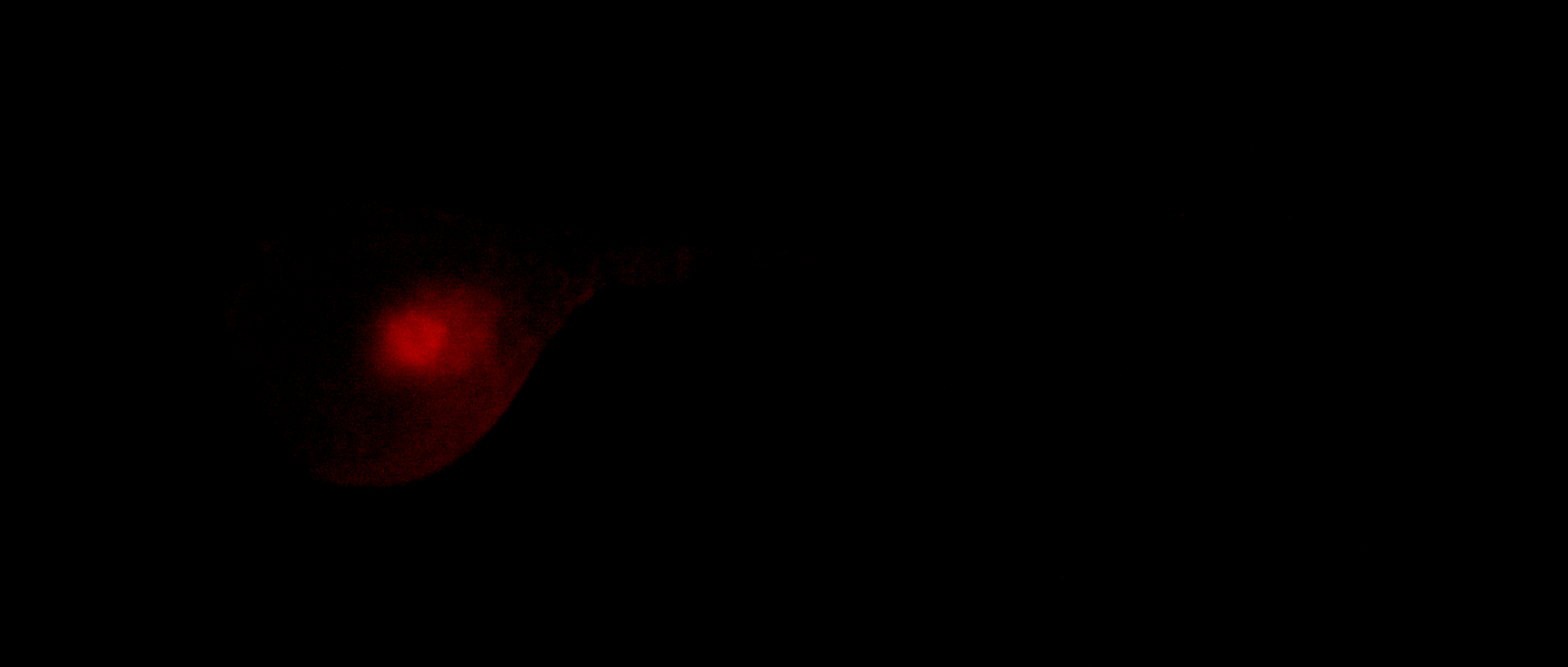

Supplement: Supplementary file 7 [file DataSheet10.ZIP › original data of zebrafish xenograft assay/7m-2.5a╠m-2.jpg]

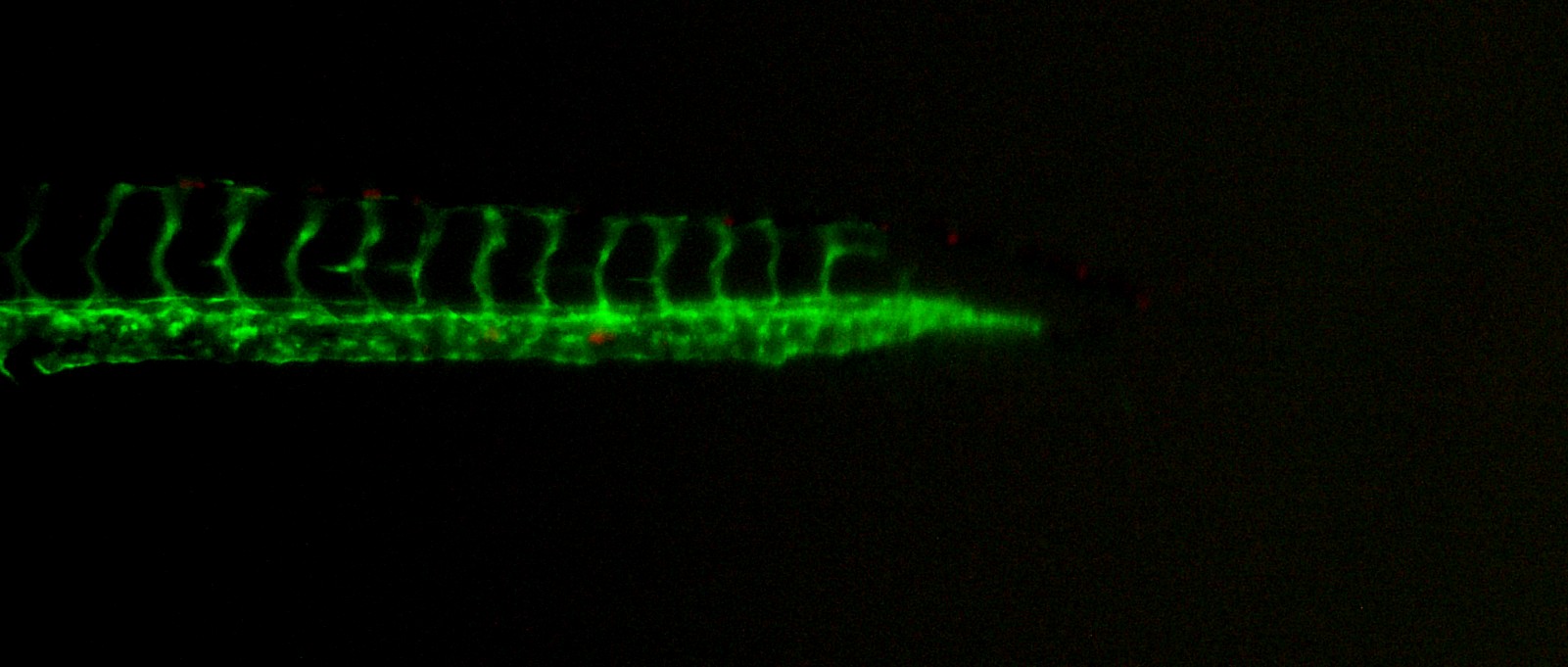

Supplement: Supplementary file 7 [file DataSheet10.ZIP › original data of zebrafish xenograft assay/7m-2.5a╠m-3.jpg]

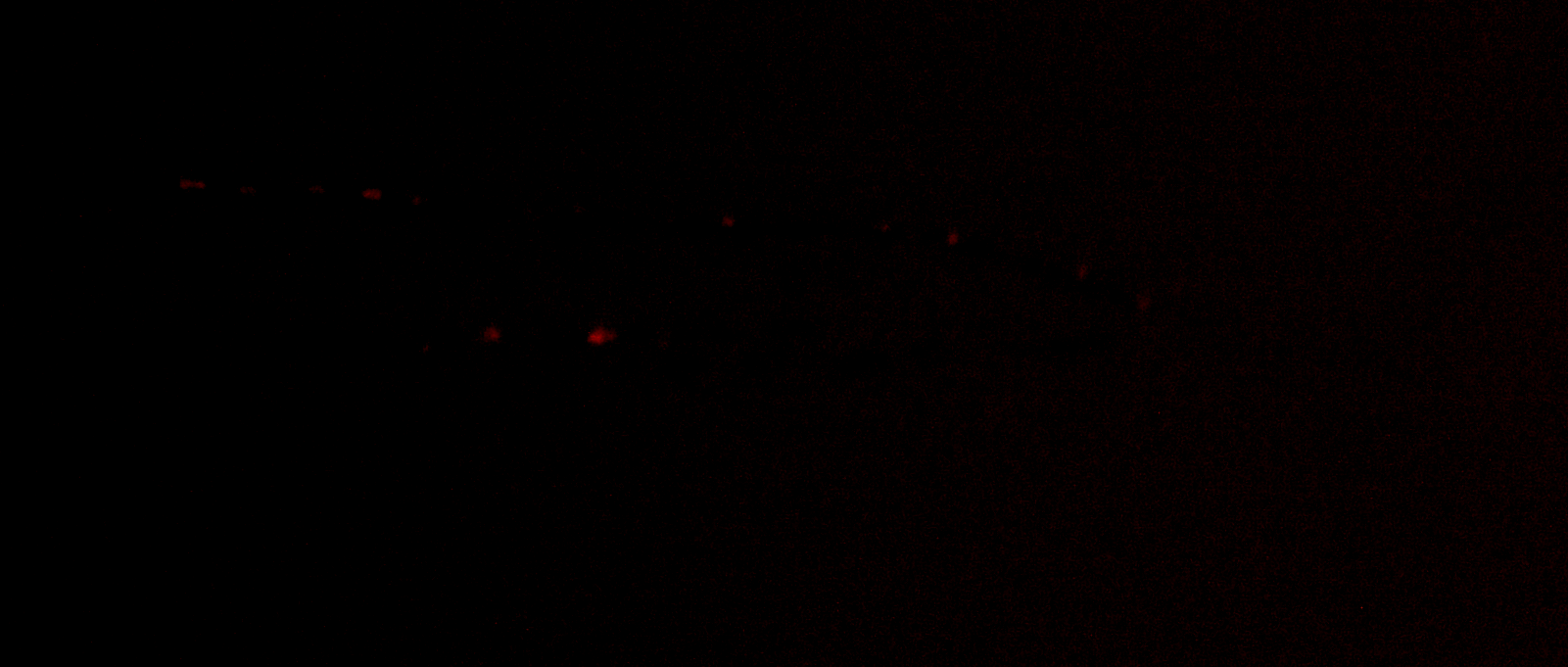

Supplement: Supplementary file 7 [file DataSheet10.ZIP › original data of zebrafish xenograft assay/7m-2.5a╠m-4.jpg]

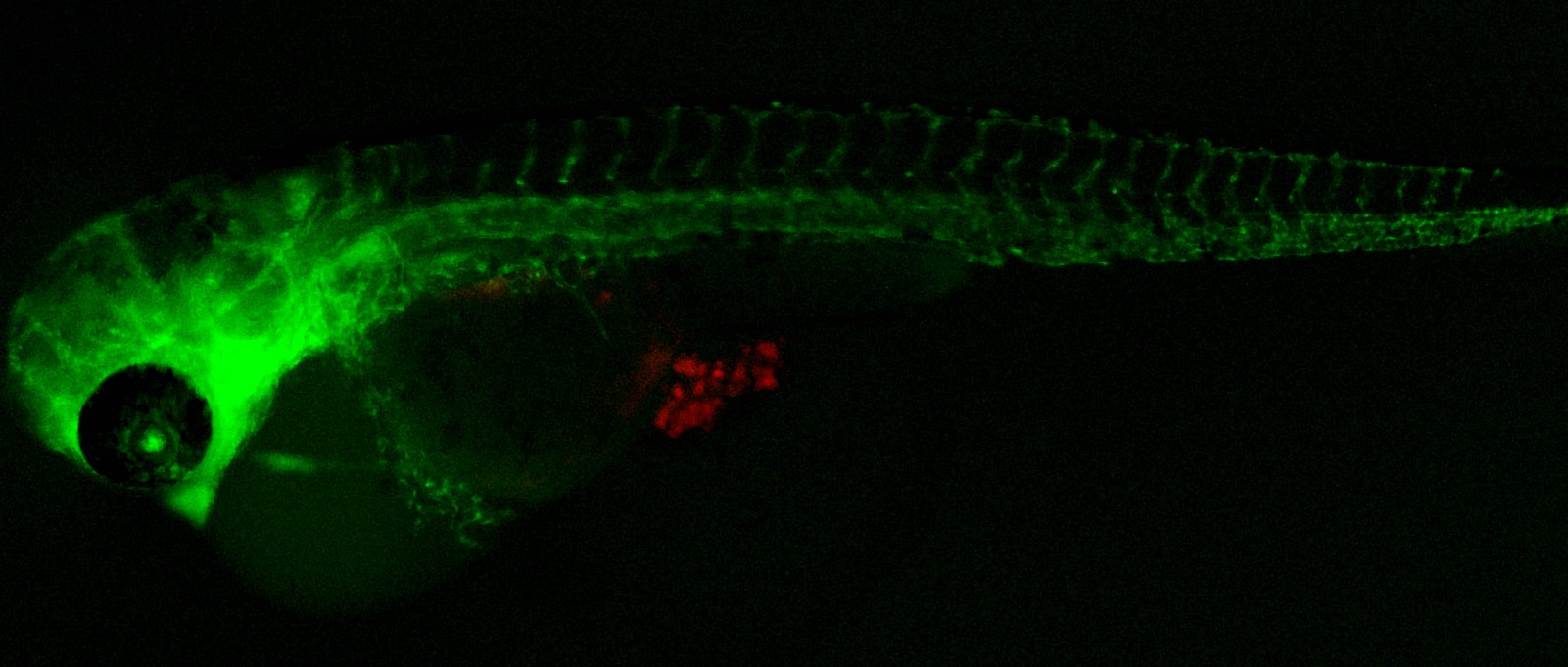

Supplement: Supplementary file 7 [file DataSheet10.ZIP › original data of zebrafish xenograft assay/7m-5a╠m-1.jpg]

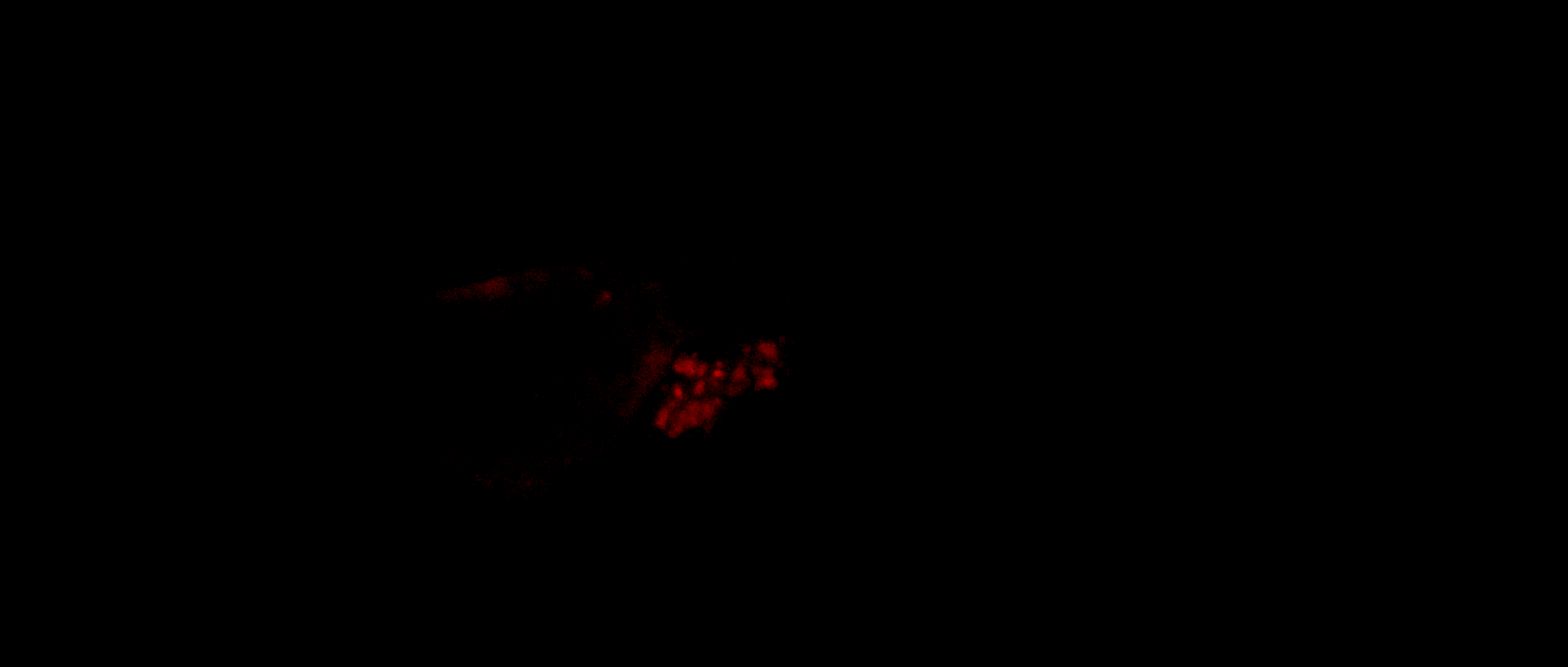

Supplement: Supplementary file 7 [file DataSheet10.ZIP › original data of zebrafish xenograft assay/7m-5a╠m-2.jpg]

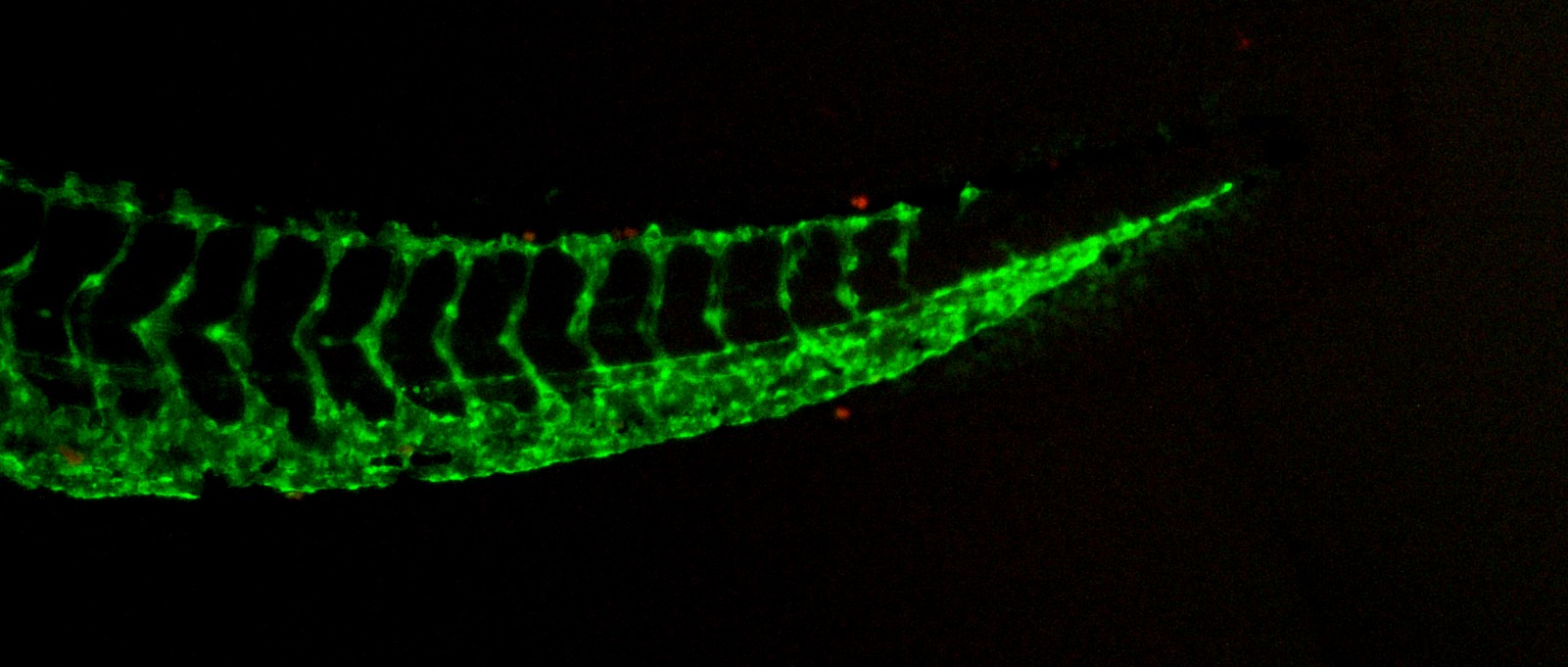

Supplement: Supplementary file 7 [file DataSheet10.ZIP › original data of zebrafish xenograft assay/7m-5a╠m-3.jpg]

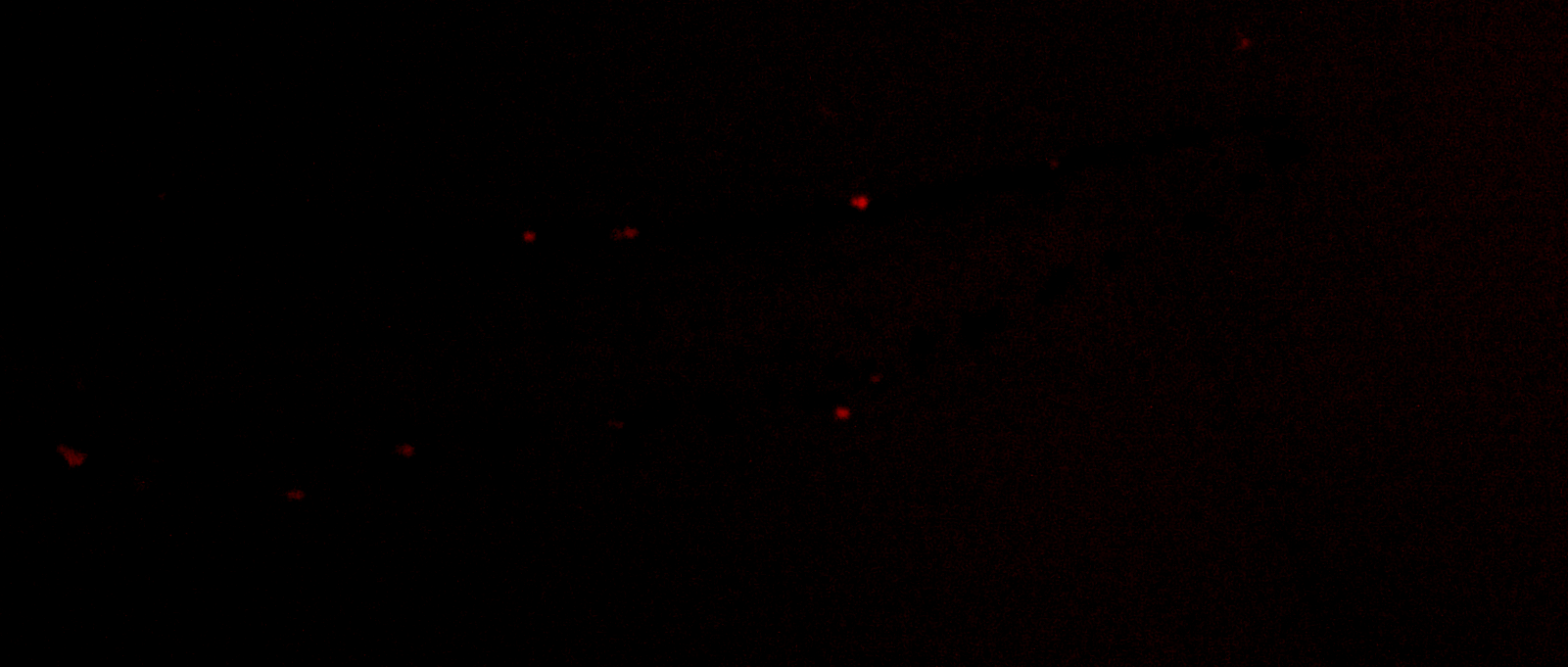

Supplement: Supplementary file 7 [file DataSheet10.ZIP › original data of zebrafish xenograft assay/7m-5a╠m-4.jpg]

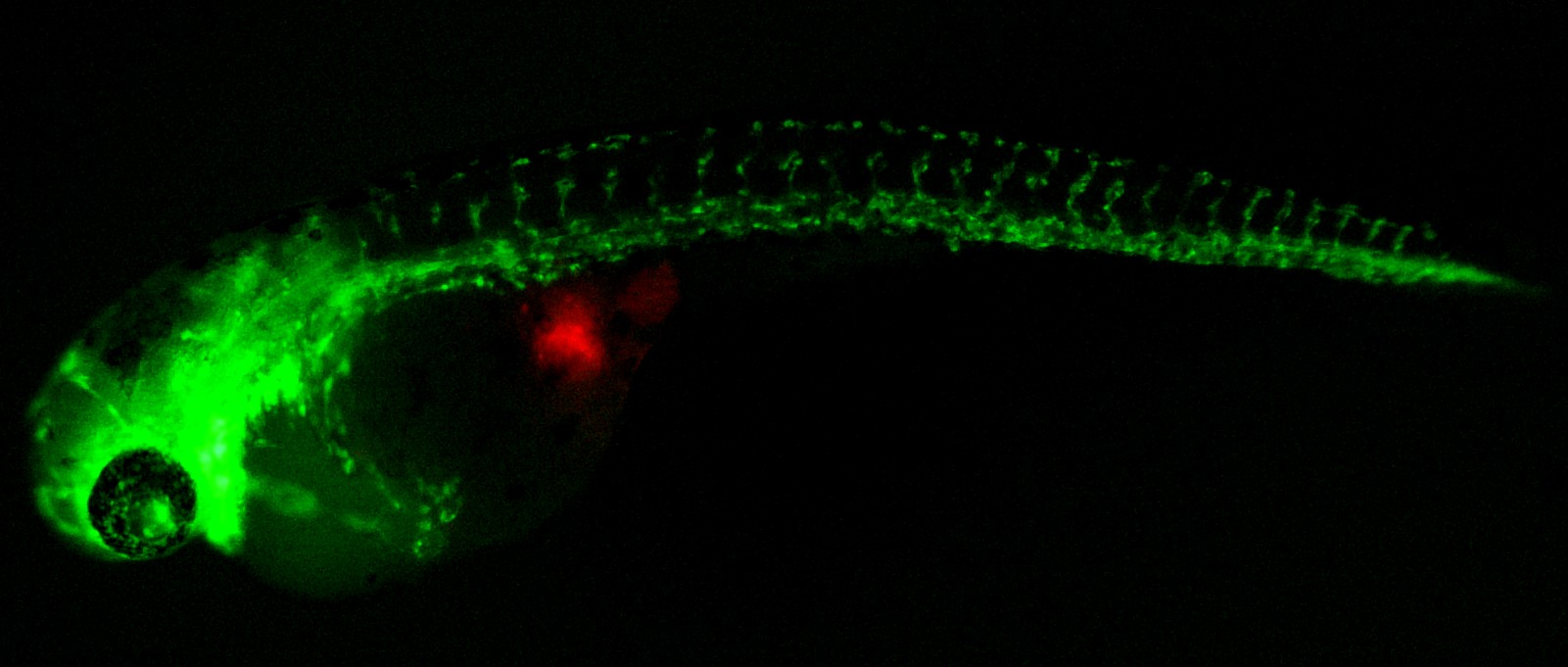

Supplement: Supplementary file 7 [file DataSheet10.ZIP › original data of zebrafish xenograft assay/7m-7.5a╠m-1.jpg]

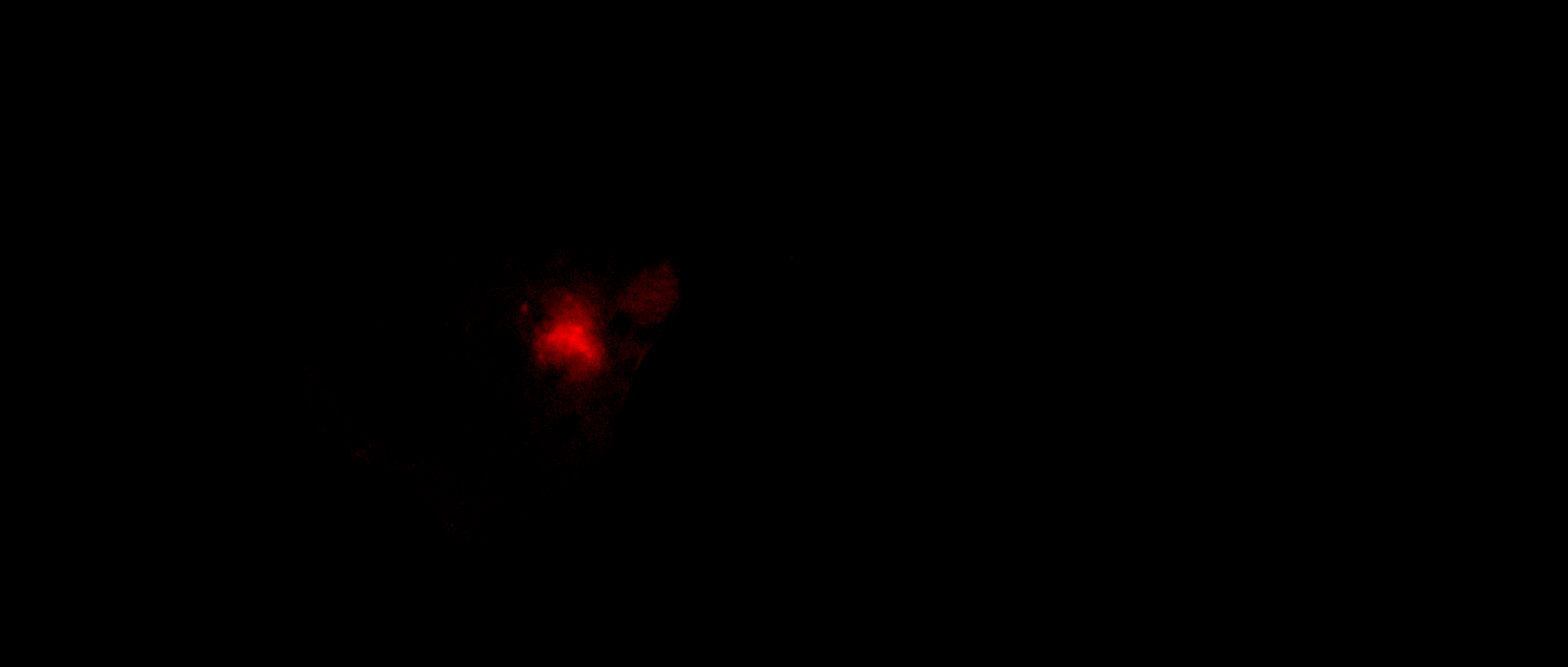

Supplement: Supplementary file 7 [file DataSheet10.ZIP › original data of zebrafish xenograft assay/7m-7.5a╠m-2.jpg]

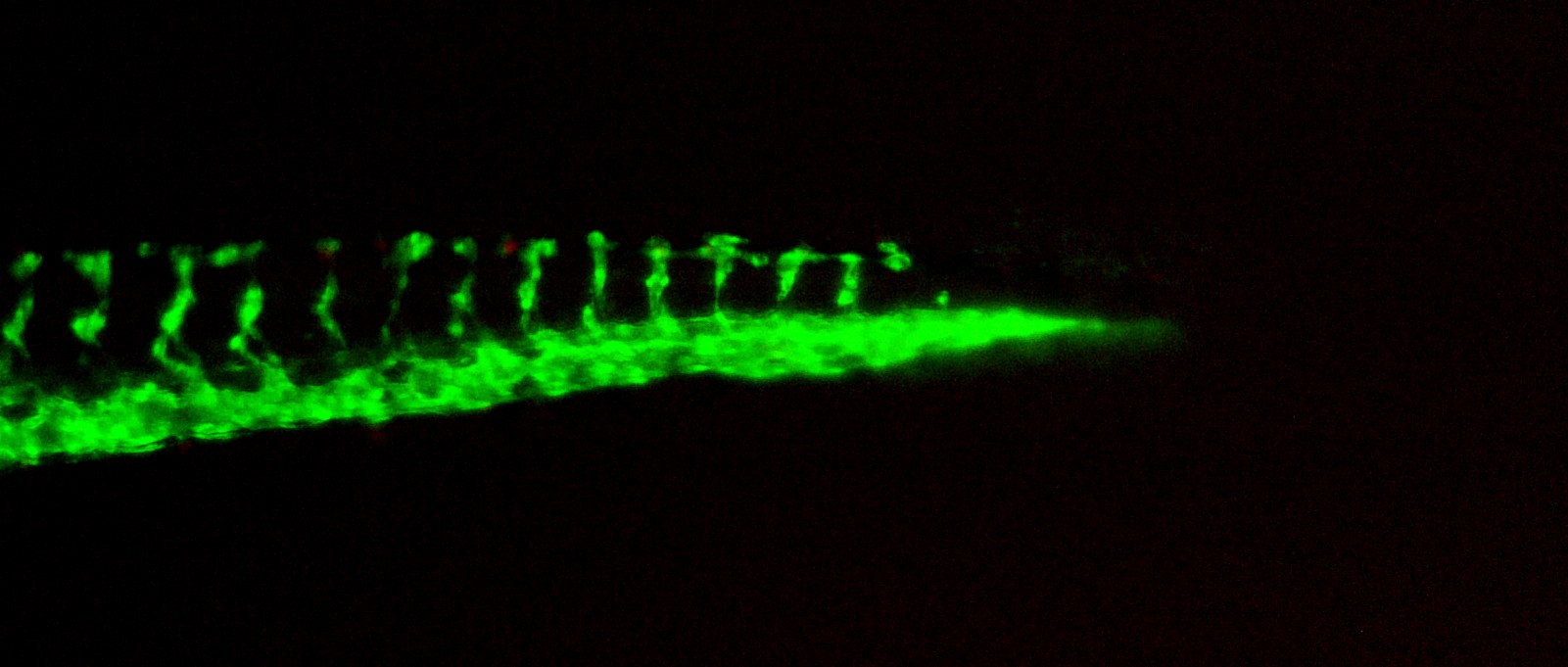

Supplement: Supplementary file 7 [file DataSheet10.ZIP › original data of zebrafish xenograft assay/7m-7.5a╠m-3.jpg]

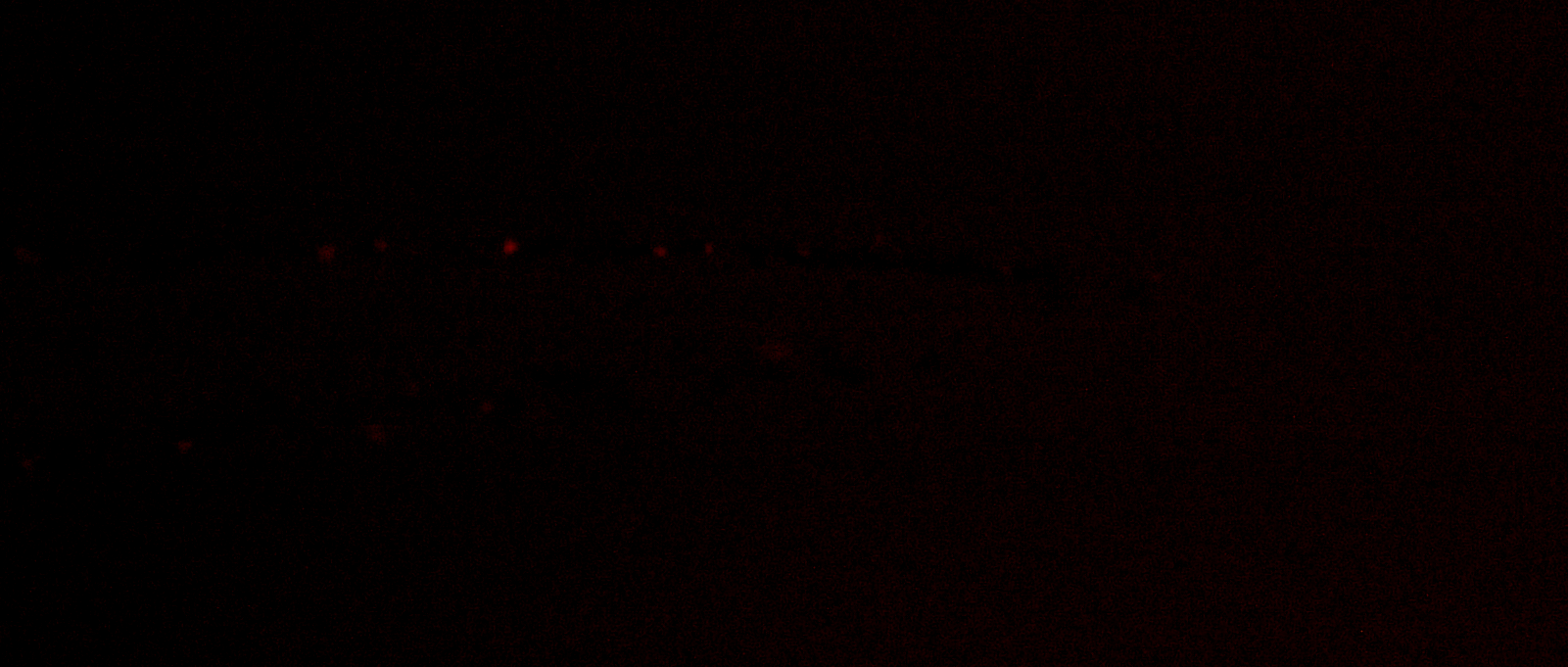

Supplement: Supplementary file 7 [file DataSheet10.ZIP › original data of zebrafish xenograft assay/7m-7.5a╠m-4.jpg]

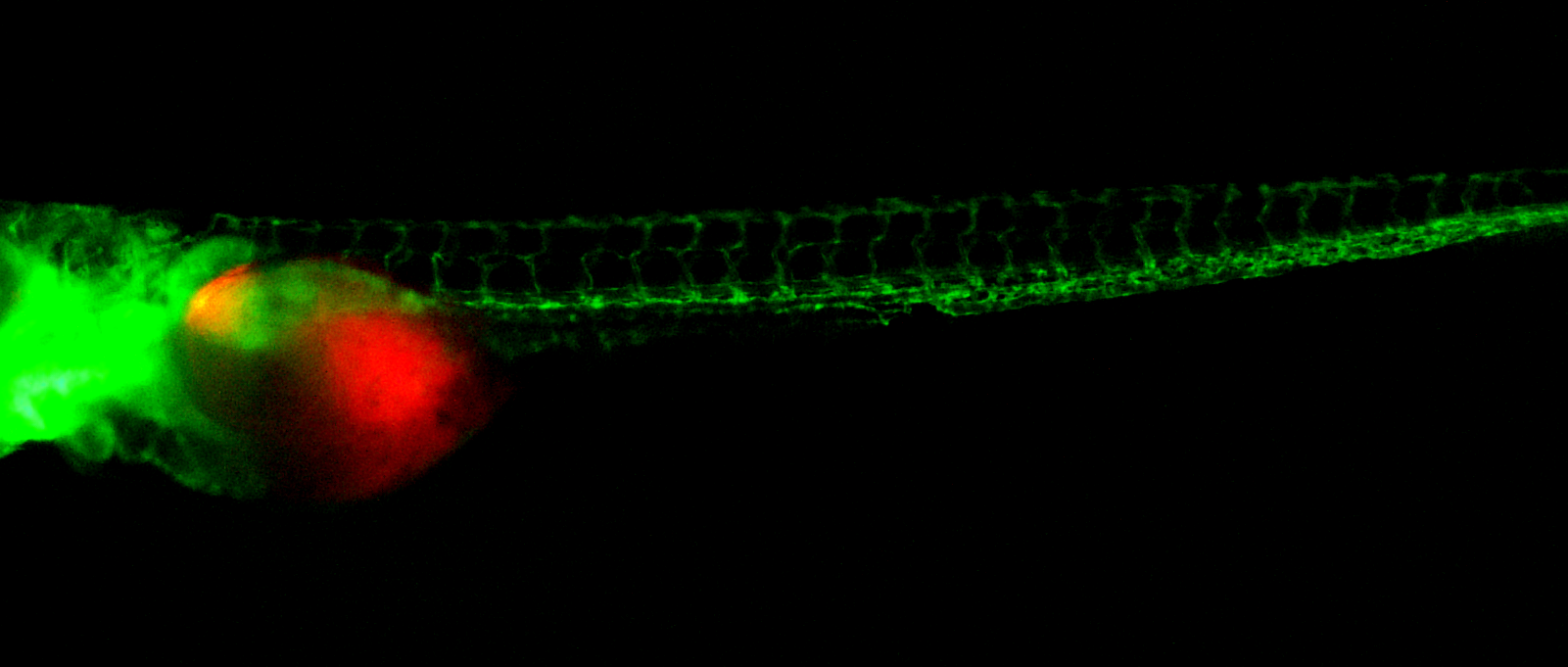

Supplement: Supplementary file 7 [file DataSheet10.ZIP › original data of zebrafish xenograft assay/NC-1.jpg]

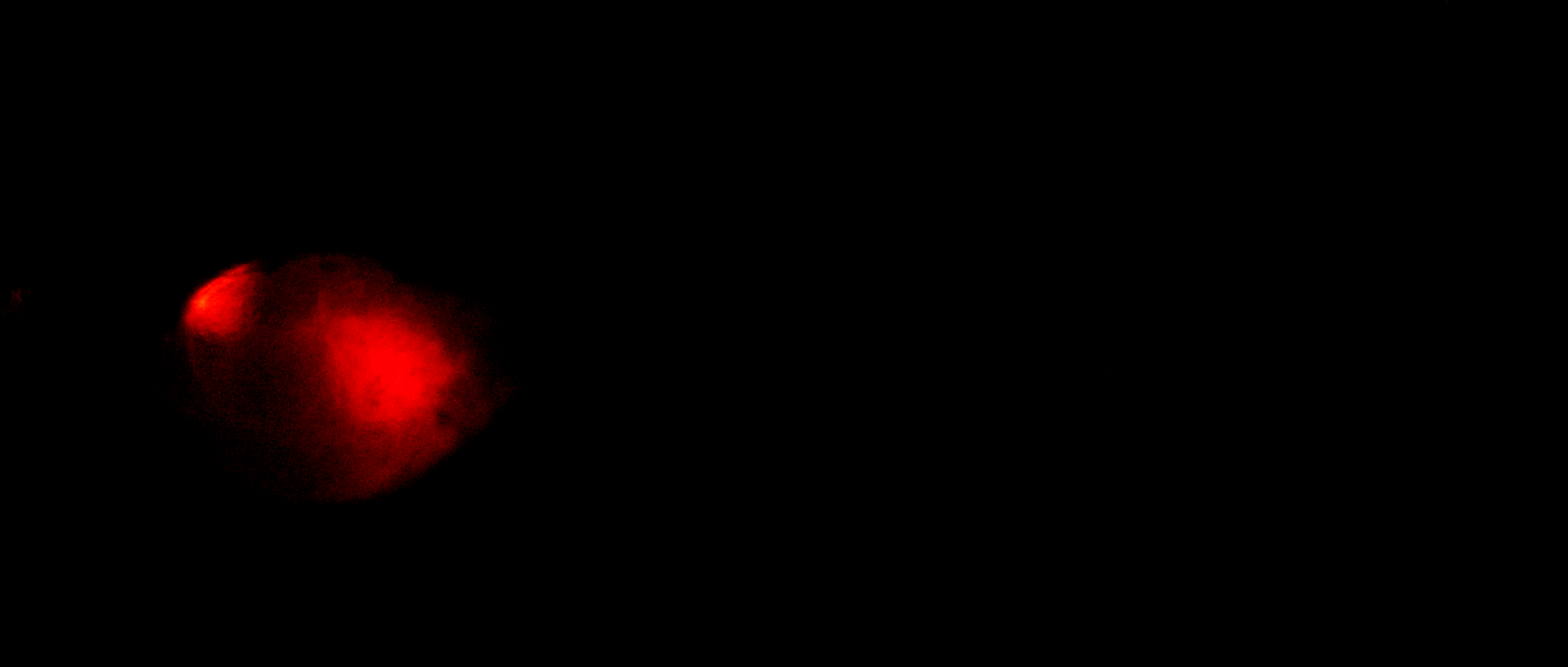

Supplement: Supplementary file 7 [file DataSheet10.ZIP › original data of zebrafish xenograft assay/NC-2.jpg]

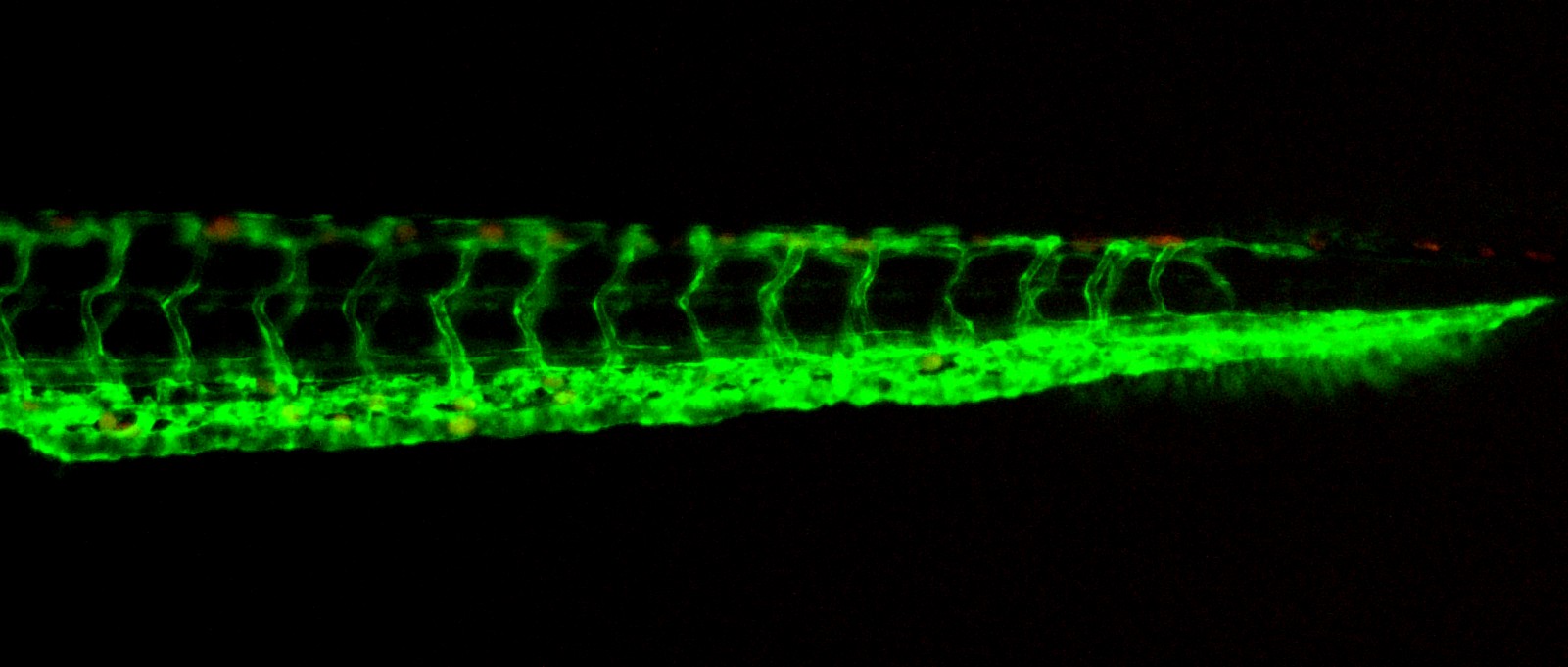

Supplement: Supplementary file 7 [file DataSheet10.ZIP › original data of zebrafish xenograft assay/NC-3.jpg]

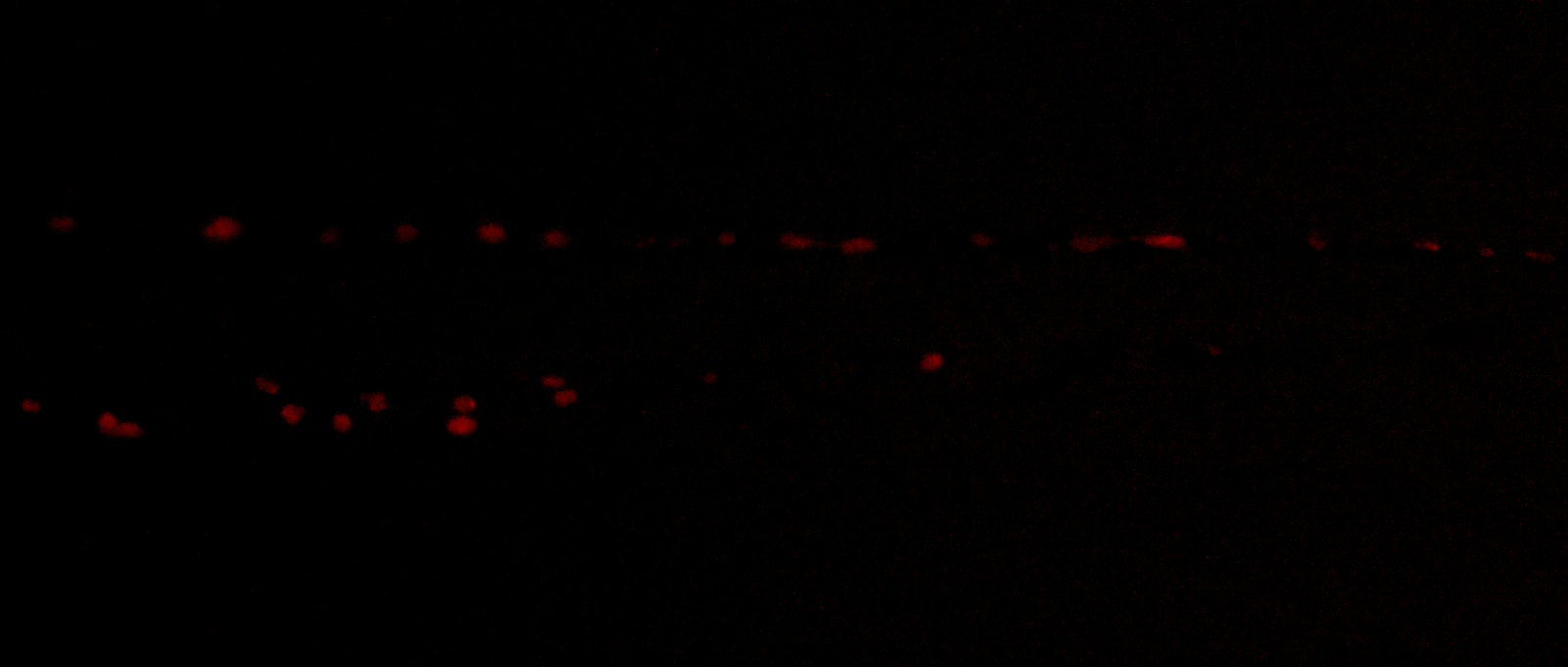

Supplement: Supplementary file 7 [file DataSheet10.ZIP › original data of zebrafish xenograft assay/NC-4.jpg]

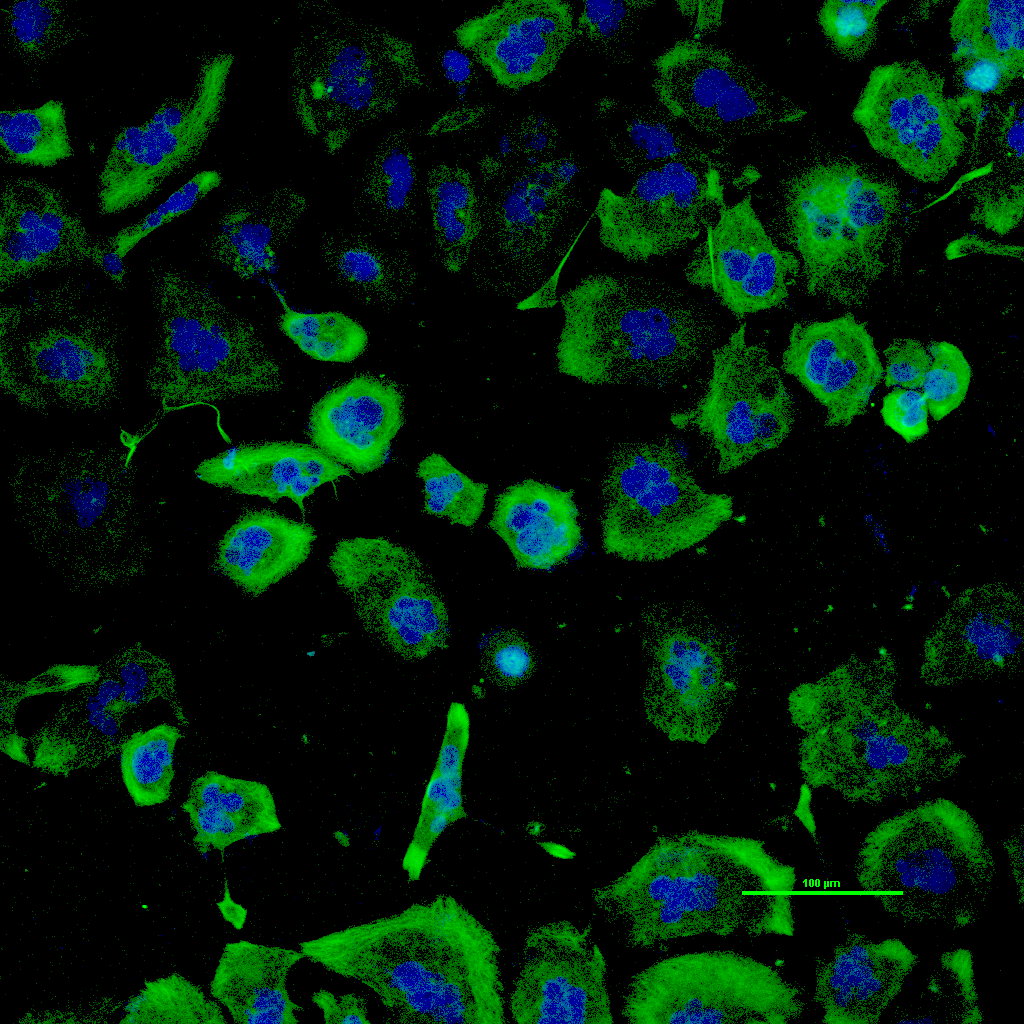

Supplement: Supplementary file 8 [file DataSheet6.ZIP › original data of immunofluorescence assay/7m-100_RGB.tif]

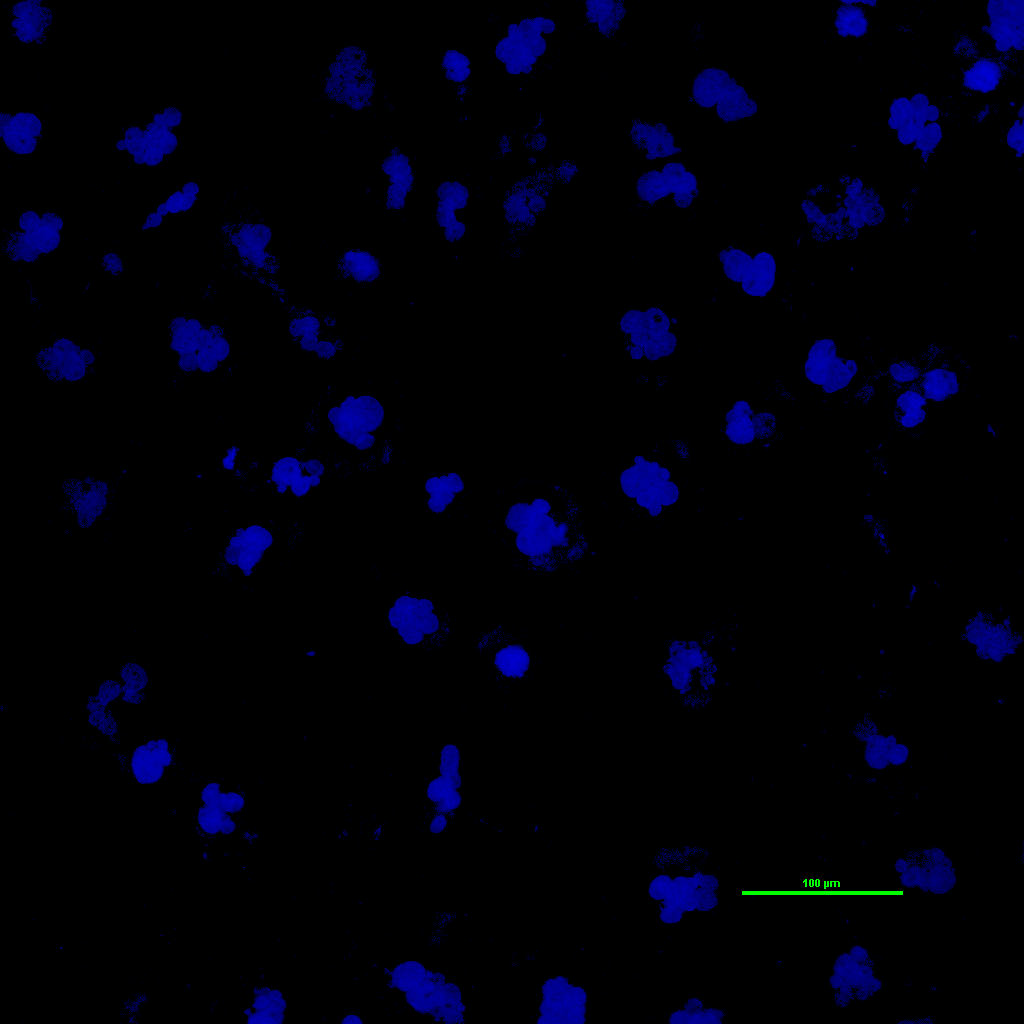

Supplement: Supplementary file 8 [file DataSheet6.ZIP › original data of immunofluorescence assay/7m-100_RGB_DAPI.tif]

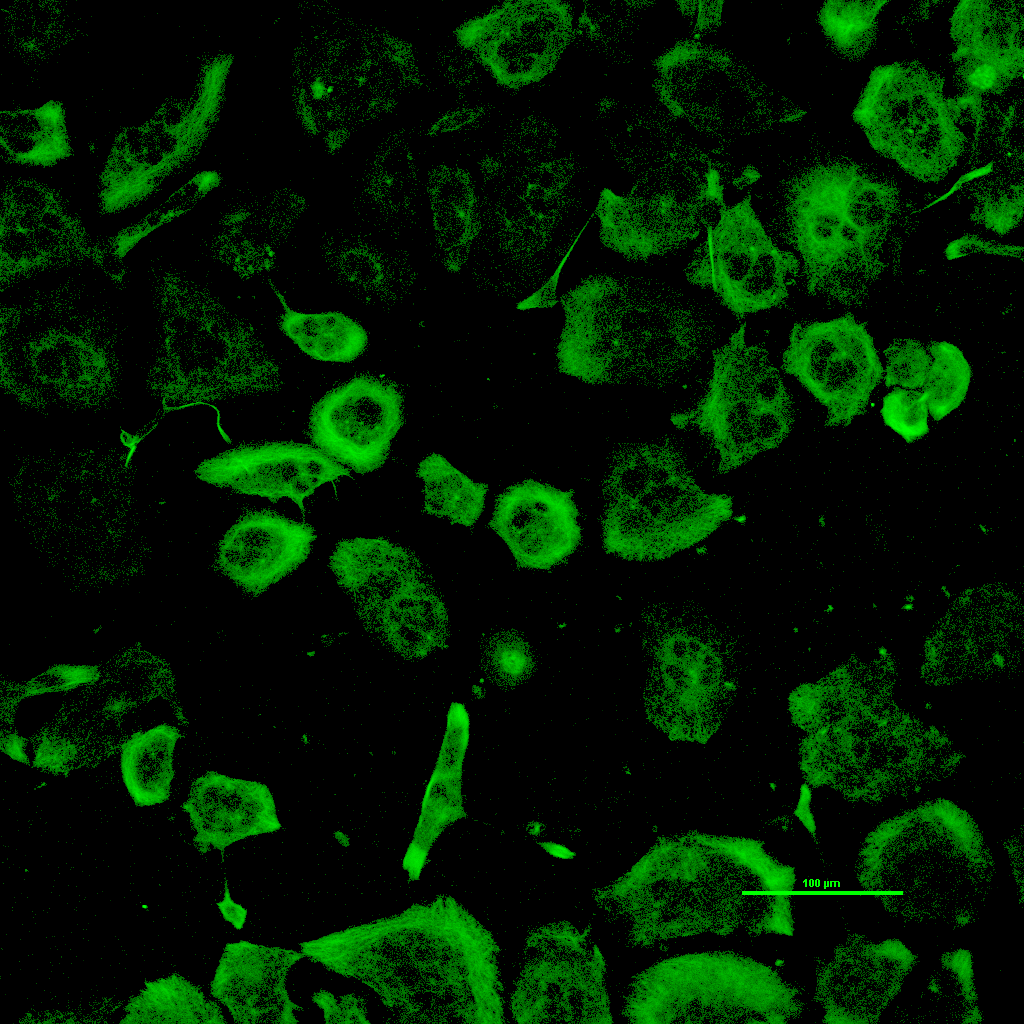

Supplement: Supplementary file 8 [file DataSheet6.ZIP › original data of immunofluorescence assay/7m-100_RGB_FITC.tif]

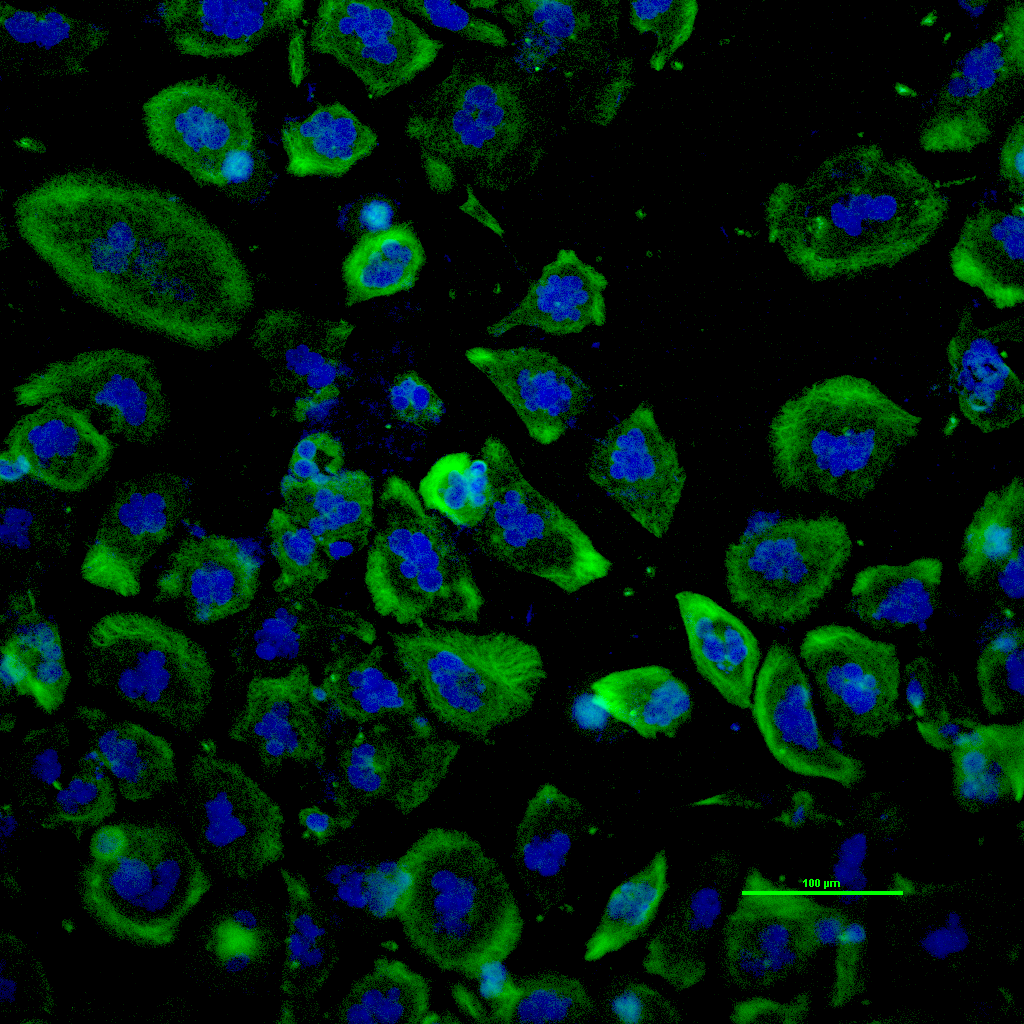

Supplement: Supplementary file 8 [file DataSheet6.ZIP › original data of immunofluorescence assay/7m-100-2_RGB.tif]

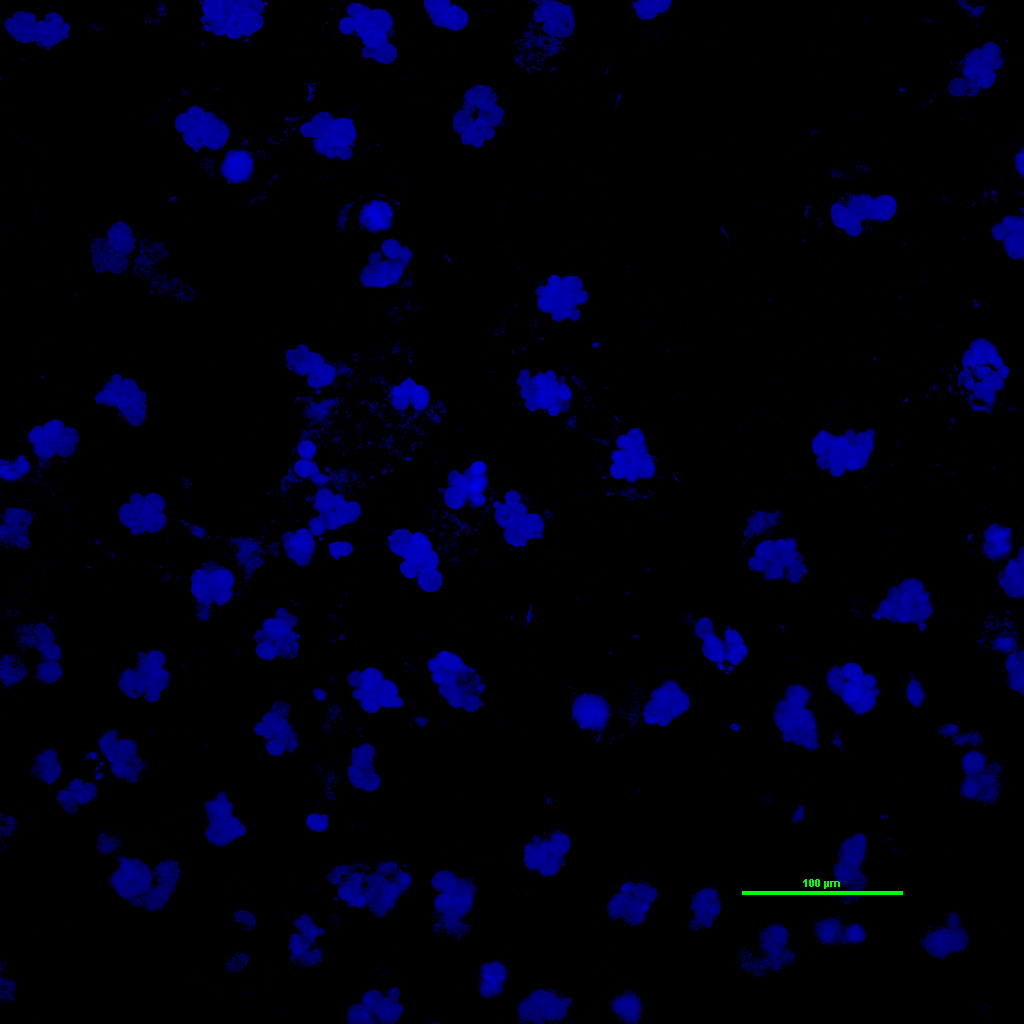

Supplement: Supplementary file 8 [file DataSheet6.ZIP › original data of immunofluorescence assay/7m-100-2_RGB_DAPI.tif]

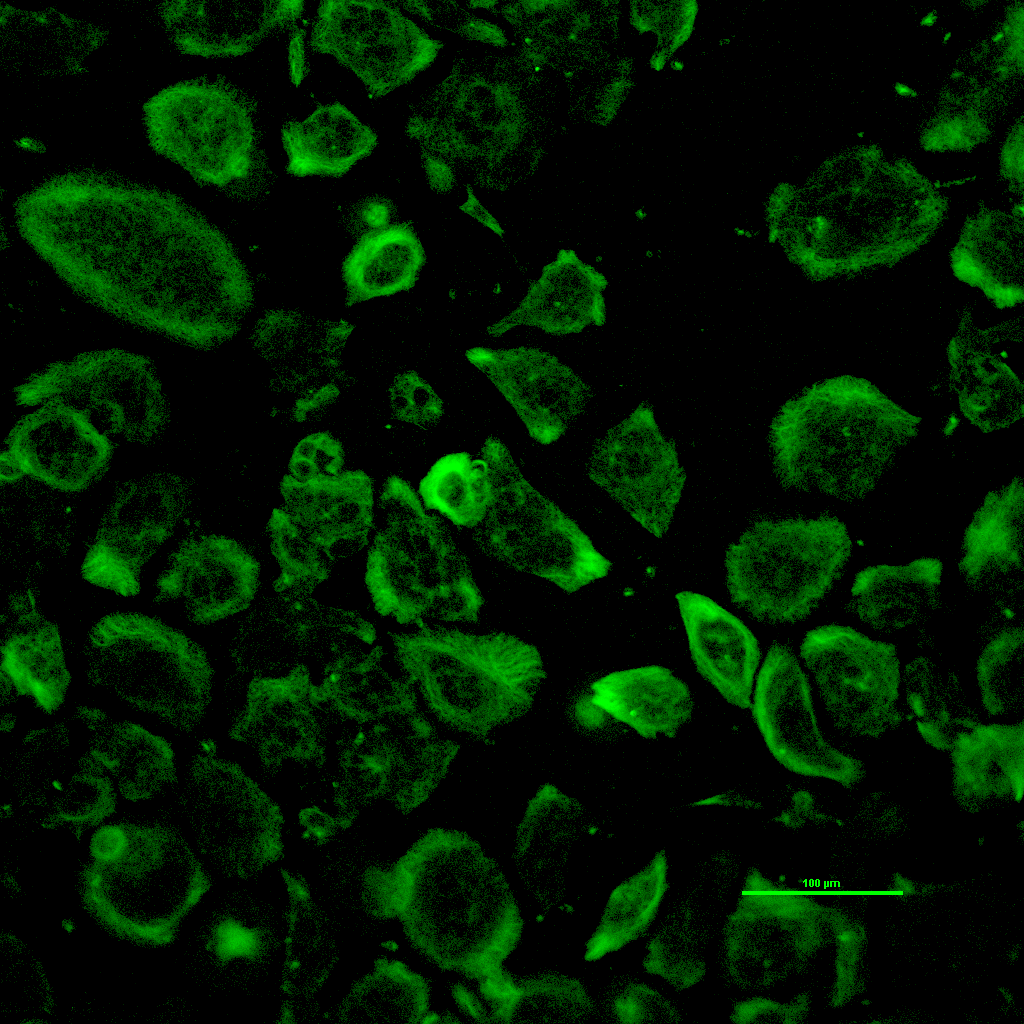

Supplement: Supplementary file 8 [file DataSheet6.ZIP › original data of immunofluorescence assay/7m-100-2_RGB_FITC.tif]

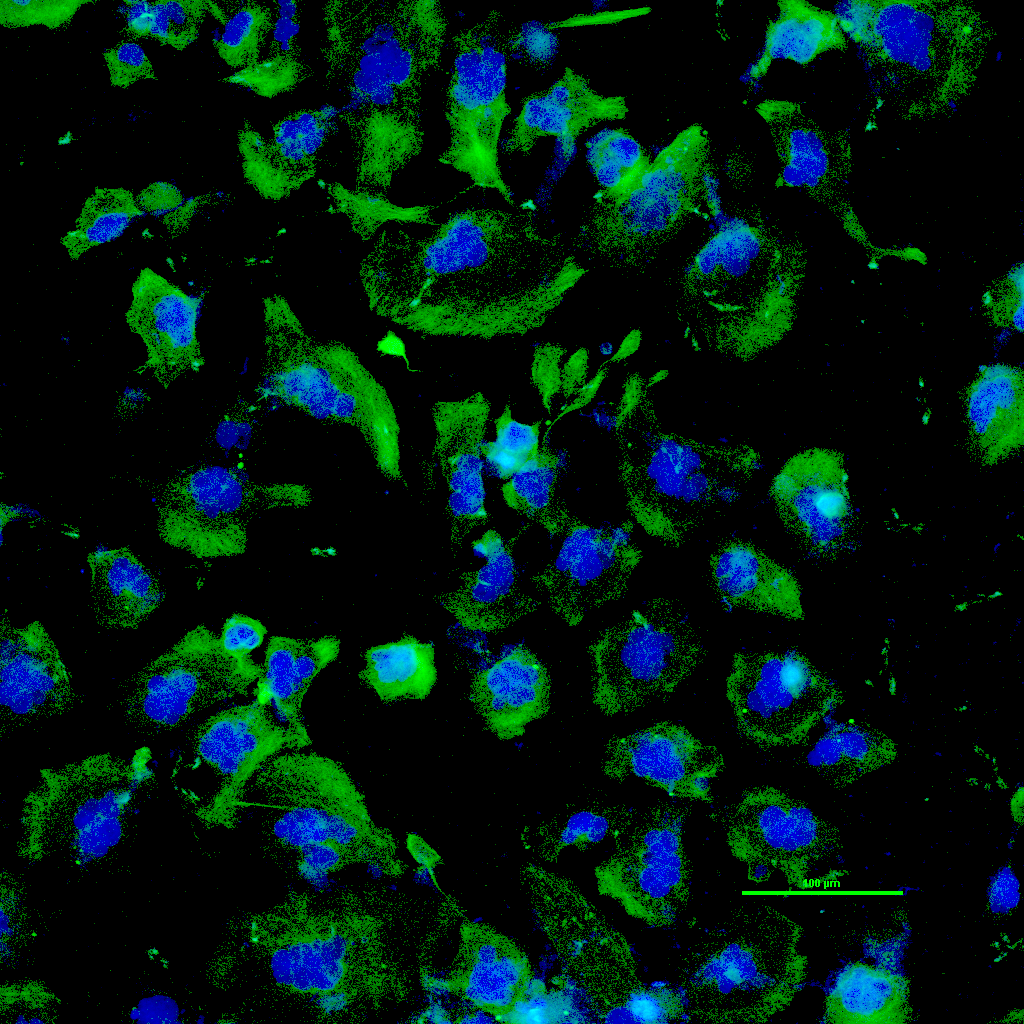

Supplement: Supplementary file 8 [file DataSheet6.ZIP › original data of immunofluorescence assay/7m-200_RGB.tif]

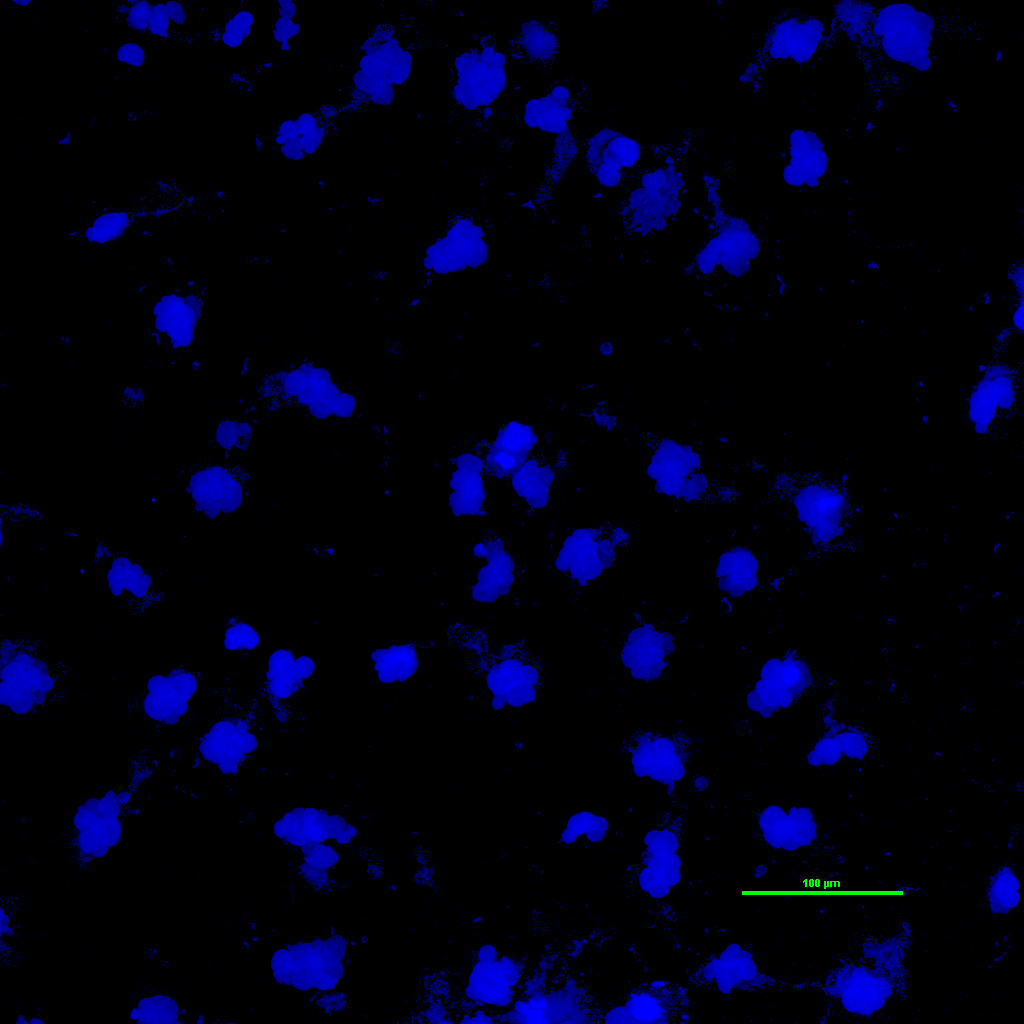

Supplement: Supplementary file 8 [file DataSheet6.ZIP › original data of immunofluorescence assay/7m-200_RGB_DAPI.tif]

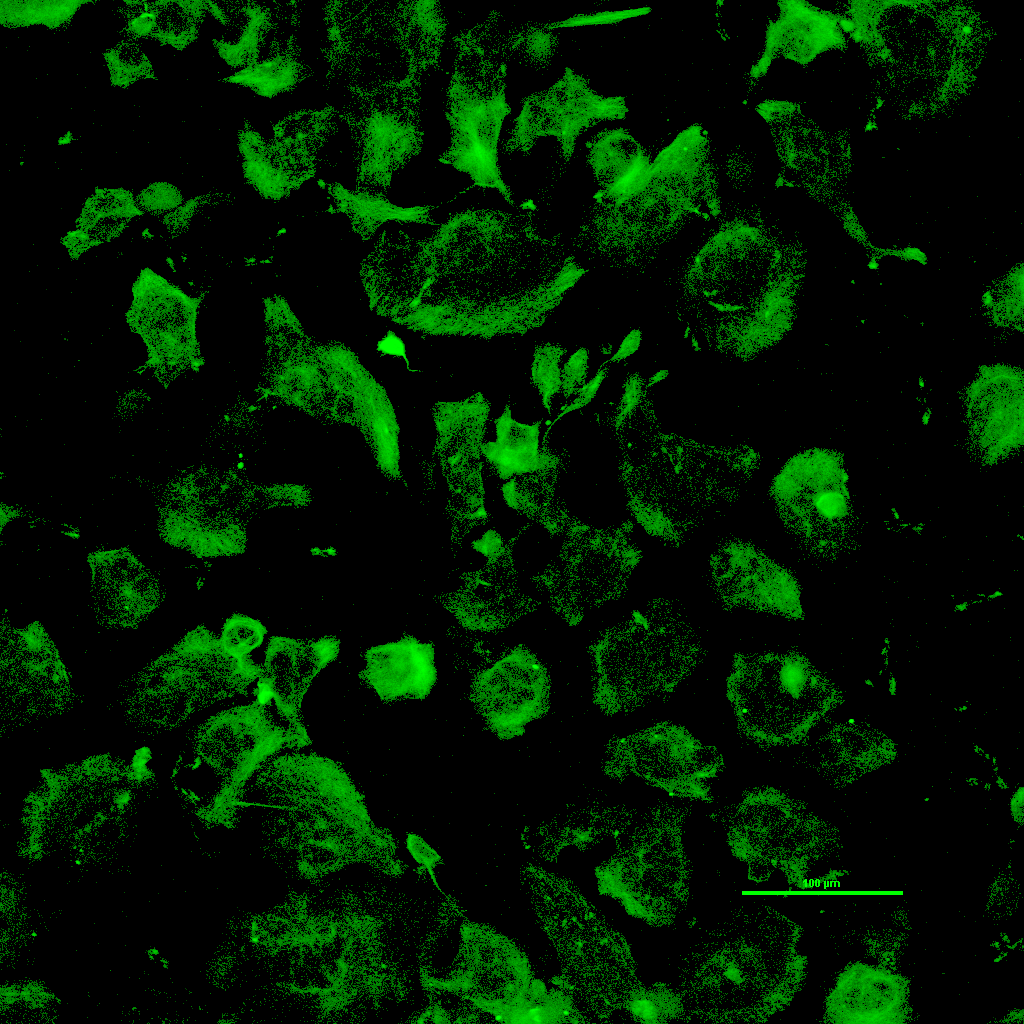

Supplement: Supplementary file 8 [file DataSheet6.ZIP › original data of immunofluorescence assay/7m-200_RGB_FITC.tif]

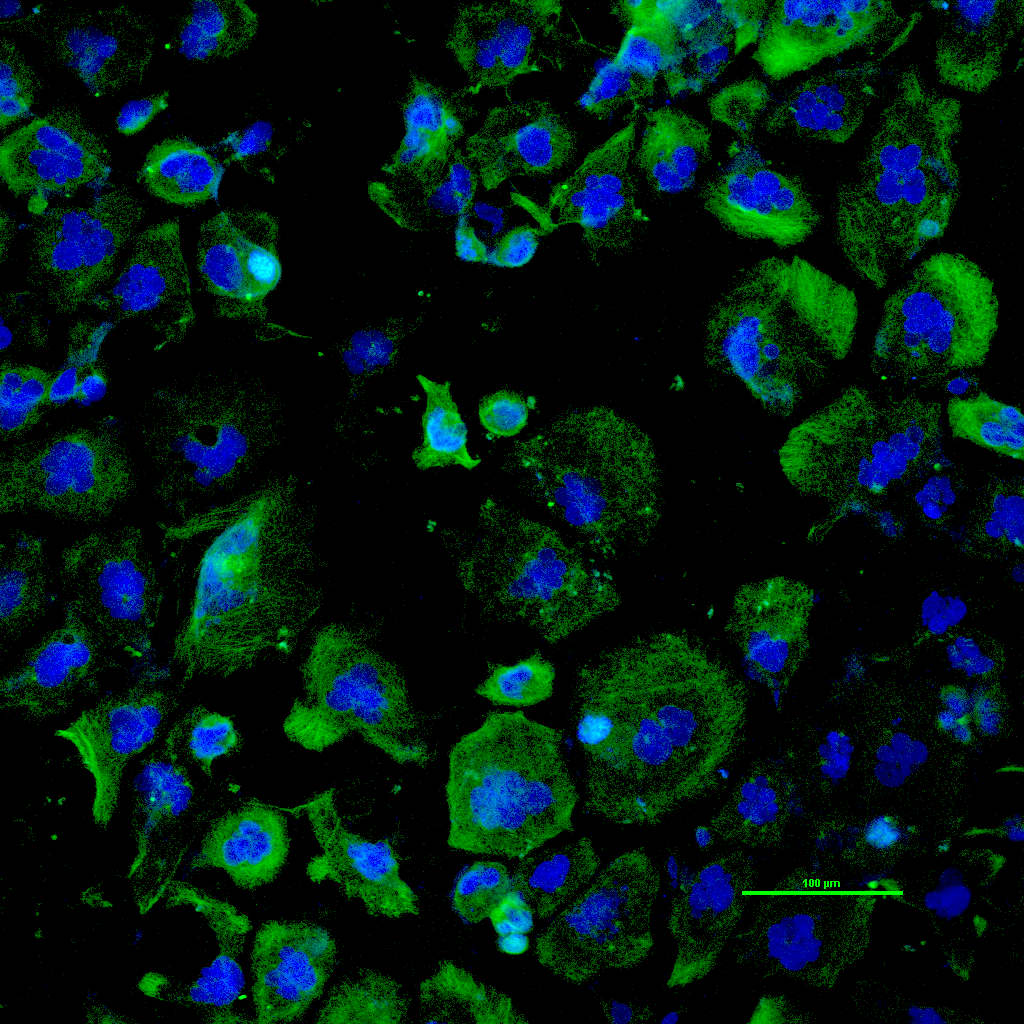

Supplement: Supplementary file 8 [file DataSheet6.ZIP › original data of immunofluorescence assay/7m-200-2_RGB.tif]

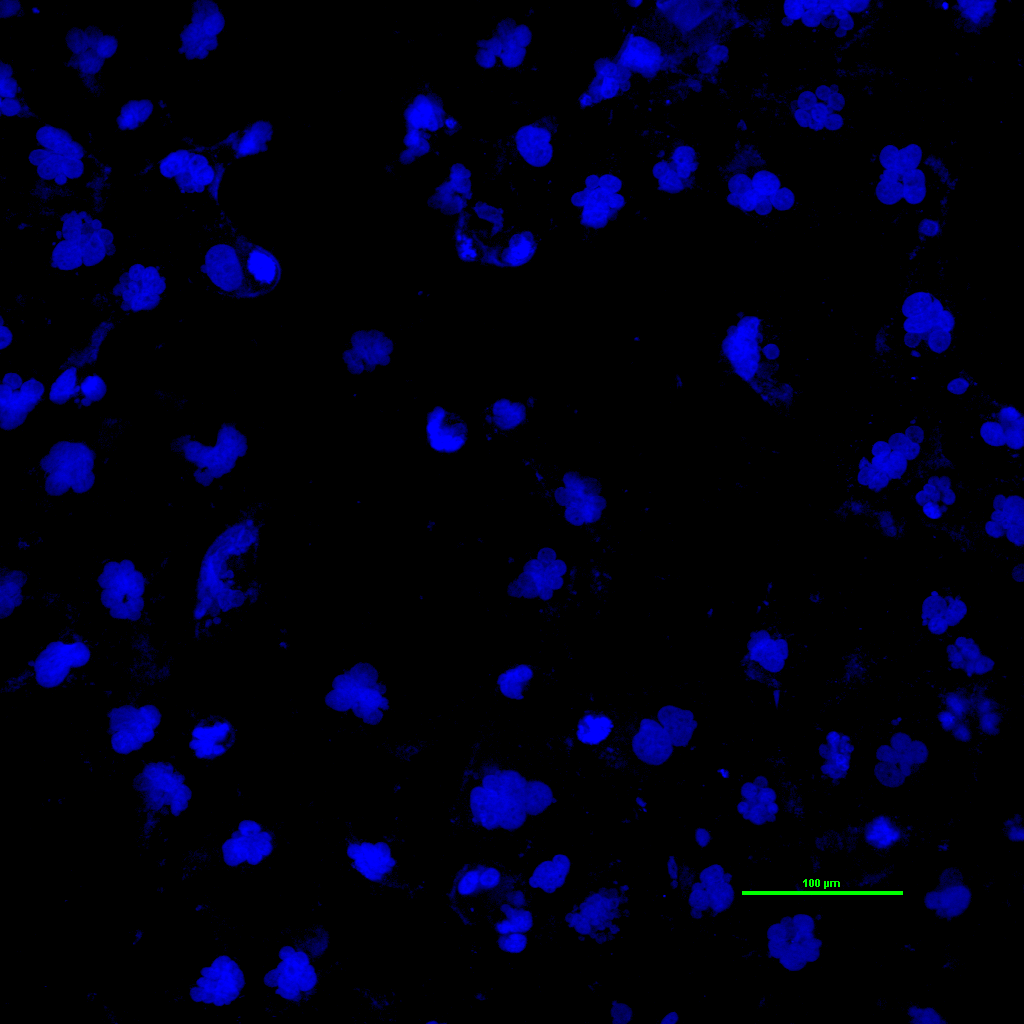

Supplement: Supplementary file 8 [file DataSheet6.ZIP › original data of immunofluorescence assay/7m-200-2_RGB_DAPI.tif]

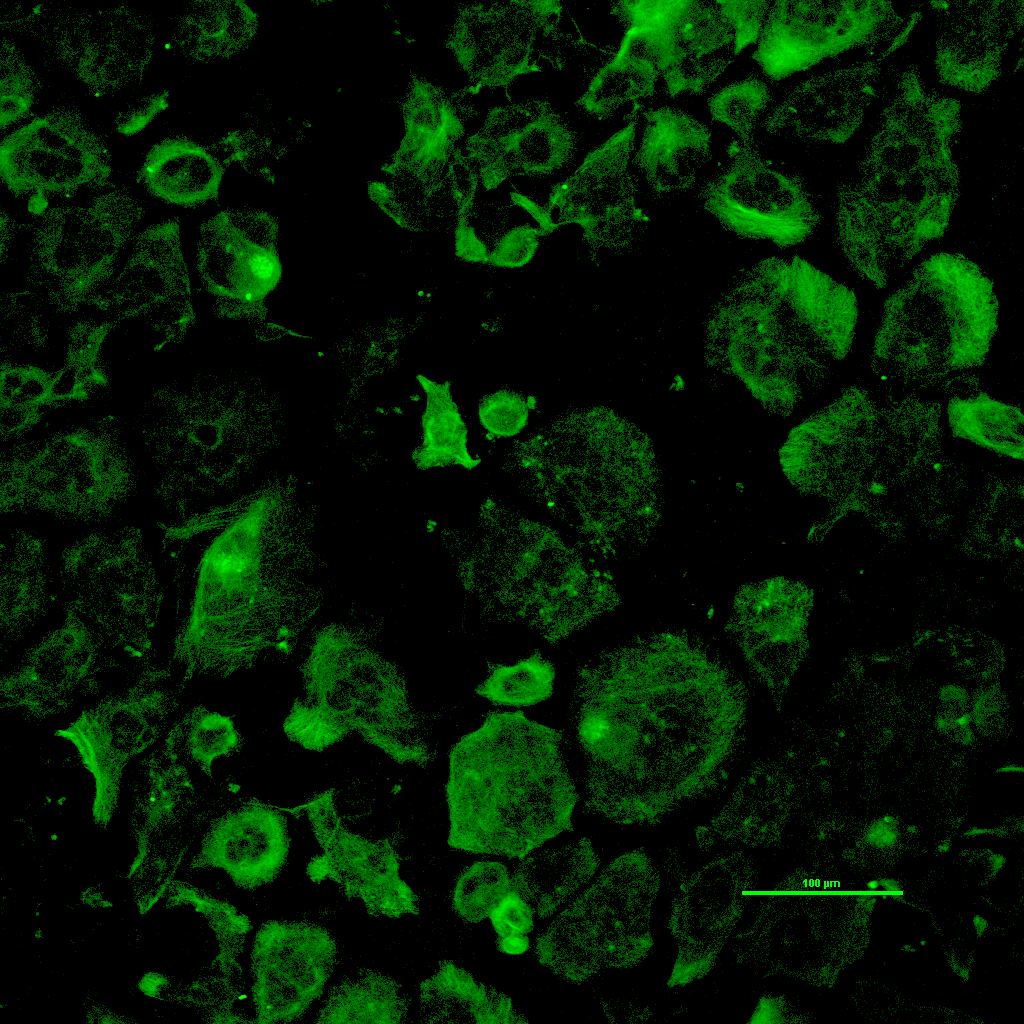

Supplement: Supplementary file 8 [file DataSheet6.ZIP › original data of immunofluorescence assay/7m-200-2_RGB_FITC.tif]

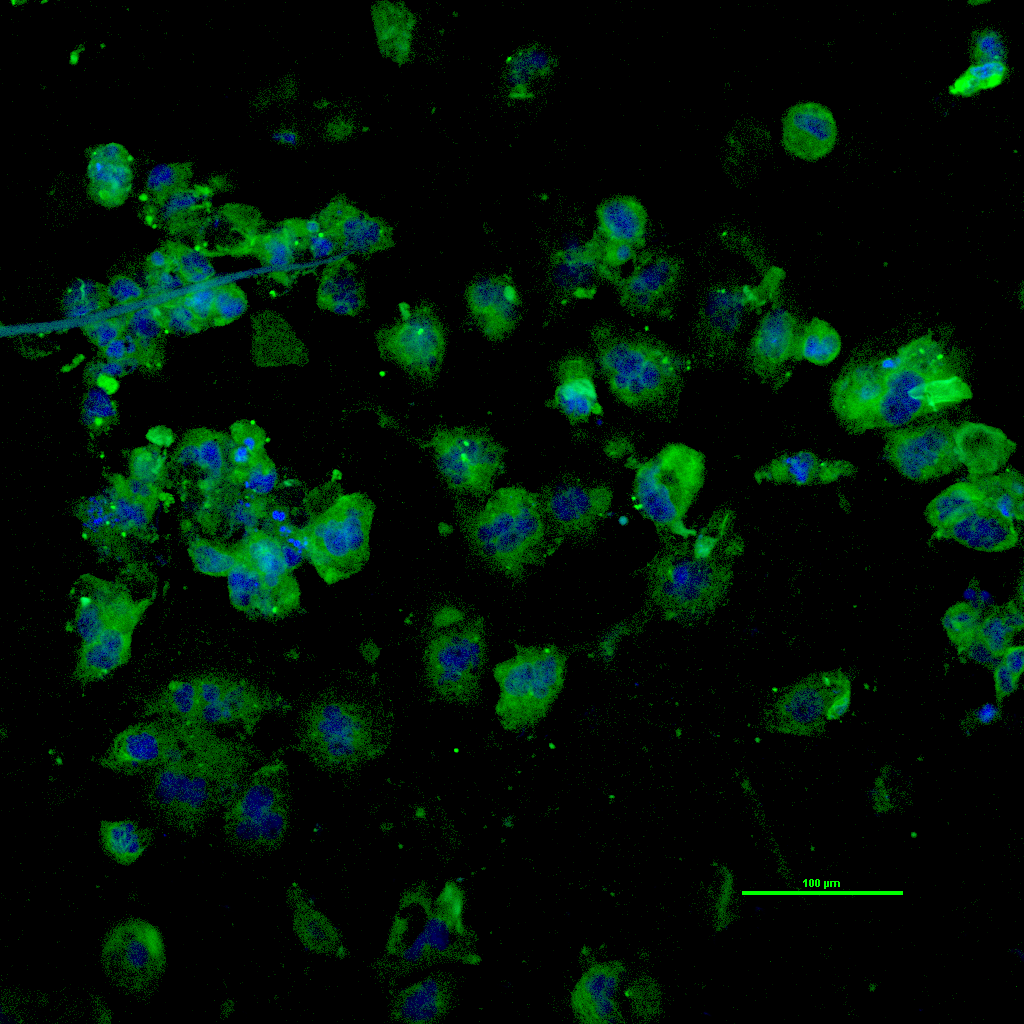

Supplement: Supplementary file 8 [file DataSheet6.ZIP › original data of immunofluorescence assay/7m-400_RGB.tif]

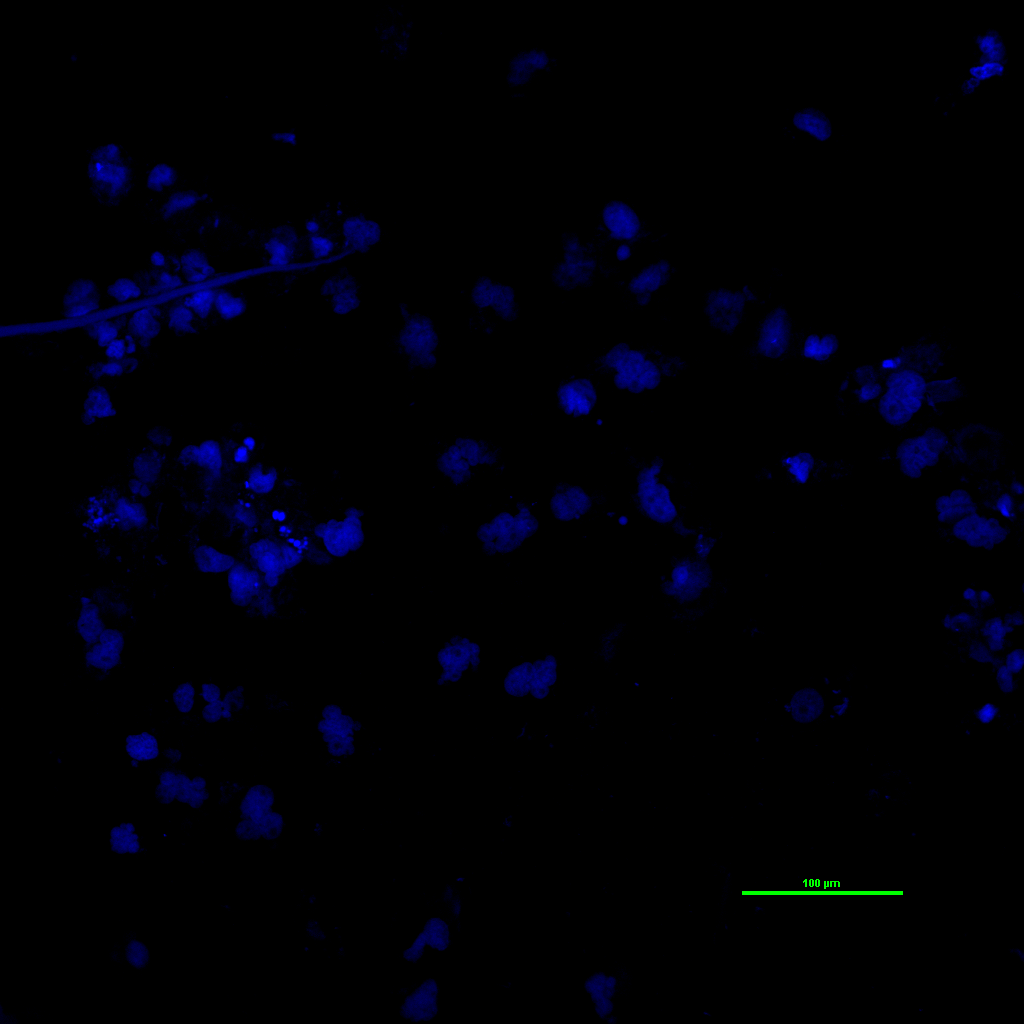

Supplement: Supplementary file 8 [file DataSheet6.ZIP › original data of immunofluorescence assay/7m-400_RGB_DAPI.tif]

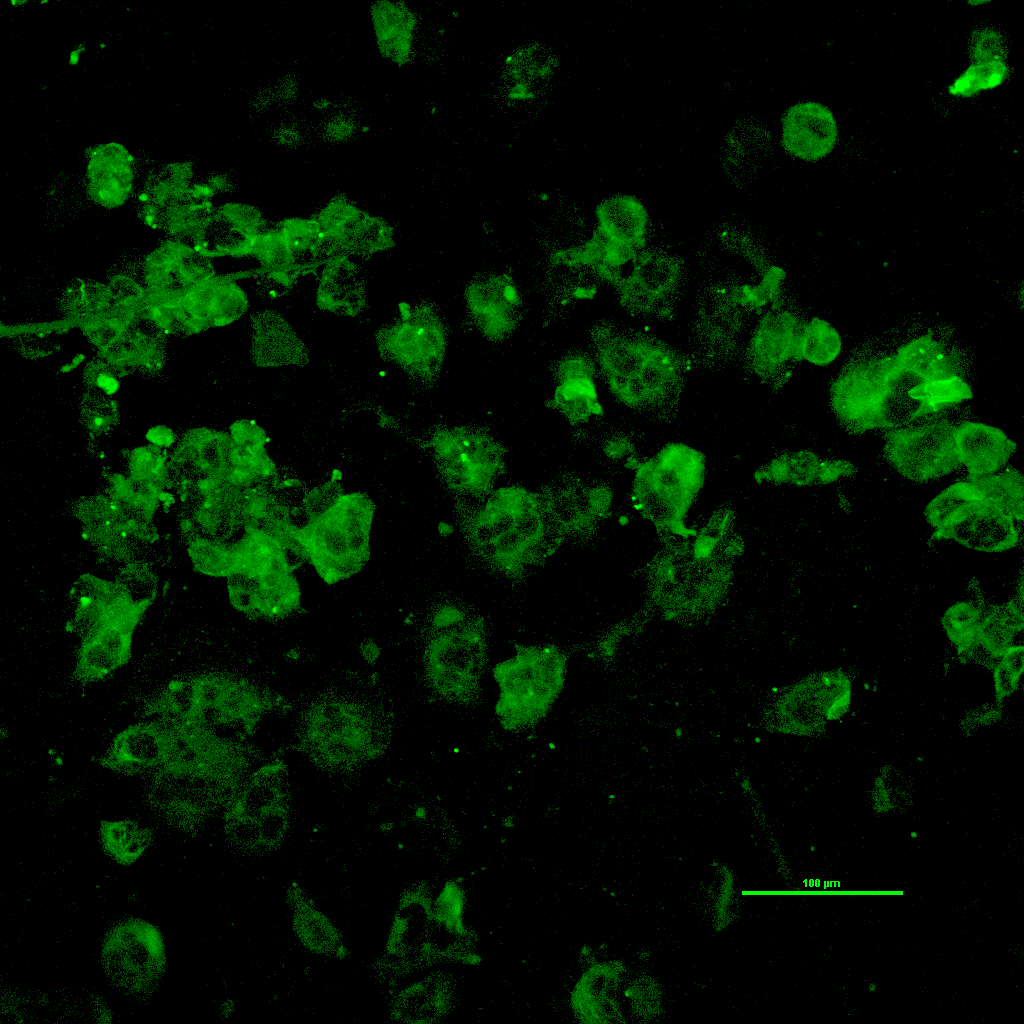

Supplement: Supplementary file 8 [file DataSheet6.ZIP › original data of immunofluorescence assay/7m-400_RGB_FITC.tif]

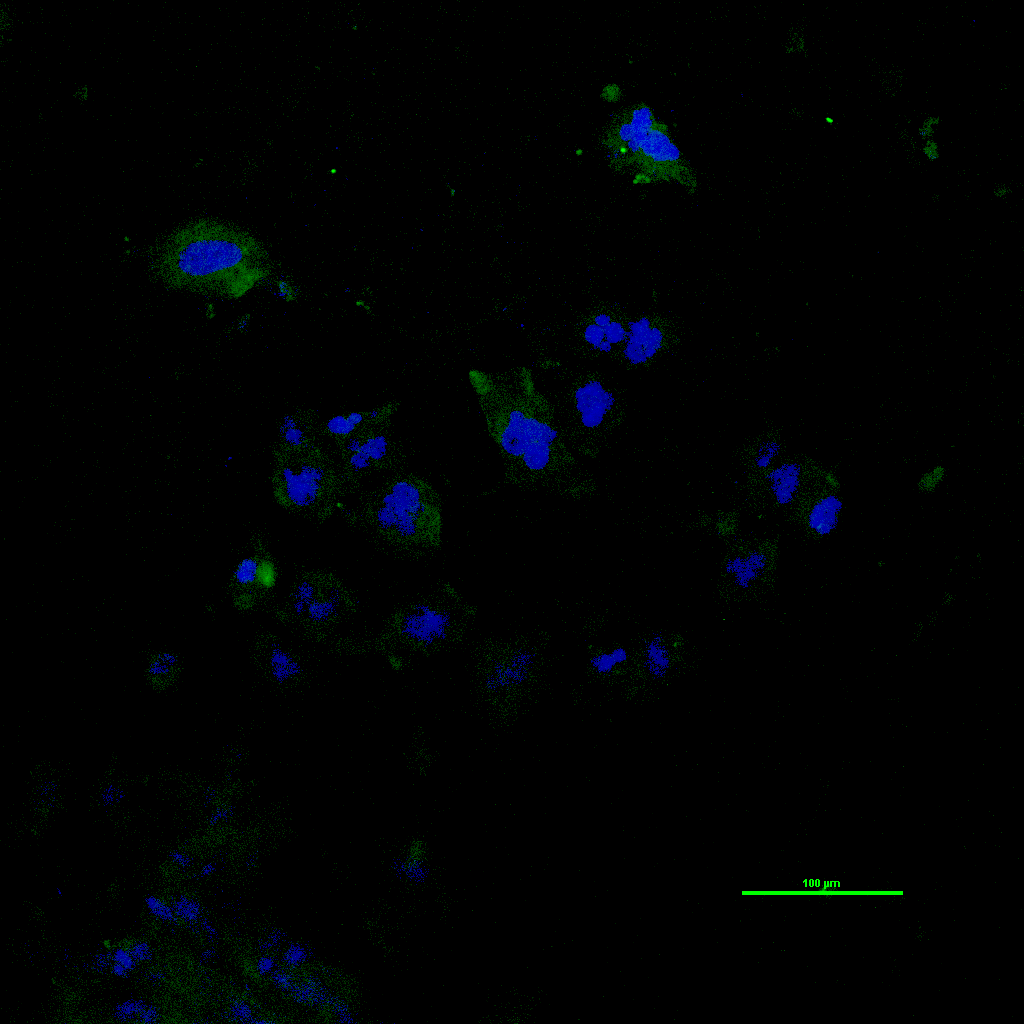

Supplement: Supplementary file 8 [file DataSheet6.ZIP › original data of immunofluorescence assay/7m-400-2_RGB.tif]

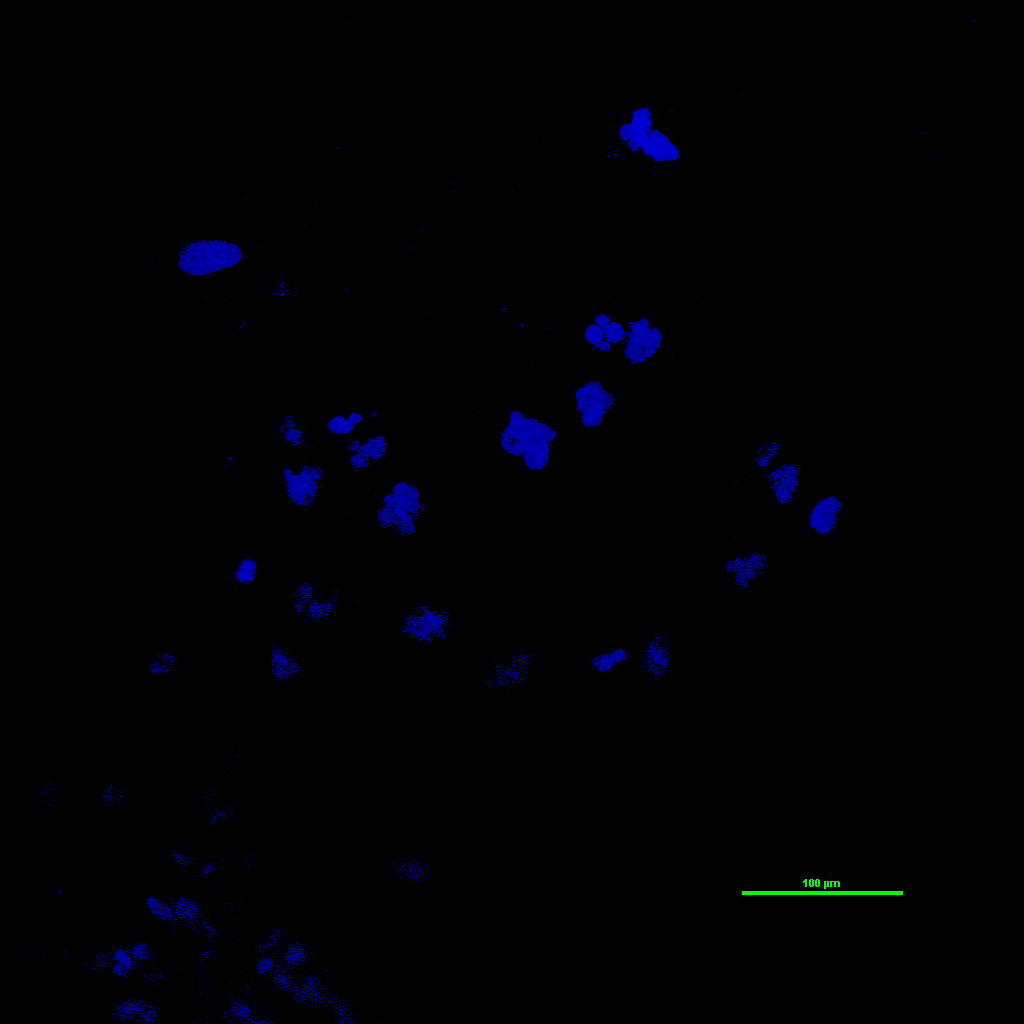

Supplement: Supplementary file 8 [file DataSheet6.ZIP › original data of immunofluorescence assay/7m-400-2_RGB_DAPI.tif]

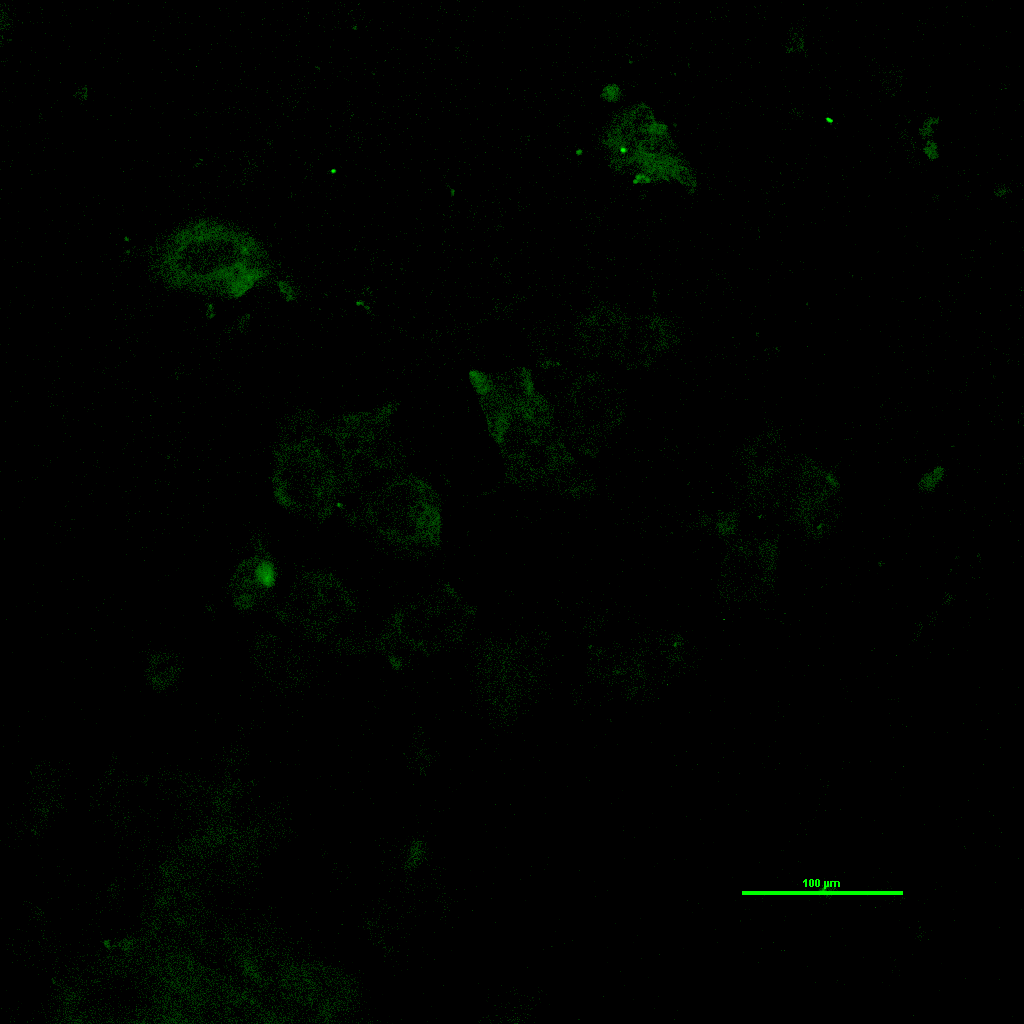

Supplement: Supplementary file 8 [file DataSheet6.ZIP › original data of immunofluorescence assay/7m-400-2_RGB_FITC.tif]

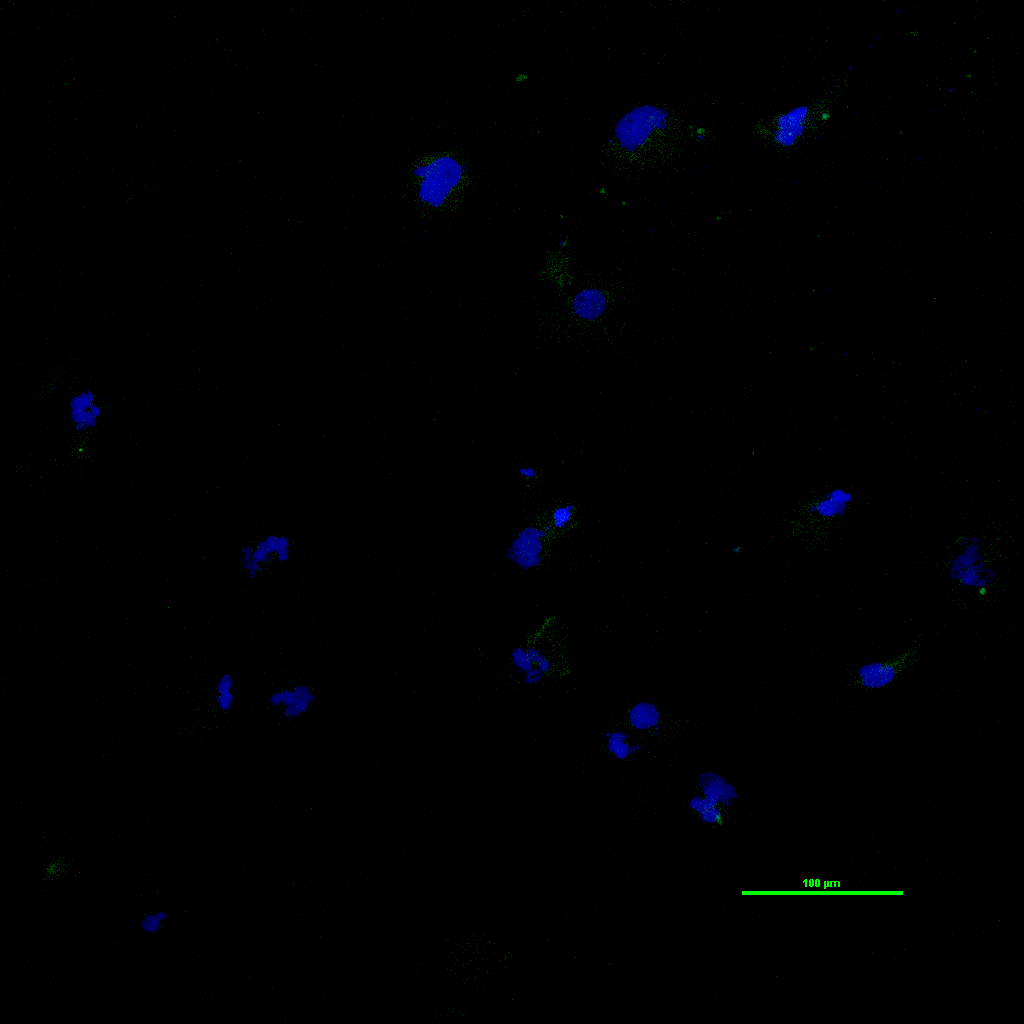

Supplement: Supplementary file 8 [file DataSheet6.ZIP › original data of immunofluorescence assay/7m-400-3_RGB.tif]

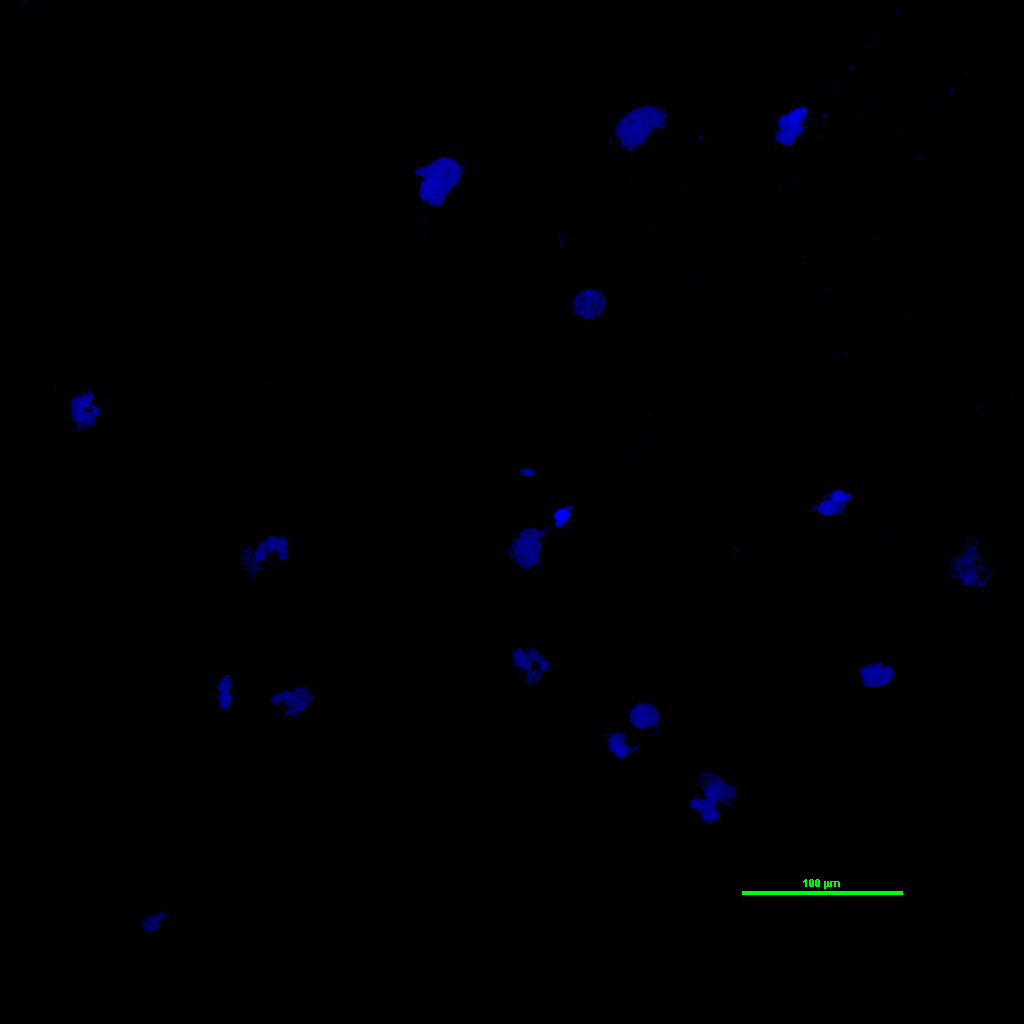

Supplement: Supplementary file 8 [file DataSheet6.ZIP › original data of immunofluorescence assay/7m-400-3_RGB_DAPI.tif]

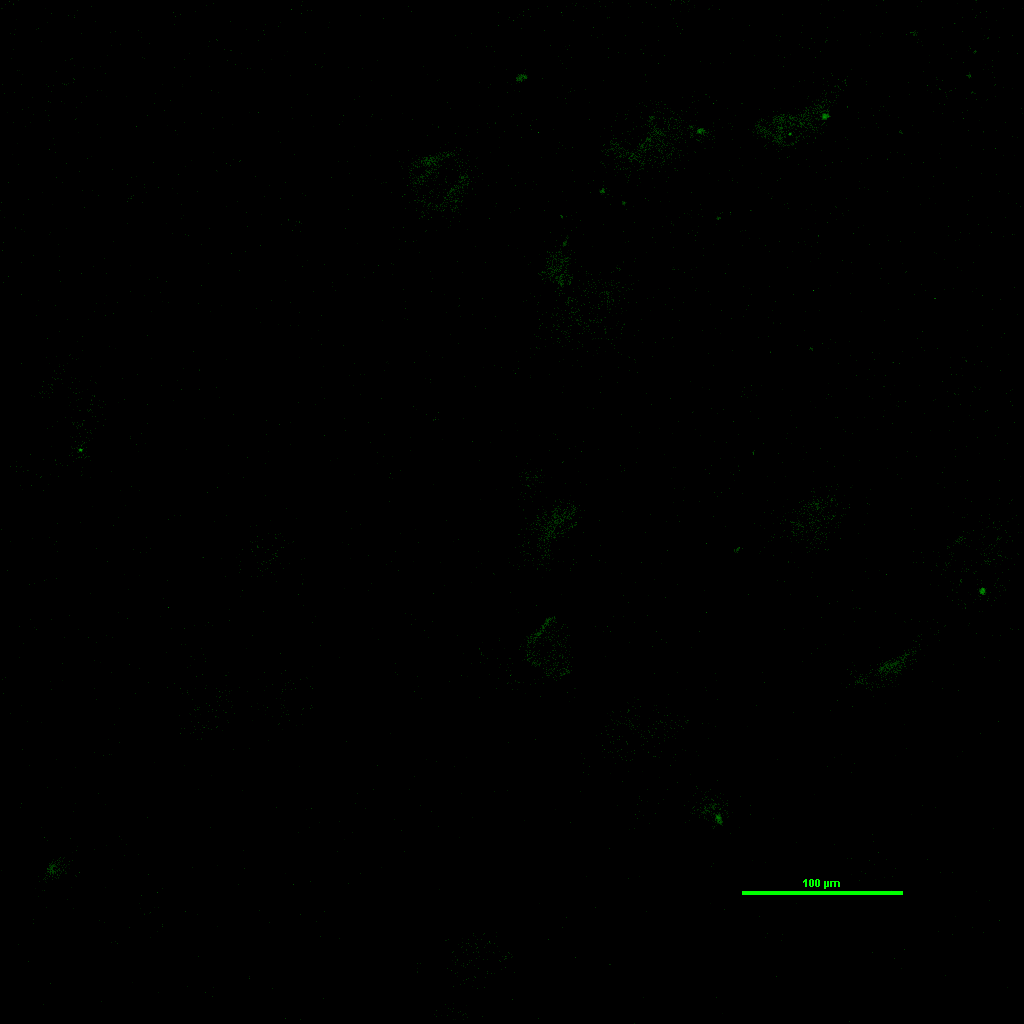

Supplement: Supplementary file 8 [file DataSheet6.ZIP › original data of immunofluorescence assay/7m-400-3_RGB_FITC.tif]

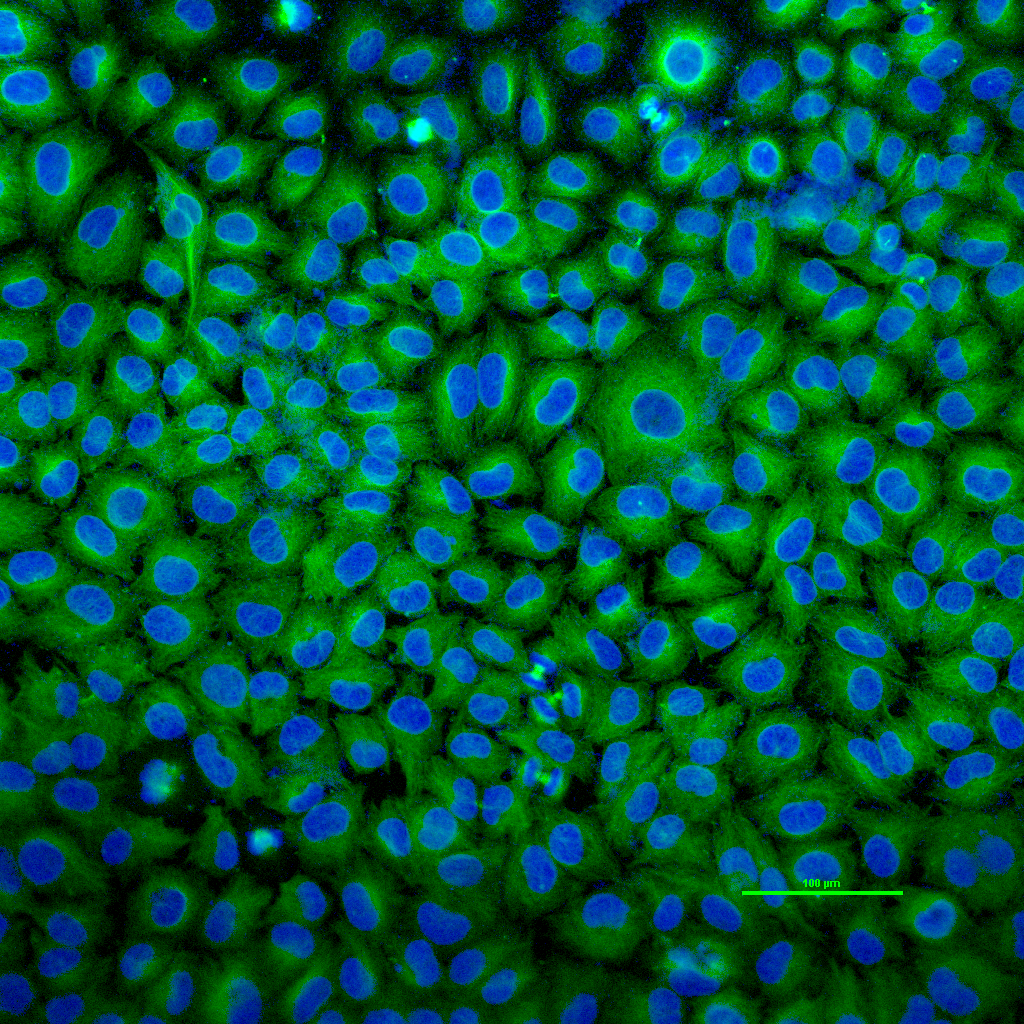

Supplement: Supplementary file 8 [file DataSheet6.ZIP › original data of immunofluorescence assay/MCF-929_RGB.tif]

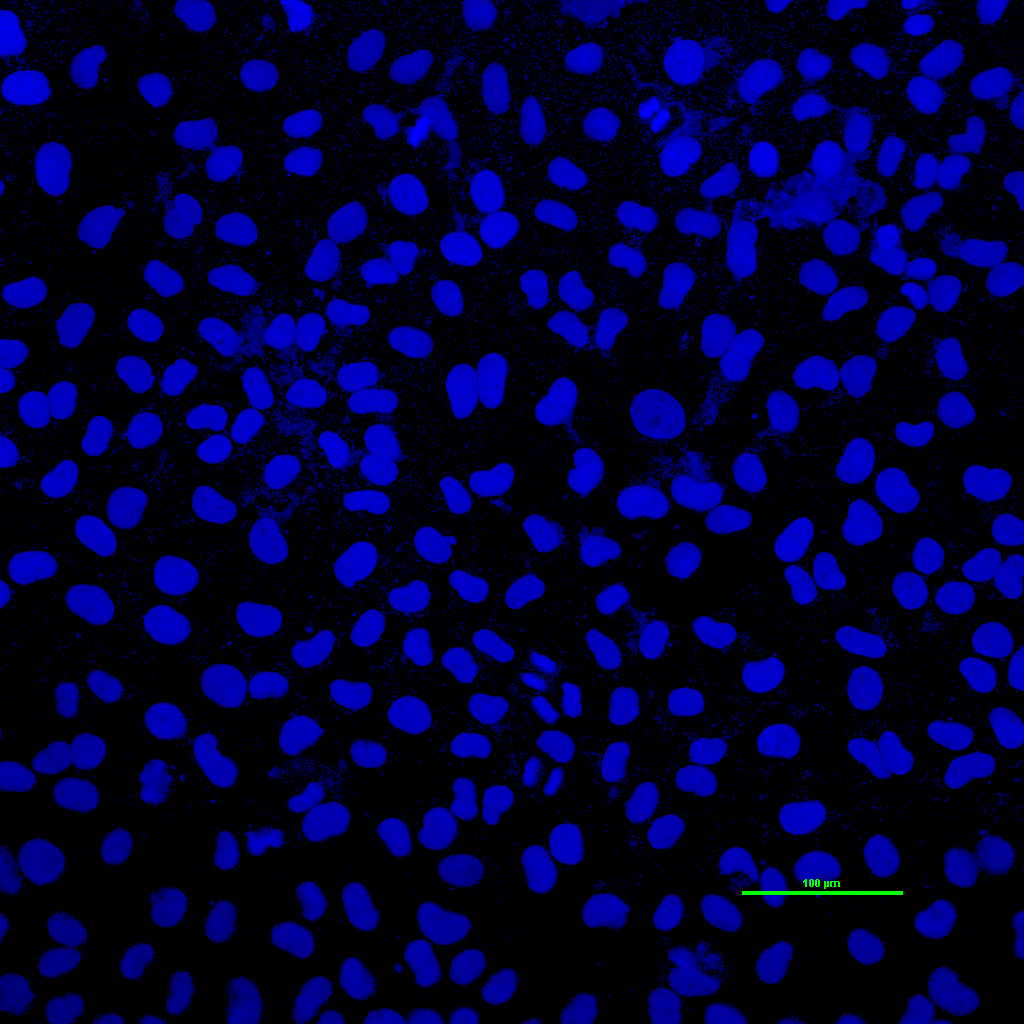

Supplement: Supplementary file 8 [file DataSheet6.ZIP › original data of immunofluorescence assay/MCF-929_RGB_DAPI.tif]

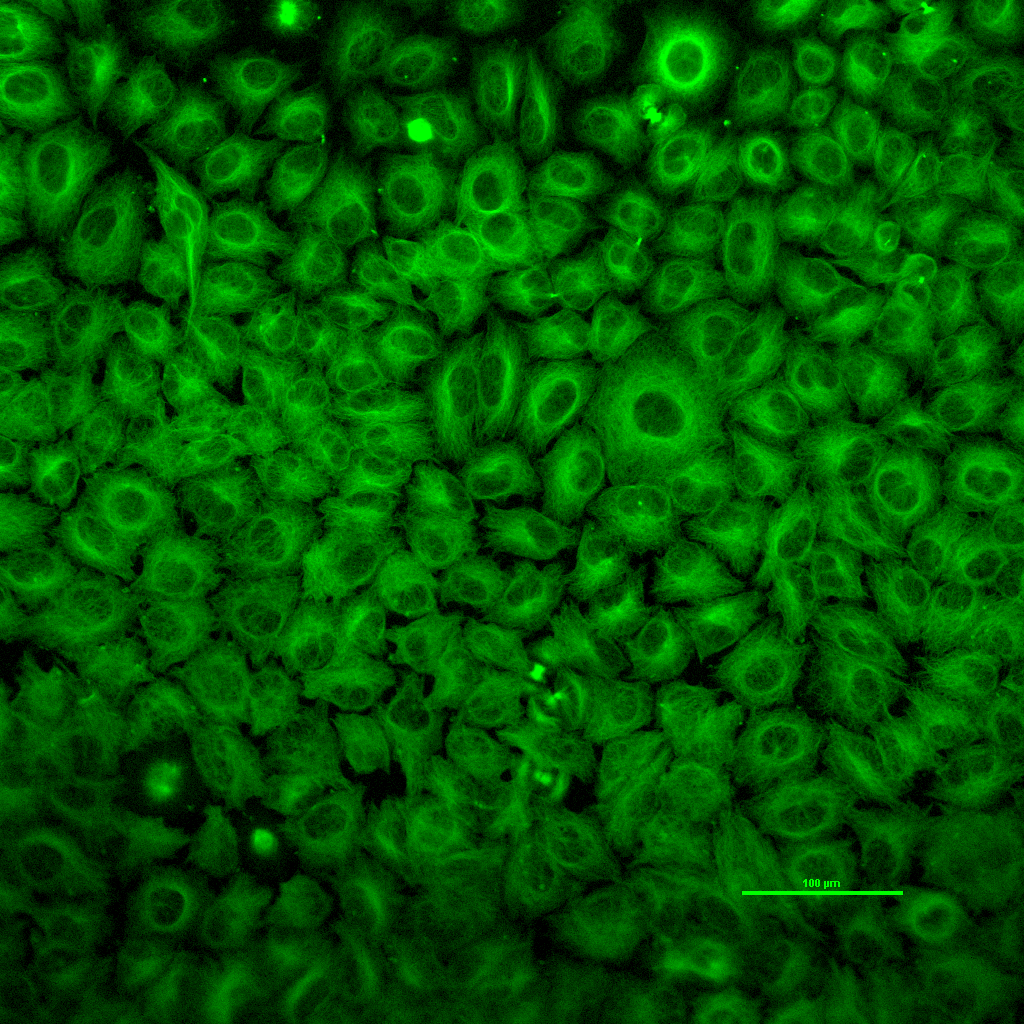

Supplement: Supplementary file 8 [file DataSheet6.ZIP › original data of immunofluorescence assay/MCF-929_RGB_FITC.tif]

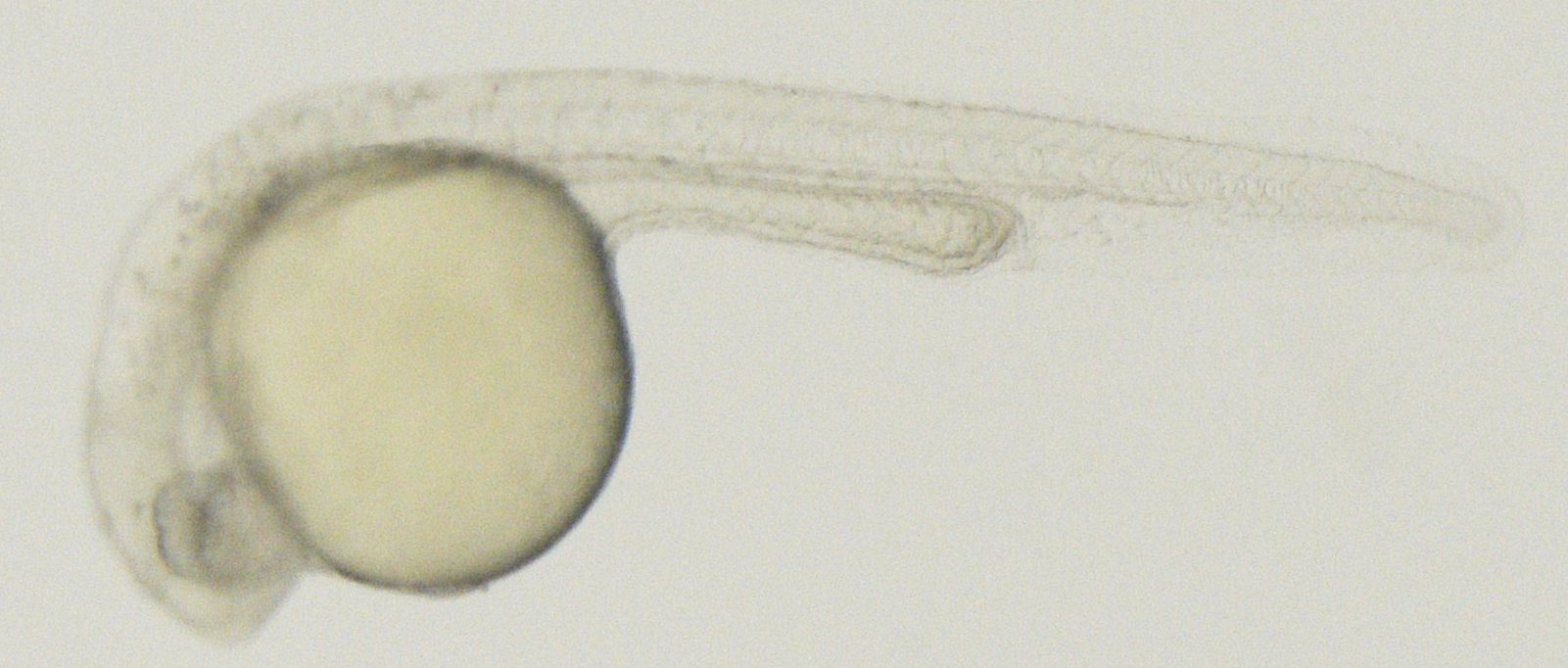

Supplement: Supplementary file 10 [file DataSheet2.ZIP › original data of anti-angiogenic effect on zebrafish embryos/7m-0.5a╠m-1.tif]

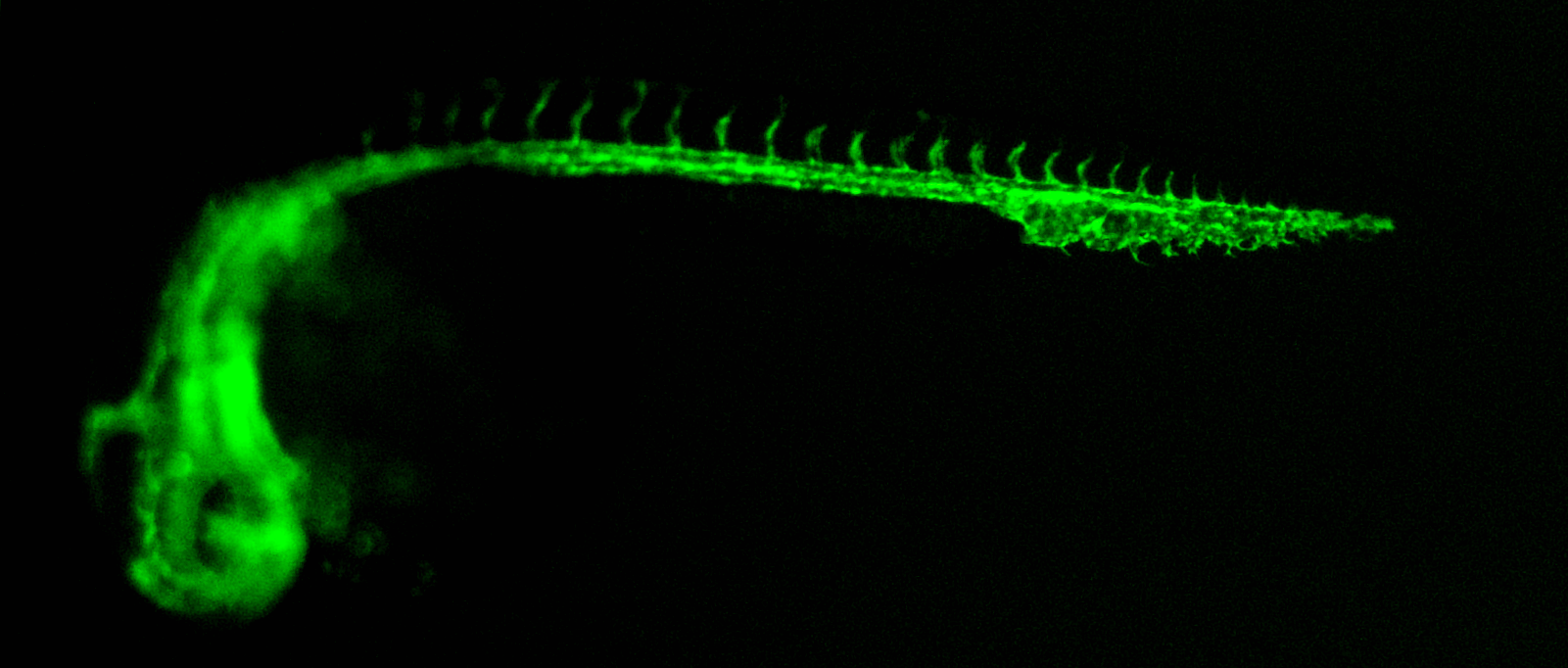

Supplement: Supplementary file 10 [file DataSheet2.ZIP › original data of anti-angiogenic effect on zebrafish embryos/7m-0.5a╠m-2.tif]

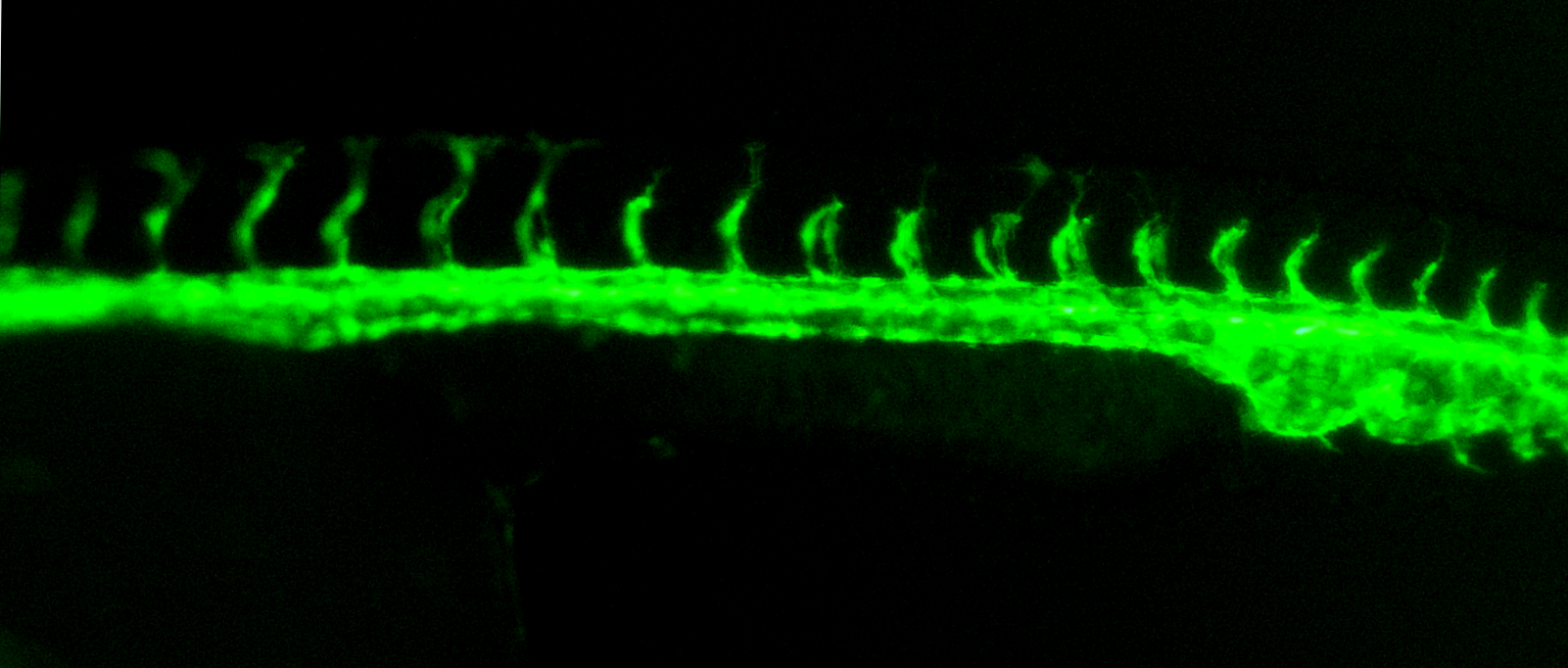

Supplement: Supplementary file 10 [file DataSheet2.ZIP › original data of anti-angiogenic effect on zebrafish embryos/7m-0.5a╠m-3.tif]

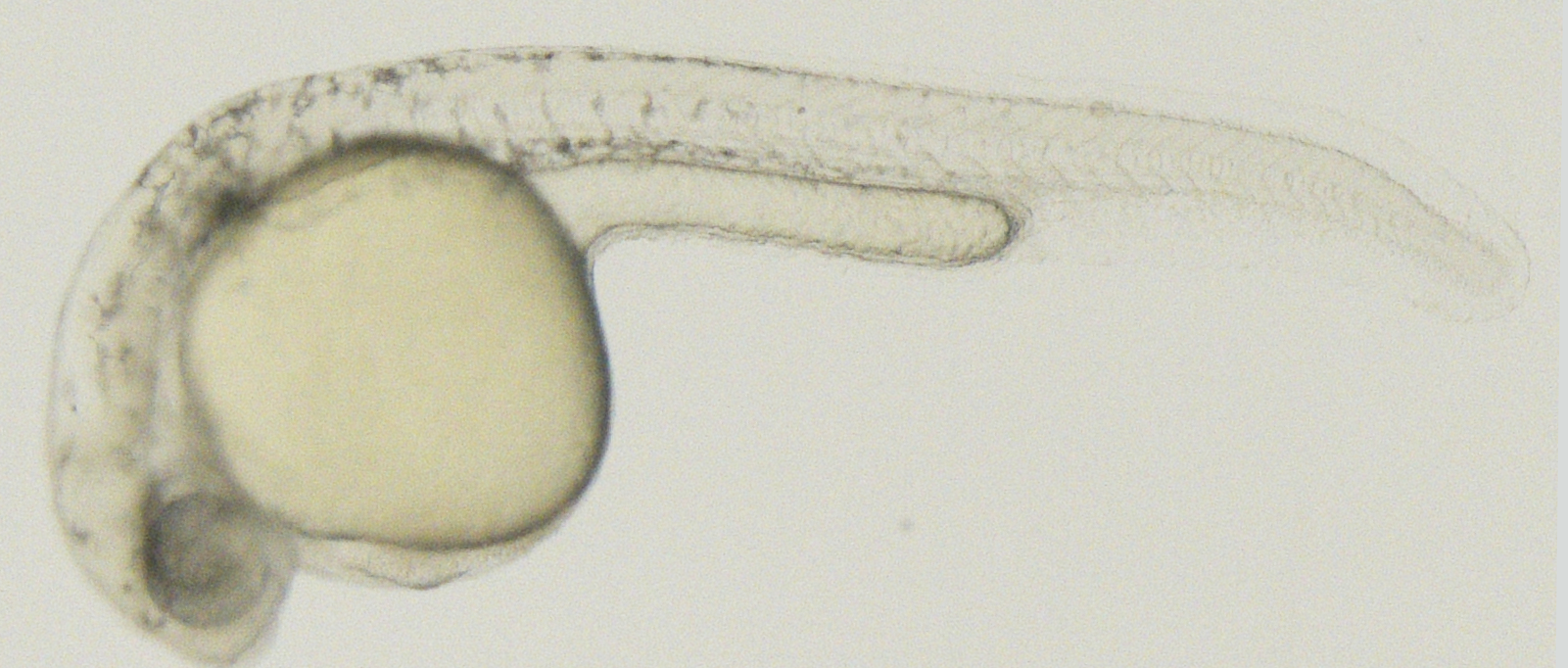

Supplement: Supplementary file 10 [file DataSheet2.ZIP › original data of anti-angiogenic effect on zebrafish embryos/7m-1a╠m-1.tif]

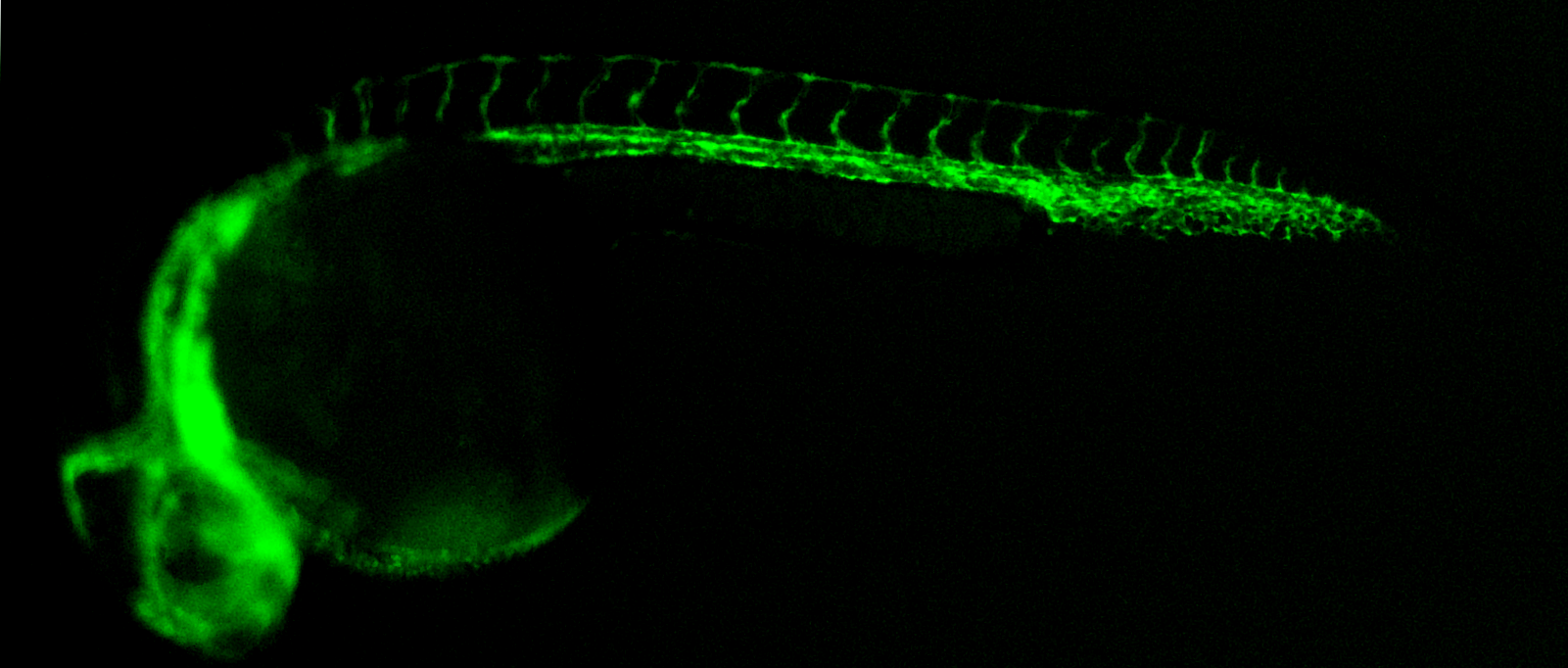

Supplement: Supplementary file 10 [file DataSheet2.ZIP › original data of anti-angiogenic effect on zebrafish embryos/7m-1a╠m-2.tif]

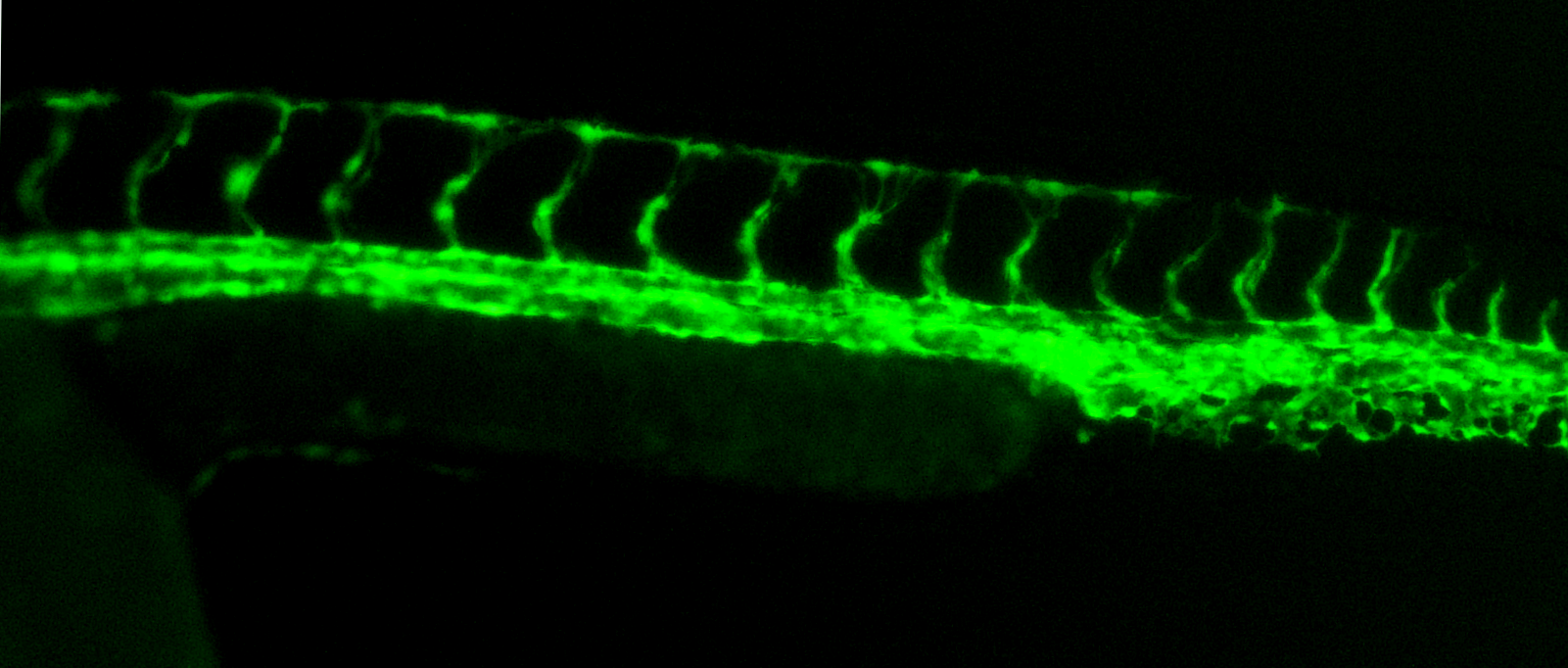

Supplement: Supplementary file 10 [file DataSheet2.ZIP › original data of anti-angiogenic effect on zebrafish embryos/7m-1a╠m-3.tif]

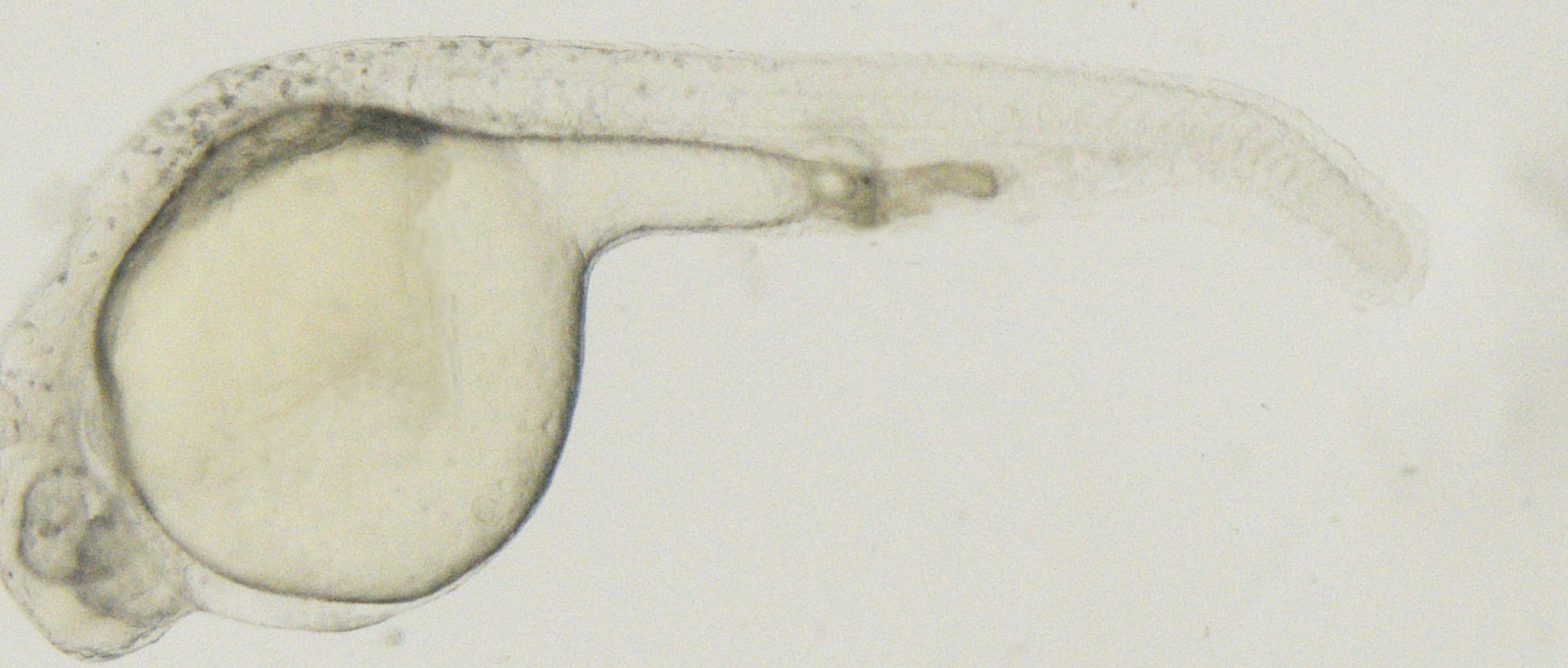

Supplement: Supplementary file 10 [file DataSheet2.ZIP › original data of anti-angiogenic effect on zebrafish embryos/7m-2.5a╠m-1.tif]

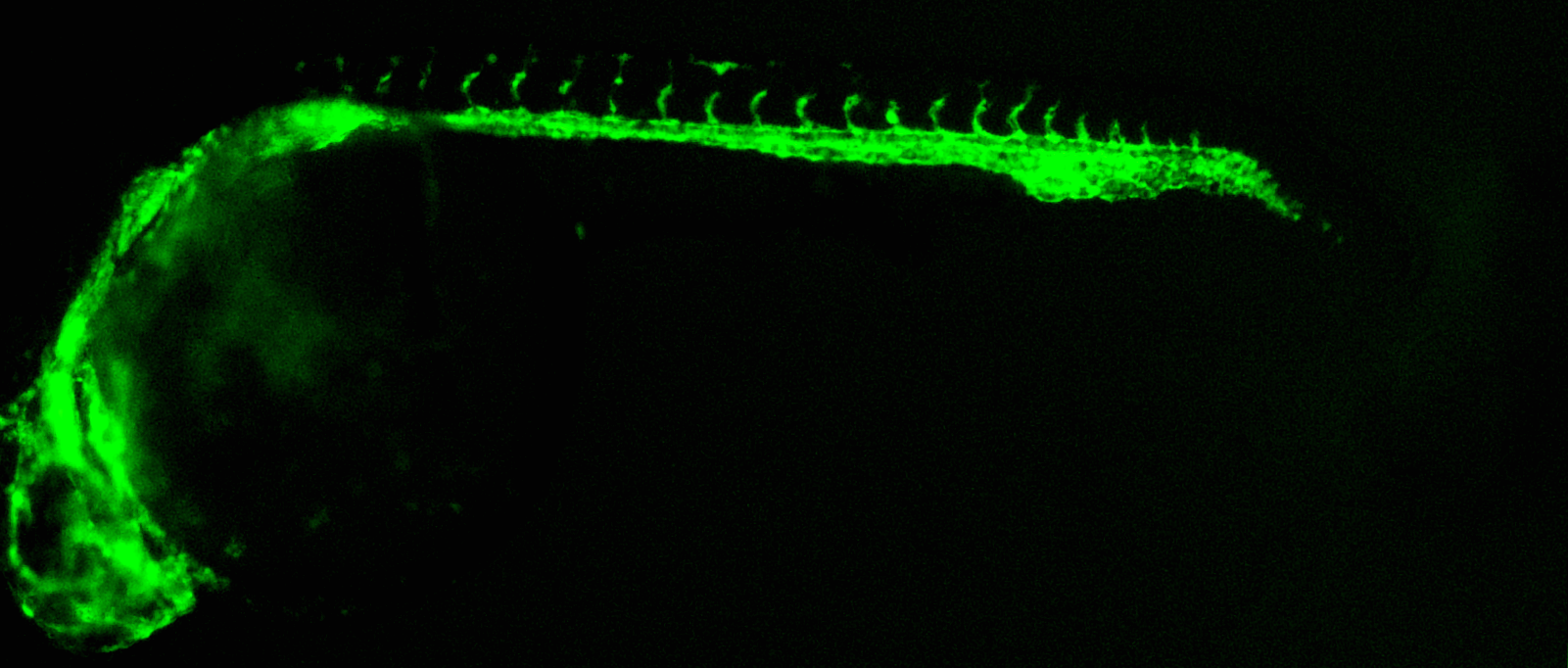

Supplement: Supplementary file 10 [file DataSheet2.ZIP › original data of anti-angiogenic effect on zebrafish embryos/7m-2.5a╠m-2.tif]

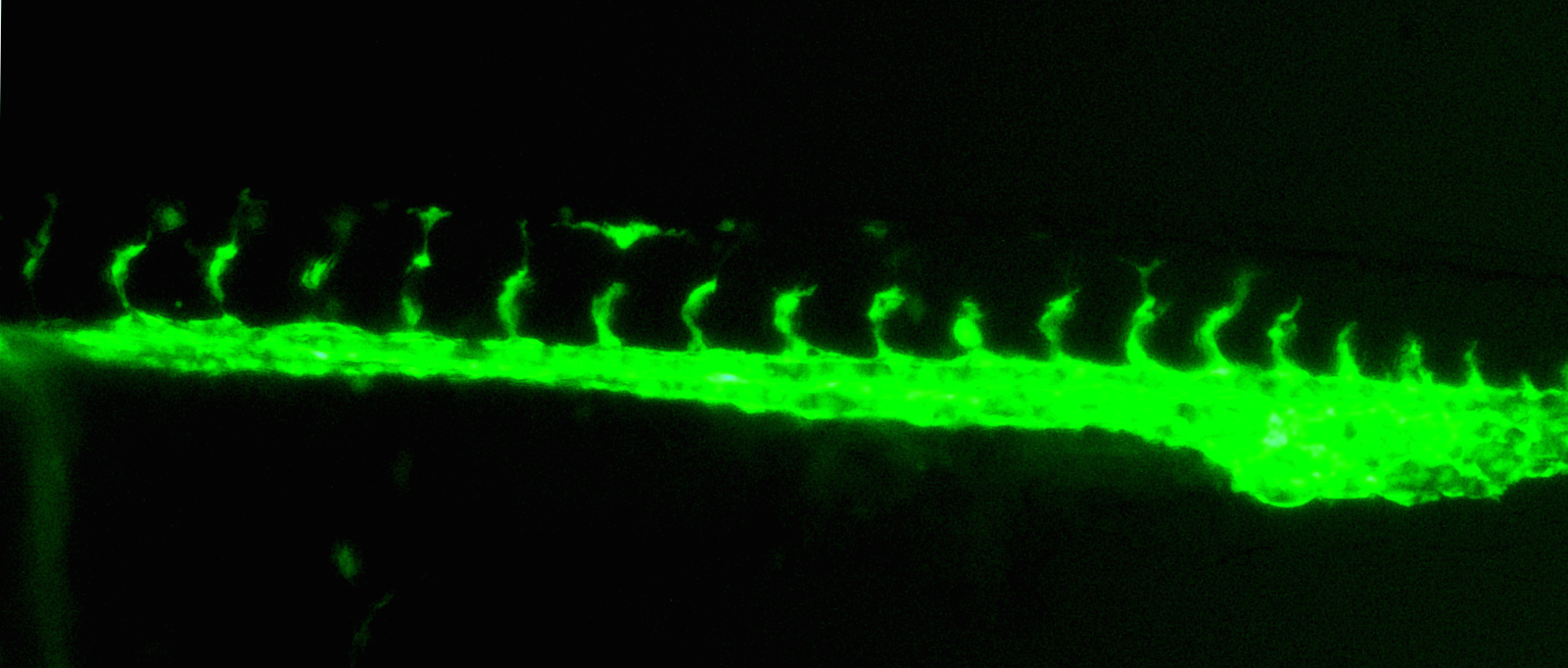

Supplement: Supplementary file 10 [file DataSheet2.ZIP › original data of anti-angiogenic effect on zebrafish embryos/7m-2.5a╠m-3.tif]

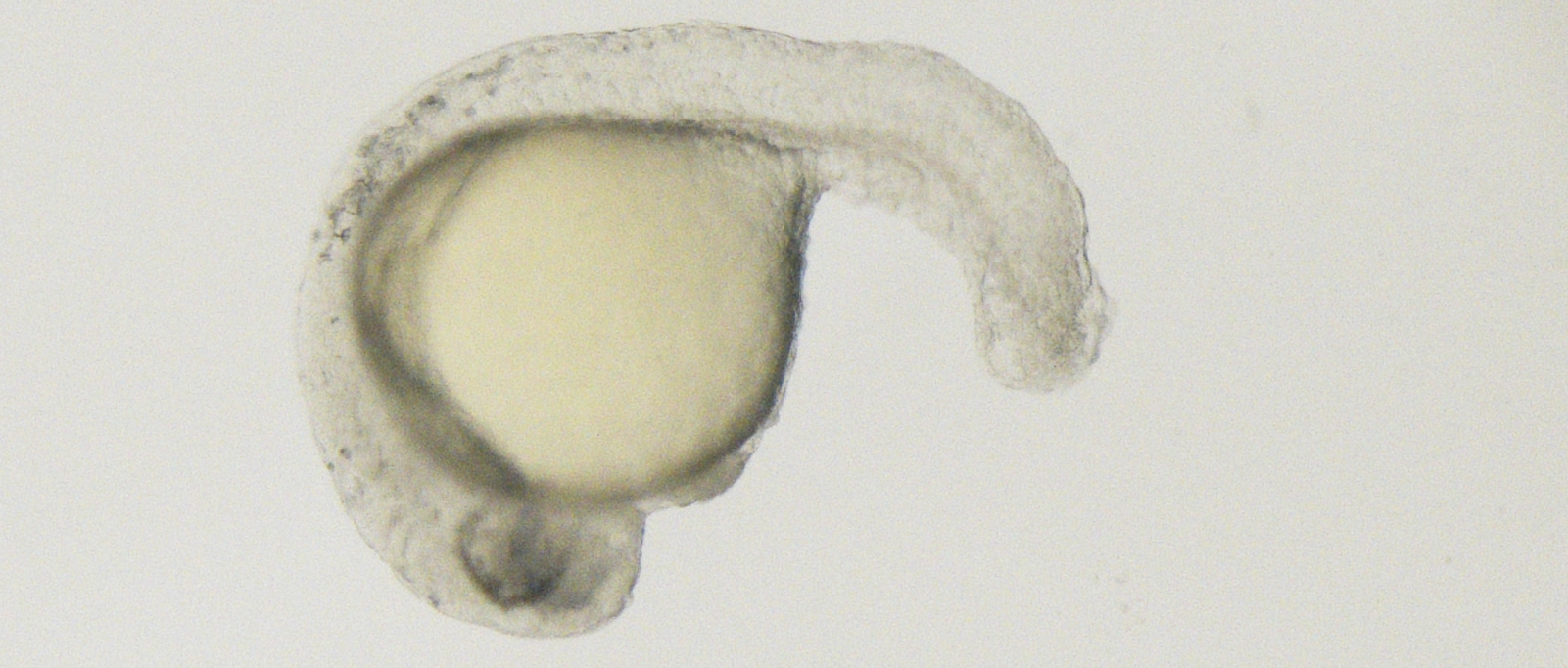

Supplement: Supplementary file 10 [file DataSheet2.ZIP › original data of anti-angiogenic effect on zebrafish embryos/7m-5a╠m-1.tif]

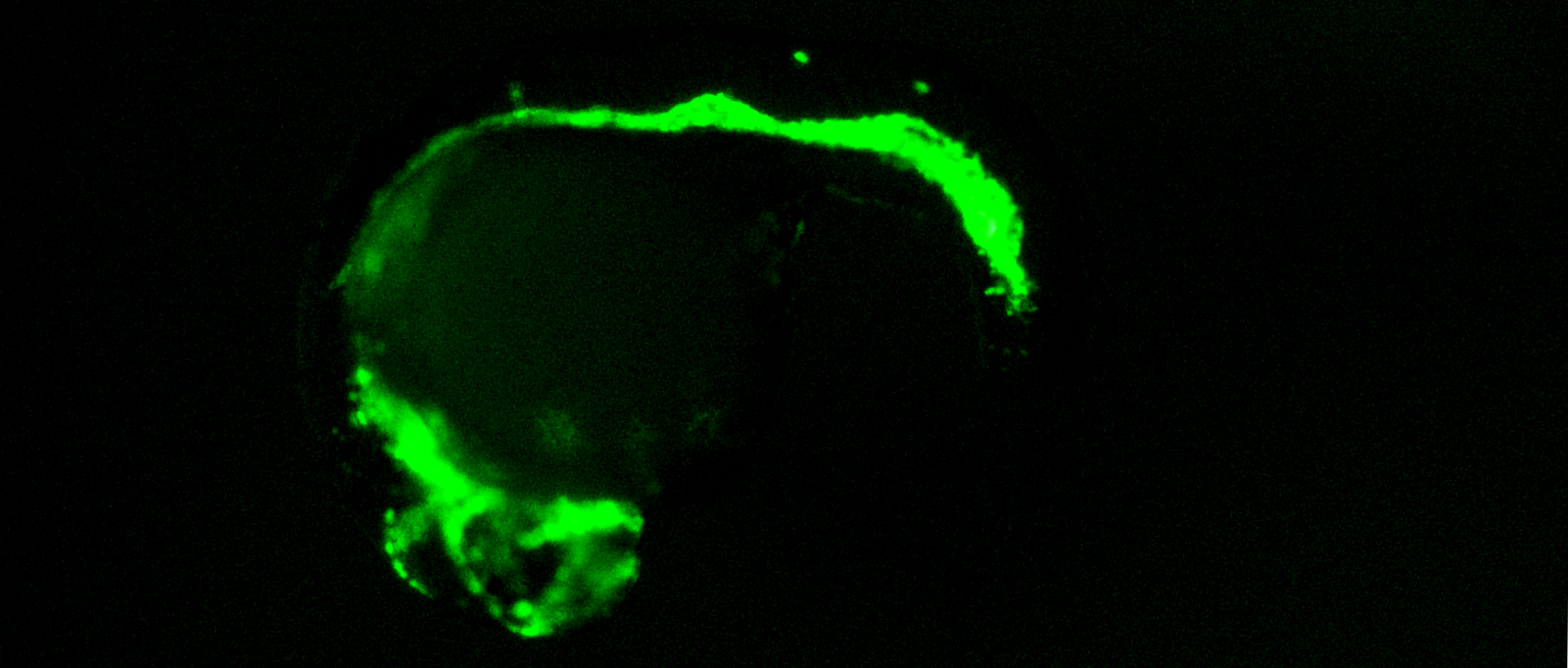

Supplement: Supplementary file 10 [file DataSheet2.ZIP › original data of anti-angiogenic effect on zebrafish embryos/7m-5a╠m-2.tif]

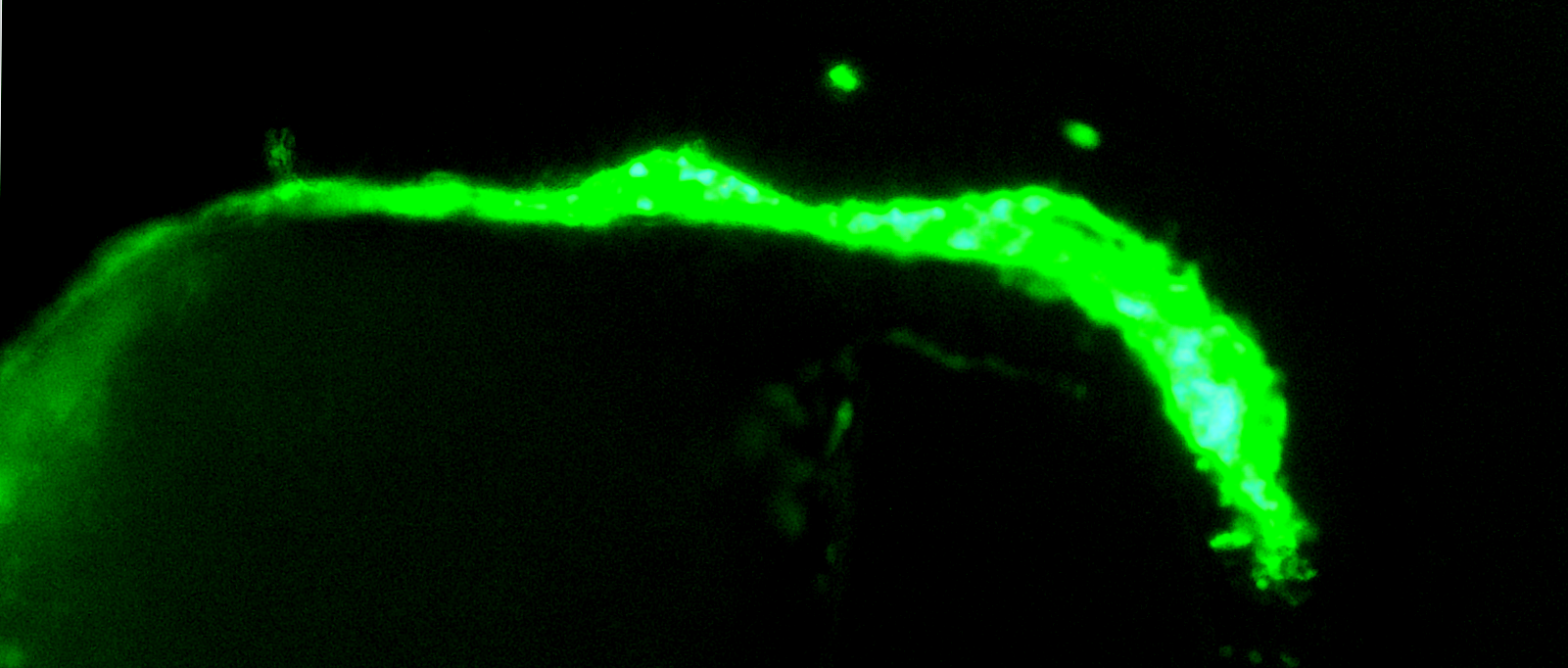

Supplement: Supplementary file 10 [file DataSheet2.ZIP › original data of anti-angiogenic effect on zebrafish embryos/7m-5a╠m-3.tif]

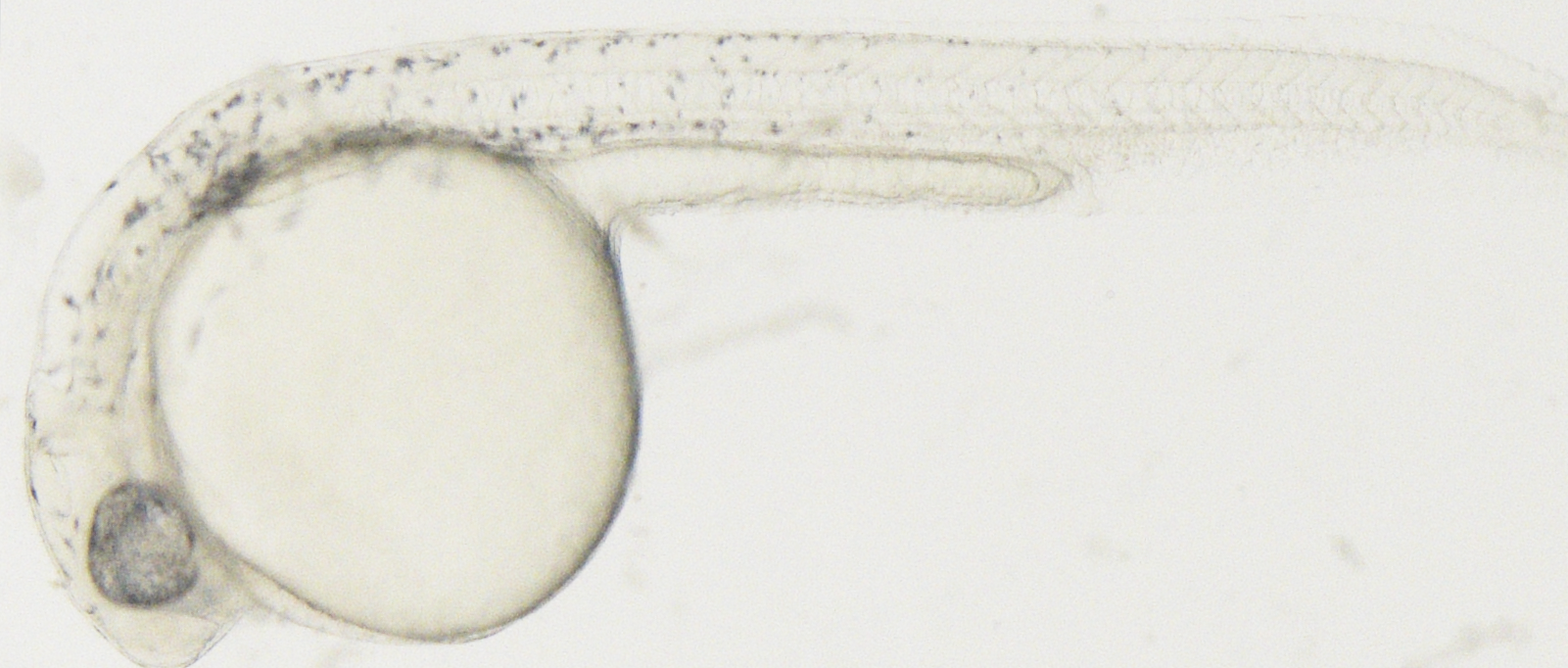

Supplement: Supplementary file 10 [file DataSheet2.ZIP › original data of anti-angiogenic effect on zebrafish embryos/NC-1.tif]

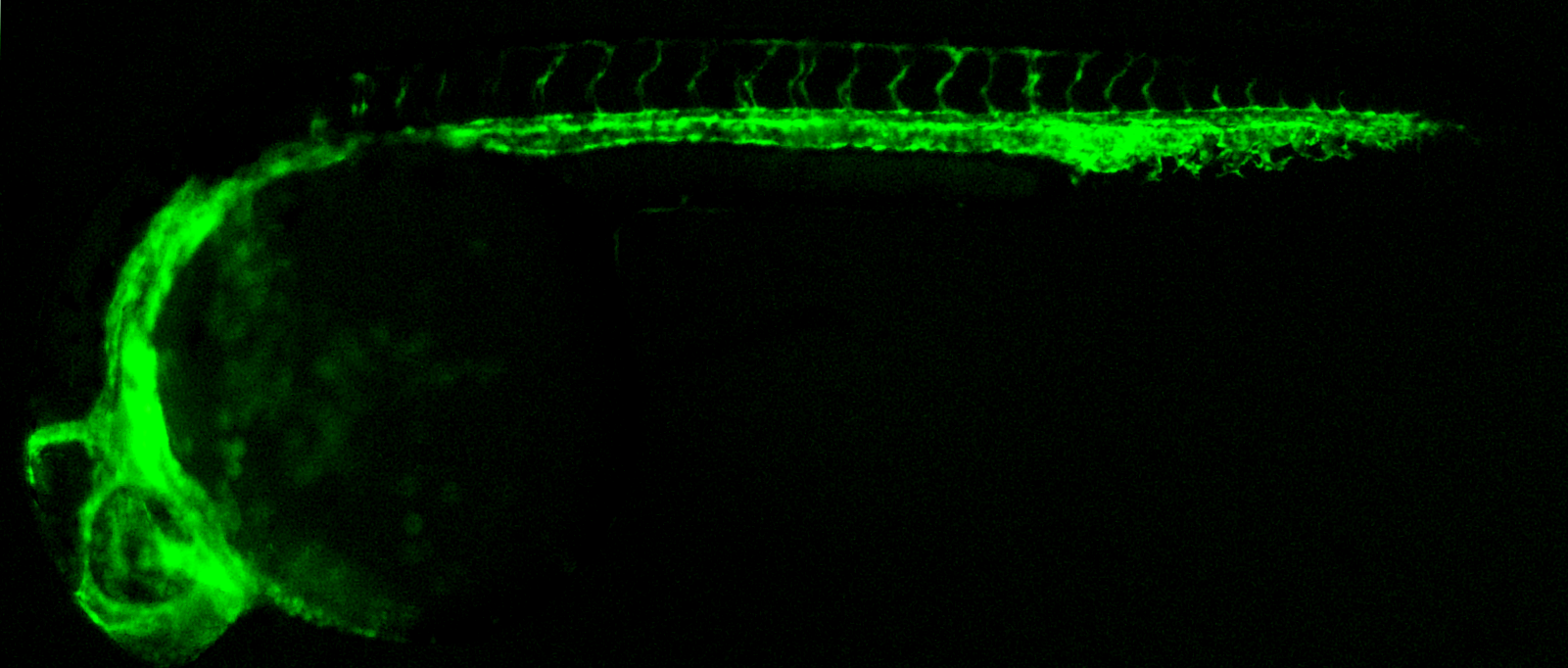

Supplement: Supplementary file 10 [file DataSheet2.ZIP › original data of anti-angiogenic effect on zebrafish embryos/NC-2.tif]

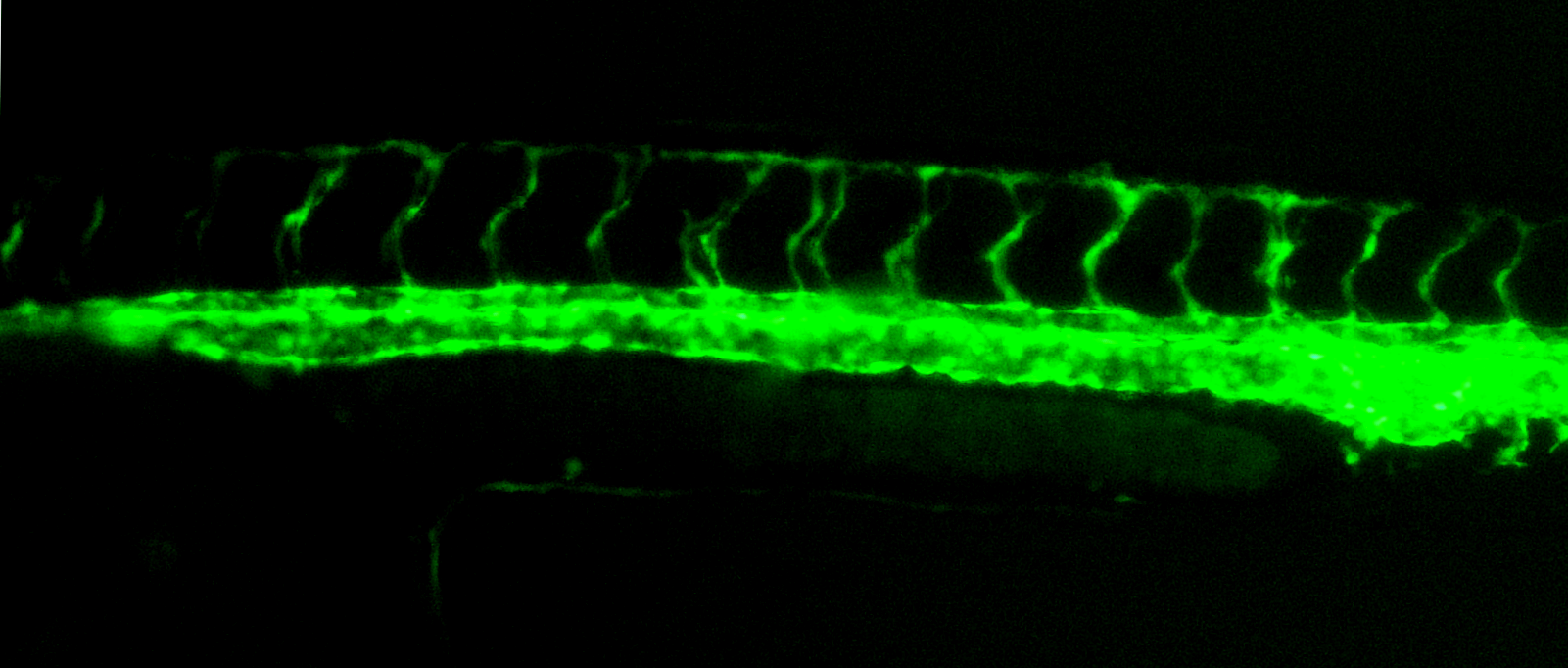

Supplement: Supplementary file 10 [file DataSheet2.ZIP › original data of anti-angiogenic effect on zebrafish embryos/NC-3.tif]

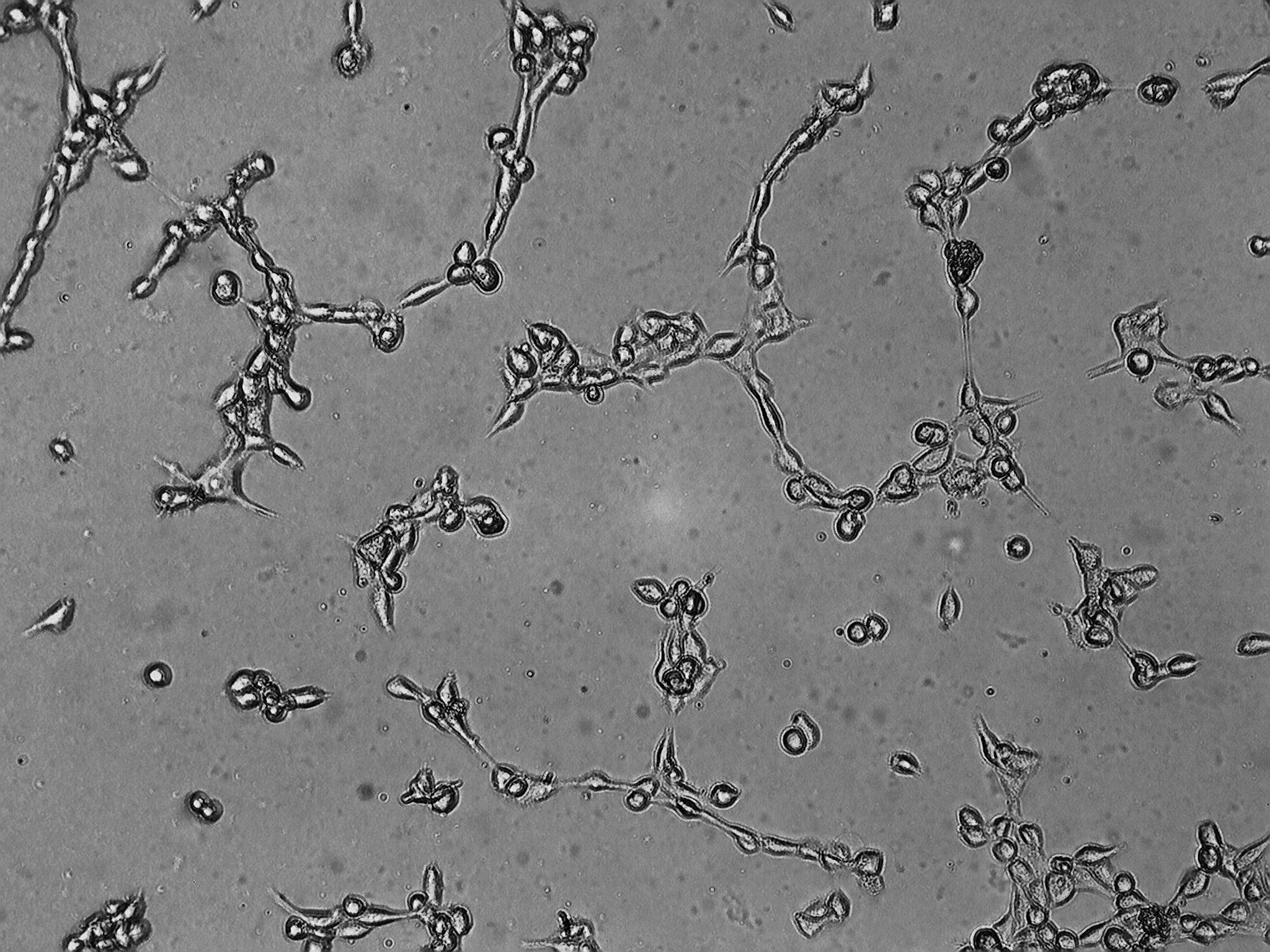

Supplement: Supplementary file 11 [file DataSheet5.ZIP › original data of HUVECs formation/7m-1a╠m.jpg]

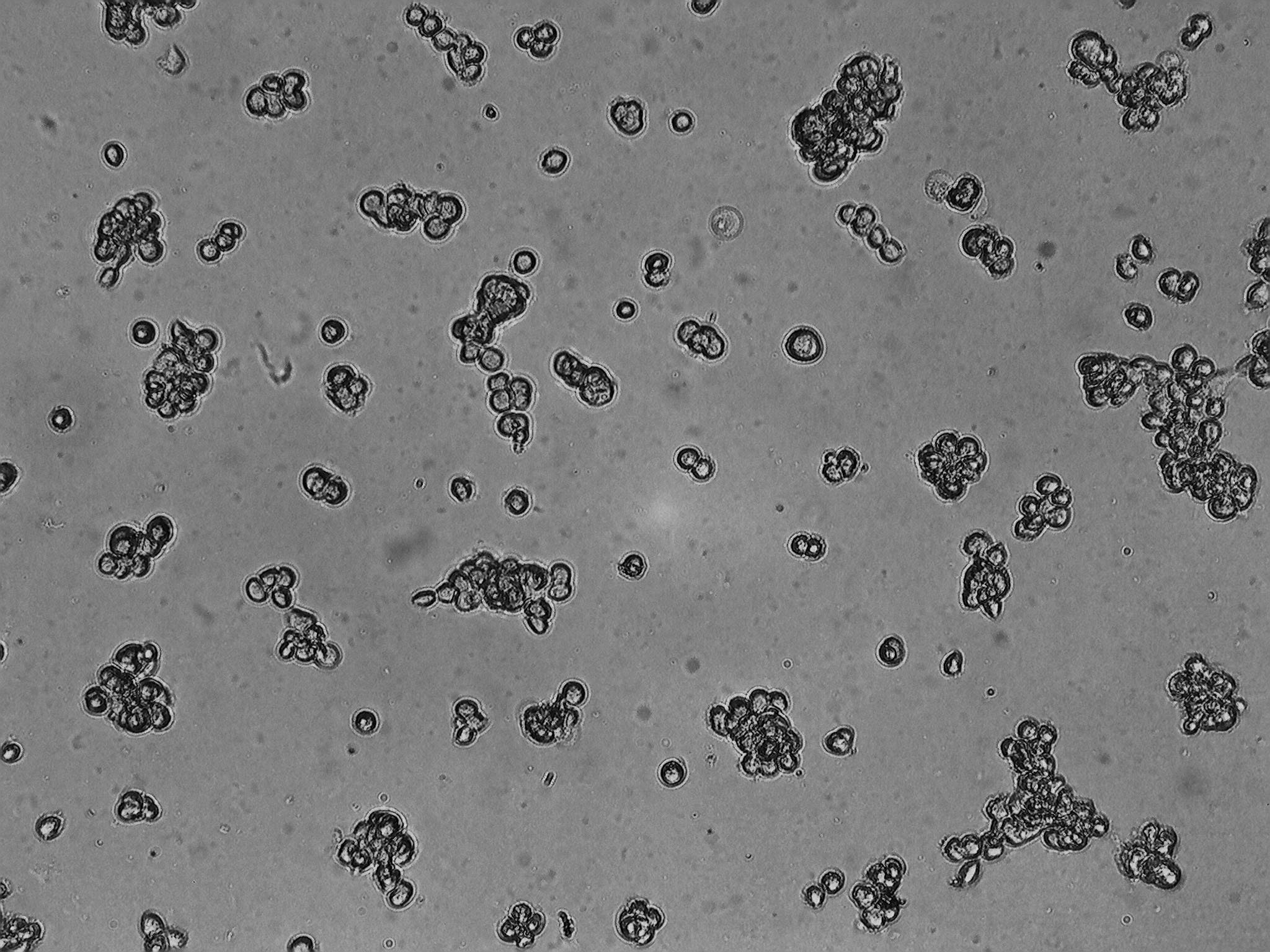

Supplement: Supplementary file 11 [file DataSheet5.ZIP › original data of HUVECs formation/7m-25a╠m.jpg]

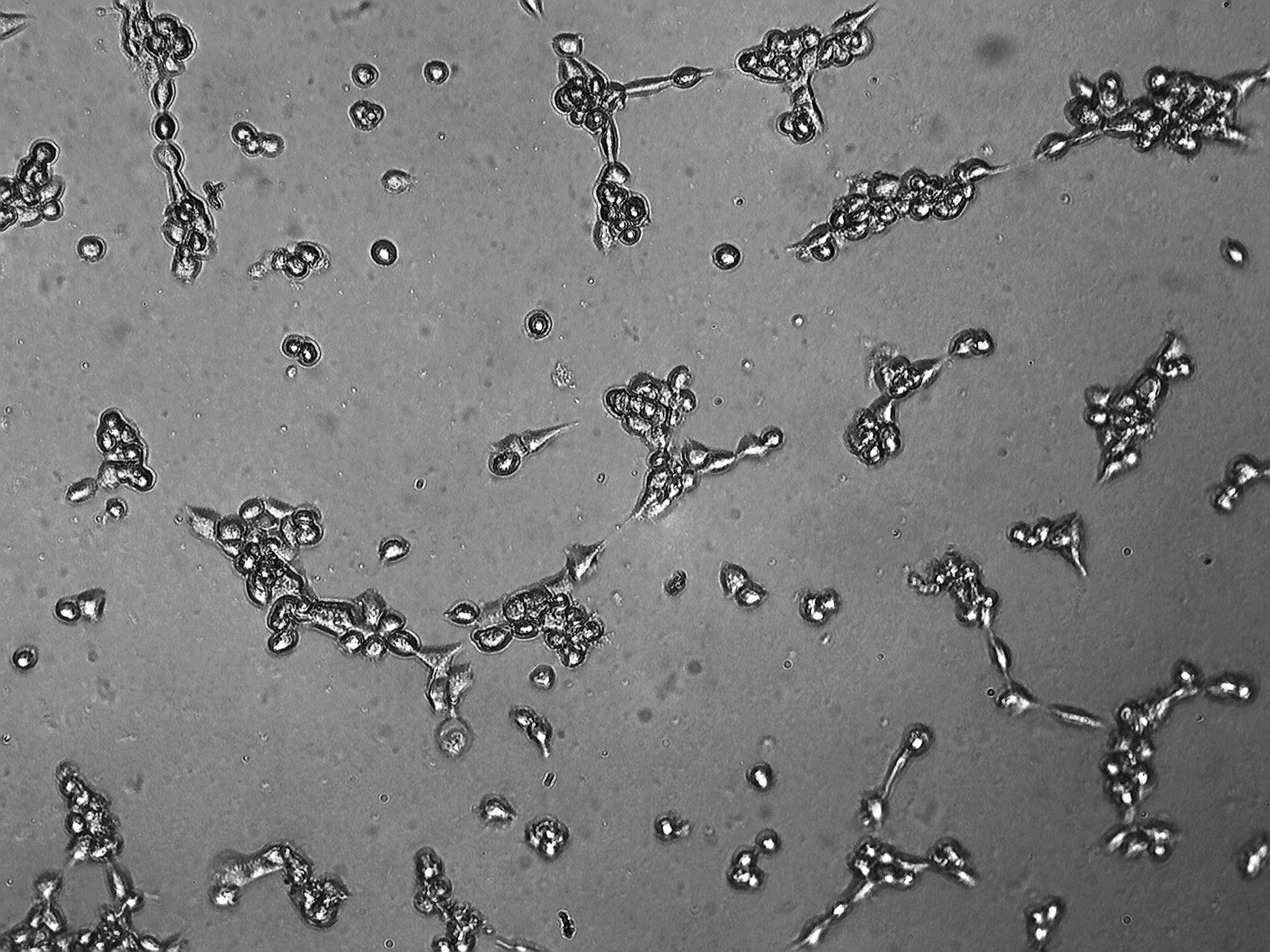

Supplement: Supplementary file 11 [file DataSheet5.ZIP › original data of HUVECs formation/7m-5a╠m.jpg]

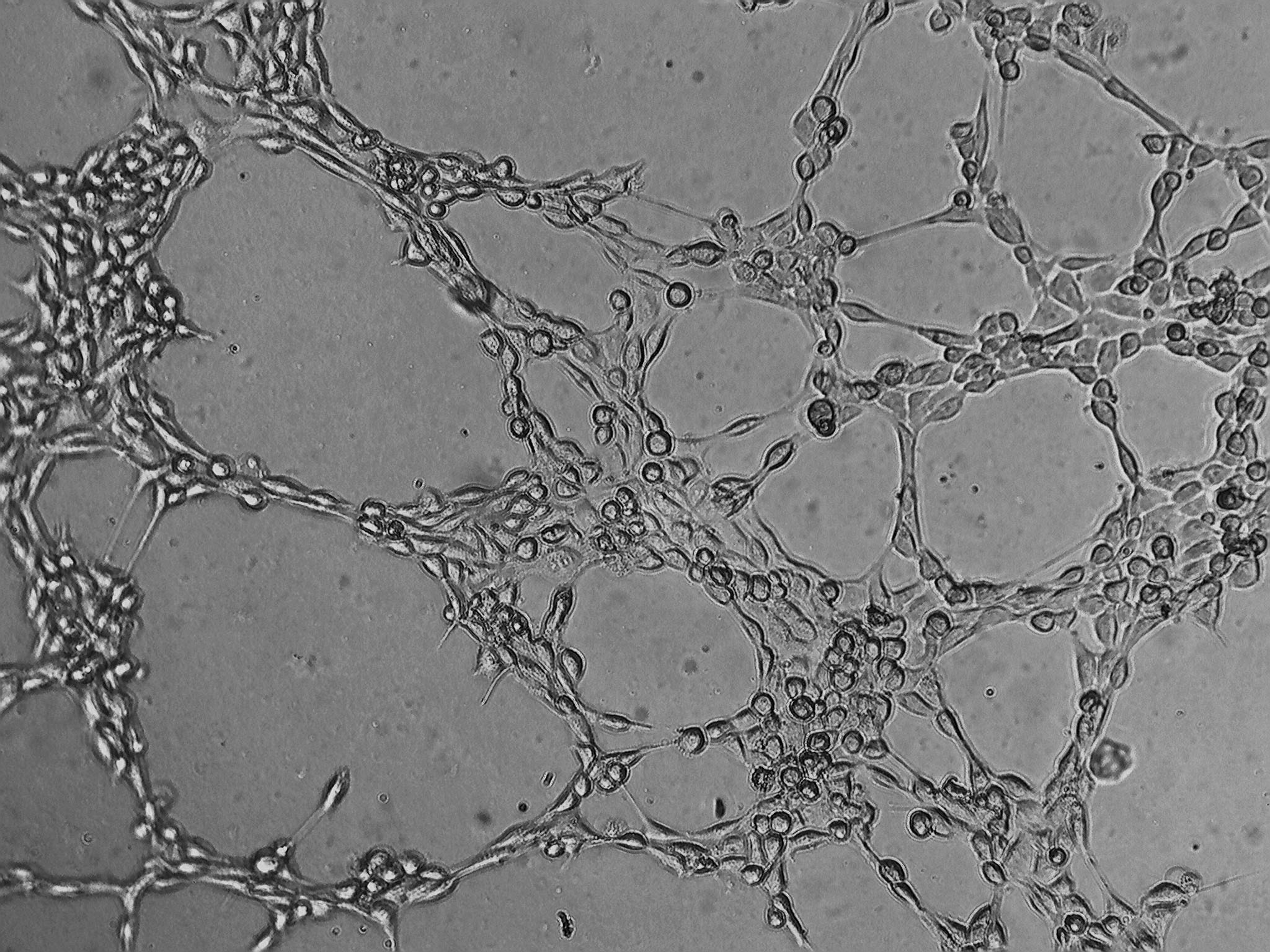

Supplement: Supplementary file 11 [file DataSheet5.ZIP › original data of HUVECs formation/NC.jpg]

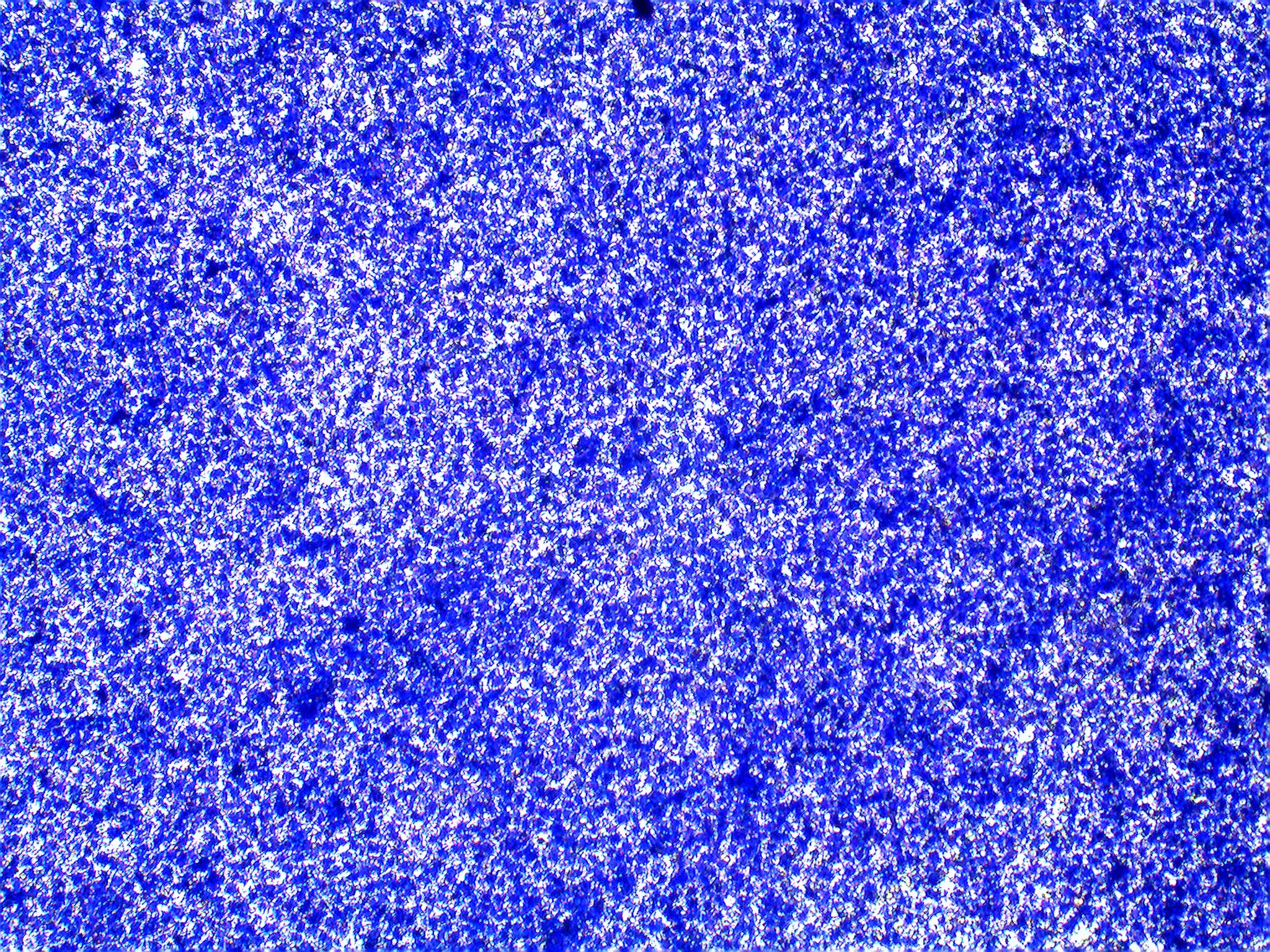

Supplement: Supplementary file 12 [file DataSheet7.ZIP › original data of transwell assay-HUVECs/7m-1a╠m.jpg]

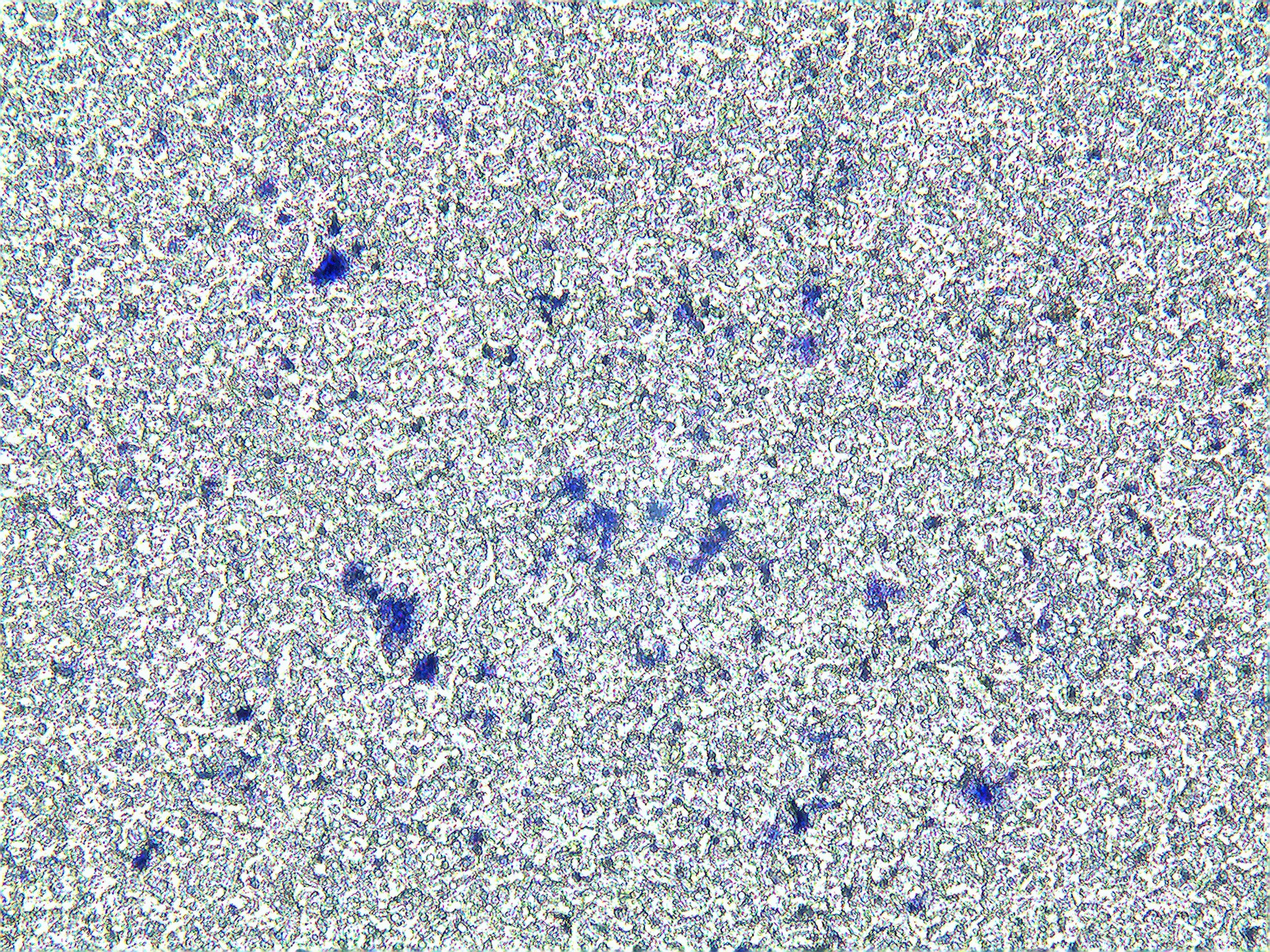

Supplement: Supplementary file 12 [file DataSheet7.ZIP › original data of transwell assay-HUVECs/7m-25a╠m.jpg]

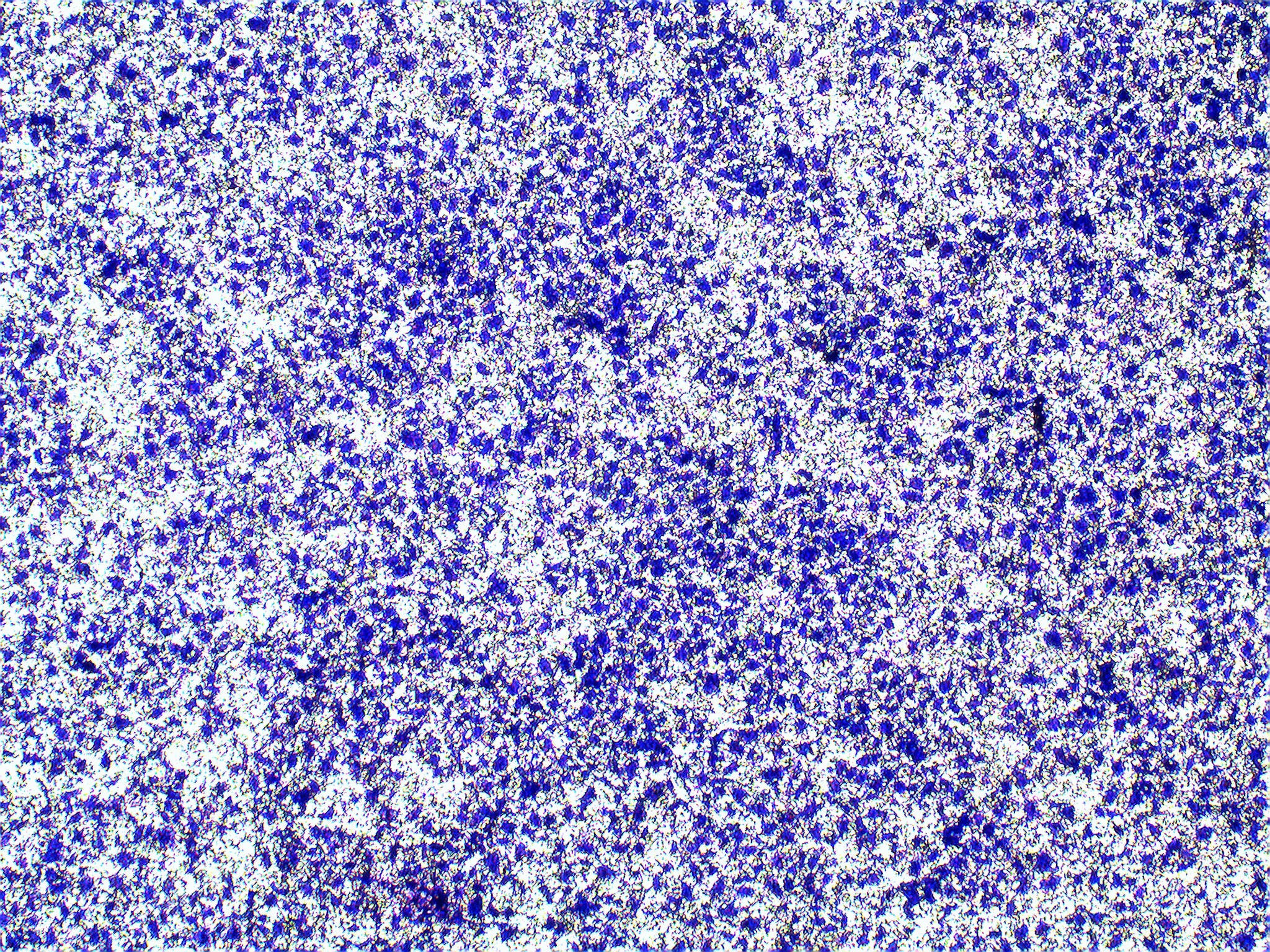

Supplement: Supplementary file 12 [file DataSheet7.ZIP › original data of transwell assay-HUVECs/7m-5a╠m.jpg]
