# Supplementary material for: Angiogenesis, Anti-Tumor, and Anti-Metastatic Activity of Novel α-Substituted Hetero-Aromatic Chalcone Hybrids as Inhibitors of Microtubule Polymerization
Source: Front Chem. 2021 Nov 19;9:766201. doi: 10.3389/fchem.2021.766201 (PMC8652888; doi:10.3389/fchem.2021.766201)
Supplement: Supplementary file 9 [file DataSheet1.pdf]

1. Chemistry

*General procedure for synthesis of compounds **6b-e, g**.*

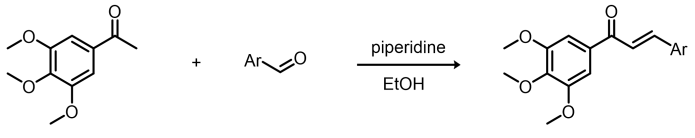

3 6b-e, g

110 mg of compound **3** (0.56 mmol, 1.0 eq) was dissolved by adding 6 mL of anhydrous EtOH, then 57.29 mg piperidine (0.67 mmol, 1.2 eq) and the corresponding aromatic aldehyde (0.8 eq) were added. The reaction mixture was allowed to reflux. After the reaction of the raw materials was completed, the reaction mixture was cooled to room temperature, and a solid was precipitated. The solid was directly filtered, washed with cooled ethanol and dried to obtain the corresponding target compounds ***6b-e, g*** (Chaitanya et al., 2018).

*(E)-3-(2-methyl-1H-indol-3-yl)-1-(3,4,5-trimethoxyphenyl)prop-2-en-1-one (**6b**):*

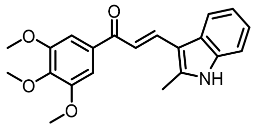

yellow solid, m.p. 236.7-238.2 °C;^{1}H NMR (400 MHz, DMSO-d_6) δ 11.81 (s, 1H), 8.06(d, J = 15.3 Hz, 1H), 8.03-8.01 (m, 1H), 7.50 (d, J = 15.3 Hz, 1H), 7.41-7.39 (m, 1H), 7.35 (s, 2H), 7.23 – 7.17 (m, 2H,), 3.92 (s, 6H), 3.77 (s, 3H), 2.60 (s, 3H);^{13}C NMR (101 MHz, DMSO-d_6) δ 187.89, 152.79, 143.90, 141.18, 137.65, 136.09, 134.31, 122.02, 121.16, 120.04, 114.35, 111.46, 109.15, 105.59, 60.13, 56.08, 11.93;IR（KBr）: 3248, 2991, 2831, 1639, 1549, 1459, 1128, 726 cm^{-1};HR-MS（ESI-TOF）Calcd for $C_{21}H_{21}NO_4$ $[M+H]^+$:352.1549, found: 352.1543.

*(E)-3-(2-phenyl-1H-indol-3-yl)-1-(3,4,5-trimethoxyphenyl)prop-2-en-1-one (**6c**) :*

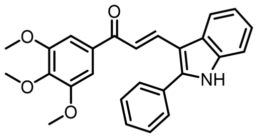

yellow solid, m.p. 60.2-61.7 °C;^{1}H NMR (400 MHz, Chloroform-d) δ 9.03 (s, 1H), 8.20 (d, J = 15.5 Hz, 1H), 8.08 – 8.01 (m, 1H), 7.63 (d, J = 15.5 Hz, 1H), 7.56 (dd, J = 7.6, 1.8 Hz, 2H), 7.49 – 7.42 (m, 4H), 7.35 – 7.30 (m, 2H), 7.28 (s, 2H), 3.93 (s, 3H), 3.91 (s, 6H);IR（KBr）: 3368, 2924, 2853, 1644, 1559, 1454, 1127 cm^{-1};HR-MS（ESI-TOF）Calcd for $C_{26}H_{23}NO_4[M+H]^+$:414.1705, found: 414.1709.

*methyl(E)-3-(3-oxo-3-(3,4,5-trimethoxyphenyl)prop-1-en-1-yl)-1H-indole-4-carboxylate (**6d**):*

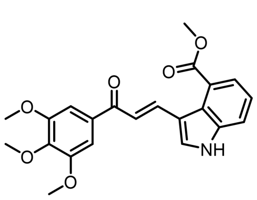

yellow solid, m.p. 221.7-223.6 °C;^{1}H NMR (400 MHz, DMSO-d_6) δ 12.32 (s, 1H), 8.59 – 8.49 (m, 2H), 7.75 (d, J = 8.1 Hz, 1H), 7.67 – 7.59 (m, 2H), 7.40 (s, 2H), 7.28 (t, J = 7.8 Hz, 1H), 3.92 (s, 9H), 3.77 (s, 3H);^{13}C NMR (101 MHz, DMSO-d_6) δ 187.77, 168.16, 152.80, 141.27, 140.28, 138.02, 133.85, 130.30, 123.59, 123.26, 121.29, 116.81, 116.54, 112.58, 105.76, 60.12, 56.08, 51.94;IR（KBr）: 3211, 1712, 1547, 1356, 1100 cm^{-1}; HR-MS（ESI-TOF）Calcd for $[M+H]^+$

$C_{22}H_{21}NO_6$:396.1447, found: 396.1442.

*methyl(E)-3-(3-oxo-3-(3,4,5-trimethoxyphenyl)prop-1-en-1-yl)-1H-indole-5-carboxylate (**6e**):*

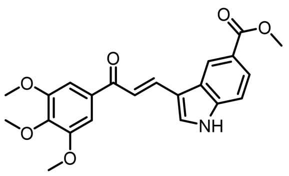

yellow solid, m.p. 218.7-220.3 °C;^{1}H NMR (400 MHz, DMSO-d_6) δ 12.25 (s, 1H), 8.65 – 8.62 (m, 1H), 8.29 (s, 1H), 8.05 (d, J = 15.5 Hz, 1H), 7.86 (dd, J = 8.6, 1.5 Hz, 1H), 7.72 (d, J = 15.5 Hz, 1H), 7.59 (d, J = 8.6 Hz, 1H), 7.38 (s, 2H), 3.93 (s, 6H), 3.88 (s, 3H), 3.78 (s, 3H);^{13}C NMR (101 MHz, DMSO-d_6) δ 187.57, 166.90, 152.82, 141.35, 139.92, 137.31, 133.68, 133.44, 125.01, 123.44, 122.30, 121.87, 116.83, 113.54, 112.50, 105.50, 60.13, 55.93, 51.84;IR（KBr）: 3370, 1701, 1568, 1265, 1122 cm^{-1};HR-MS （ESI-TOF） Calcd for $[M+H]^+$ $C_{22}H_{21}NO_6$:396.1447, found: 396.1442.

*(E)-3-(6-bromo-1H-indol-3-yl)-1-(3,4,5-trimethoxyphenyl)prop-2-en-1-one (**6g**):*

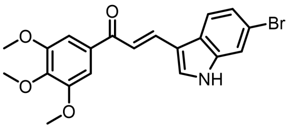

orange solid, m.p. 216.4-217.6 °C;^{1}H NMR (400 MHz, DMSO-d_6) δ 12.01 (s, 1H), 8.19 (s, 1H), 8.07 – 7.99 (m, 2H), 7.71 – 7.62 (m, 2H), 7.38 (s, 2H), 7.34 (dd, J = 8.5, 1.7 Hz, 1H), 3.92 (s, 6H), 3.77 (s, 3H);^{13}C NMR (101 MHz, DMSO-d_6) δ 187.84, 152.76, 141.35, 138.16, 137.75, 133.78, 133.04, 124.29, 123.73, 121.86, 116.40, 115.13, 114.90, 112.74, 105.75, 60.08, 56.07;IR（KBr）: 3282, 1648, 1556, 1413, 1122, 620 cm^{-1};HR-MS （ESI-TOF） Calcd for $[M+H]^+$ $C_{20}H_{18}BrNO_4$:416.0497, found: 416.0492.

*General procedure for synthesis of compound **6h**.*

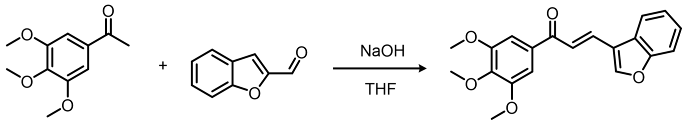

100 mg of compound **3** (0.509 mmol, 1.0 eq) was dissolved by adding 4 mL of anhydrous THF, and then 122.32 mg of NaOH (3.058 mmol, 6.0 eq) was added. After the benzofuran-2-carbaldehyde (1.6 eq) was dissolved by adding 1 mL of anhydrous THF, it was added dropwise to the above reaction mixture at 0 °C and then stirred the reaction mixture at room temperature. After the reaction of the raw materials was completed, the reaction mixture was added an appropriate amount of silica gel to evaporate the solvent, and was purified by silica-gel column chromatography (V_{PET}:V_{EA}=10:1) to obtain the target compound ***6h*** .

*(E)-3-(benzofuran-2-yl)-1-(3,4,5-trimethoxyphenyl)prop-2-en-1-one (**6h**):*

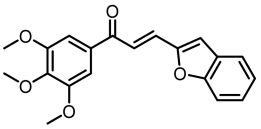

light yellow solid, m.p. 79.5-80.3 °C; ^{1}H NMR (400 MHz, Chloroform-d) δ 7.75 – 7.66 (t, J = 15.2, 12.8 Hz, 2H), 7.62 (d, J = 8.6 Hz, 1H), 7.54 (d, J = 8.3 Hz, 1H), 7.42 – 7.37 (m, 1H), 7.35 (s,

2H), 7.30 – 7.27 (m, 1H), 7.06 (s, 1H), 3.99 (s, 6H), 3.96 (s, 3H);^{13}C NMR (101 MHz, Chloroform-d) δ 188.25, 155.63, 153.22, 153.05, 142.74, 133.27, 130.87, 128.57, 126.79, 123.48, 121.93, 121.50, 112.63, 111.40, 106.15, 61.03, 56.48;IR（KBr）: 3429, 1654, 1598, 1581, 1335, 1126, 743 cm^{-1};HR-MS（ESI-TOF）Calcd for $[M+H]^+$ $C_{20}H_{18}O_5$:339.1232, found: 339.1236.

*General procedure for synthesis of compound **6j**.*

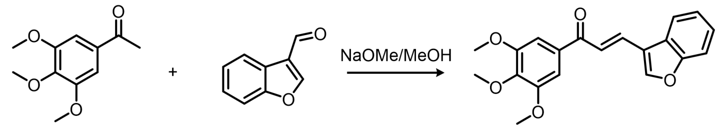

3 6j

57.54 mg of compound **3** (0.273 mmol, 0.8 eq) was dissolved in 2.5 mL of freshly prepared MeONa solution, and 50 mg of 3-benzofuranaldehyde (0.342 mmol, 1.0 eq) was added. The reaction mixture was stirred at room temperature. After the reaction of the raw materials was completed, the reaction mixture was added an appropriate amount of silica gel to evaporate the solvent, and was purified by silica-gel column chromatography (V_{PET}:V_{EA}=15:1) to obtain the target compound ***6j***.

*(E)-3-(benzofuran-3-yl)-1-(3,4,5-trimethoxyphenyl)prop-2-en-1-one (**6j**):*

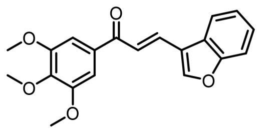

yellow solid, m.p. 117.5-120.8 °C; ^{1}H NMR (400 MHz, Chloroform-d) δ 8.02 – 7.90 (m, 3H), 7.64 – 7.56 (m, 2H), 7.44 – 7.39 (m, 2H), 7.30 (s, 2H), 3.97 (s, 6H), 3.95 (s, 3H); ^{13}C NMR (101 MHz, Chloroform-d) δ 189.24, 156.25, 153.24,148.61, 142.66, 134.50, 133.52, 125.53, 124.90, 123.90, 122.15, 120.91, 118.56, 112.21, 106.24, 61.00, 56.48;IR（KBr）: 3430, 1668, 1614, 1583, 1342, 1127, 731 cm^{-1}; HR-MS（ESI-TOF）Calcd for $[M+H]^+$ $C_{20}H_{18}O_5$:339.1232, found: 339.1236.

*General procedure for synthesis of compounds **6l-m***

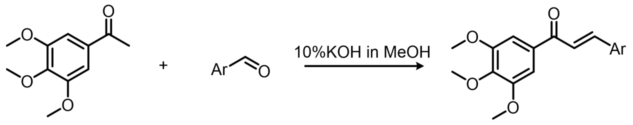

3 6l-m

60 mg of compound **3** (0.274 mmol, 0.8 eq) and the corresponding aromatic aldehyde (generally 1.0 eq) were placed in a 25 ml round-bottomed flask, and then 2 mL of 10% KOH methanol solution was added to the reaction mixture, and stirred at room temperature. After 8 h, the reaction mixture was quenched with 10% HCl (2 ml) and extracted with EA three times. The organic phase was dried and concentrated with anhydrous $MgSO_4$, and the target compounds **6m-l** were obtained by silica-gel column chromatography (Koneni et al., 2014).

*(E)-3-(dibenzo[b,d]furan-2-yl)-1-(3,4,5-trimethoxyphenyl)prop-2-en-1-one (**6l**):*

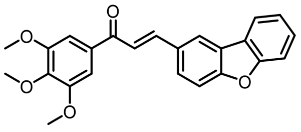

light yellow solid, m.p. 56.5-57.6 °C; ^{1}H NMR (400 MHz, Chloroform-*d*) δ 8.23 (s, 1H), 8.04 – 7.96 (m, 2H), 7.79 (dd, J = 8.6, 1.7 Hz, 1H), 7.61 (dd, J = 8.4, 4.9 Hz, 2H), 7.57 – 7.47 (m, 2H),

7.39 (t, J = 7.6 Hz, 1H), 7.32 (s, 2H), 3.98 (s, 6H), 3.96 (s, 3H); IR（KBr）: 2935, 1653, 1573, 1124, 814 cm^{-1}; HR-MS（ESI-TOF）Calcd for $[M+H]^+$ $C_{24}H_{20}O_5$:389.1389, found: 389.1384.

(E)-3-(benzofuran-5-yl)-1-(3,4,5-trimethoxyphenyl)prop-2-en-1-one ***(6m)**:*

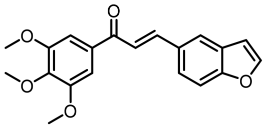

light yellow solid, m.p. 110.5-115.3 °C; ^{1}H NMR (400 MHz, Chloroform-*d*) δ 7.94 (d, J = 15.6 Hz, 1H), 7.88 (s, 1H), 7.67 (d, J = 2.2 Hz, 1H), 7.64 (dd, J = 8.6, 1.6 Hz, 1H) , 7.54 (d, J = 8.6 Hz, 1H), 7.49 (d, J = 15.6 Hz, 1H), 7.30 (s, 2H), 6.82 (dd, J = 2.1, 0.8 Hz, 1H), 3.96 (s, 6H), 3.95 (s, 3H); ^{13}C NMR (101 MHz, Chloroform-*d*) δ 189.21, 156.25, 153.17, 146.19, 145.35, 142.44, 133.70, 130.02, 128.18, 124.60, 122.22, 120.66, 112.04, 106.82, 106.09, 61.00, 56.42;IR（KBr）: 3425, 1652, 1573, 1331, 1127, 802 cm^{-1}; HR-MS（ESI-TOF）Calcd for $[M+H]^+$ $C_{20}H_{18}O_5$:339.1232, found: 339.1237.

General procedure for synthesis of compound ***6n**.*

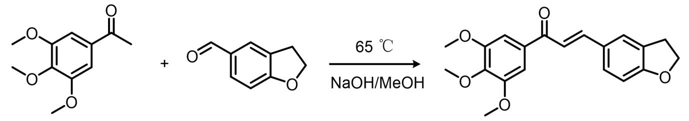

3 6n

40 mg of 2,3-dihydrobenzofuran-5-carbaldehyde (0.27 mmol, 1.0 eq) was dissolved in 4 mL of MeOH, then 43.19 mg of sodium hydroxide (1.08 mmol, 4.0 eq) and 56.76 mg of compound 3 (0.27 mmol, 1.0 eq) were added. The reaction mixture was heated to reflux and stirred for 5 h and then cooled to room temperature. After the solid precipitated, it was filtered directly, washed with cold methanol and dried to obtain the target compound **6n**.

(E)-3-(2,3-dihydrobenzofuran-5-yl)-1-(3,4,5-trimethoxyphenyl)prop-2-en-1-one ***(6n)**:*

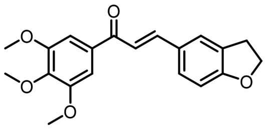

light yellow solid, m.p. 107.5-110.3 °C; ^{1}H NMR (400 MHz, Chloroform-*d*) δ 7.79 (d, J = 15.5 Hz, 1H), 7.53 (s, 1H), 7.44 (d, J = 9.6 Hz, 1H), 7.34 (d, J = 15.5 Hz, 1H), 7.27 (s, 2H), 6.82 (d, J = 8.3 Hz, 1H), 4.65 (t, J = 8.7 Hz, 2H), 3.95 (s, 6H), 3.94 (s, 3H), 3.26 (t, J = 8.7 Hz, 2H); ^{13}C NMR (101 MHz, Chloroform-*d*) δ 189.27, 162.65, 153.11, 145.15, 142.23, 133.93, 130.08, 128.21, 127.74, 125.02, 118.72, 109.84, 105.97, 71.95, 60.98, 56.40, 29.26;

IR（KBr）: 3439, 1650, 1593, 1572, 1124, 814 cm^{-1};HR-MS（ESI-TOF）Calcd for $[M+H]^+$ $C_{20}H_{20}O_5$:341.1389, found: 341.1385.

General procedure for synthesis of compounds ***6o-p**.*

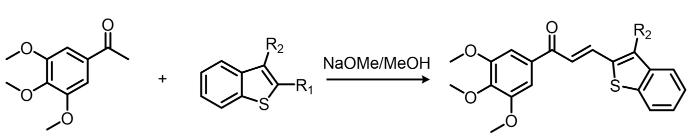

3 o-p o: R1=-CHO,R2=H p: R1=H,R2=-CHO 6o-p

50 mg of compound **3** (0.24 mmol, 1.0 eq) was added to sodium methoxide solution, and then

aromatic aldehyde (usually 1.25 eq) was added. After the reaction mixture was stirred at room temperature until the reaction of the raw materials was completed, the yellow solid was filtered out, washed with cooled methanol and dried. The corresponding target compounds **6o-p** are obtained (Karunakaran et al., 2019).

*(E)-3-(benzo[b]thiophen-2-yl)-1-(3,4,5-trimethoxyphenyl)prop-2-en-1-one (**6o**):*

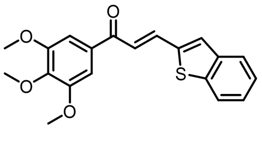

yellow solid, m.p. 115.5-116.9 °C; ^{1}H NMR (400 MHz, Chloroform-*d*) δ 8.03 (d, *J* = 15.2 Hz, 1H), 7.83 – 7.76 (m, 2H), 7.57 (s, 1H), 7.43 – 7.31 (m, 3H), 7.28 (s, 2H), 3.96 (s, 6H), 3.95 (s, 3H).^{13}C NMR (101 MHz, Chloroform-*d*) δ 183.03,147.90, 137.40, 134.92, 134.40, 132.27, 127.95, 124.58, 121.21, 119.69, 119.27, 117.43, 117.17, 100.81, 55.71, 51.13; IR（KBr）: 3050, 1654, 1574, 1131, 721 cm^{-1};HR-MS（ESI-TOF）Calcd for $[M+H]^+$ $C_{20}H_{18}O_4S$:355.1004, found: 355.1007.

*(E)-3-(benzo[b]thiophen-3-yl)-1-(3,4,5-trimethoxyphenyl)prop-2-en-1-one (**6p**):*

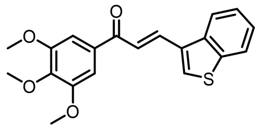

yellow solid, m.p. 95.5-96.4 °C;^{1}H NMR (400 MHz, Chloroform-*d*) δ 8.13 (d, *J* = 15.7 Hz, 1H), 8.09 (d, *J* = 7.9 Hz, 1H), 7.93 (d, *J* = 9.7 Hz, 2H), 7.60 (d, *J* = 15.7 Hz, 1H), 7.54 – 7.49 (m, 1H), 7.48 – 7.42 (m, 1H), 7.33 (s, 2H), 3.97 (d, *J* = 2.5 Hz, 9H);^{13}C NMR (101 MHz, Chloroform-*d*) δ 189.18, 153.19, 142.55, 140.54, 137.39, 136.22, 133.51, 132.35, 128.39, 125.18, 125.08, 123.10, 122.37, 122.10, 106.13, 61.00, 56.42;IR（KBr）: 3421, 3071, 1650, 1571, 1334, 1128, 730 cm^{-1};HR-MS（ESI-TOF）Calcd for $[M+H]^+$ $C_{20}H_{18}O_4S$:355.1004, found: 355.1007.

*General procedure for synthesis of compound **6q**.*

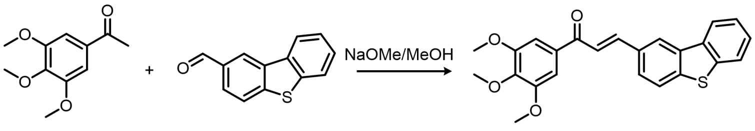

50 mg of compound **3** (0.238 mmol, 1.0 eq) was added to 2 mL of sodium methoxide solution, followed by 63.15 mg of dibenzothiophene-2-carbaldehyde (0.298 mmol, 1.25 eq). After the reaction of the raw materials disappeared, an appropriate amount of silica gel was added to evaporate the solvent, and the target compound **6q** was obtained by silica gel column chromatography.

*(E)-3-(dibenzo[b,d]thiophen-2-yl)-1-(3,4,5-trimethoxyphenyl)prop-2-en-1-one (**6q**):*

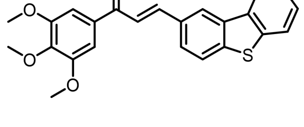

yellow solid, m.p. 118.7-119.2 °C;^{1}H NMR (400 MHz, Chloroform-*d*) δ 8.36 (s, 1H), 8.24 – 8.19 (m, 1H), 8.00 (d, *J* = 15.6 Hz, 1H), 7.92 – 7.85 (m, 2H), 7.78 (dd, *J* = 8.4, 1.5 Hz, 1H), 7.59 (d, *J* = 15.6 Hz, 1H), 7.53 – 7.48 (m, 2H), 7.32 (s, 2H), 3.98 (s, 6H), 3.96 (s, 3H);^{13}C NMR (101 MHz, Chloroform-*d*) δ 189.19, 153.21, 144.92, 142.56, 141.92, 139.82, 136.19, 135.02, 133.64, 131.41,

127.34, 125.93, 124.81, 123.24, 123.00, 122.31, 121.76, 121.35, 106.19, 61.02, 56.48;IR（KBr）: 2924, 1654, 1597, 1575, 1341, 1126, 758 cm^{-1};HR-MS（ESI-TOF）Calcd for $[M+H]^+$ $C_{24}H_{20}O_4S$:405.1161, found: 405.1166.

General procedure for synthesis of compound ***6r***.

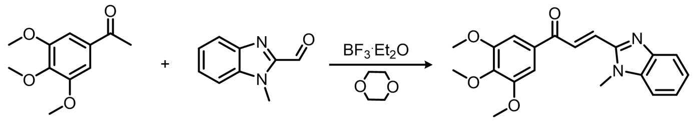

3 6r

100 mg of compound **3** (0.476 mmol, 1.0 eq) and 1-methyl-1H-benzo[d]imidazole-2-carbaldehyde (usually 1.2 eq) were placed in a 25 ml round-bottomed flask, 5 mL of anhydrous dioxane was added and $BF_3.Et_2O$ (2.38 mmol, 5.0 eq) solution was slowly added dropwise under Ar protected. Then the reaction mixture was heated to 75 °C with stirring. After the reaction of the raw materials was completed, the reaction mixture was added H_2O (5 ml), 1M NaOH (3 ml), and extracted three times with EA. The organic phase was dried with anhydrous $MgSO_4$, and then concentrated. Silica gel column chromatography was used to obtain the target compound **6r** (Narender and Reddy, 2007).

*(E)-3-(1-methyl-1H-benzo[d]imidazol-2-yl)-1-(3,4,5-trimethoxyphenyl)prop-2-en-1-one (**6r**):*

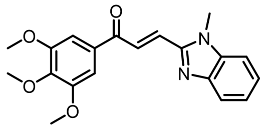

yellow solid, m.p. 123.5-125.2 °C;^{1}H NMR (400 MHz, Chloroform-*d*) δ 8.36 (d, J = 14.9 Hz, 1H), 7.91 (d, J = 14.9 Hz, 1H), 7.82 (dd, J = 6.1, 2.7 Hz, 1H), 7.42 (s, 2H), 7.41 – 7.32 (m, 3H), 3.98 (s, 6H), 3.97 (s, 3H), 3.96(s, 3H);^{13}C NMR (101 MHz, Chloroform-*d*) δ 187.76, 153.29, 148.60, 143.14, 136.36, 132.69, 127.87, 127.54, 124.18, 123.60, 120.14, 109.87, 106.33, 61.04, 56.51, 30.06;IR（KBr）: 3411, 1659, 1586, 1345, 1127, 748 cm^{-1};HR-MS（ESI-TOF）Calcd for $[M+H]^+$ $C_{20}H_{20}N_2O_4$:353.1501, found: 353.1505.

General procedure for synthesis of compound ***6s***.

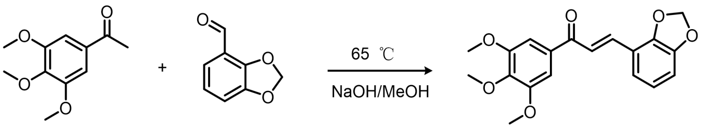

3 6s

40 mg 2,3-(methylenedioxy)benzaldehyde (0.266 mmol, 1.0 eq) was dissolved by adding 4 mL MeOH, then 42.63 mg NaOH (1.066 mmol, 4.0 eq), 56.0 mg compound **3** (0.266 mmol, 1.0 eq) were added.The temperature was raised to reflux and stirred. After TLC monitoring the raw material reaction was completed, the reaction mixture was cooled to room temperature, and a solid was precipitated. The solid was obtained by direct filtration, washed with cooled methanol and dried to obtain the target compound **6s**.

*(E)-3-(benzo[d][1,3]dioxol-4-yl)-1-(3,4,5-trimethoxyphenyl)prop-2-en-1-one (**6s**):* yellow solid, m.p. 138.4-140.3 °C;^{1}H NMR (400 MHz, Chloroform-*d*) δ 7.74 (d, J = 15.7 Hz, 1H), 7.68 (d, J = 15.7 Hz, 1H), 7.28 (s, 2H), 7.03 (dd, J = 6.3, 2.8 Hz, 1H), 6.89 – 6.85 (m, 2H), 6.11 (s, 2H), 3.95 (s, 6H), 3.94 (s, 3H);^{13}C NMR (101 MHz, Chloroform-*d*) δ 189.52, 153.13, 148.01, 146.72, 142.54, 139.08, 133.51, 124.44, 123.20, 121.96, 118.02, 110.01, 106.25, 101.50, 60.99, 56.42;IR

（KBr）: 3423, 1660, 1604, 1583, 1452, 1131, 768 cm^{-1};HR-MS（ESI-TOF）Calcd for $[M+H]^+$ $C_{19}H_{18}O_6$:343.1182, found: 343.1187.

General procedure for synthesis of compound ***4****.*

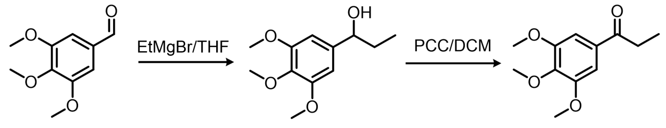

4

500 mg of 3,4,5-trimethoxybenzaldehyde (2.55 mmol, 1.0 eq) was placed in a two-necked flask, and 5 mL of anhydrous THF was added to dissolve it. Under argon protection and 0 °C, 3.83 mL of 1M ethylmagnesium bromide in THF solution (3.83 mmol, 1.5 eq) was slowly added, then stirred at room temperature for 8 h. The reaction was quenched by adding water, and was extracted three times with EA, dried the organic phase with anhydrous $MgSO_4$, and then concentrated, and obtained colorless liquid intermediate by silica gel column chromatography (V_{PET}:V_{EA}=5:1). 460 mg of this intermediate (2.033 mmol, 1.0 eq) was added to 4 mL of anhydrous DCM and appropriate amount of silica gel. At 0 °C, 876.46 mg PCC (4.066 mmol, 2.0 eq) was added to the reaction mixture. After stirring at room temperature, the reaction of the raw materials was complete after 1 h, quenched with water, extracted with DCM three times, dried with anhydrous $MgSO_4$, and then concentrated, purified by silica gel column chromatography (V_{PET}:V_{EA} = 6:1) to obtain compound **4** (Tanpure et al., 2009).

1-(3,4,5-trimethoxyphenyl)propan-1-one (4): white solid, ^1H NMR (400 MHz, Chloroform-*d*) δ 7.22 (s, 2H), 3.91 (s, 6H), 3.90 (s, 3H), 2.97 (q, *J* = 7.2 Hz, 2H), 1.22 (t, *J* = 7.2 Hz, 3H); ^{13}C NMR (101 MHz, Chloroform-*d*) δ 199.65, 153.07, 142.41, 132.26, 105.49, 60.93, 56.29, 31.58, 8.44.

General procedure for synthesis of compounds ***7b, 7e-f****.*

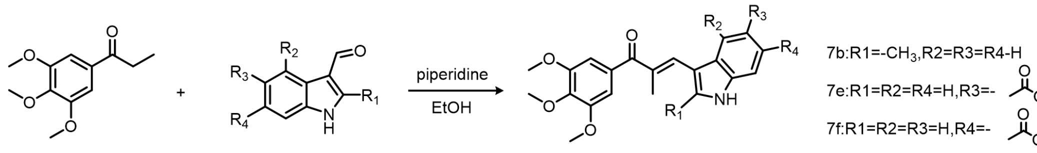

4

7b,e-f

50 mg of compound **4** (0.223 mmol, 1.0 eq) was dissolved by adding 3 mL of anhydrous EtOH, then piperidine (0.268 mmol, 1.2 eq), 4A molecular sieve (100 mg), and the corresponding aromatic aldehyde (0.8 eq) were added. The temperature was increased and the reaction was stirred under reflux conditions. After the raw material reaction was completed, added 1M HCl to the reaction mixture to adjust PH=6, extracted three times with EA, dried the organic phase with anhydrous $MgSO_4$, and then concentrated, purified by silica gel column chromatography (V_{PET}:V_{EA} = 10 :1-2:1). The corresponding target compounds **7b, 7e-f** were obtained (Yan et al., 2016).

*(E)-2-methyl-3-(2-methyl-1H-indol-3-yl)-1-(3,4,5-trimethoxyphenyl)prop-2-en-1-one (7****b****):*

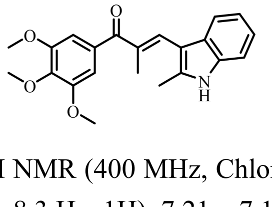

yellow solid, m.p. 152.7-153.2 °C; ^1H NMR (400 MHz, Chloroform-*d*) δ 8.24 (s, 1H), 7.47 (d, *J* = 7.3 Hz, 1H), 7.40 (s, 1H), 7.33 (d, *J* = 8.3 Hz, 1H), 7.21 – 7.13 (m, 2H), 7.07 (s, 2H), 3.93 (s, 3H),

3.91 (s, 6H), 2.40 (s, 3H), 2.14 (d, J = 1.1 Hz, 3H);^{13}C NMR (101 MHz, Chloroform-d) δ 198.44, 152.79, 141.02, 136.34, 135.48, 135.29, 134.81, 134.48, 127.33, 121.96, 120.33, 119.64, 110.61, 109.79, 107.00, 60.97, 56.27, 15.90, 13.29;IR（KBr）: 3216, 1589, 1574, 1234, 1131 cm^{-1}; HR-MS（ESI-TOF）Calcd for $[M+H]^+$ $C_{22}H_{24}O_4$:343.1705, found: 343.1709.

*methyl(E)-3-(2-methyl-3-oxo-3-(3,4,5-trimethoxyphenyl)prop-1-en-1-yl)-1H-indole-5-carboxylate (7**e**):*

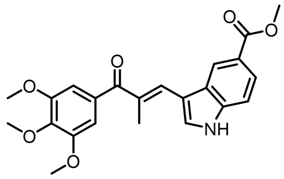

yellow solid, m.p. 210.4-213.7 °C;^{1}H NMR (400 MHz, Chloroform-d) δ 8.97 (s, 1H), 8.36 (s, 1H), 7.99 (dd, J = 8.6, 1.5 Hz, 1H), 7.67 (d, J = 2.0 Hz, 2H), 7.46 (d, J = 8.9 Hz, 1H), 7.08 (s, 2H), 3.98 (s, 3H), 3.93 (s, 9H), 2.32 (s, 3H);^{13}C NMR (101 MHz, Chloroform-d) δ 197.82, 167.68, 152.93,141.26, 138.12, 133.98, 133.15, 132.90, 127.28, 126.90, 124.67, 123.06, 121.38, 114.35, 111.26, 107.26, 61.01, 56.32, 52.01, 15.68;IR（KBr）: 3432, 3252, 1707, 1617, 1586, 1253, 1123, 740 cm^{-1};HR-MS（ESI-TOF）Calcd for $[M+H]^+$ $C_{23}H_{23}NO_6$:410.1604, found: 410.1608.

*methyl(E)-3-(2-methyl-3-oxo-3-(3,4,5-trimethoxyphenyl)prop-1-en-1-yl)-1H-indole-6-carboxylate (7**f**):*

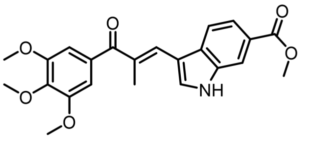

yellow solid, m.p. 229.3-231.7 °C;^{1}H NMR (400 MHz, DMSO-d_6) δ 12.27 (s, 1H), 8.13 (dd, J = 8.6, 1.9 Hz, 2H), 7.72 (dd, J = 8.4, 1.5 Hz, 1H), 7.62 – 7.57 (m, 2H), 6.99 (s, 2H), 3.86 (s, 3H), 3.82 (s, 6H), 3.78 (s, 3H), 2.24 (s, 3H);^{13}C NMR (101 MHz, DMSO-d_6) δ 196.50, 166.84, 152.44, 140.08, 135.10, 134.09, 133.55, 131.38, 130.80, 130.63, 123.44, 120.92, 117.76, 113.92, 111.55, 106.62, 60.16, 56.00, 51.91, 15.14;IR（KBr）: 3433, 1615, 1365, 773 cm^{-1};HR-MS（ESI-TOF）Calcd for $[M+H]^+$ $C_{23}H_{23}NO_6$:410.1604, found: 410.1608.

*General procedure for synthesis of compound **7h**.*

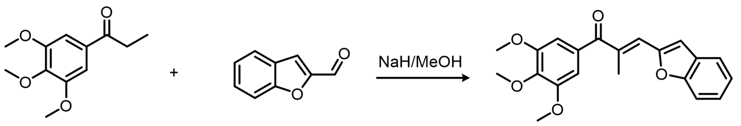

4　　　　7h

100 mg of benzo[b]furan-2-carboxaldehyde (0.684 mmol, 1.0 eq) was dissolved by adding 2.5 mL of anhydrous MeOH, then 107.42 mg of compound **4** (0.479 mmol, 0.7 eq) and 273.71 mg NaH solid (6.84 mmol, 10.0 eq) was added in batches at 0 °C then the reaction was stirred at room temperature. After the reaction of the raw materials was completed, the reaction mixture was added an appropriate amount of silica gel to evaporate the solvent, and was purified by silica-gel column chromatography (V_{PET}:V_{EA}=15:1) to obtain the target compound ***7h***.

*(E)-3-(benzofuran-2-yl)-2-methyl-1-(3,4,5-trimethoxyphenyl)prop-2-en-1-one (7**h**):*

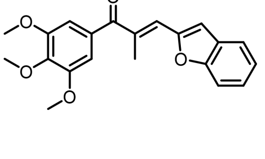

yellow oily solid m.p. 50.1-53.2 °C;^{1}H NMR (400 MHz, Chloroform-*d*) δ 7.60 (d, *J* = 7.7 Hz, 1H), 7.50 (d, *J* = 8.2 Hz, 1H), 7.37 – 7.31 (m, 1H), 7.28 – 7.22 (m, 1H), 7.10 (d, *J* = 1.08 Hz, 1H), 7.01 (s, 2H), 6.96 (s, 1H), 3.94 (s, 3H), 3.89 (s, 6H), 2.48 (d, *J* = 0.92 Hz, 3H);^{13}C NMR (101 MHz, Chloroform-*d*) δ 197.60, 155.31, 153.16, 141.48, 136.73, 133.14, 128.47, 128.28, 125.97, 123.35, 121.57, 111.39, 111.09, 106.97, 60.95, 56.25, 15.29;IR（KBr）: 3429, 1583, 1330, 1223, 996, 750 cm^{-1};HR-MS（ESI-TOF）Calcd for $[M+H]^+$ $C_{21}H_{20}O_5$:353.1389, found: 353.13869.

*General procedure for synthesis of compound **7i**.*

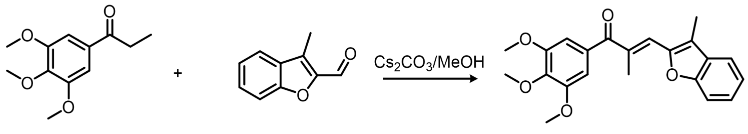

4 7i

50 mg of 3-methyl-benzofuran-2-carbaldehyde (0.312 mmol, 1.0 eq) was dissolved by adding 2.5 mL of anhydrous MeOH, then 49 mg of compound **4** (0.219 mmol, 0.7 eq) and 508.55 mg Cs_2CO_3 (1.56 mmol, 5.0 eq) was added then the reaction was stirred at room temperature. After the reaction of the raw materials was completed, the reaction mixture was added an appropriate amount of silica gel to evaporate the solvent, and was purified by silica-gel column chromatography (V_{PET}:V_{EA}=15:1) to obtain the target compound *7i.*

*(E)-2-methyl-3-(3-methylbenzofuran-2-yl)-1-(3,4,5-trimethoxyphenyl)prop-2-en-1-one (7**i**):* yellow solid, m.p. 113.2-115.1 °C;^{1}H NMR (400 MHz, Chloroform-*d*) δ 7.52 (dd, *J* = 11.6, 7.9 Hz, 2H), 7.40 – 7.35 (m, 1H), 7.30 – 7.22 (m, 2H), 7.13 – 7.10 (m, 1H), 7.02 (s, 2H), 3.94 (s, 3H), 3.90 (s, 6H), 2.55 (s, 3H), 2.25 (s, 3H);^{13}C NMR (101 MHz, Chloroform-*d*) δ 197.95, 154.96, 152.90, 149.23, 141.44，134.93, 133.50, 129.20, 126.19, 125.76, 122.87, 119.86, 111.31, 107.10, 60.97, 56.31, 15.06, 8.69;IR（KBr）: 3441, 1613, 1324, 1128, 747 cm^{-1};HR-MS（ESI-TOF）Calcd for $[M+H]^+$ $C_{22}H_{22}O_5$:367.1545, found: 367.1549.

*General procedure for synthesis of compound **7m**.*

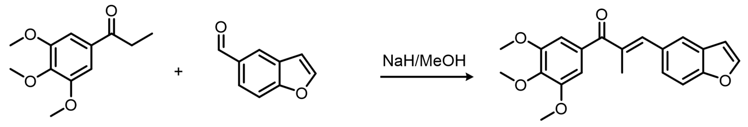

4 7m

100 mg of 1-benzofuran-5-formaldehyde (0.684 mmol, 1.0 eq) was dissolved by adding 2.5 mL of anhydrous MeOH, then 107.42 mg of compound **4** (0.479 mmol, 0.7 eq) and 273.71 mg NaH solid (6.84 mmol, 10.0 eq) was added in batches at 0 °C then the reaction was stirred at 85 °C. After the reaction of the raw materials was completed, filtered out the insoluble matter, concentrated the solution, and was purified by silica-gel column chromatography (V_{PET}:V_{EA}=5:1) to obtain the target compound ***7m***.

*(E : Z=2 : 1)3-(benzofuran-6-yl)-2-methyl-1-(3,4,5-trimethoxyphenyl)prop-2-en-1-one (7**m**):* light yellow solid, ^{1}H NMR (400 MHz, Chloroform-*d*) δ 7.64 (d, *J* =1.6Hz,1H), 7.60(d, *J* =2.2Hz,1H), 7.30 (dd, *J* =8.6Hz,1.7Hz, 1H), 7.19 (s, 1H), 7.09 (s, 1H), 6.97 (s, 2H), 6.74 (dd, *J* =2.2,0.9Hz, 1H),3.86(s, 3H), 3.83 (s, 6H), 3.75 (s, 1H), 3.70 (s, 3H), 2.25 (d, *J* =1.3Hz, 3H), 2.12(d, *J* =1.6Hz,1H). HRMS(ESI-TOF) m/z $[M + H]^+$ calcd for $C_{21}H_{20}O_5$: 353.1389, found 353.1384.

*General procedure for synthesis of compound **7n**.*

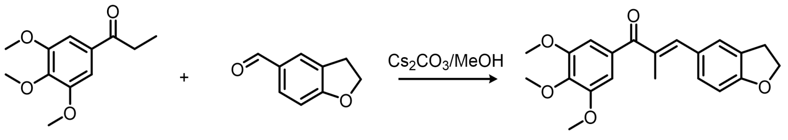

4 7n

50 mg of 2,3-dihydrobenzofuran-5-carbaldehyde (0.337 mmol, 1.0 eq) was dissolved by adding 2.5 mL of anhydrous MeOH, then 68 mg of compound **4** (0.303 mmol, 0.9 eq) and 549 mg Cs_2CO_3 (1.685 mmol, 5.0 eq) was added. The reaction was stirred at room temperature. After the reaction of the raw materials was completed, filtered out the insoluble matter, concentrated the solution, and then purified by silica-gel column chromatography (V_{PET}:V_{EA}=15:1) to obtain the target compound ***7n***.

*(E)-3-(2,3-dihydrobenzofuran-5-yl)-2-methyl-1-(3,4,5-trimethoxyphenyl)prop-2-en-1-one (7**n**):*

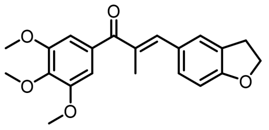

yellow solid, m.p. 99.7-100.5 °C;^{1}H NMR (400 MHz, Chloroform-*d*) δ 7.34 (s, 1H), 7.25 (s, 1H), 7.17 (s, 1H), 6.98 (s, 2H), 6.83 (d, *J* = 8.3 Hz, 1H), 4.63 (t, *J* = 8.7 Hz, 2H), 3.92 (s, 3H), 3.89 (s, 6H), 3.25 (t, *J* = 8.7 Hz, 2H), 2.28 (d, *J* = 1.2 Hz, 3H);^{13}C NMR (101 MHz, Chloroform-*d*) δ 198.70, 160.82, 152.81, 142.44, 141.11, 134.01, 130.93, 128.39, 127.64, 126.69, 109.48, 107.02, 71.74, 60.96, 56.32, 29.49, 14.76;IR（KBr）: 3439, 1599, 1580, 1330, 1130 cm^{-1};HR-MS（ESI-TOF）Calcd for $[M+H]^+$ $C_{21}H_{22}O_5$: 355.1545, found: 355.1548.

*General procedure for synthesis of compound 7**o**.*

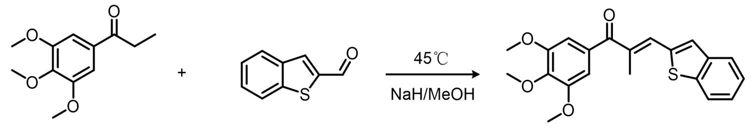

4 7o

50 mg of 1-benzothiophene-2-carboxaldehyde (0.308 mmol, 1.0 eq) was dissolved by adding 2.5 mL of anhydrous MeOH, then 48.39mg of compound **4** (0.479 mmol, 0.7 eq) and 124 mg NaH solid (6.84 mmol, 10.0 eq) was added in batches at 0 °C then the reaction was stirred at 45 °C. After the reaction of the raw materials was completed, the reaction mixture was added an appropriate amount of silica gel to evaporate the solvent, and was purified by silica-gel column chromatography (V_{PET}:V_{EA}=20:1) to obtain the target compound ***7o***.

*(E)-3-(benzo[b]thiophen-2-yl)-2-methyl-1-(3,4,5-trimethoxyphenyl)prop-2-en-1-one (7**o**):*

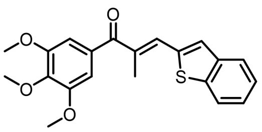

white solid, m.p. 81.4-83.2 °C; ^{1}H NMR (400 MHz, Chloroform-*d*) δ 7.86 (dd, *J* = 5.8, 3.3 Hz, 1H), 7.80 (dd, *J* = 5.8, 3.3 Hz, 1H), 7.46 (d, *J* = 4.4 Hz, 2H), 7.38 (dd, *J* = 6.0, 3.2 Hz, 2H), 7.00 (s, 2H), 3.95 (s, 3H), 3.90 (s, 6H), 2.43 (d, *J* = 0.84 Hz, 3H);^{13}C NMR (101 MHz, Chloroform-*d*) δ 197.78, 152.93, 141.51, 141.42, 138.97, 138.80, 135.60, 134.82, 133.32, 128.61, 125.63, 124.89, 124.19, 122.22, 106.98, 60.98, 56.34, 14.97;IR（KBr）: 3420, 1581, 1411, 1118 cm^{-1};HR-MS（ESI-TOF）Calcd for $[M+H]^+$ $C_{21}H_{20}O_4S$:369.1161, found: 369.1155.

*General procedure for synthesis of compound **7s**.*

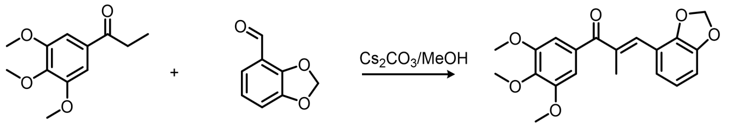

4 7s

100 mg of 2,3-(methylenedioxy)-benzaldehyde (0.666 mmol, 1.0 eq) was dissolved by adding 2.5 mL of anhydrous MeOH, then 104.5 mg of compound **4** (0.466 mmol, 0.7 eq) and 1.08g Cs_2CO_3 (3.33 mmol, 5.0 eq) was added then the reaction was stirred at room temperature. After the reaction of the raw materials was completed, the reaction mixture was added an appropriate amount of silica gel to evaporate the solvent, and was purified by silica-gel column chromatography (V_{PET}:V_{EA}=20:1) to obtain the target compound ***7s***.

*(E)-3-(benzo[d][1,3]dioxol-4-yl)-2-methyl-1-(3,4,5-trimethoxyphenyl)prop-2-en-1-one (**7s**):*

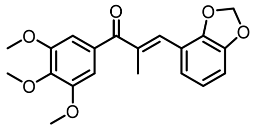

white solid, m.p. 119.7-120.9 °C;^{1}H NMR (400 MHz, Chloroform-*d*) δ 7.16 (s, 1H), 7.07 (s, 2H), 6.96 (d, *J* = 7.5 Hz, 1H), 6.88 (t, *J* = 7.8 Hz, 1H), 6.82 (dd, *J* = 7.7, 1.2 Hz, 1H), 5.98 (s, 2H), 3.93 (s, 3H), 3.91 (s, 6H), 2.22 (d, *J* = 1.3 Hz, 3H);^{13}C NMR (101 MHz, Chloroform-*d*) δ 197.99, 152.82, 147.48, 145.84, 141.55, 137.81, 133.85, 133.01, 121.92, 121.52, 118.18, 108.74, 107.29, 100.98, 60.95, 56.25, 14.99;

IR（KBr）: 3419, 1585, 1450, 1329, 1128, 999, 771 cm^{-1};HR-MS（ESI-TOF）Calcd for $[M+H]^+$ $C_{21}H_{20}O_6$: 357.1338, found: 357.1333.

*General procedure for synthesis of compound **5**.*

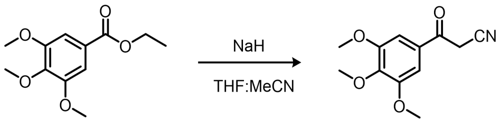

5

1.0 g of ethyl 3,4,5-trimethoxybenzoate (4.16 mmol, 1.0 eq) was added to 16 mL of dry mixed solvent (THF:MeCN=1:1), and then 0.333 g of NaH solid (8.32 mmol, 2.0 eq) was added in batches. The temperature was programmed to stir under reflux conditions. After 2 h, the reaction of the raw materials was complete. After the reaction mixture was cooled to room temperature, the solvent was evaporated, and an appropriate amount of ice water was added. It was extracted three times with EA, concentrated to obtain a yellow solid, and recrystallized with ethanol to obtain compound **5** (Wang et al., 2018).

*3-oxo-3-(3,4,5-trimethoxyphenyl)propanenitrile (**5**):*

light yellow solid, ^{1}H NMR (400 MHz, Chloroform-*d*) δ 7.15 (s, 2H), 4.09 (s, 2H), 3.94 (s, 3H), 3.92 (s, 6H); ^{13}C NMR (101 MHz, Chloroform-*d*) δ 186.01, 153.33, 144.05, 129.32, 114.00, 106.12, 61.07, 56.47; HR-MS（ESI-TOF）Calcd for $[M+H]^+$ $C_{12}H_{13}NO_4$:236.2390, found: 236.2396.

*General procedure for synthesis of compound **8a-t**.*

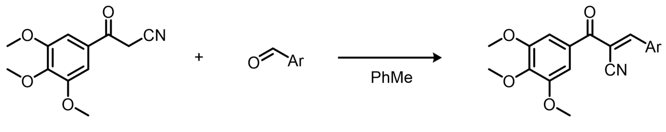

50 mg of compound **5** (0.212 mmol, 1.0 eq) was added to 5 mL of anhydrous toluene, then the corresponding aromatic aldehyde (usually 0.7 eq) was added, and piperidine (0.78 eq) and glacial acetic acid (0.82 eq) were quickly added dropwise to the reaction mixture. Then the reaction mixture was transferred to 60 °C and heat and stirred for 3-12 h. After the reaction of the raw materials was completed, the reaction mixture was cooled to room temperature, and most of the reaction mixtures had solid precipitation (some reaction mixtures were still clear solutions, then the reaction mixture was transferred to ice water and stirred for 30 min to precipitate solids), the reaction mixture was directly filtered to obtain the solid and washed with cooled toluene and dried to obtain the corresponding target compounds **8a-t** (Wang et al., 2016).

*(E)-3-(1H-indol-3-yl)-2-(3,4,5-trimethoxybenzoyl)acrylonitrile (**8a**):*

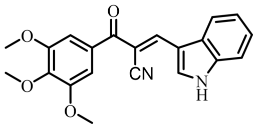

white solid, m.p. 224.2-229.3 °C; 1H NMR (400 MHz, Chloroform-d) δ 9.24 (s, 1H), 8.79 (d, J = 3.2 Hz, 1H), 8.70 (s, 1H), 7.84 (dd, J = 6.3, 2.2 Hz, 1H), 7.51 (dd, J = 6.3, 2.2 Hz, 1H), 7.40 – 7.33 (m, 2H), 7.23 (s, 2H), 3.96 (s, 3H), 3.94 (s, 6H);13C NMR (101 MHz, Chloroform-d) δ 153.01, 147.71, 135.68, 130.93, 127.70, 124.51, 122.93, 118.45, 112.23, 112.05, 106.76, 102.22, 61.04, 56.37;

IR（KBr）: 3307, 2925, 2217, 1334, 1232, 1126, 749 cm^{-1}; HR-MS（ESI-TOF）Calcd for $[M+H]^+$ $C_{21}H_{18}N_2O_4$: 363.1345, found: 363.1349.

*(E)-3-(2-methyl-1H-indol-3-yl)-2-(3,4,5-trimethoxybenzoyl)acrylonitrile (**8b**):*

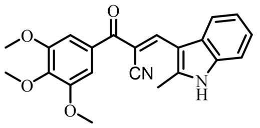

yellow solid, m.p. 216.5-218.2 °C;1H NMR (400 MHz, Chloroform-d) δ 8.76 (s, 1H), 8.53 (s, 1H), 8.22 (dd, J = 6.8, 1.5 Hz, 1H), 7.38-7.35 (m, 1H), 7.31 (td, J = 7.0, 1.5 Hz, 2H), 7.25 (s, 2H), 3.95 (s, 3H), 3.95 (s, 6H), 2.68 (s, 3H);

IR（KBr）: 3299, 2214, 1561, 1215, 1127, 757 cm^{-1}; HR-MS（ESI-TOF）Calcd for $[M+H]^+$ $C_{22}H_{20}N_2O_4$: 377.1501, found: 377.1506.

*(E)-2-((l2-azaneylidene)-l3-methyl)-3-(2-phenyl-1H-indol-3-yl)-1-(3,4,5-trimethoxyphenyl)prop-2-en-1-one (**8c**):*

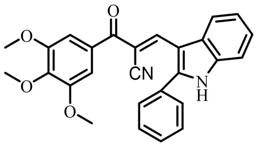

yellow solid, m.p. 253.8-255.9 °C;^{1}H NMR (400 MHz, DMSO-d_6) δ 12.94 (s, 1H), 8.40 (d, *J* = 7.5 Hz, 1H), 8.18 (s, 1H), 7.62-7.54 (m, 6H), 7.39-7.32 (m, 2H), 7.09 (s, 2H), 3.80 (s, 6H), 3.74 (s, 3H);

IR（KBr）: 3228, 2206, 1561, 1453, 1223, 1124, 751 cm^{-1}; HR-MS（ESI-TOF）Calcd for $[M+H]^+$ $C_{27}H_{22}N_2O_4$:439.1658, found: 439.1654.

methyl(E)-3-(2-cyano-3-oxo-3-(3,4,5-trimethoxyphenyl)prop-1-en-1-yl)-1H-indole-4-carboxylate ***(8d)****:*

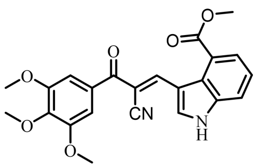

yellow solid, m.p. 176.7-178.2 °C;^{1}H NMR (400 MHz, Chloroform-*d*) δ 9.81 (s, 1H), 9.30 (s, 1H), 8.97 (s, 1H), 7.89 (d, J = 7.5 Hz, 1H), 7.71 (d, J = 8.1 Hz, 1H), 7.36 (t, J = 7.8 Hz, 1H), 7.17 (s, 2H), 3.96 (s, 6H), 3.95 (s, 3H), 3.86 (s, 3H);^{13}C NMR (101 MHz, Chloroform-*d*) δ 189.81, 167.95, 152.94, 152.40, 141.77, 137.35, 132.75, 131.93, 126.35, 125.03, 124.28, 123.31, 116.96, 111.82, 106.84, 103.29, 60.99, 56.28, 52.37; IR（KBr）: 3283, 2202, 1720, 1328, 1216, 1126, 735 cm^{-1};HR-MS（ESI-TOF）Calcd for $[M+H]^+$ $C_{23}H_{20}N_2O_6$: 421.1400, found: 421.1394.

methyl(E)-3-(2-cyano-3-oxo-3-(3,4,5-trimethoxyphenyl)prop-1-en-1-yl)-1H-indole-5-carboxylate ***(8e)****:*

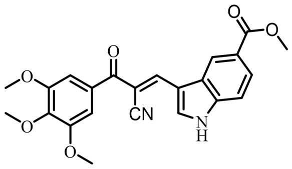

yellow solid, m.p. >260 °C;^{1}H NMR (400 MHz, DMSO-d_6) δ 8.78 (s, 1H), 8.60 (s, 1H), 8.53 (s, 1H), 7.90 (d, J = 8.6 Hz, 1H), 7.69 (d, J = 8.7 Hz, 1H), 7.18 (s, 2H), 3.88 (s, 3H), 3.87 (s, 6H), 3.80 (s, 3H);^{13}C NMR (101 MHz, DMSO-d_6) δ 188.26, 166.69, 152.64, 147.19, 141.21, 135.02, 131.98, 127.10, 124.28, 123.32, 120.76, 113.46, 111.37, 109.31, 106.76, 105.12, 60.19, 56.11, 51.98;IR（KBr）: 3287, 2203, 1724, 1323, 1226, 1120, 744 cm^{-1};HR-MS（ESI-TOF）Calcd for $[M+H]^+$ $C_{23}H_{20}N_2O_6$: 421.1400, found: 421.1405.

methyl(E)-3-(2-cyano-3-oxo-3-(3,4,5-trimethoxyphenyl)prop-1-en-1-yl)-1H-indole-6-carboxylate ***(8f)****:*

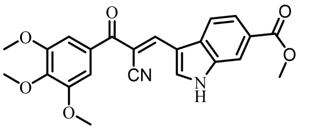

yellow solid, m.p. 239.7-241.0 °C;^{1}H NMR (400 MHz, Chloroform-*d*) δ 9.50 (s, 1H), 8.91 (s, 1H), 8.67 (s, 1H), 8.27 (s, 1H), 8.03 (dd, J = 8.4, 1.3 Hz, 1H), 7.88 (d, J = 8.4 Hz, 1H), 7.24 (s, 2H), 3.98 (s, 3H), 3.97 (s, 3H), 3.95 (s, 6H);^{13}C NMR (101 MHz, Chloroform-*d*) δ 186.33, 167.23, 153.04, 147.07, 135.16, 133.09, 131.66, 131.17, 126.37, 124.31, 123.74, 118.24, 114.46, 111.99, 106.80, 103.29, 61.07, 56.38, 52.37;IR（KBr）: 3274, 2000, 1723, 1291, 1230, 1131, 750 cm^{-1};HR-MS（ESI-TOF）Calcd for $[M+H]^+$ $C_{23}H_{20}N_2O_6$: 421.1400, found: 421.1405.

(E)-3-(6-bromo-1H-indol-3-yl)-2-(3,4,5-trimethoxybenzoyl)acrylonitrile ***(8g)****:*

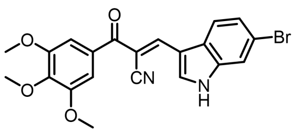

yellow solid, m.p. 266.5-268.2 °C;^{1}H NMR (400 MHz, DMSO-d_6) δ 12.49 (s, 1H), 8.70 (s, 1H), 8.51 (s, 1H), 7.90 (d, J = 8.5 Hz, 1H), 7.80 (d, J = 1.6 Hz, 1H), 7.39 (dd, J = 8.5, 1.7 Hz, 1H), 7.15 (s, 2H), 3.84 (s, 6H), 3.79 (s, 3H);IR（KBr）: 3297, 2199, 1556, 1331, 1227,1125 cm^{-1}; HR-MS（ESI-TOF）Calcd for $[M+H]^+$ $C_{21}H_{17}BrN_2O_4$: 442.0450, found: 442.0454.

*(E)-3-(benzofuran-2-yl)-2-(3,4,5-trimethoxybenzoyl)acrylonitrile (**8h**):*

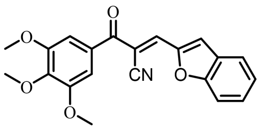

yellow solid, m.p. 168.3-169.5 °C;^{1}H NMR (400 MHz, Chloroform-d) δ 8.10 (s, 1H), 7.72 (d, J = 9.0 Hz, 2H), 7.62 (d, J = 8.4 Hz, 1H), 7.52 (t, J = 7.8 Hz, 1H), 7.35 (t, J = 7.5 Hz, 1H), 7.26 (s, 2H), 3.97 (s, 3H), 3.95 (s, 6H);^{13}C NMR (101 MHz, Chloroform-d) δ 186.24, 156.54, 153.06, 149.95, 142.98, 140.92, 130.55, 129.15, 127.74, 124.34, 122.97, 118.09, 117.13, 112.27, 107.84, 107.04, 61.06, 56.39;IR(KBr): 3442, 2215, 1599, 1581, 1337, 1129, 752 cm^{-1};HR-MS(ESI-TOF) Calcd for $[M+H]^+$ $C_{21}H_{17}NO_5$: 364.1185, found: 364.1189.

*(E)-3-(3-methylbenzofuran-2-yl)-2-(3,4,5-trimethoxybenzoyl)acrylonitrile (**8i**):*

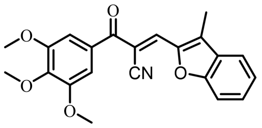

yellow solid, m.p. 136.7-138.5 °C;^{1}H NMR (400 MHz, Chloroform-d) δ 8.13 (s, 1H), 7.64 (d, J = 7.8 Hz, 1H), 7.61 (d, J = 8.4 Hz, 1H), 7.55 – 7.50 (m, 1H), 7.36 – 7.31 (m, 1H), 7.29 (s, 2H), 3.96 (s, 3H), 3.95 (s, 6H), 2.52 (s, 3H);^{13}C NMR (101 MHz, Chloroform-d) δ 186.84, 156.02, 152.97, 146.69, 142.81, 137.35, 130.85, 129.76, 128.75, 123.80, 121.17, 117.63, 112.40, 107.06, 105.62, 61.04, 56.37, 9.29;IR（KBr）: 3442, 2212, 1600, 1581, 1336, 1128, 752 cm^{-1};HR-MS（ESI-TOF） Calcd for $[M+H]^+$ $C_{22}H_{19}NO_5$: 378.1341, found: 378.1346.

*(E)-3-(benzofuran-3-yl)-2-(3,4,5-trimethoxybenzoyl)acrylonitrile (**8j**):*

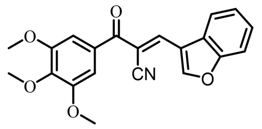

yellow solid, m.p. 176.5-178.4 °C;^{1}H NMR (400 MHz, Chloroform-d) δ 8.97 (s, 1H), 8.43 (s, 1H), 7.79 (d, J = 8.2 Hz, 1H), 7.62 (d, J = 7.7 Hz, 1H), 7.48 – 7.39 (m, 2H), 7.26 (s, 2H), 3.97 (s, 3H), 3.95 (s, 6H);^{13}C NMR (101 MHz, Chloroform-d) δ 186.06, 155.14, 153.11, 149.85, 144.80,142.99, 130.71, 126.27, 125.62, 124.46, 119.24, 118.35, 115.92, 112.25, 108.83, 106.99, 61.07, 56.40; IR（KBr）: 3445, 2204, 1580, 1335, 1128, 749 cm^{-1};HR-MS（ESI-TOF）Calcd for $[M+H]^+$ $C_{21}H_{17}NO_5$: 364.1185, found: 364.1189.

*(E)-3-(6-nitrobenzo[d][1,3]dioxol-5-yl)-2-(3,4,5-trimethoxybenzoyl)acrylonitrile (**8k**):*

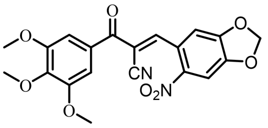

yellow solid, m.p. 160.1-163.8 °C;^{1}H NMR (400 MHz, Chloroform-d) δ 8.33 (s, 1H), 7.75 (s, 1H), 7.26 (s, 2H), 7.17 (d, J = 7.6 Hz, 1H), 6.26 (s, 2H), 3.98 (s, 6H), 3.96 (s, 3H);^{13}C NMR (101 MHz,

Chloroform-*d*) δ 187.21, 153.30, 152.83, 152.53, 150.43, 143.27, 142.36, 129.71, 129.04, 128.23, 125.29, 124.98, 115.06, 114.89, 109.16, 107.14, 106.14, 104.10, 61.05, 56.41;IR（KBr）: 3426, 2228, 1583, 1508, 1129, 1033 cm^{-1};HR-MS（ESI-TOF）Calcd for $[M+H]^+$ $C_{20}H_{16}N_2O_8$: 413.0985, found: 413.0988.

*(E)-3-(dibenzo[b,d]furan-2-yl)-2-(3,4,5-trimethoxybenzoyl)acrylonitrile (**8l**):*

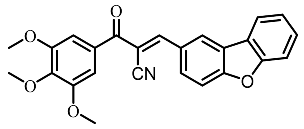

yellow solid, m.p. 181.3-182.6 °C;^{1}H NMR (400 MHz, Chloroform-*d*) δ 8.73 (d, *J* = 1.8 Hz, 1H), 8.29 (s, 1H), 8.16 (dd, *J* = 8.7, 1.8 Hz, 1H), 8.03 (d, *J* = 7.6 Hz, 1H), 7.68 (d, *J* = 8.7 Hz, 1H), 7.62 (d, *J* = 8.2 Hz, 1H), 7.54 (td, *J* = 7.3, 1.2 Hz, 1H), 7.42 (td, *J* = 7.8, 0.9 Hz, 1H), 7.24 (s, 2H), 3.97 (s, 3H), 3.95 (s, 6H);^{13}C NMR (101 MHz, Chloroform-*d*) δ 187.43, 158.90, 156.92, 155.73, 153.07, 142.85, 131.04, 130.79, 128.48, 126.94, 125.58, 124.11, 123.71, 123.11, 121.22, 117.86, 112.77, 112.06, 108.05, 107.06, 61.06, 56.41;IR（KBr）: 3422, 2205, 1664, 1573, 1334, 1135, 749 cm^{-1};HR-MS（ESI-TOF）Calcd for $[M+H]^+$ $C_{25}H_{19}NO_5$: 414.1341, found: 414.1346.

*(E)-3-(benzofuran-5-yl)-2-(3,4,5-trimethoxybenzoyl)acrylonitrile (**8m**):*

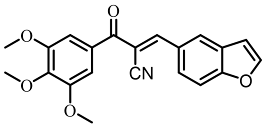

yellow solid, m.p. 169.7-172.1 °C; ^{1}H NMR (400 MHz, Chloroform-*d*) δ 8.45 (s, 1H), 8.26 (s, 1H), 8.01 (dd, *J* = 8.7, 1.5 Hz, 1H), 7.75 (d, *J* = 2.1 Hz, 1H), 7.65 (d, *J* = 8.7 Hz, 1H), 7.23 (s, 2H), 6.91 (d, *J* = 1.4 Hz, 1H), 3.98 (s, 3H), 3.96 (s, 6H);^{13}C NMR (101 MHz, Chloroform-*d*) δ 187.57, 157.45, 156.24, 153.05, 146.94, 142.79, 130.81, 128.55, 128.20, 127.07, 125.11, 117.81, 112.56, 108.04, 107.16, 107.03, 61.05, 56.38;IR（KBr）: 3421, 2201, 1660, 1570, 1334, 1136, 736 cm^{-1};HR-MS（ESI-TOF）Calcd for $[M+H]^+$ $C_{21}H_{17}NO_5$: 364.1185, found: 364.1189.

*(E)-3-(2,3-dihydrobenzofuran-5-yl)-2-(3,4,5-trimethoxybenzoyl)acrylonitrile (**8n**):*

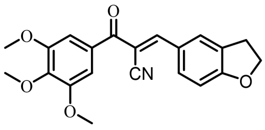

yellow solid, m.p. 173.4-175.0 °C;^{1}H NMR (400 MHz, Chloroform-*d*) δ 8.15 (s, 1H), 8.07 (s, 1H), 7.77 (dd, *J* = 8.5, 1.7 Hz, 1H), 7.18 (s, 2H), 6.90 (d, *J* = 8.4 Hz, 1H), 4.72 (t, *J* = 8.8 Hz, 2H), 3.95 (s, 3H), 3.93 (s, 6H), 3.31 (t, *J* = 8.7 Hz, 2H);^{13}C NMR (101 MHz, Chloroform-*d*) δ 187.94, 165.27, 155.76, 152.98, 142.49, 135.15, 131.22, 129.08, 127.59, 125.01, 118.38, 110.35, 106.89, 105.18, 72.68, 61.02, 56.35,28.96;IR（KBr）: 3443, 2202, 1659, 1493, 1332, 1131, 752 cm^{-1};HR-MS（ESI-TOF）Calcd for $[M+H]^+$ $C_{21}H_{19}NO_5$: 366.1341, found: 366.1346.

*(E)-3-(benzo[b]thiophen-2-yl)-2-(3,4,5-trimethoxybenzoyl)acrylonitrile (**8o**):*

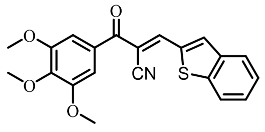

yellow solid, m.p. 187.5-189.3 °C;^{1}H NMR (400 MHz, Chloroform-*d*) δ 8.42 (s, 1H), 8.07 (s, 1H),

7.92 (dd, J = 8.0, 4.0 Hz, 2H), 7.52 (td, J = 7.7, 1.1 Hz, 1H), 7.45 (td, J = 8.1, 0.8 Hz, 1H), 7.25 (s, 2H), 3.97 (s, 3H), 3.95 (s, 6H);^{13}C NMR (101 MHz, Chloroform-*d*) δ 186.40, 153.08, 148.57, 143.21, 142.95, 138.21, 136.24, 135.88, 130.73, 128.36, 125.69, 125.61, 122.80, 117.52, 107.88, 107.01, 61.07, 56.41;IR（KBr）: 3431, 2202, 1655, 1569, 1333, 1131, 742 cm^{-1};HR-MS（ESI-TOF） Calcd for $[M+H]^+$ $C_{21}H_{17}NO_4S$: 380.0957, found: 380.0952.

*(E)-3-(benzo[b]thiophen-3-yl)-2-(3,4,5-trimethoxybenzoyl)acrylonitrile (**8p**):*

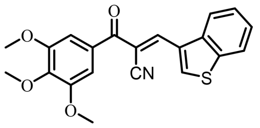

yellow solid, m.p. 201.8-203.1 °C;^{1}H NMR (400 MHz, Chloroform-*d*) δ 9.10 (s, 1H), 8.54 (s, 1H), 7.95 (dd, J = 7.4, 4.8 Hz, 2H), 7.57 – 7.47 (m, 2H), 7.26 (s, 2H), 3.97 (s, 3H), 3.95 (s, 6H);13C NMR (101 MHz, Chloroform-d) δ 186.88, 153.11, 144.64, 142.92, 139.29, 138.17, 134.72, 130.84, 128.35, 125.94, 125.71, 123.13, 120.91, 118.43, 108.79, 107.02, 61.08, 56.40;IR（KBr）: 3426, 2199, 1664, 1574, 1332, 1126, 756 cm-1;HR-MS（ESI-TOF） Calcd for $[M+H]^+$ $C_{21}H_{17}NO_4S$:380.0957, found: 380.0952.

*(E)-3-(dibenzo[b,d]thiophen-2-yl)-2-(3,4,5-trimethoxybenzoyl)acrylonitrile (**8q**):*

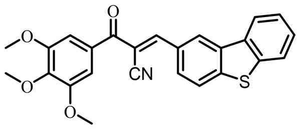

yellow solid, m.p. 199.7-201.1 °C;^{1}H NMR (400 MHz, Chloroform-*d*) δ 8.86 (s, 1H), 8.29 (s, 1H), 8.25 – 8.20 (m, 1H), 8.12 (dd, J = 8.5, 1.6 Hz, 1H), 7.96 (d, J = 8.4 Hz, 1H), 7.90 – 7.86 (m, 1H), 7.56 – 7.50 (m, 2H), 7.25 (s, 2H), 3.97 (s, 3H), 3.95 (s, 6H);^{13}C NMR (101 MHz, Chloroform-*d*) δ 187.34, 155.63, 153.08, 145.17, 142.90, 139.68, 136.32, 134.66, 130.76, 128.69, 128.22, 127.86, 125.23, 124.46, 123.58, 123.00, 122.05, 117.86, 108.52, 107.10; 61.06, 56.41;IR（KBr）: 3440, 2208, 1567, 1335, 1143, 756 cm^{-1};HR-MS（ESI-TOF）Calcd for $[M+H]^+$ $C_{25}H_{19}NO_4S$: 430.1113, found: 430.1116.

*(E)-3-(1-methyl-1H-benzo[d]imidazol-2-yl)-2-(3,4,5-trimethoxybenzoyl)acrylonitrile (**8r**):*

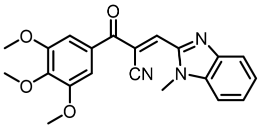

yellow solid, m.p. 201.8-203.1 °C;^{1}H NMR (400 MHz, Chloroform-*d*) δ 8.18 (s, 1H), 7.95 (d, J = 8.1 Hz, 1H), 7.46 – 7.38 (m, 3H), 7.34 (s, 2H), 3.97 (s, 6H), 3.95 (s, 6H);^{13}C NMR (101 MHz, Chloroform-*d*) δ 185.95, 153.03, 144.77, 143.80, 143.38, 137.30, 136.16, 130.00, 126.19, 124.32, 121.96, 116.76, 113.77, 110.13, 107.35, 61.07, 56.44, 30.26;IR（KBr）: 3428, 2229, 1666, 1586, 1336, 1128, 754 cm^{-1};HR-MS（ESI-TOF）Calcd for $[M+H]^+$ $C_{21}H_{19}N_3O_4$: 378.1454, found: 378.1458.

*(E)-3-(benzo[d][1,3]dioxol-4-yl)-2-(3,4,5-trimethoxybenzoyl)acrylonitrile (**8s**):*

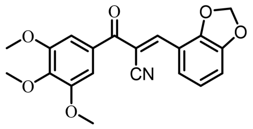

yellow solid, m.p. 176.3-177.8 ℃;^{1}H NMR (400 MHz, Chloroform-*d*) δ 8.24 (s, 1H), 7.92 (dd, *J* = 7.0, 2.2 Hz, 1H), 7.18 (s, 2H), 6.99 – 6.96 (m, 2H), 6.08 (s, 2H), 3.96 (s, 3H), 3.93 (s, 6H);^{13}C NMR (101 MHz, Chloroform-*d*) δ 187.30, 153.06, 149.19, 148.00, 147.17, 142.92, 130.47, 122.42, 119.90, 117.09, 114.75, 112.67, 110.06, 107.13, 101.96, 61.05, 56.38; IR（KBr）: 3440, 2210, 1581, 1457, 1336, 1127 cm^{-1};HR-MS （ESI-TOF） Calcd for $[M+H]^+$ $C_{20}H_{17}NO_6$: 368.1134, found: 368.1138.

(E)-3-(6-bromobenzo[d][1,3]dioxol-5-yl)-2-(3,4,5-trimethoxybenzoyl)acrylonitrile (***8t***)*:*

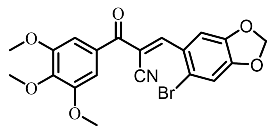

yellow solid, m.p. 207.1-209.0 ℃;^{1}H NMR (400 MHz, Chloroform-*d*) δ 8.37 (s, 1H), 7.91 (s, 1H), 7.18 (s, 3H), 6.13 (s, 2H), 3.96 (s, 3H), 3.94 (s, 6H); IR（KBr）: 3424, 2200, 1581, 1486, 1336, 1130 cm^{-1}; HR-MS（ESI-TOF）Calcd for $[M+H]^+$ $C_{20}H_{16}BrNO_6$: 447.0239, found: 447.0234.

2. ^{1}H and ^{13}C NMR spectra of synthetized products

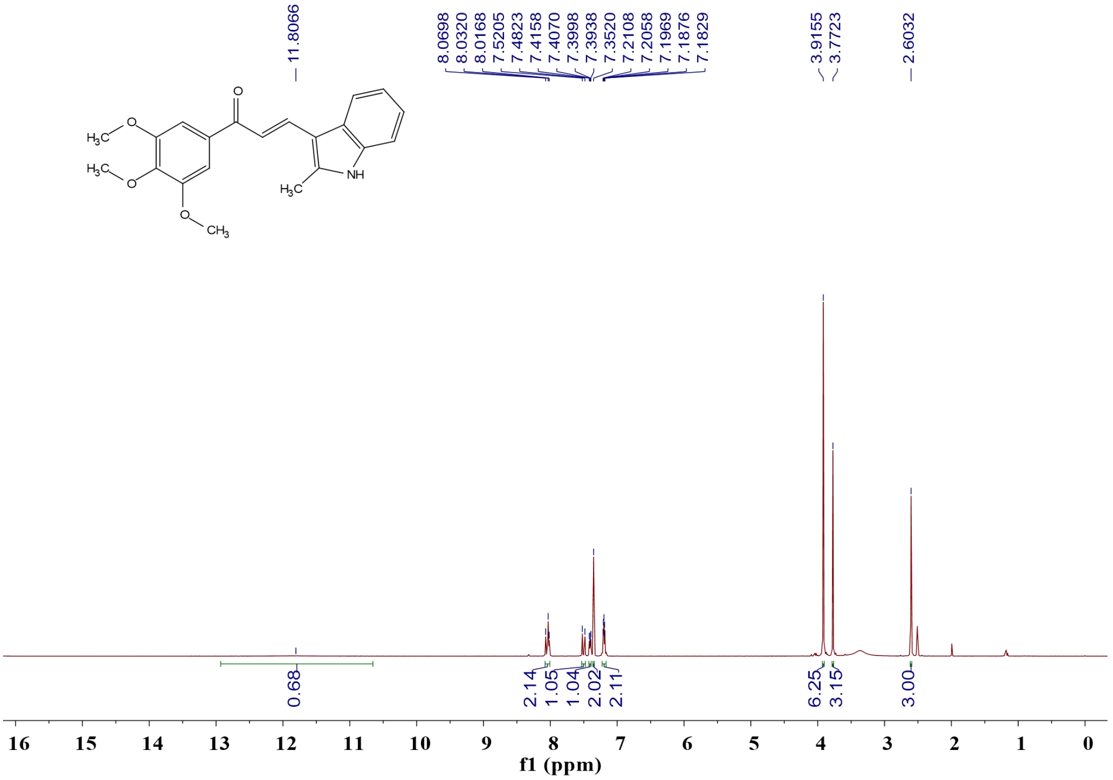

^{1}H NMR of **6b**

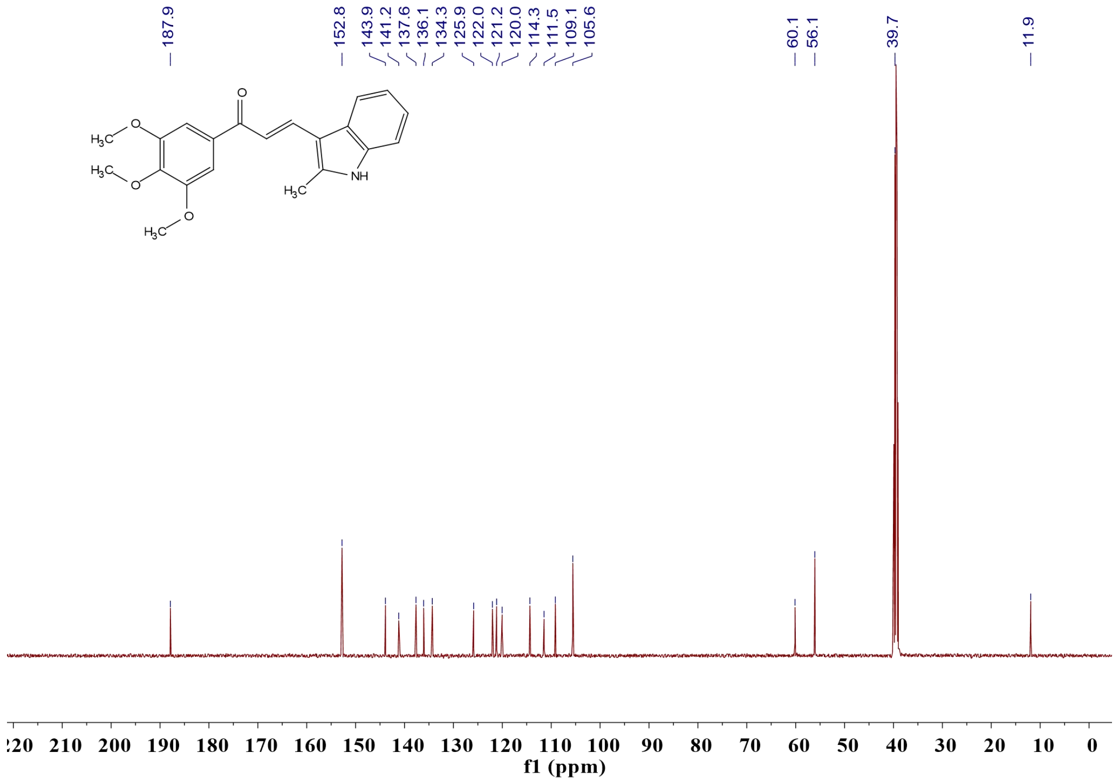

^{13}C NMR of **6b**

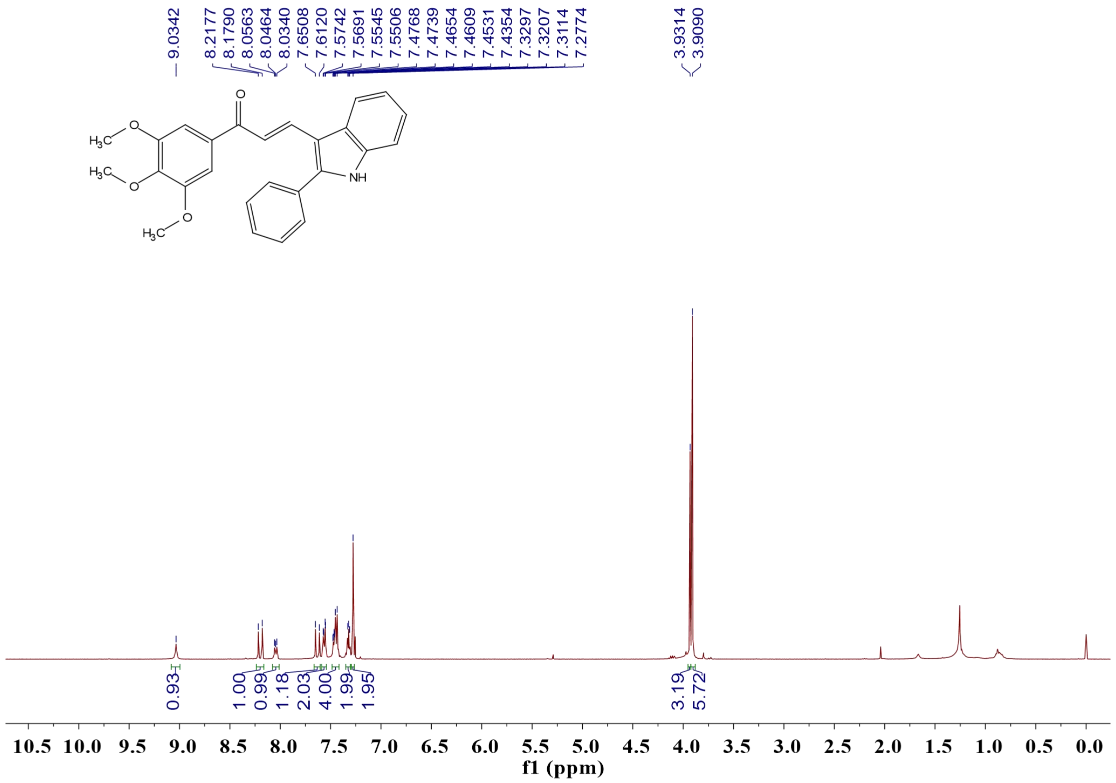

^{1}H NMR of **6c**

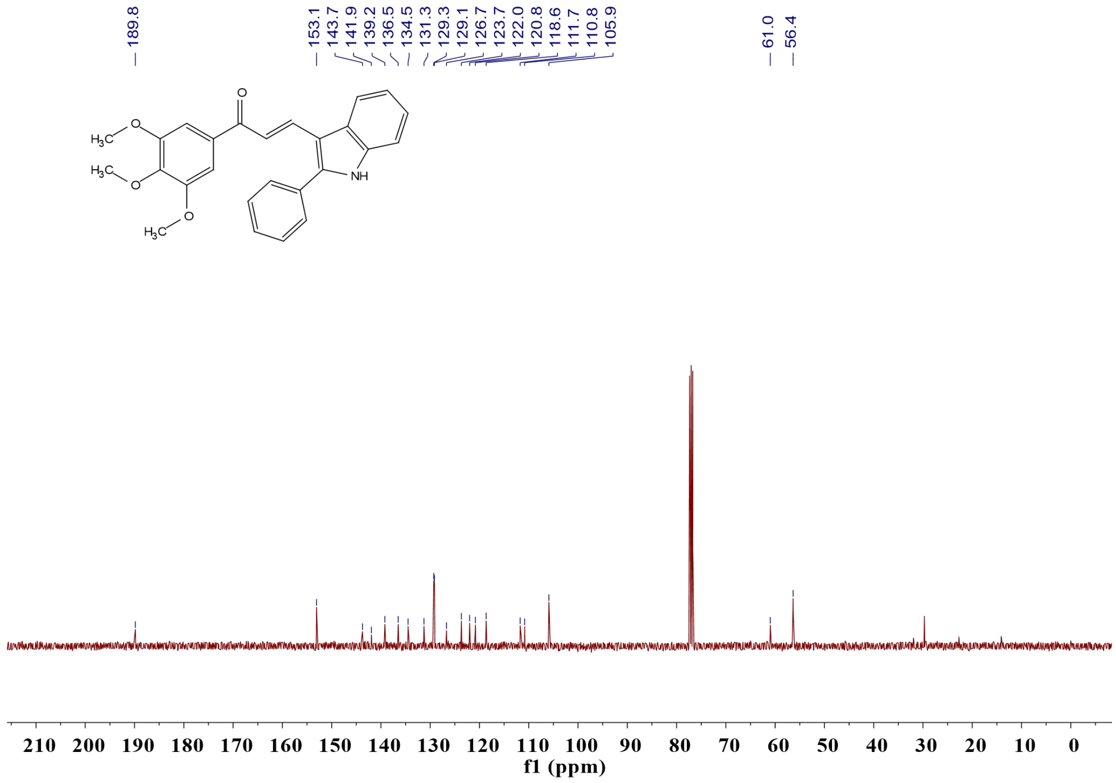

^{13}C NMR of **6c**

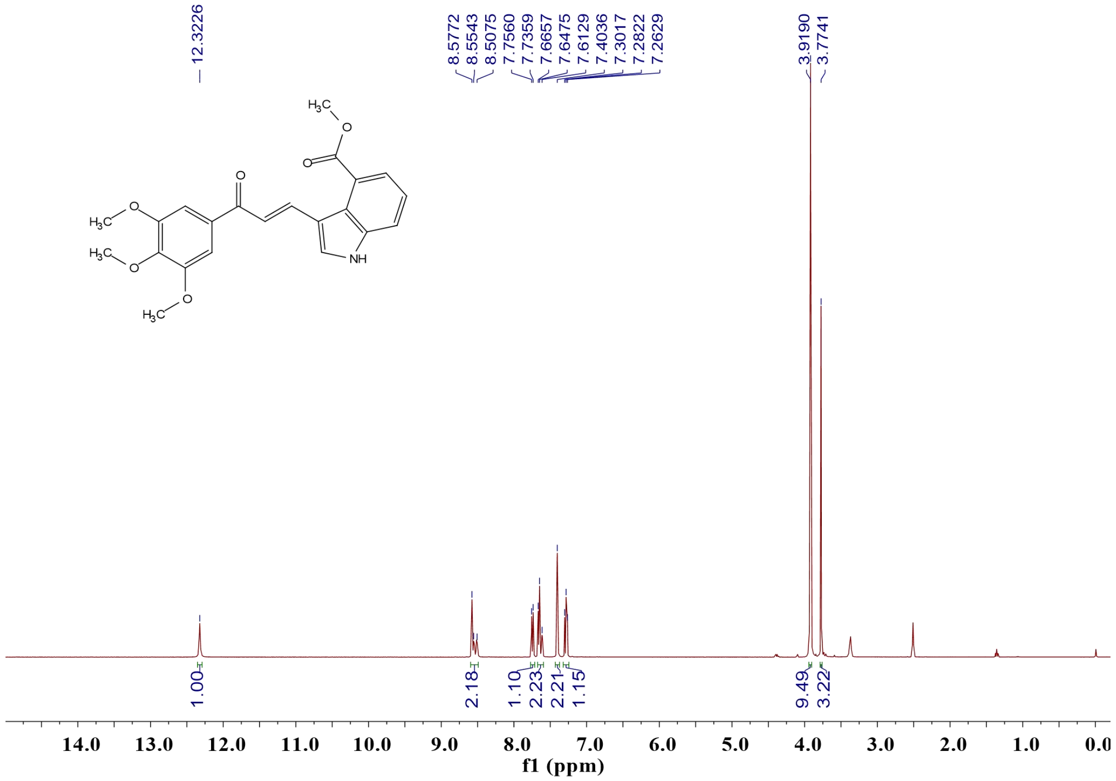

^{1}H NMR of **6d**

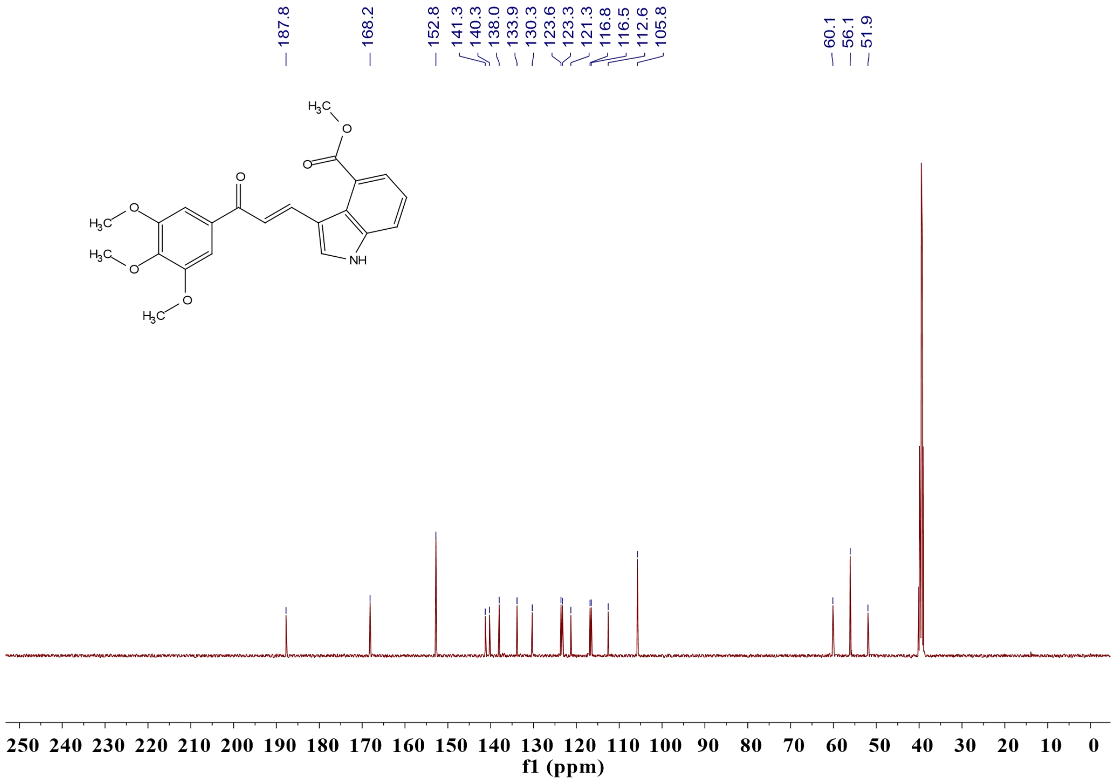

^{13}C NMR of **6d**

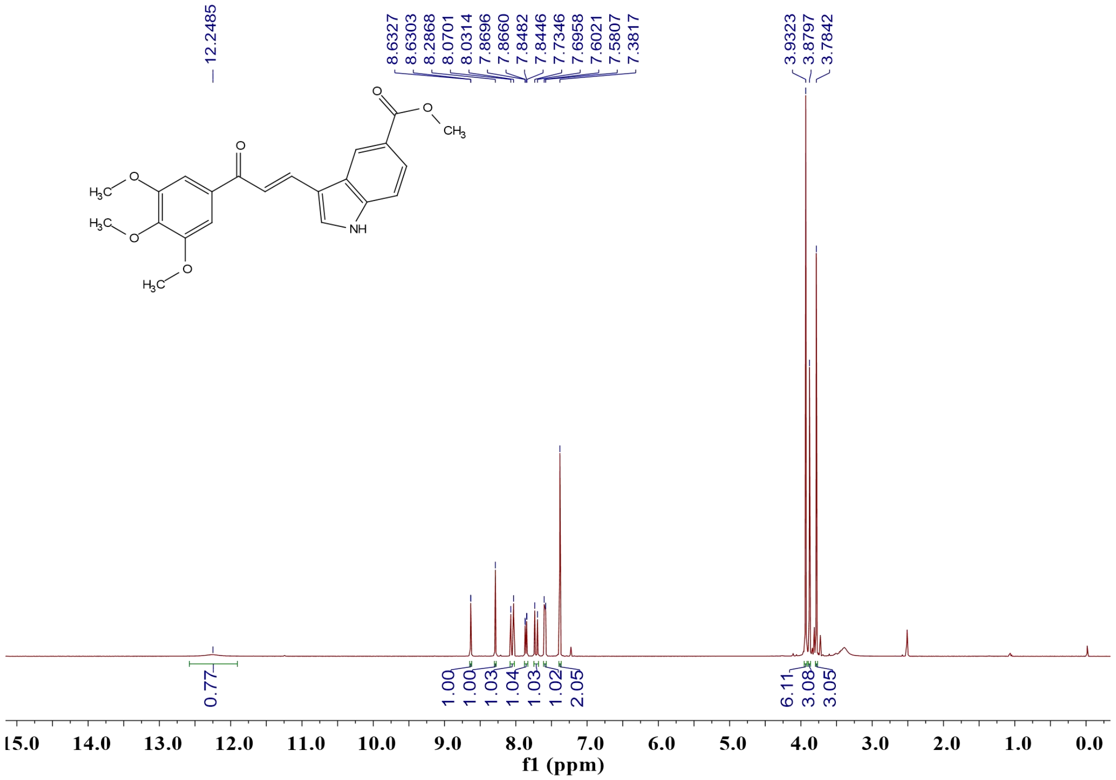

^{1}H NMR of **6e**

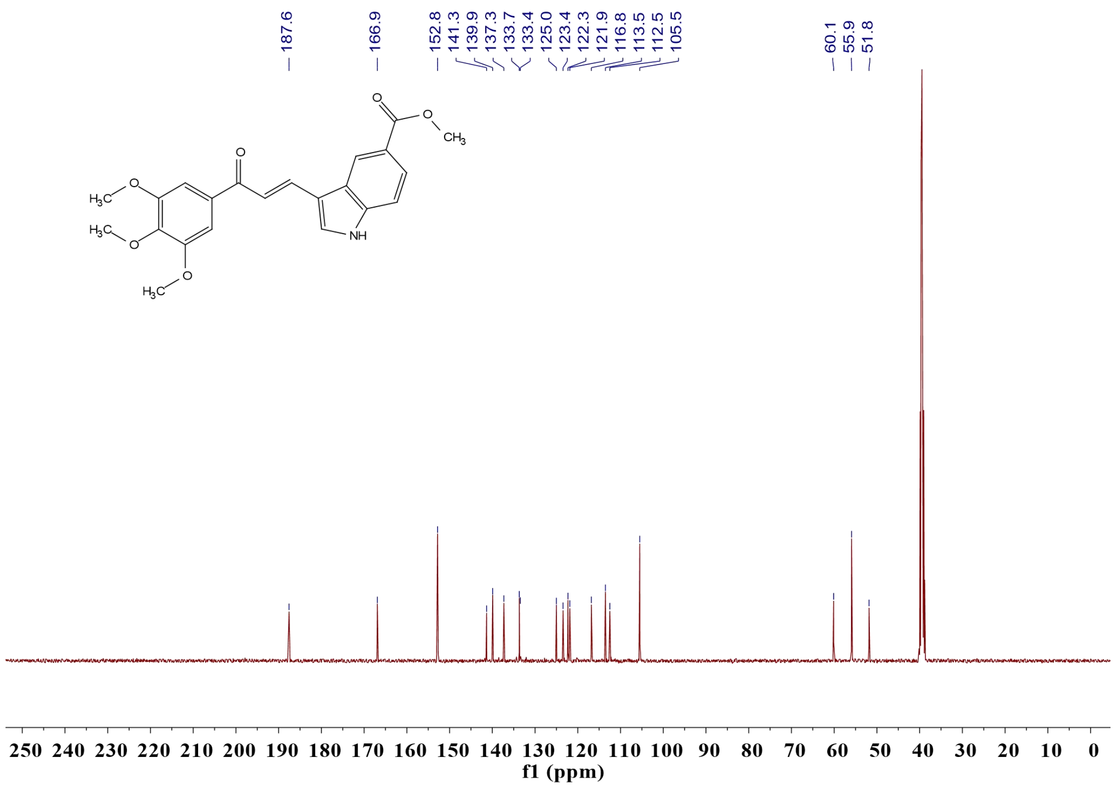

^{13}C NMR of **6e**

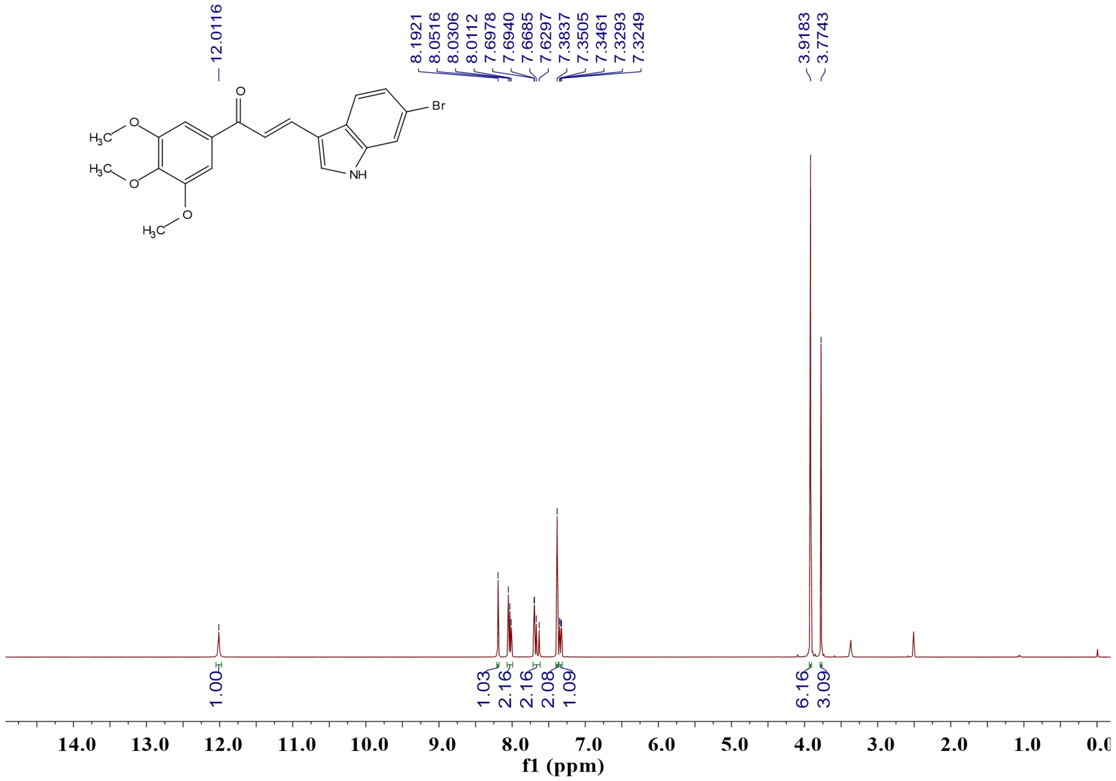

^{1}H NMR of **6g**

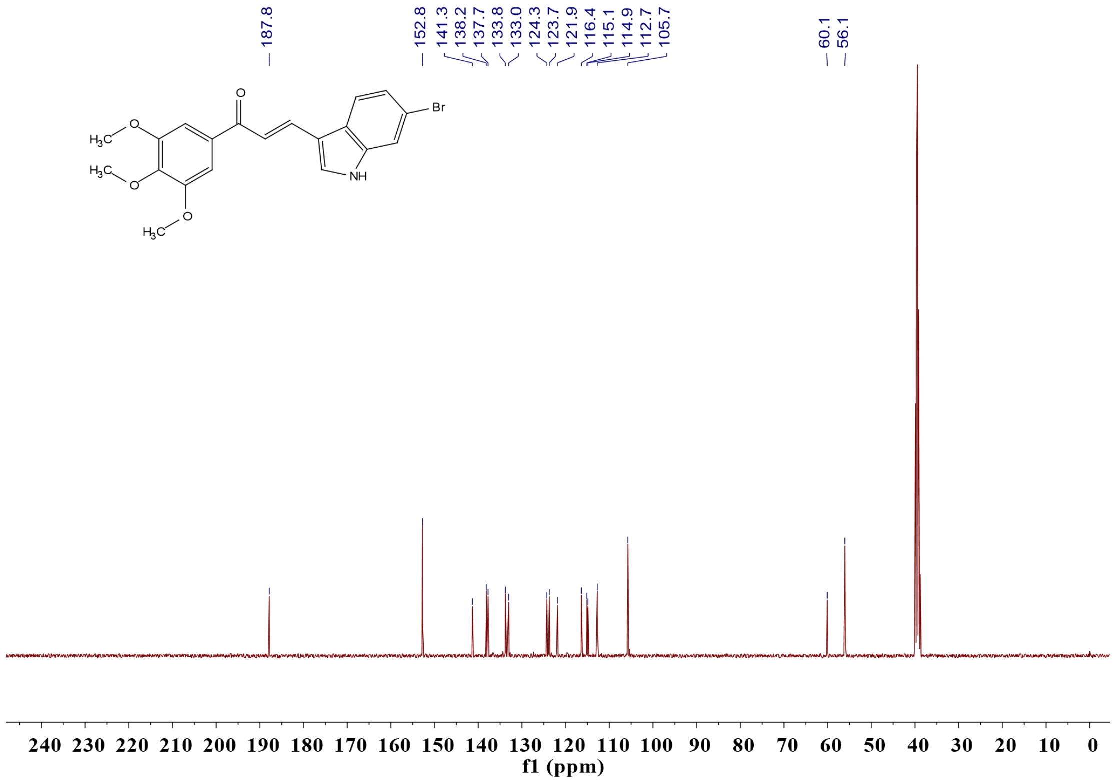

^{13}C NMR of **6g**

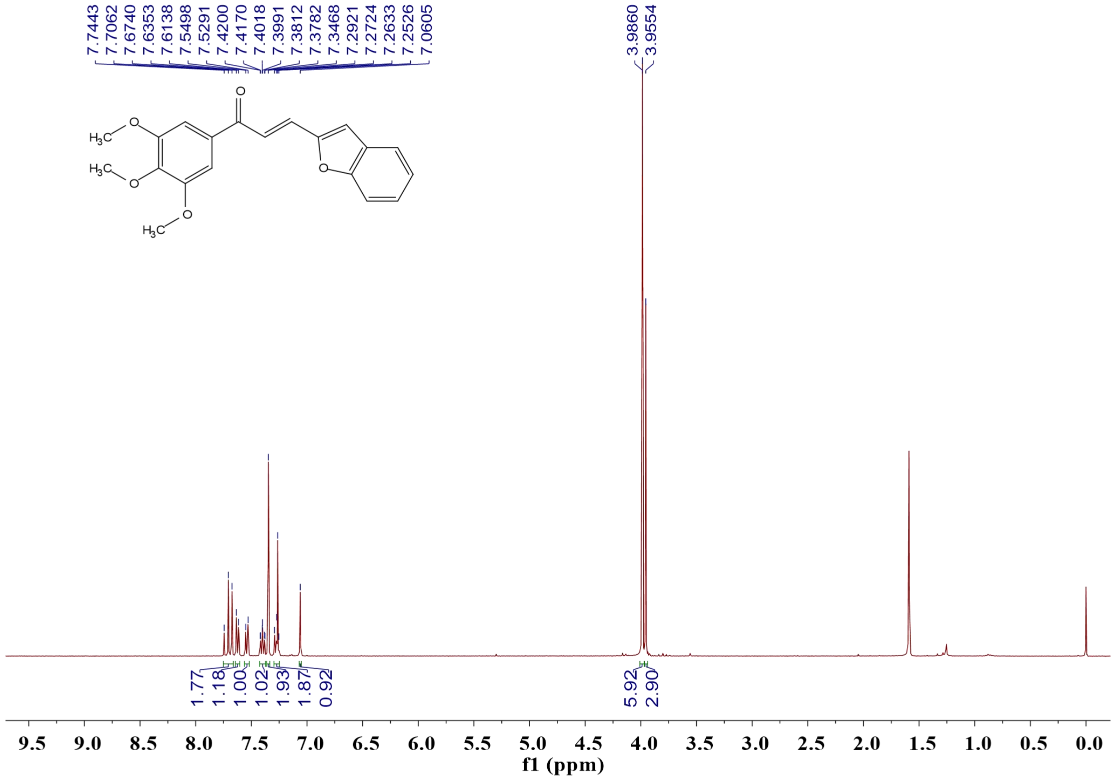

^{1}H NMR of **6h**

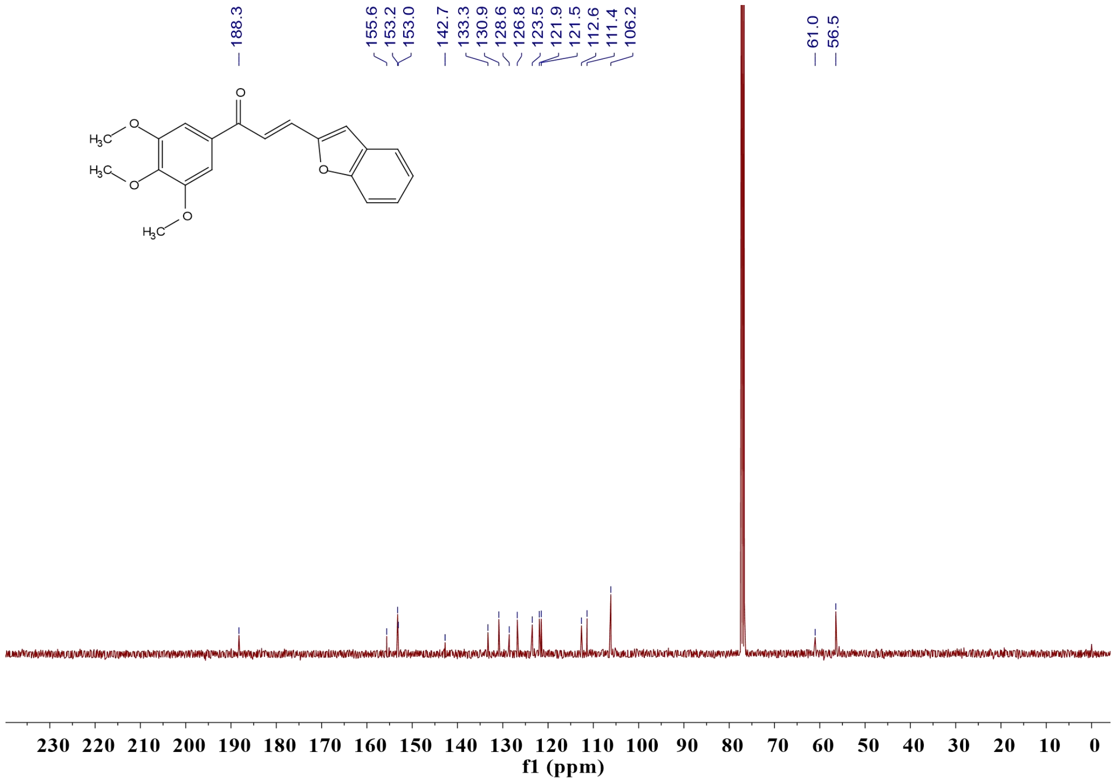

^{13}C NMR of **6h**

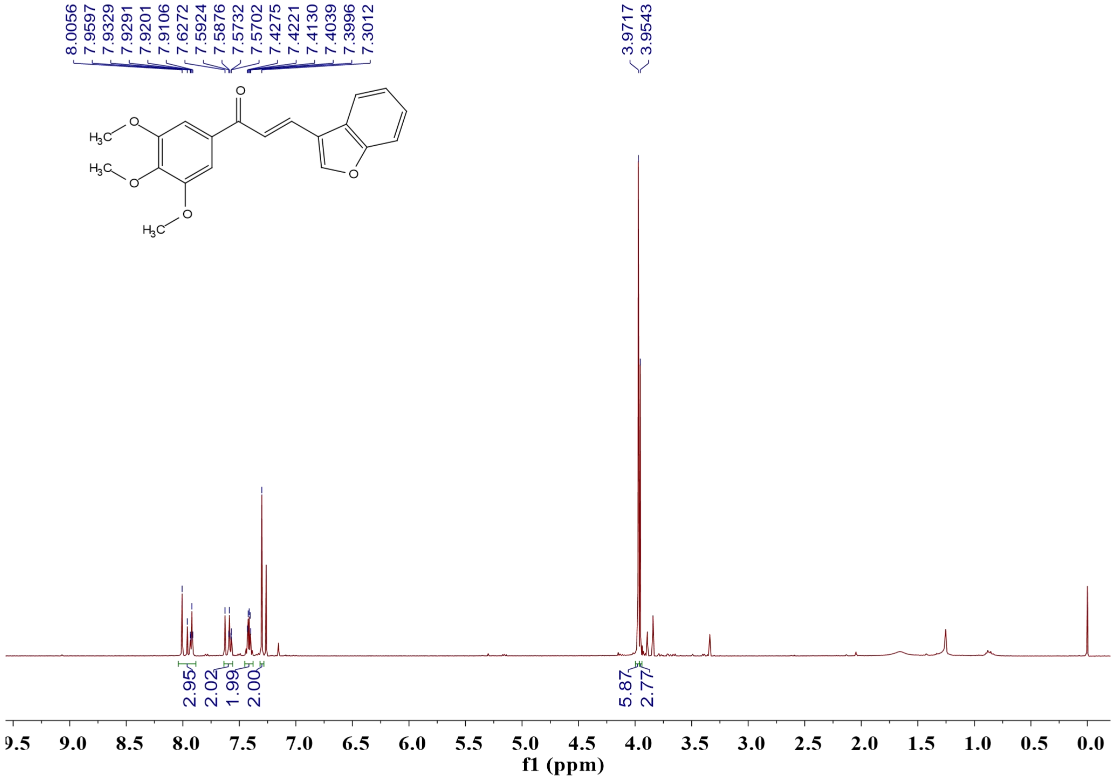

^{1}H NMR of **6j**

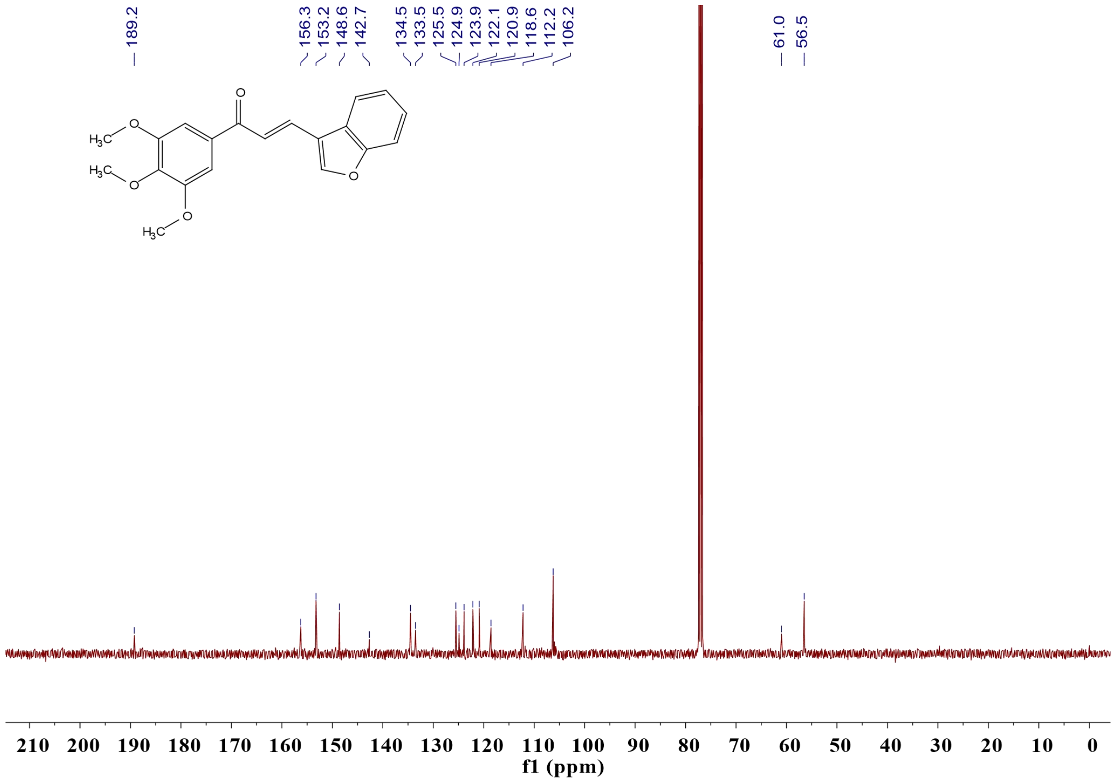

^{13}C NMR of **6j**

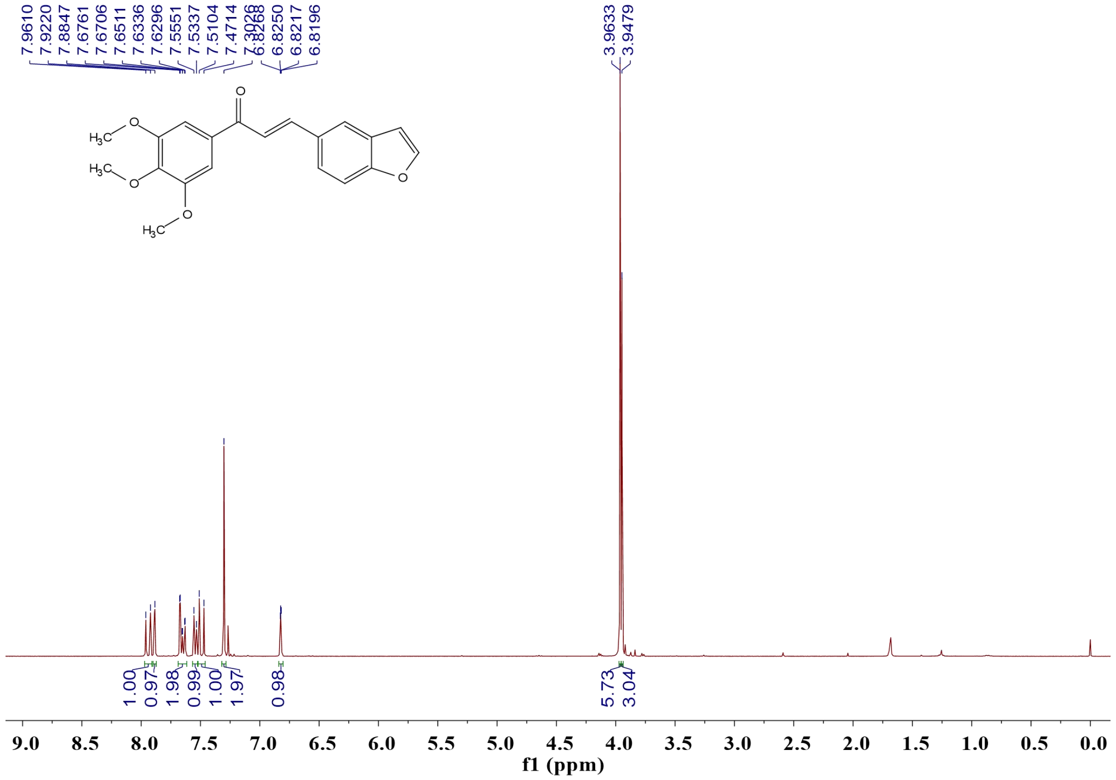

^{1}H NMR of **6m**

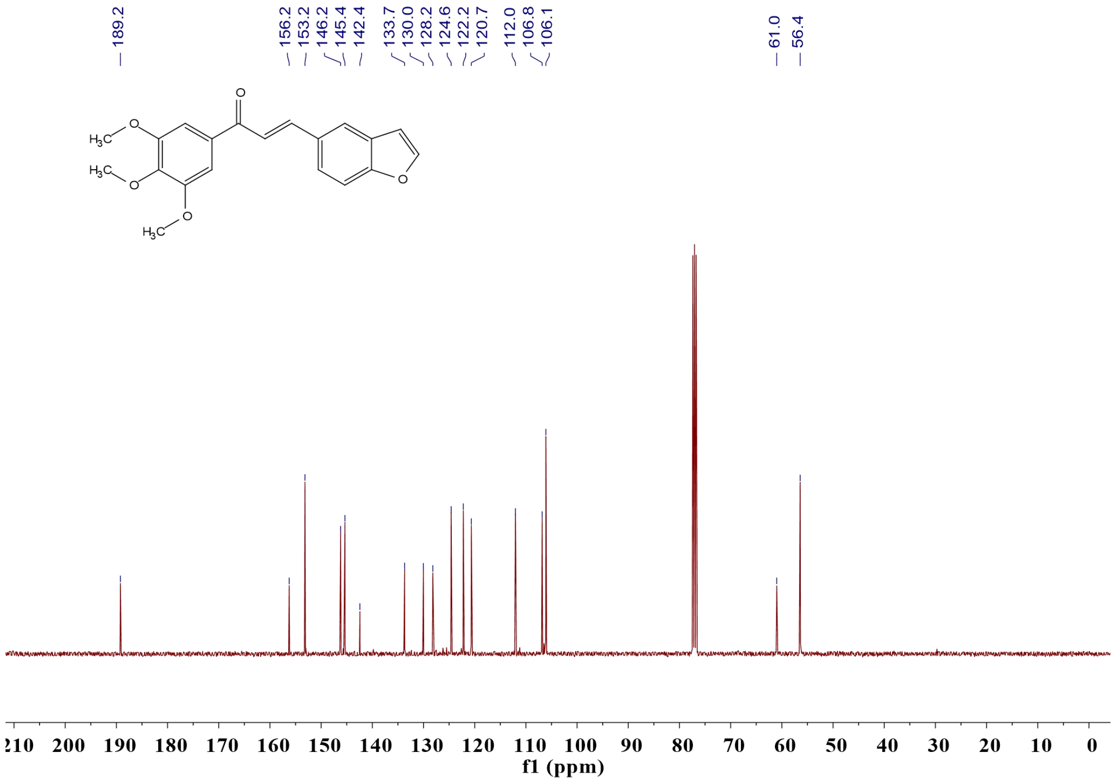

^{13}C NMR of **6m**

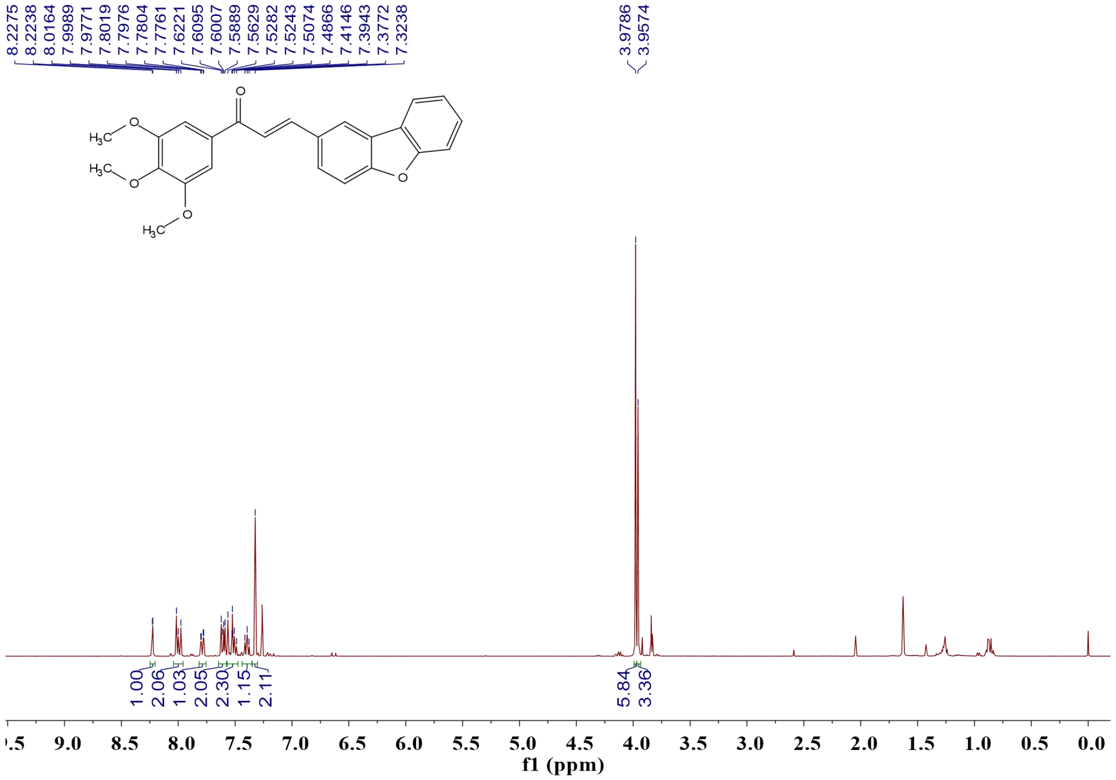

^{1}H NMR of **6l**

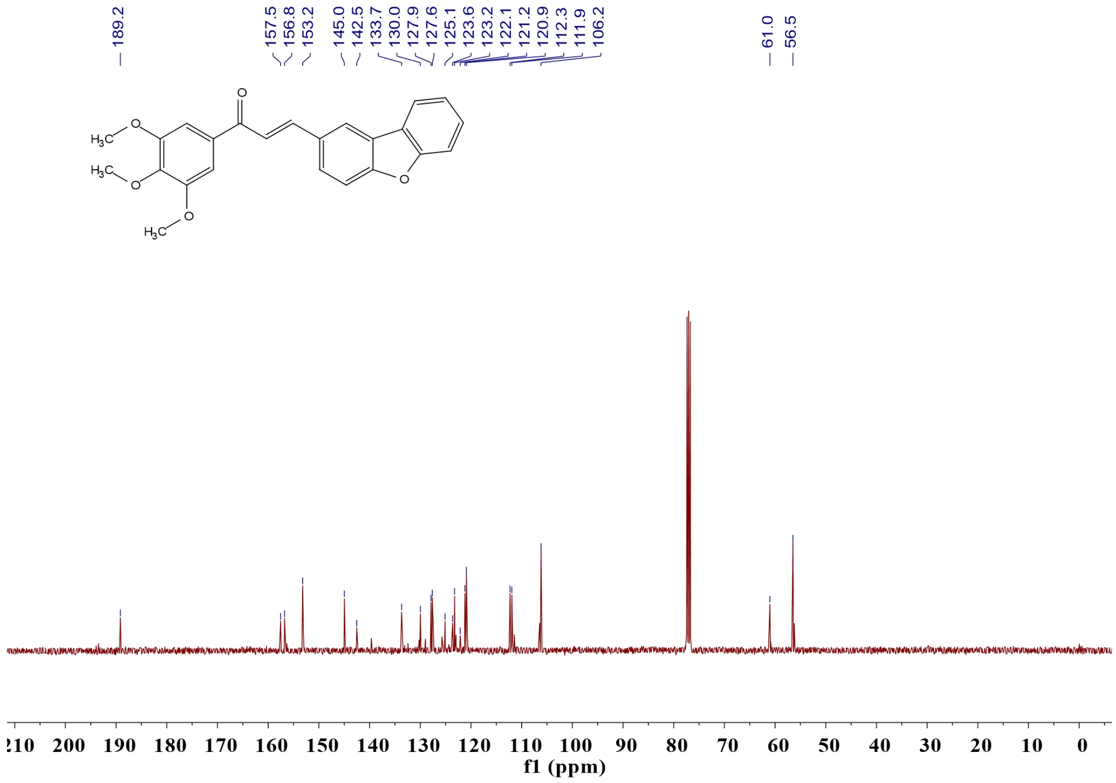

^{13}C NMR of **6l**

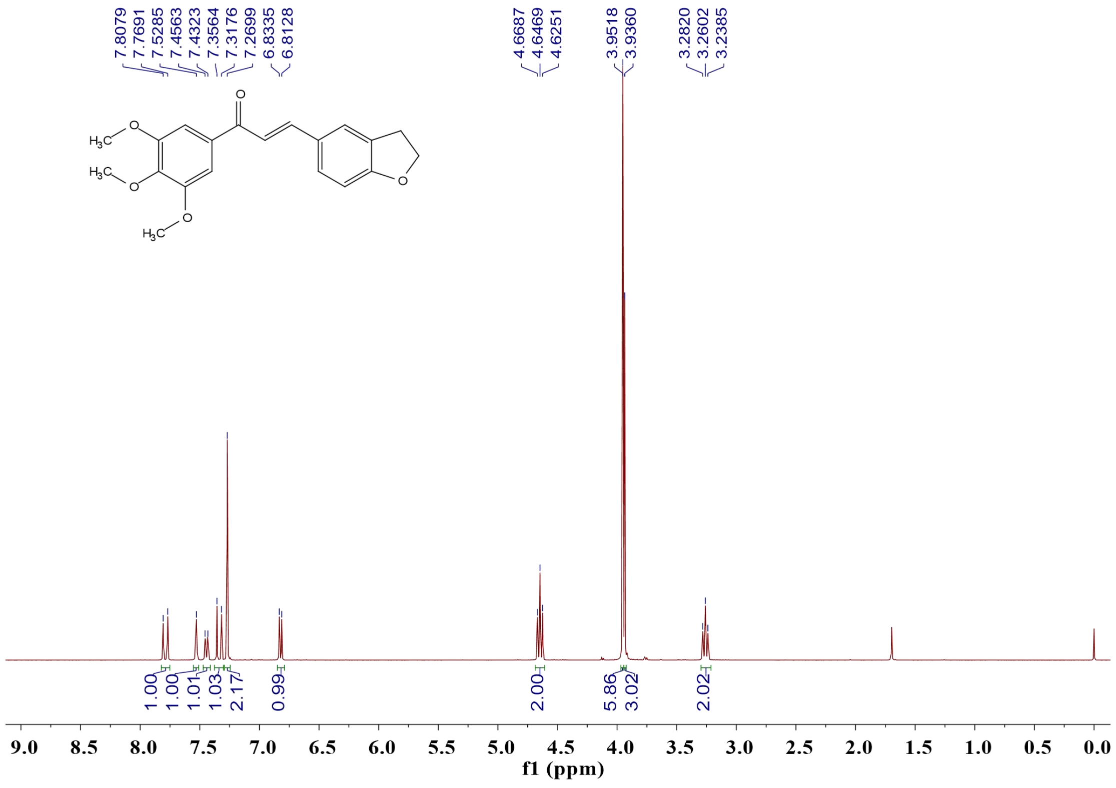

^{1}H NMR of **6n**

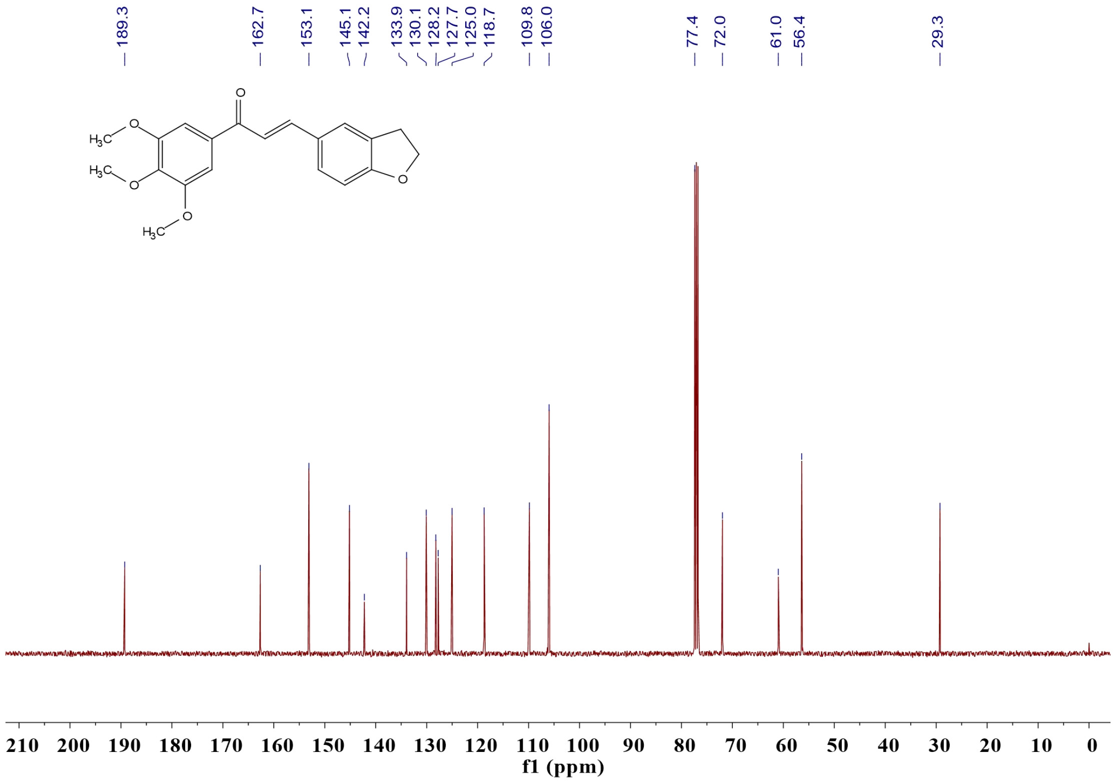

^{13}C NMR of **6n**

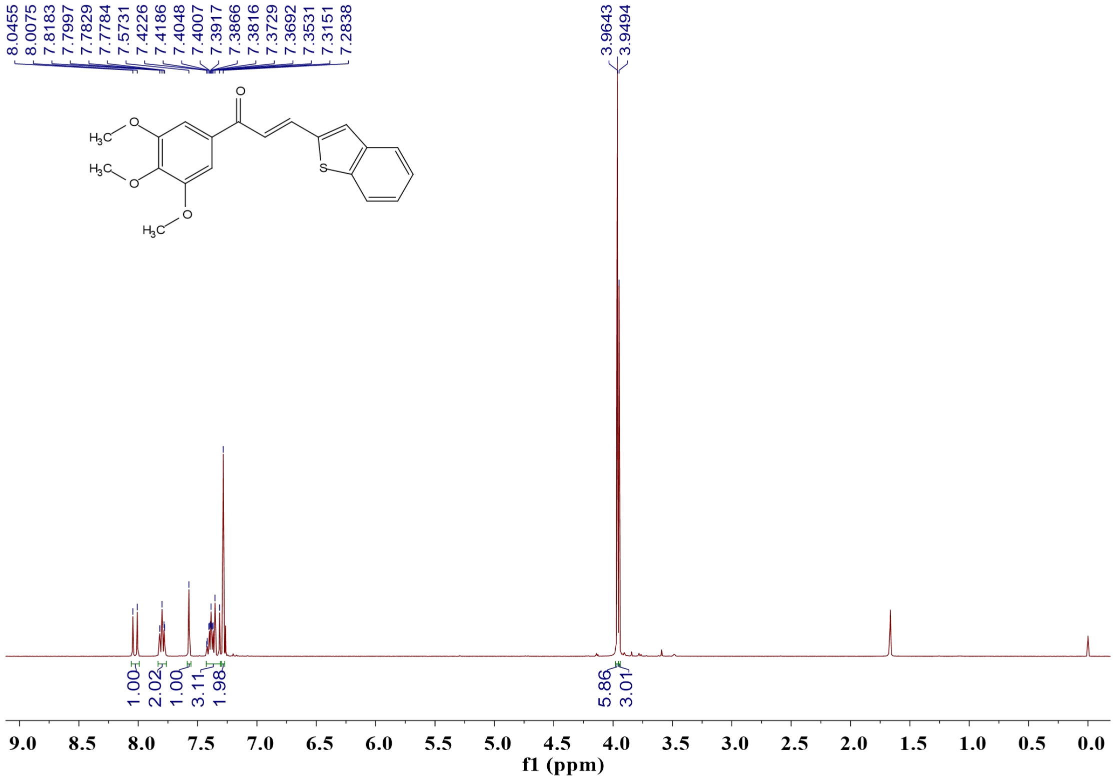

^{1}H NMR of **6o**

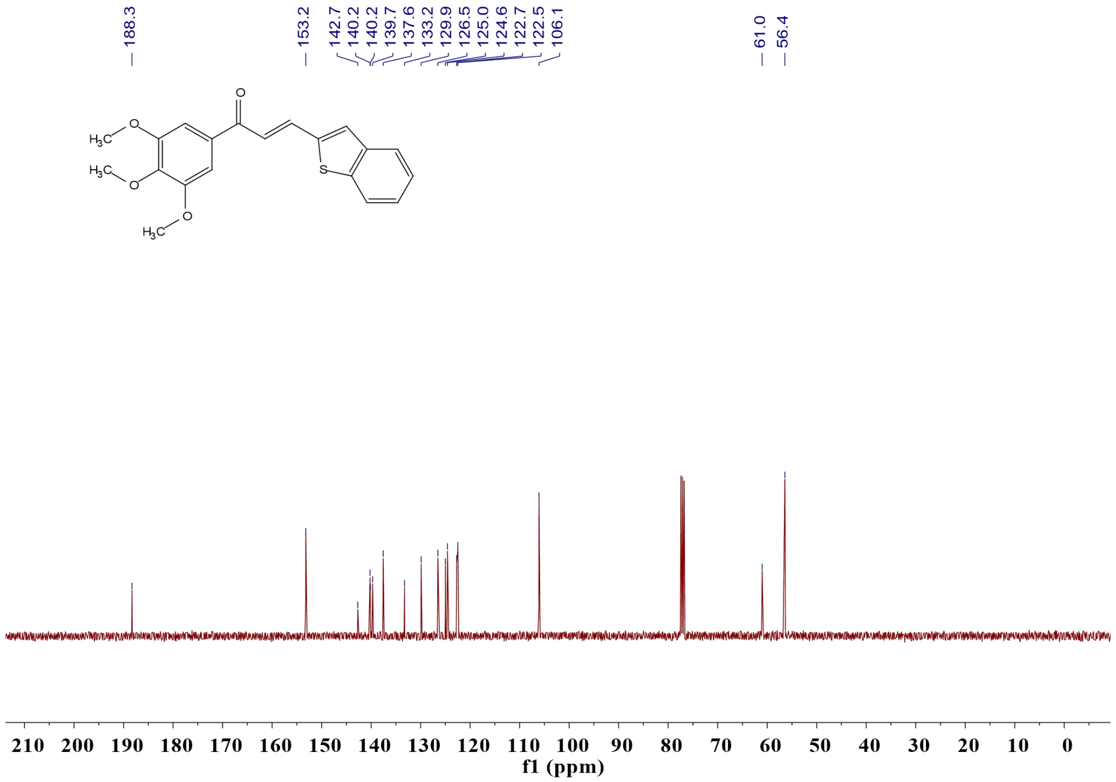

^{13}C NMR of **6o**

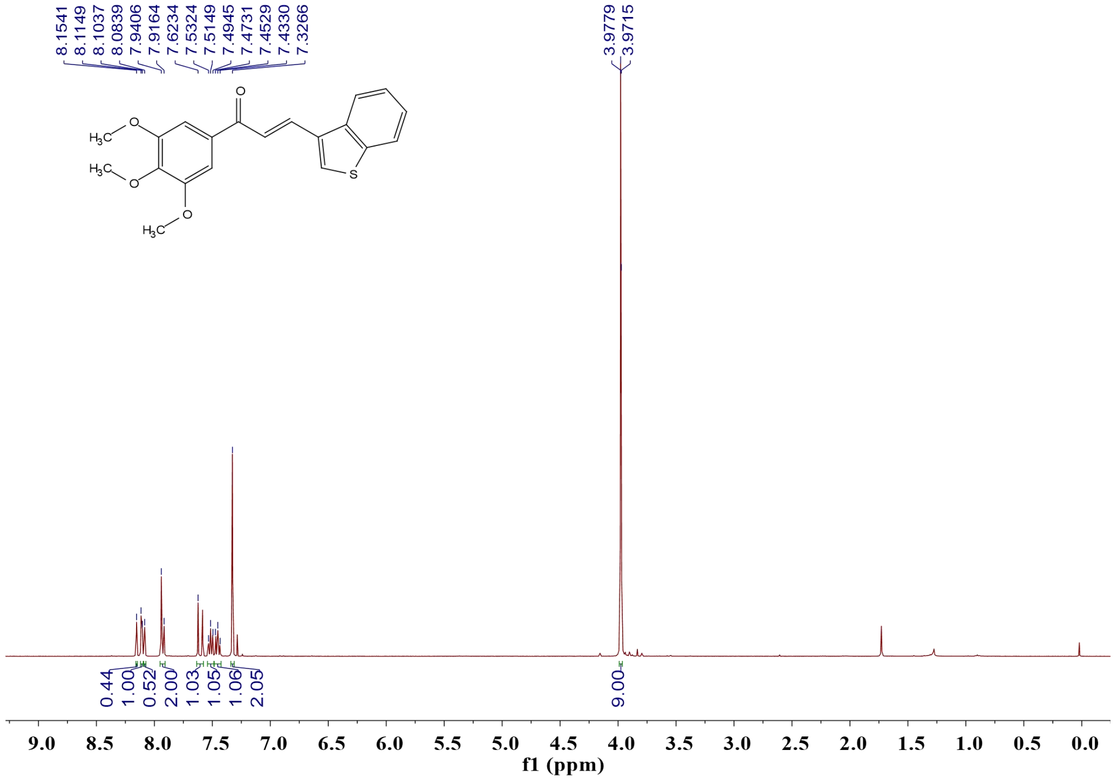

^{1}H NMR of **6p**

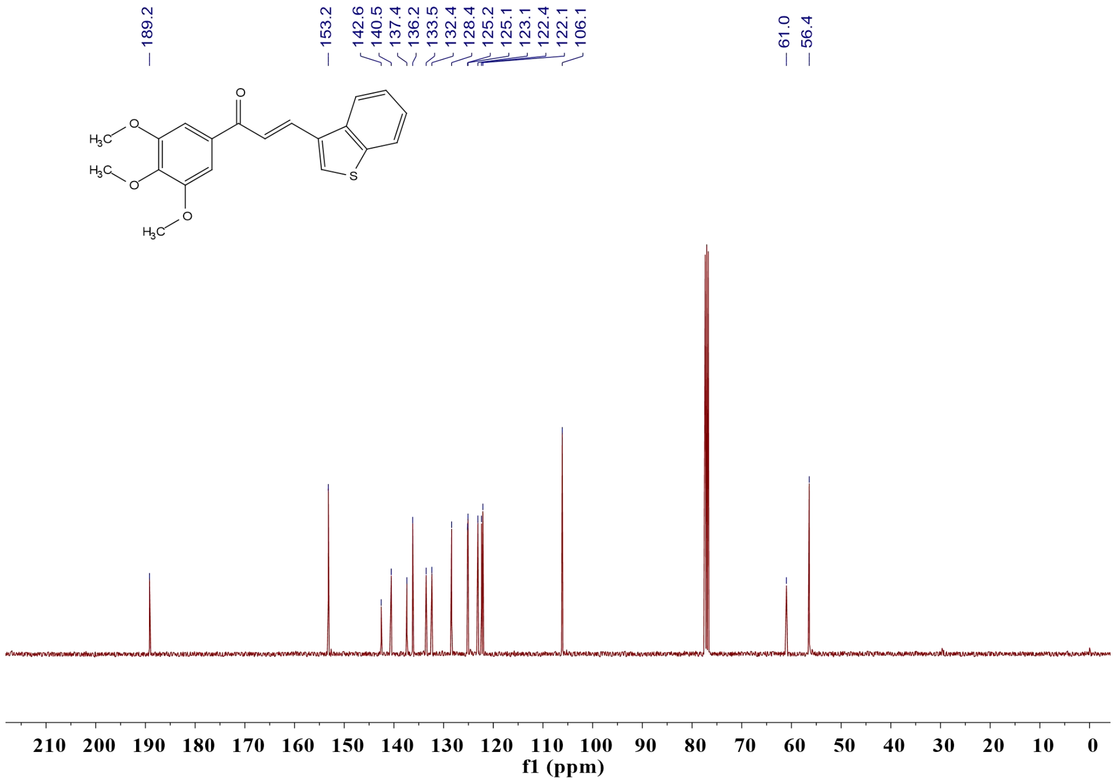

^{13}C NMR of **6p**

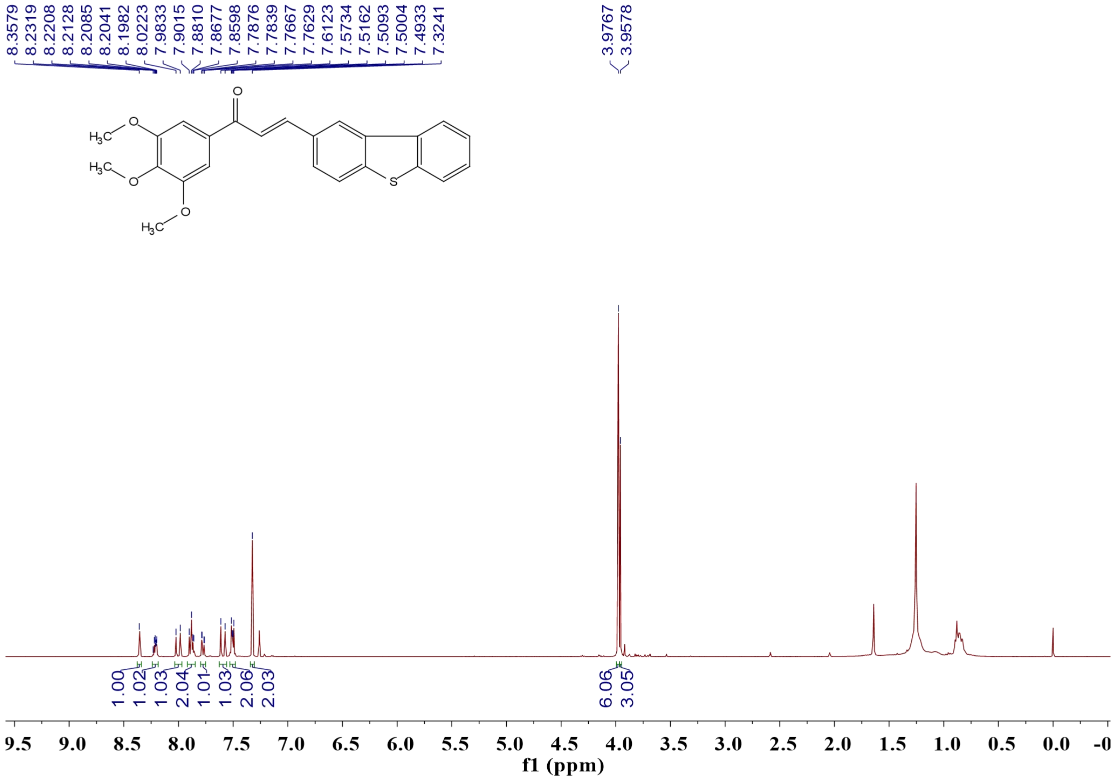

^{1}H NMR of **6q**

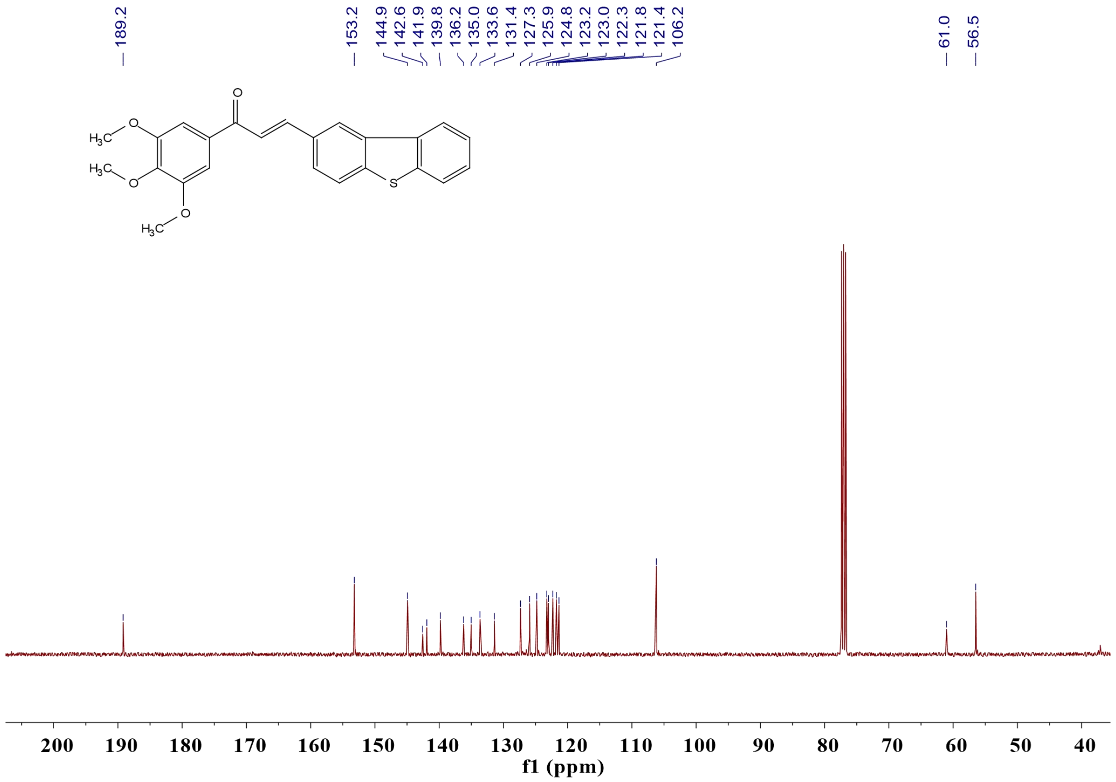

^{13}C NMR of **6q**

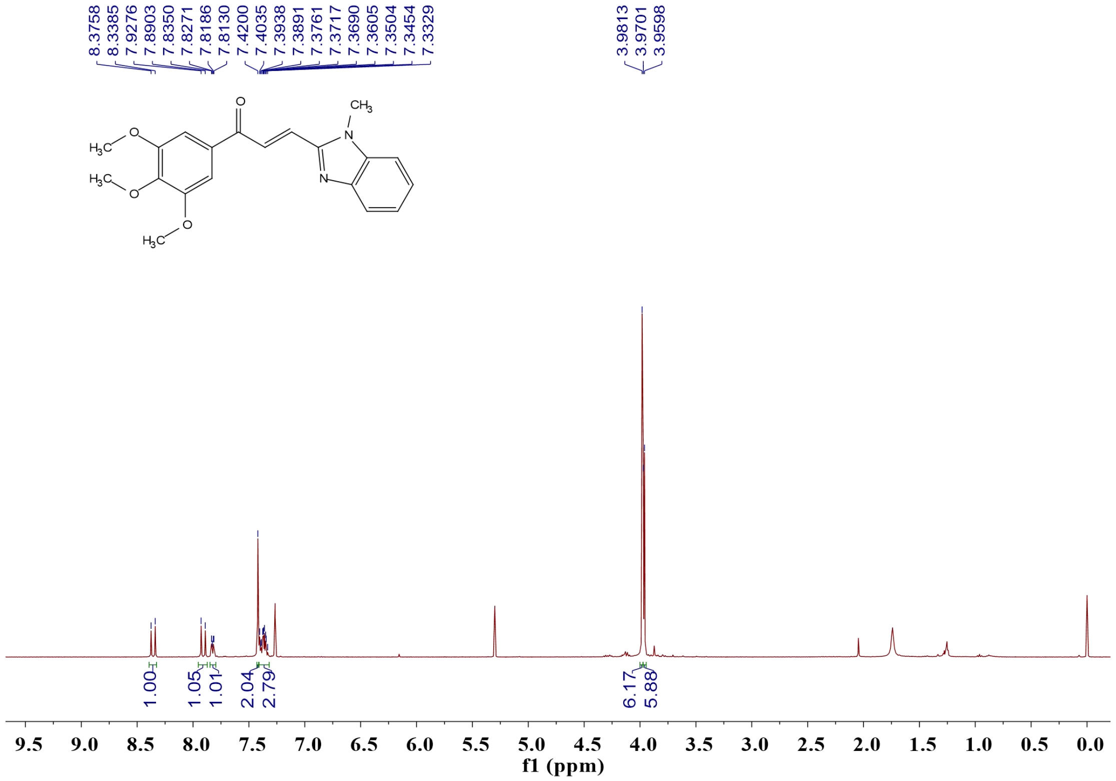

^{1}H NMR of **6r**

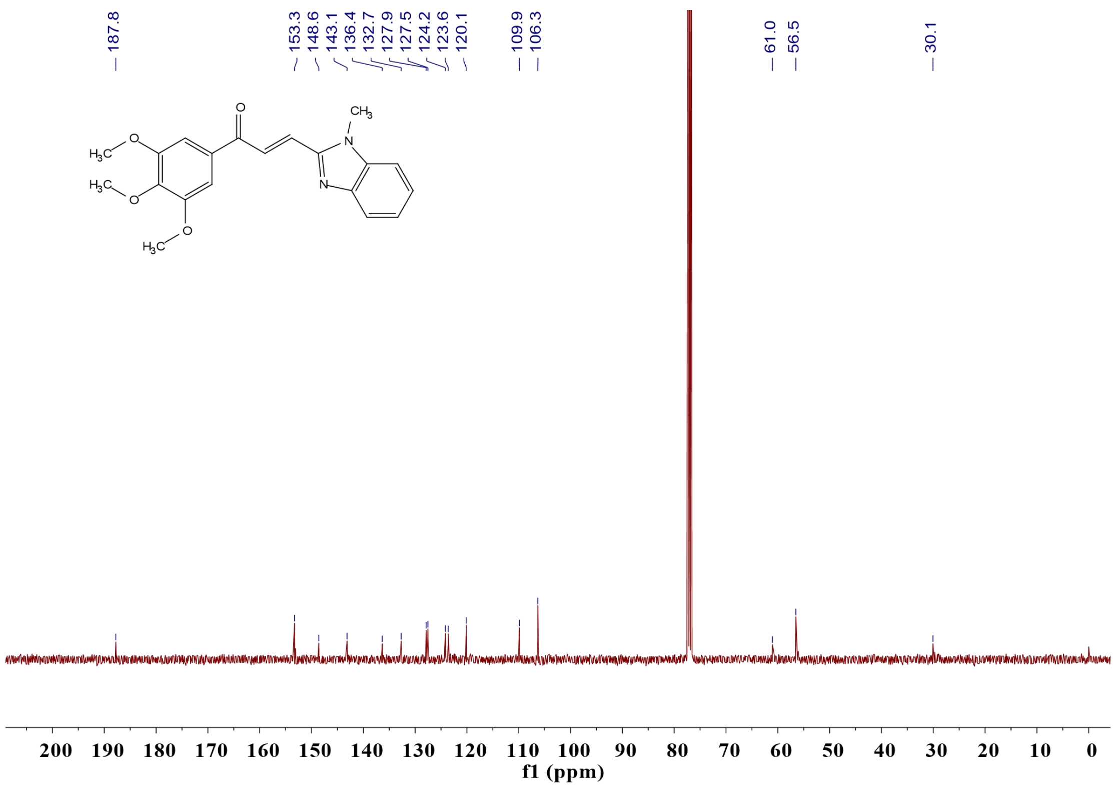

^{13}C NMR of **6r**

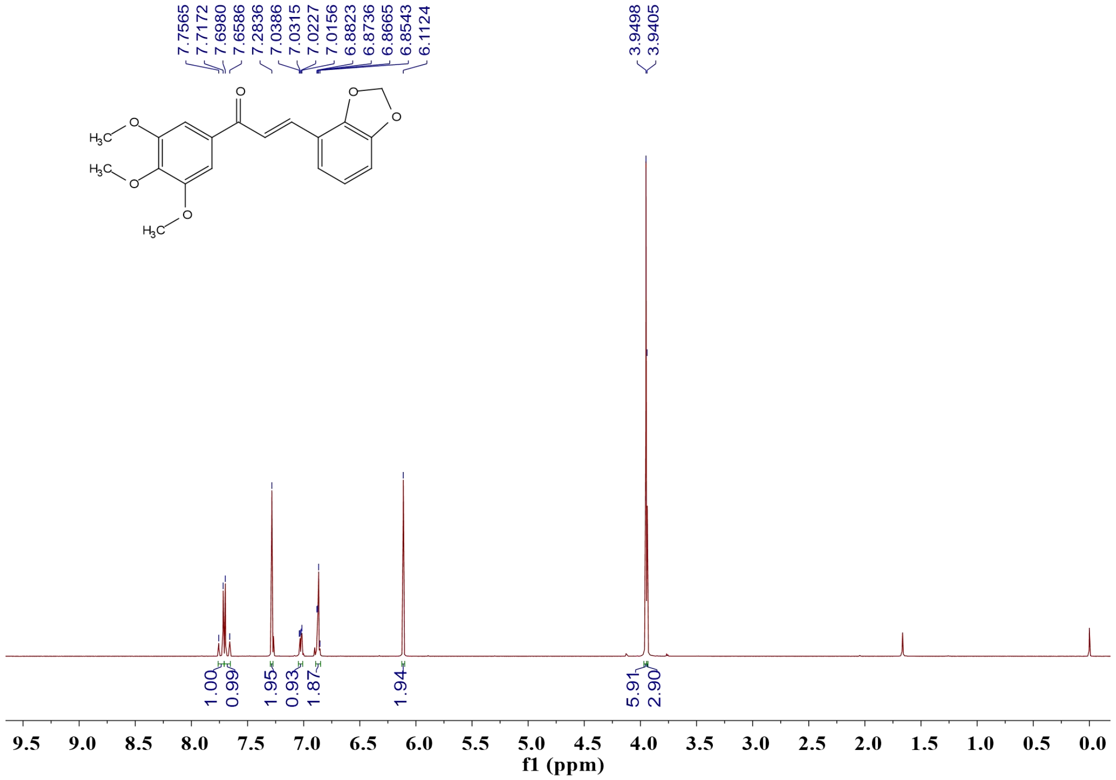

^{1}H NMR of **6s**

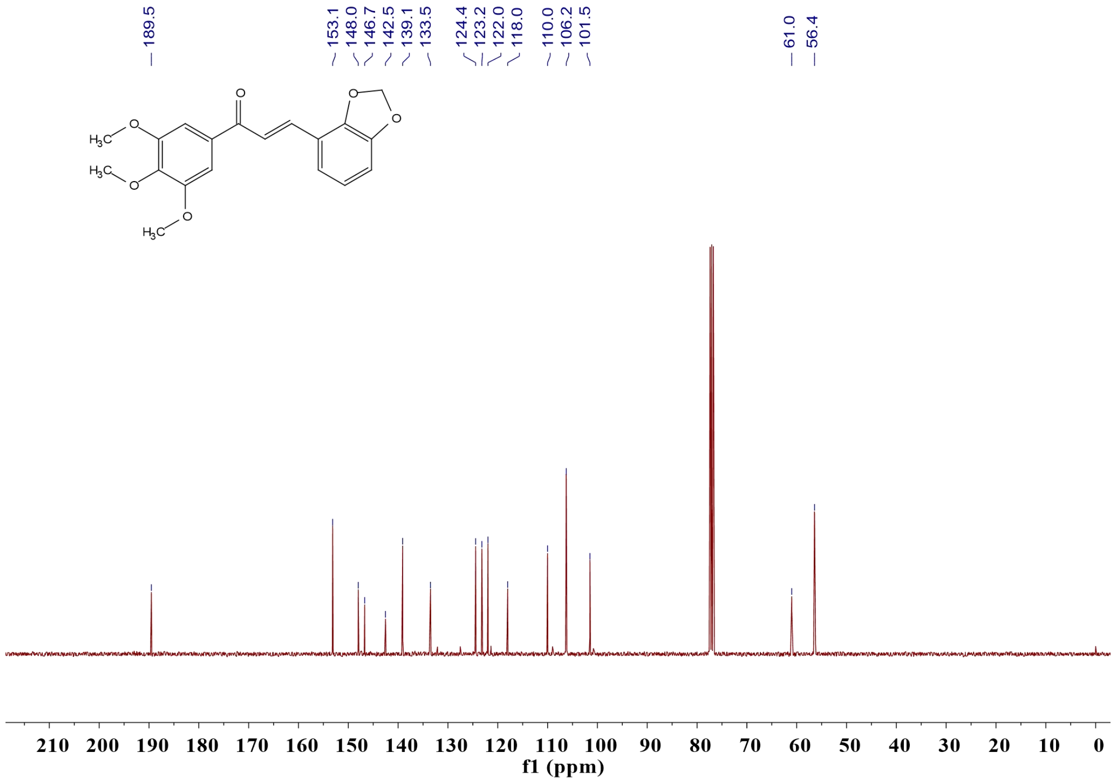

^{13}C NMR of **6s**

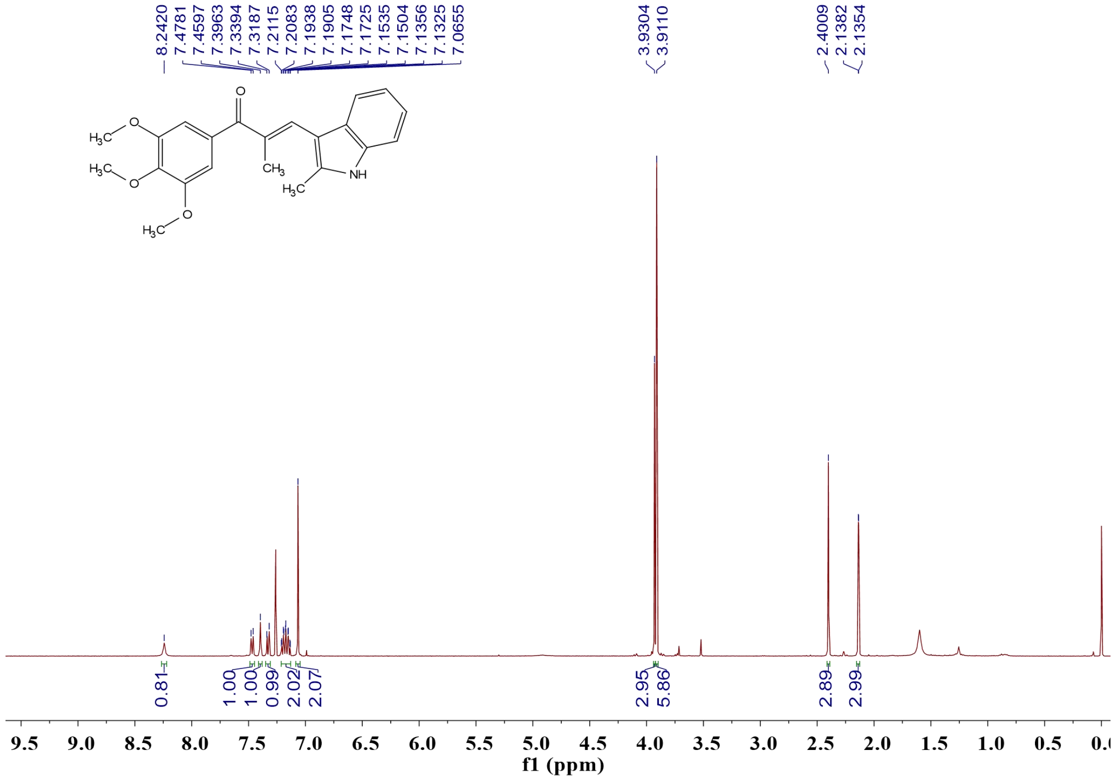

^{1}H NMR of **7b**

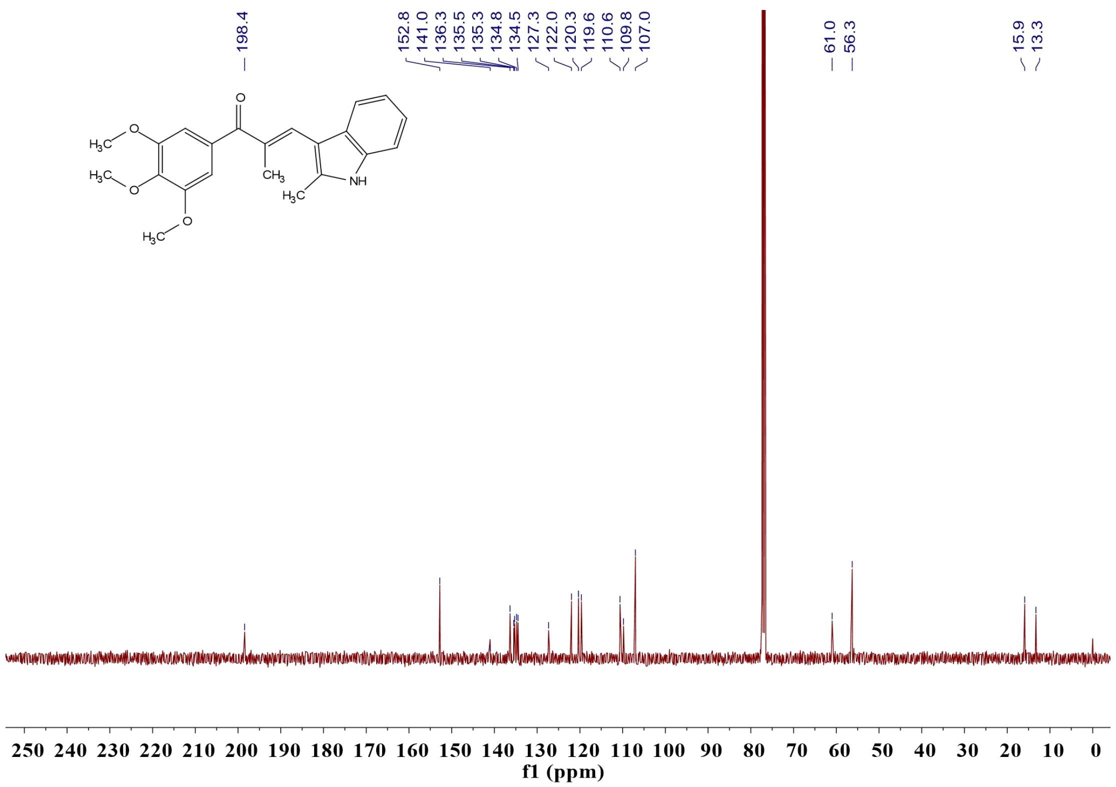

^{13}C NMR of **7b**

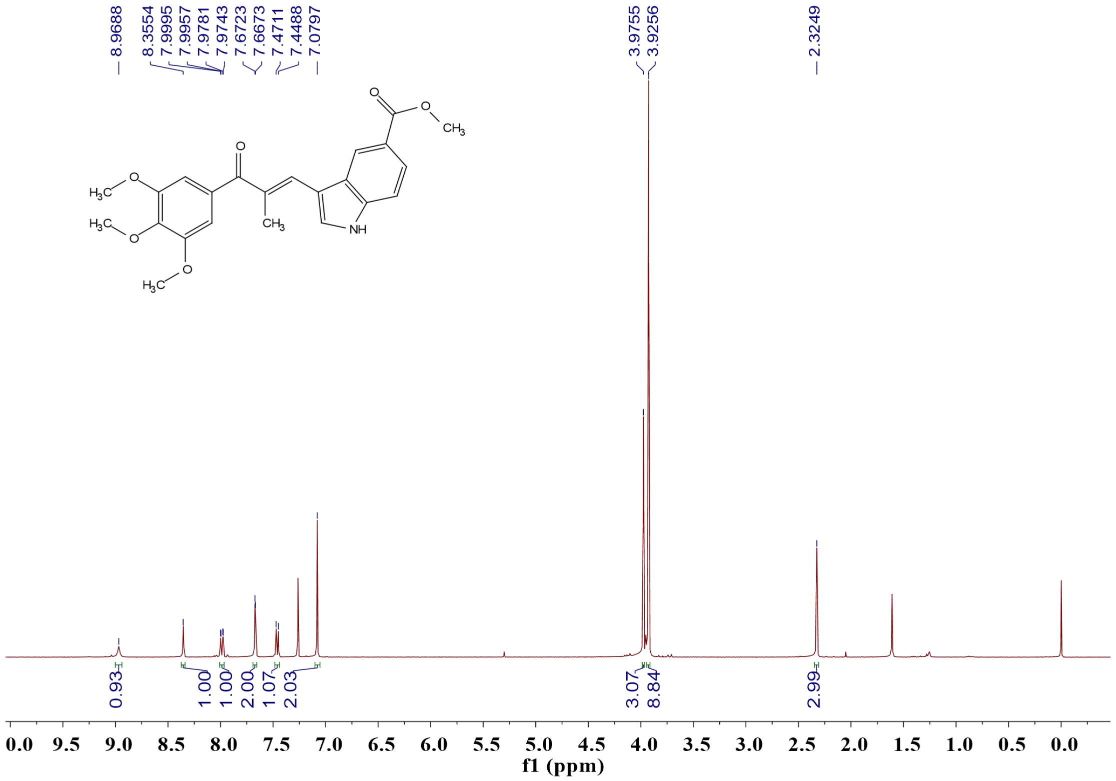

^{1}H NMR of **7e**

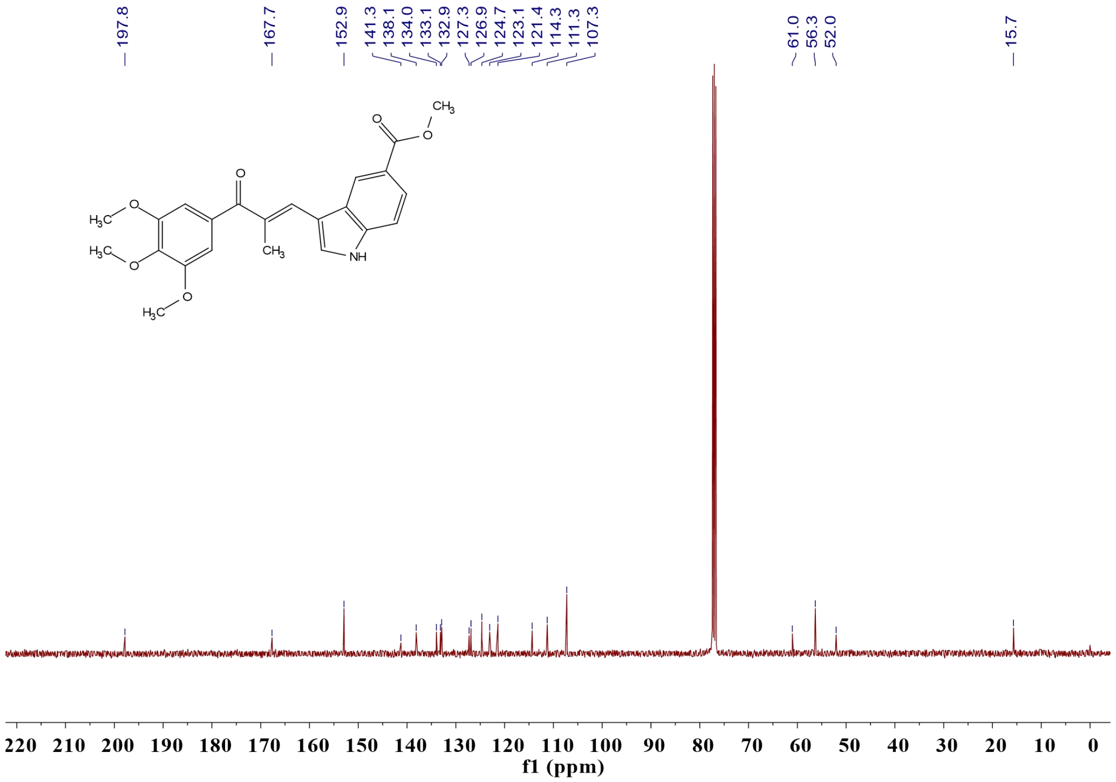

^{13}C NMR of **7e**

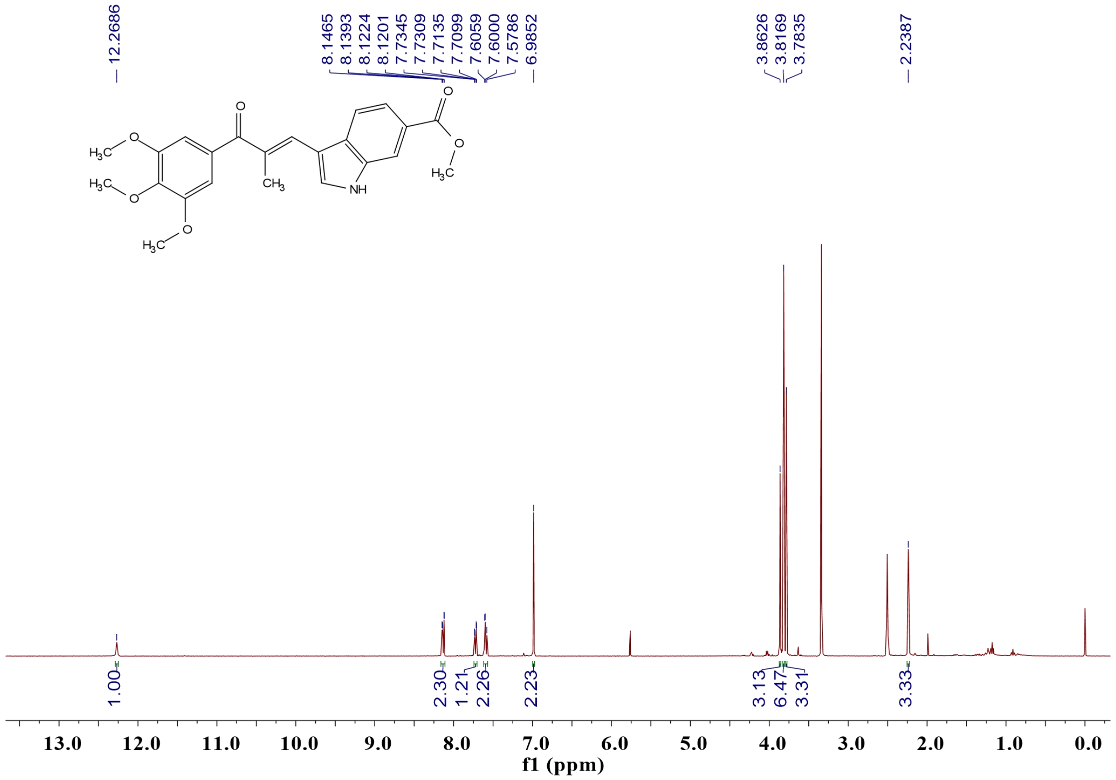

^{1}H NMR of **7f**

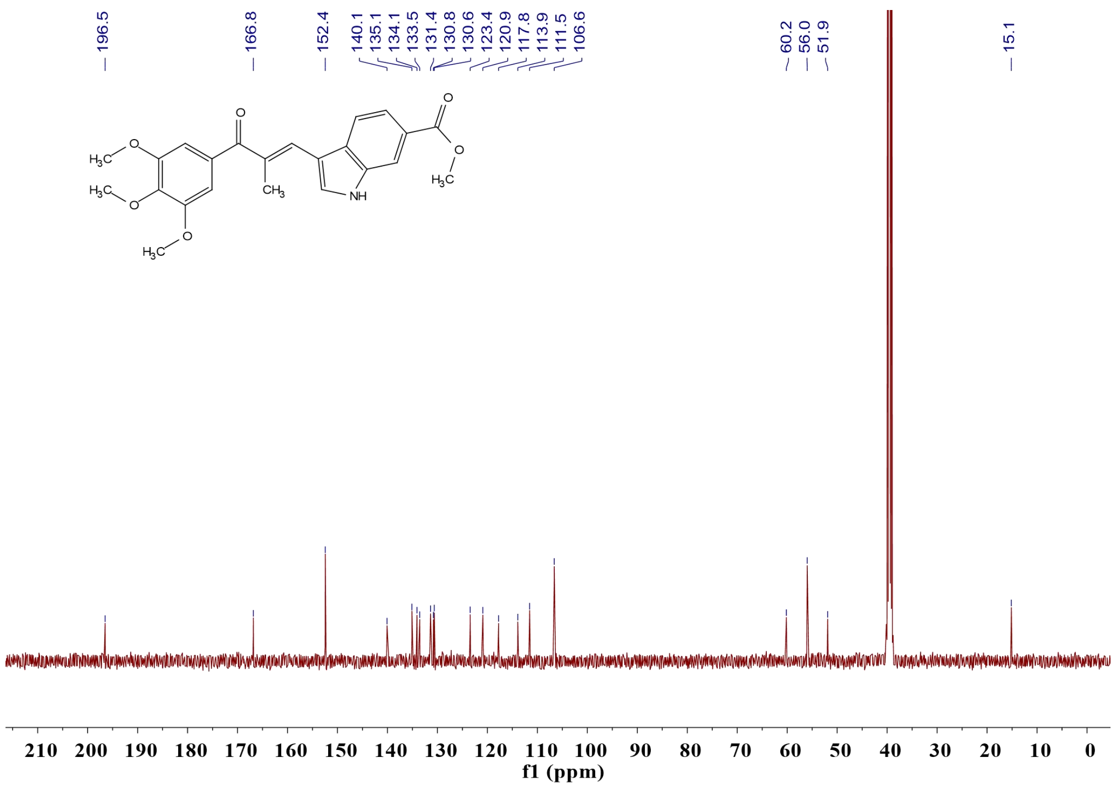

^{13}C NMR of **7f**

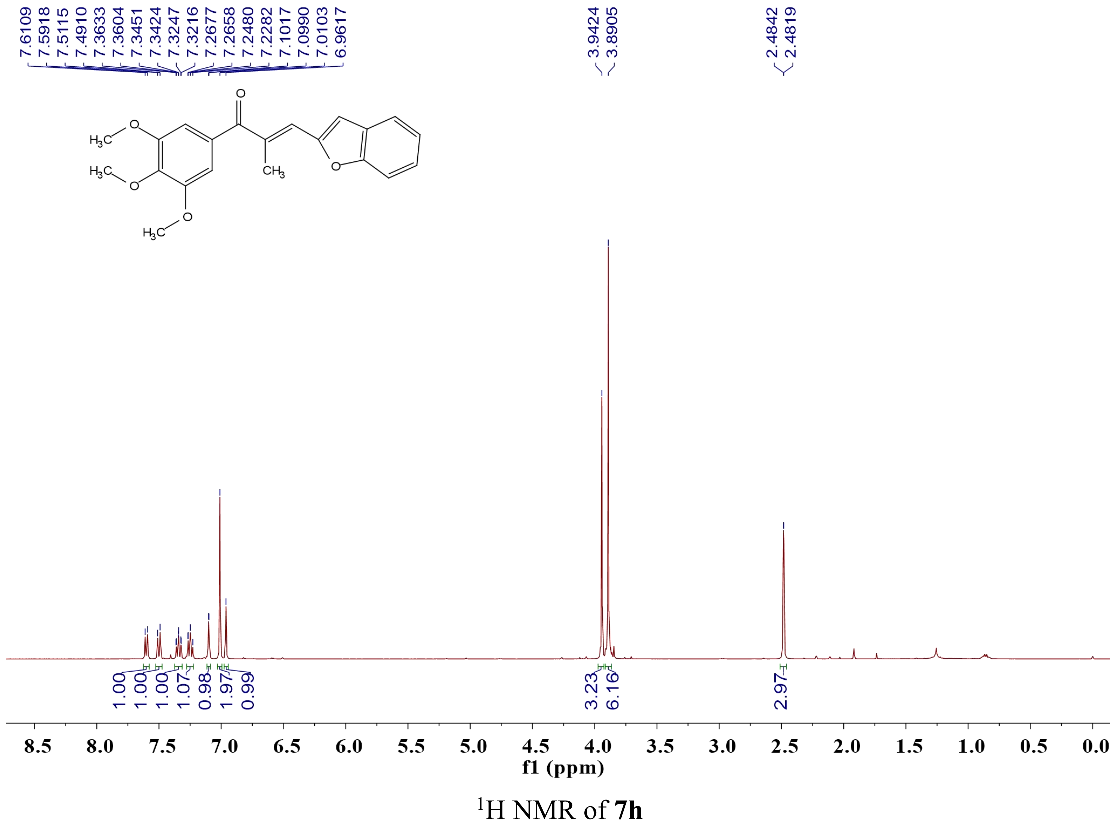

^{1}H NMR of **7h**

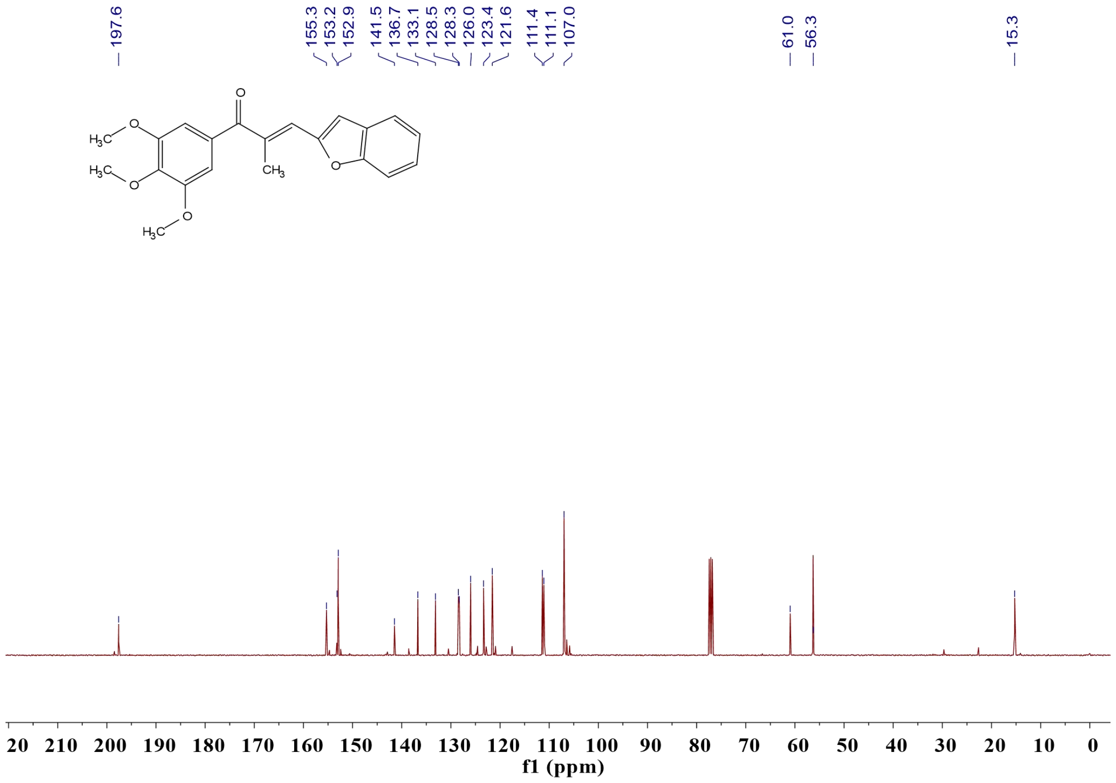

^{13}C NMR of **7h**

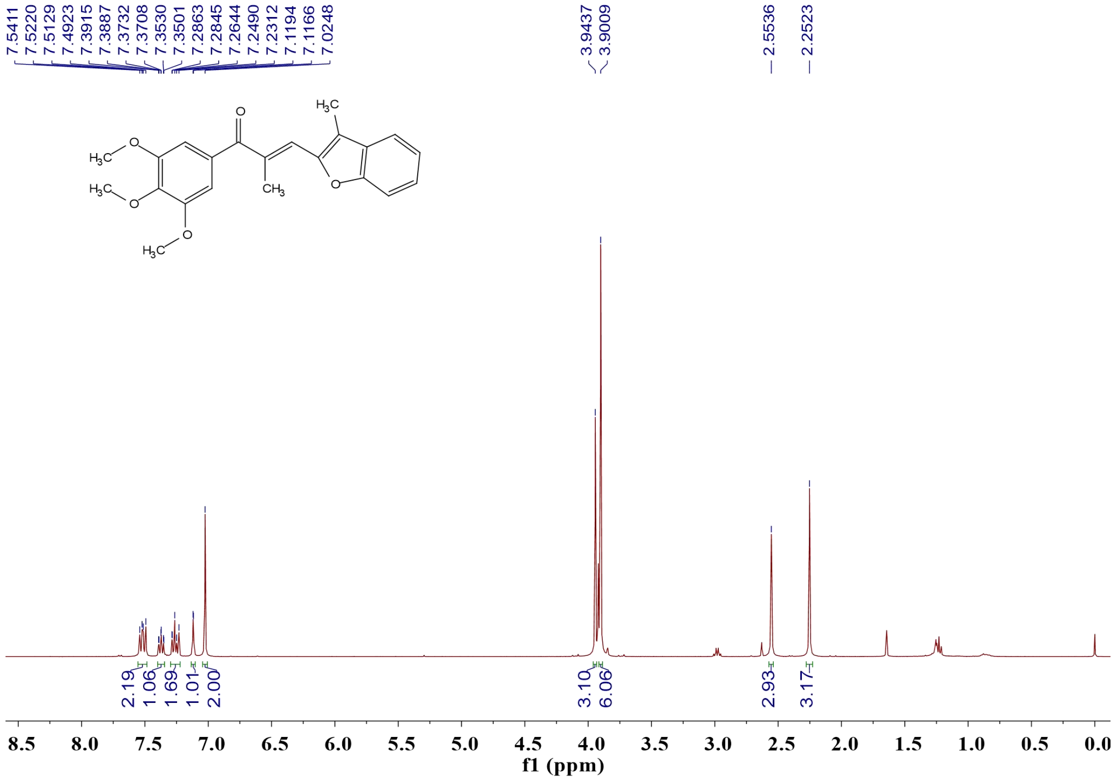

^{1}H NMR of **7i**

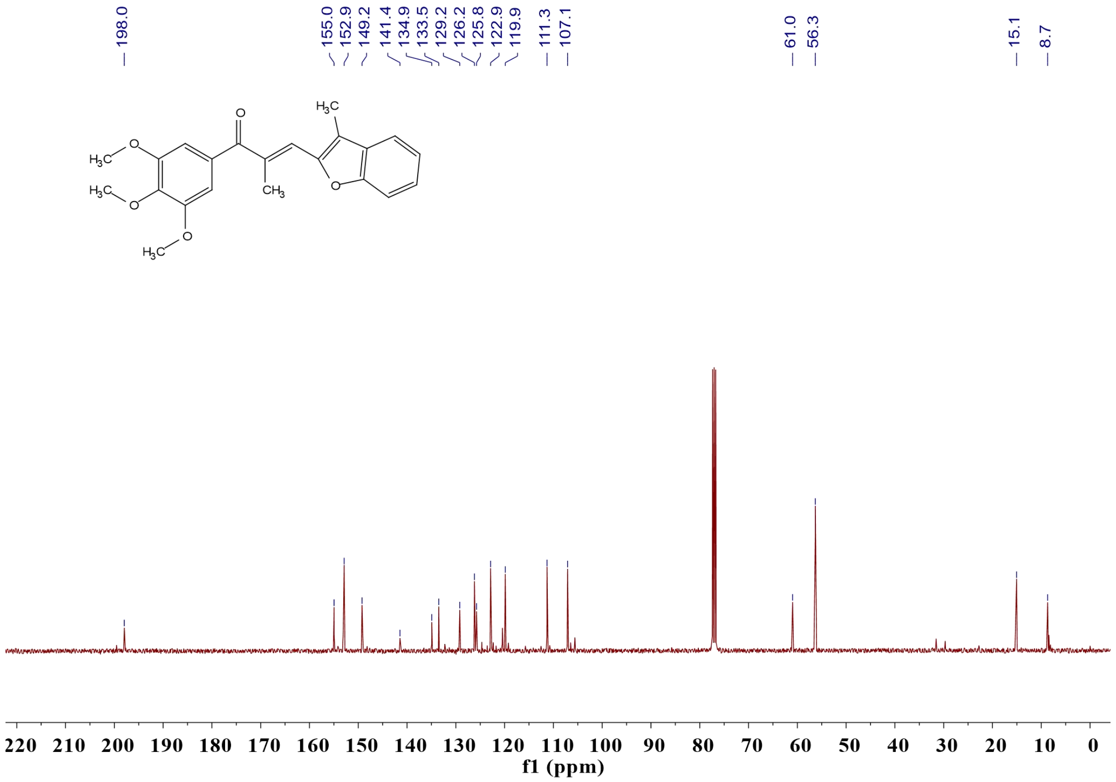

198.0
155.0
152.9
149.2
141.4
134.9
133.5
129.2
126.2
125.8
122.9
119.9
111.3
107.1
61.0
56.3
15.1
8.7
f1 (ppm)

^{13}C NMR of **7i**

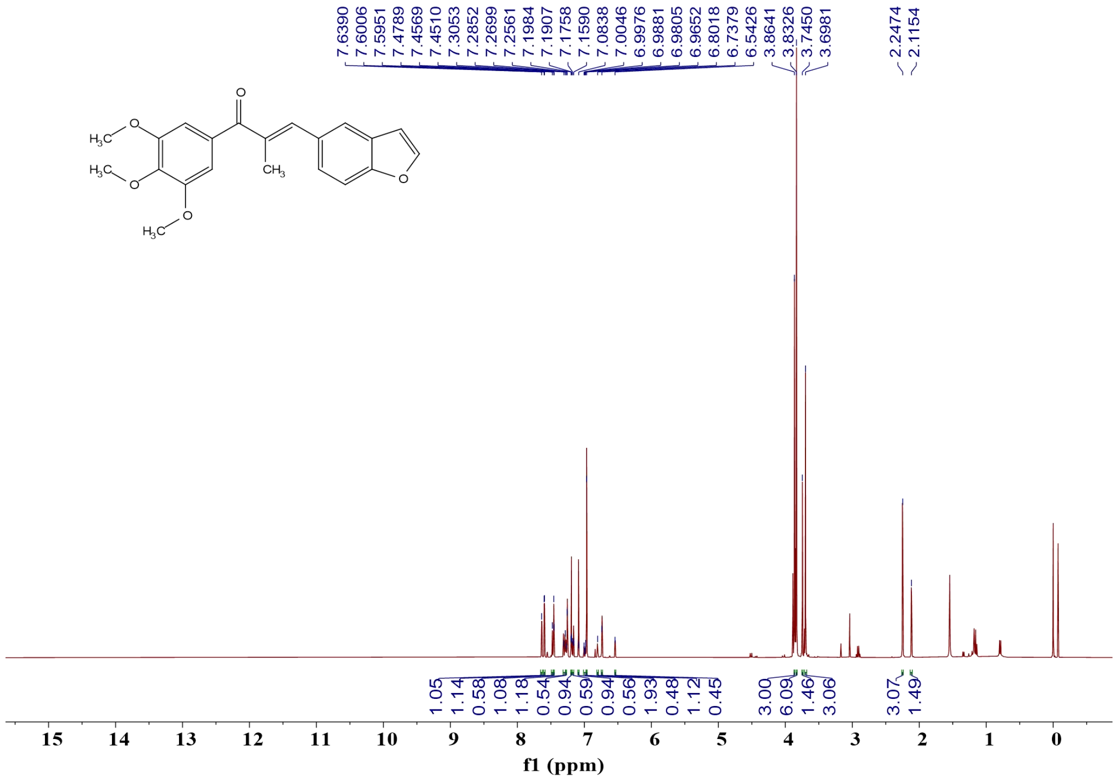

7.6390
7.6006
7.5951
7.4789
7.4569
7.4510
7.3053
7.2852
7.2699
7.2561
7.1984
7.1907
7.1758
7.1590
7.0838
7.0046
6.9976
6.9881
6.9805
6.9652
6.8018
6.7379
6.5426
3.8641
3.8326
3.7450
3.6981
2.2474
2.1154
1.05
1.14
0.58
1.08
1.18
0.54
0.94
0.59
0.94
0.56
1.93
0.48
1.12
0.45
3.00
6.09
1.46
3.06
3.07
1.49
f1 (ppm)

^{1}H NMR of **7m**

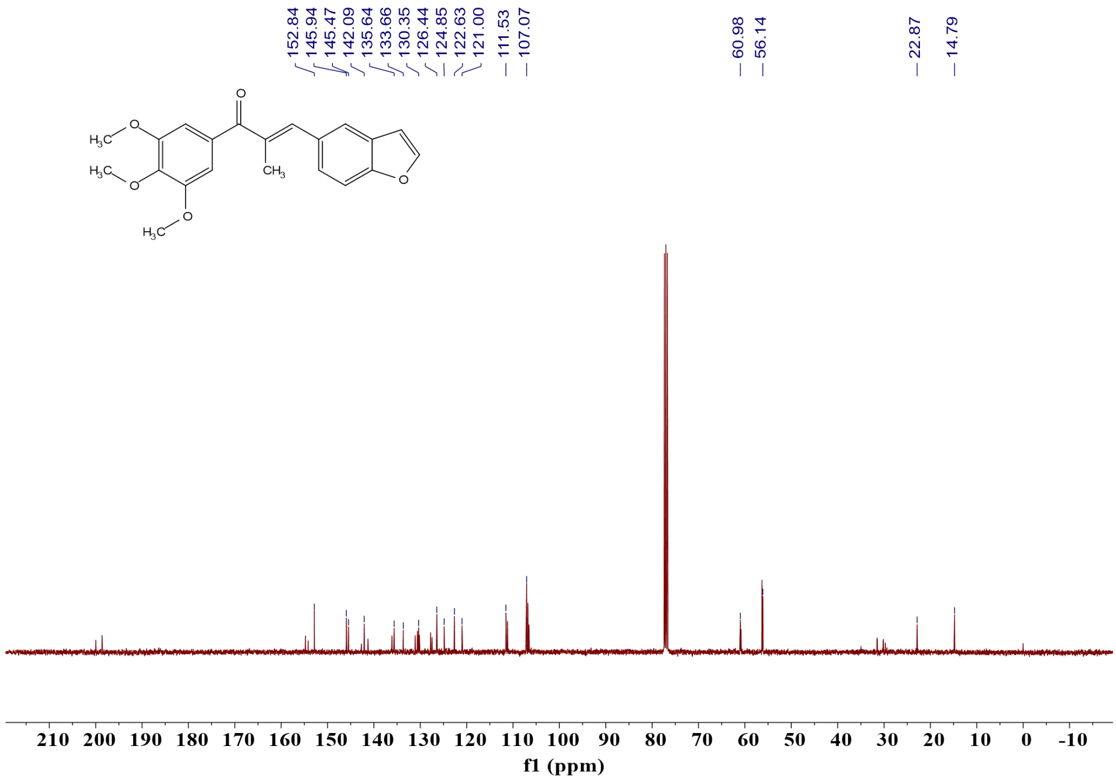

^{13}C NMR of **7m**

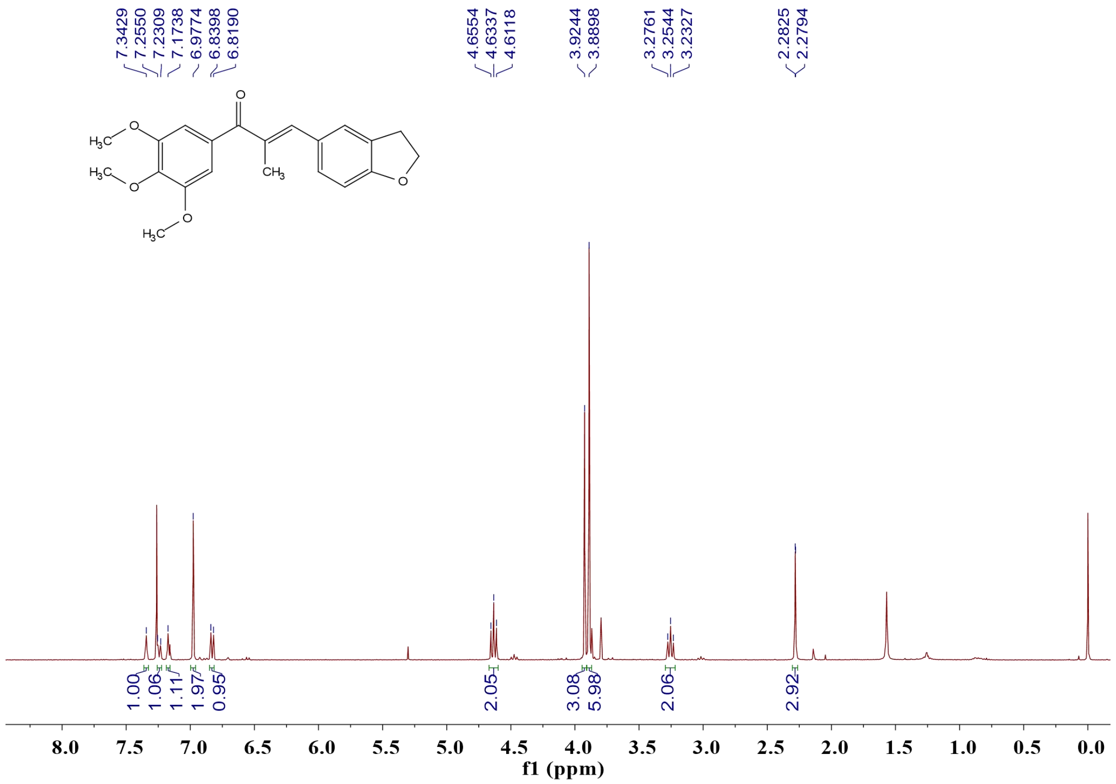

^{1}H NMR of **7n**

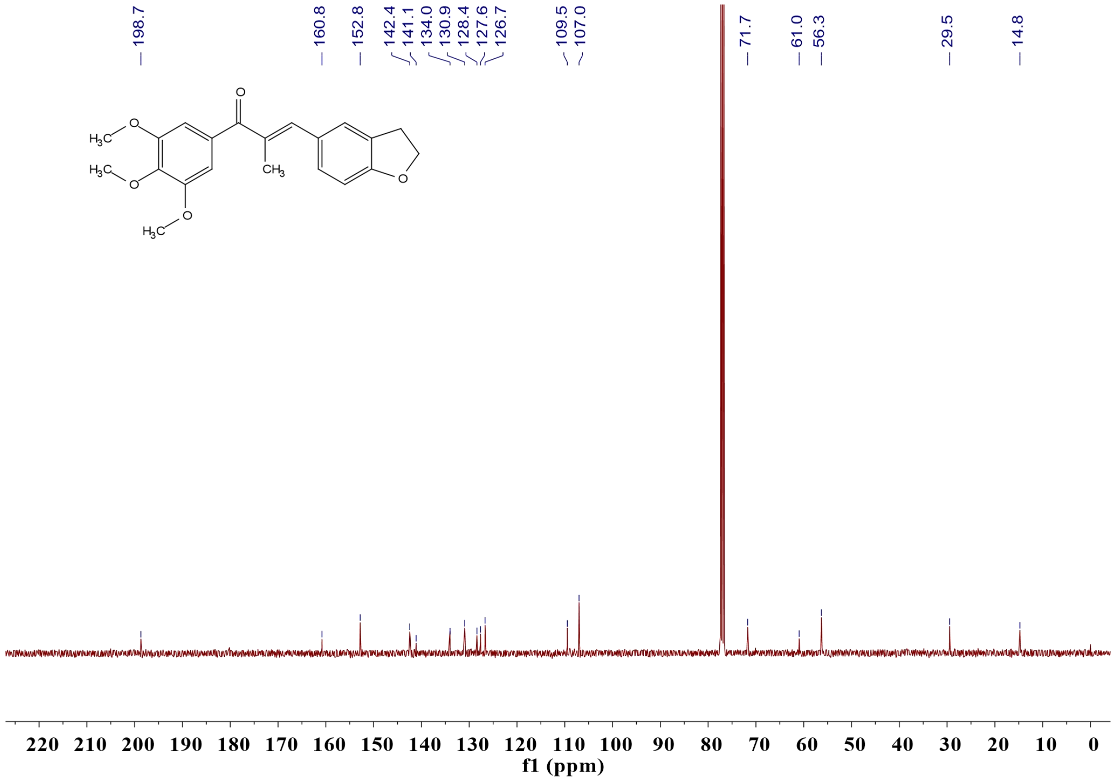

^{13}C NMR of **7n**

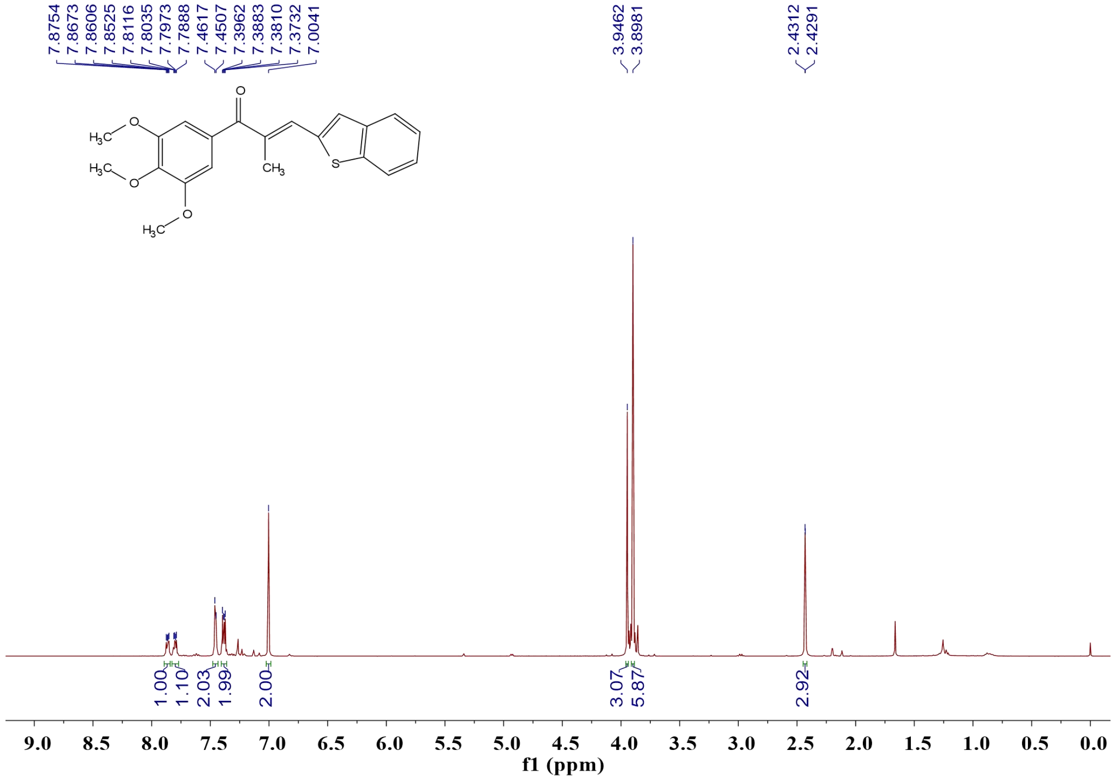

^{1}H NMR of **7o**

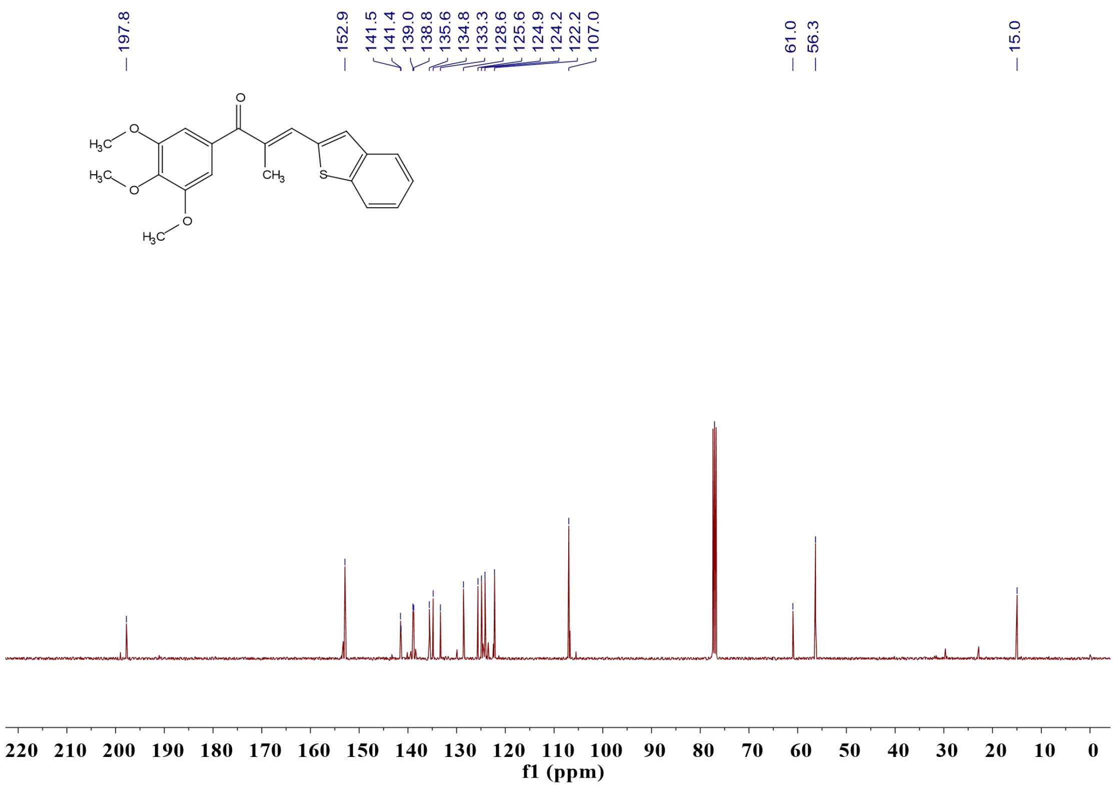

^{13}C NMR of **7o**

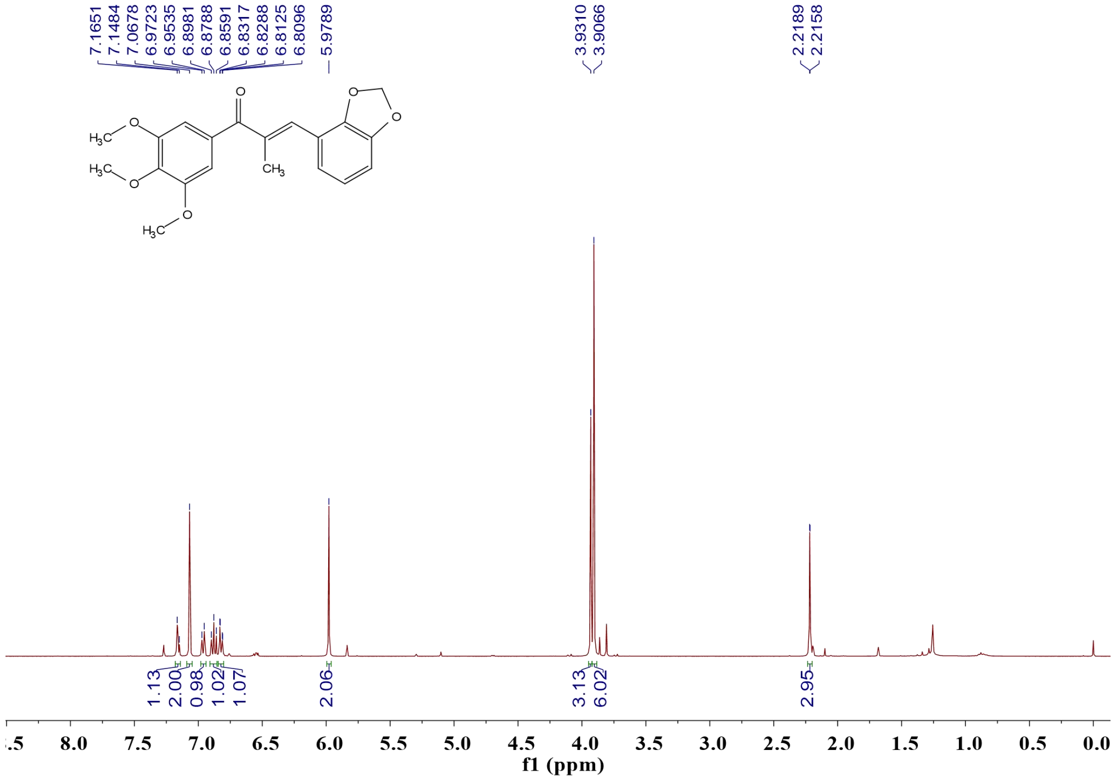

^{1}H NMR of **7s**

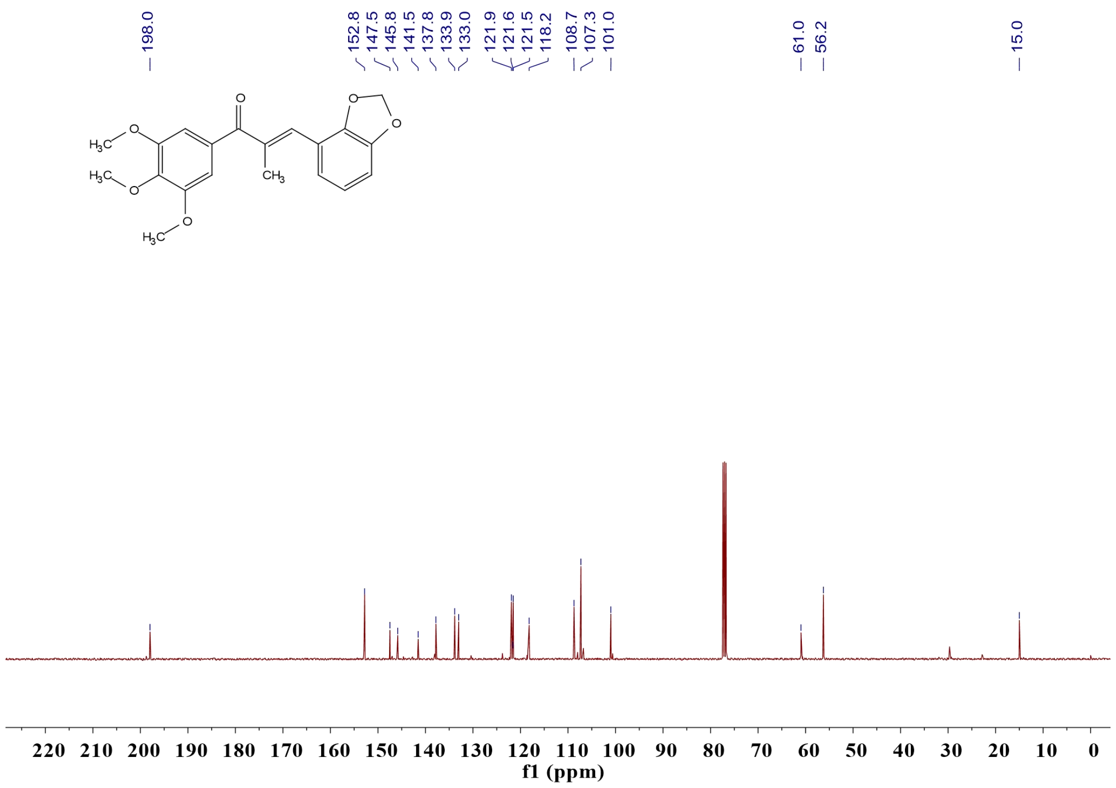

^{13}C NMR of **7s**

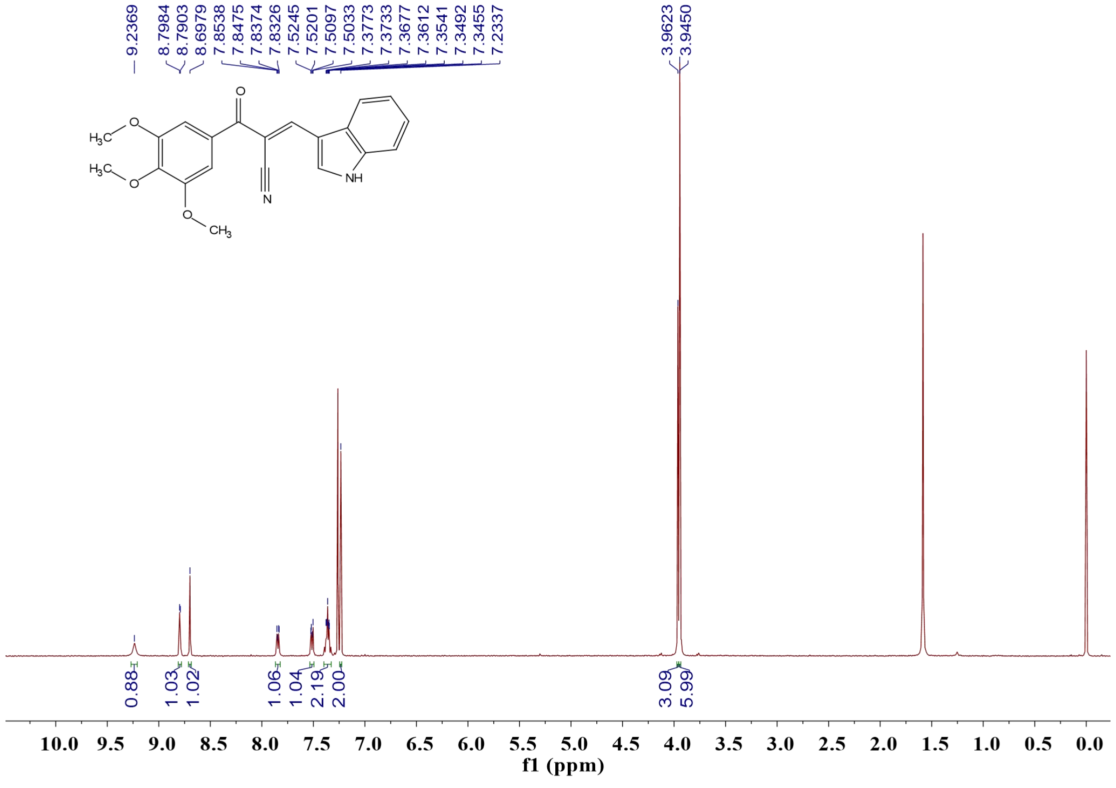

^{1}H NMR of **8a**

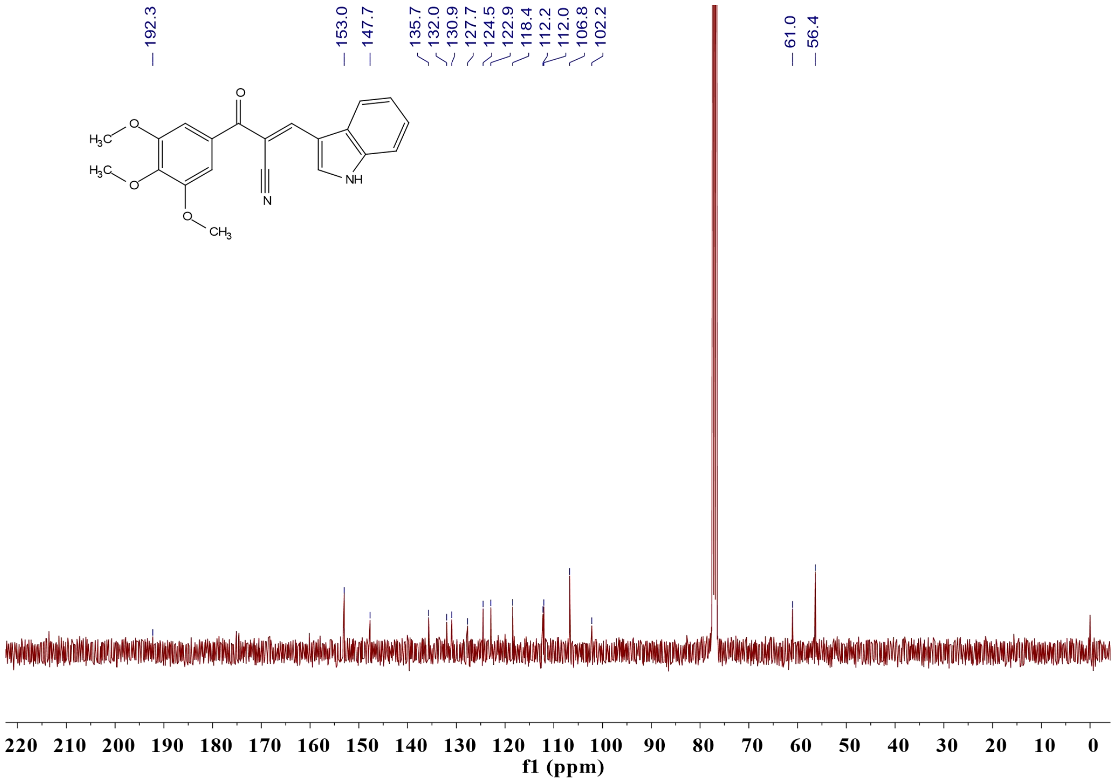

^{13}C NMR of **8a**

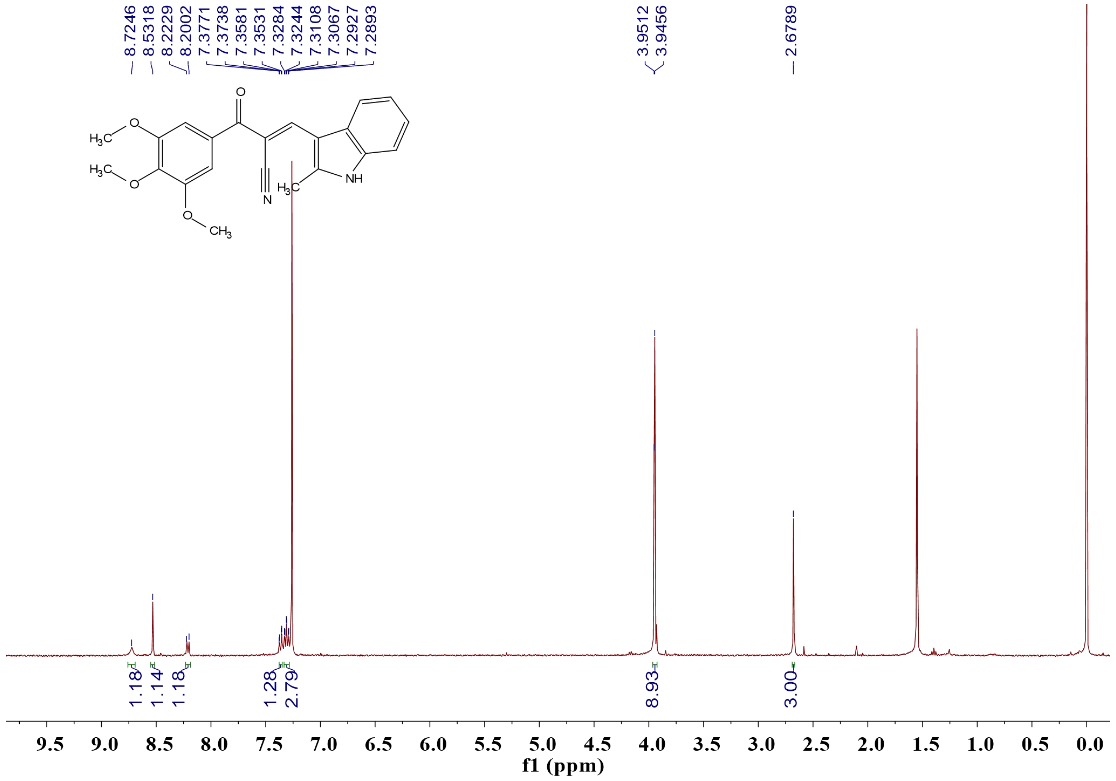

^{1}H NMR of **8b**

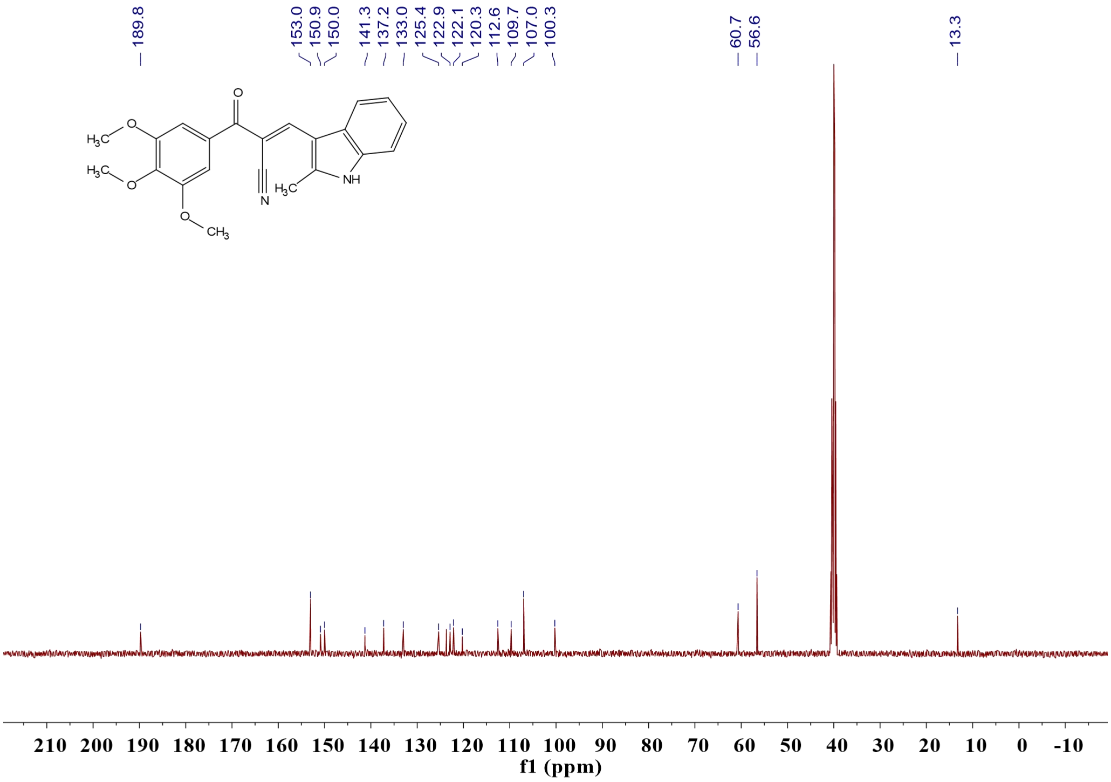

^{13}C NMR of **8b**

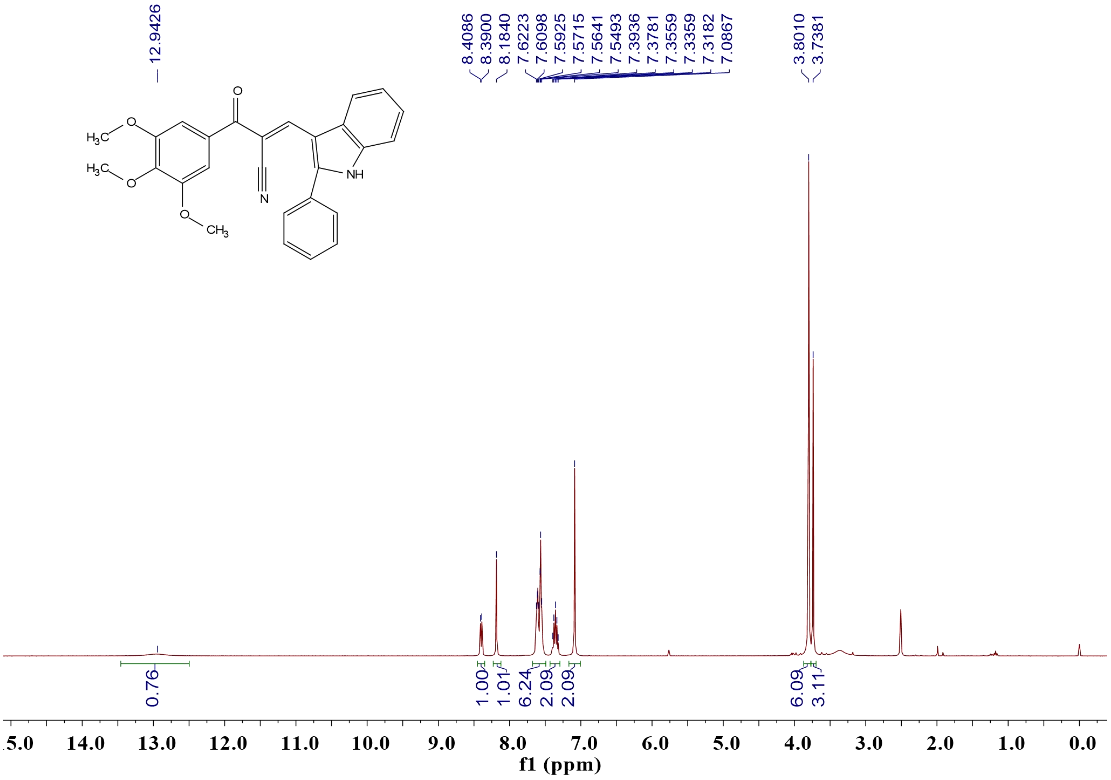

^{1}H NMR of **8c**

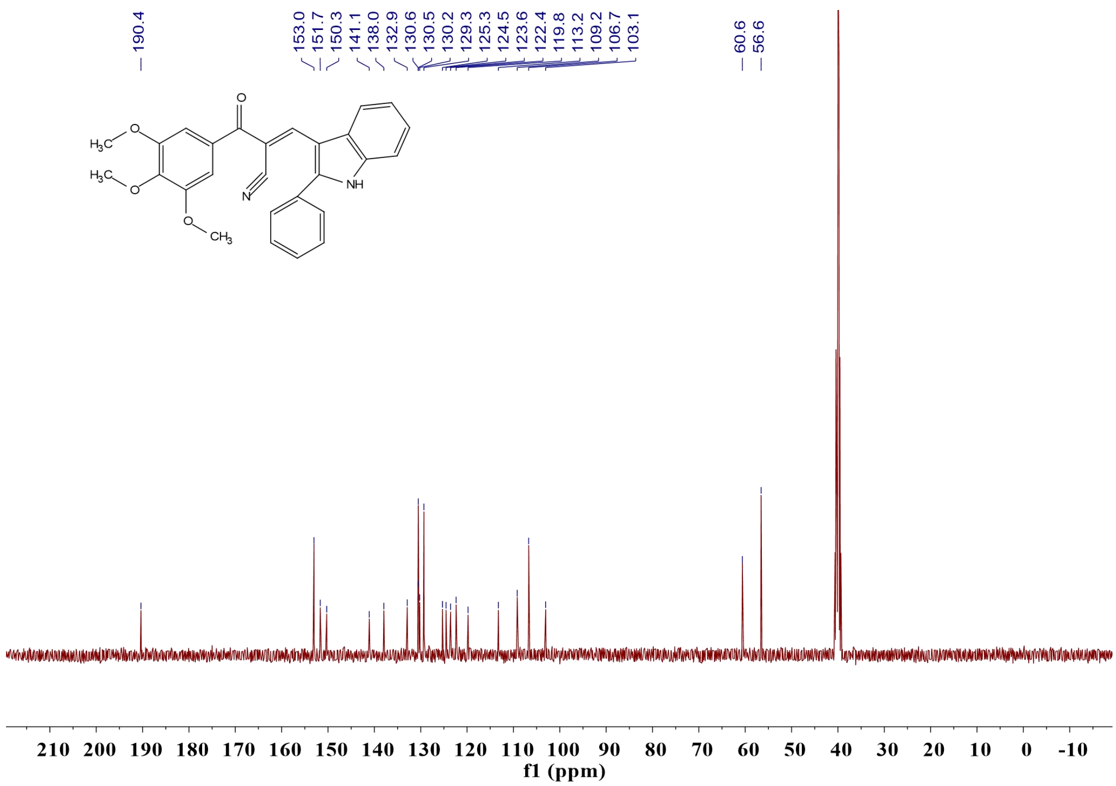

^{13}C NMR of **8c**

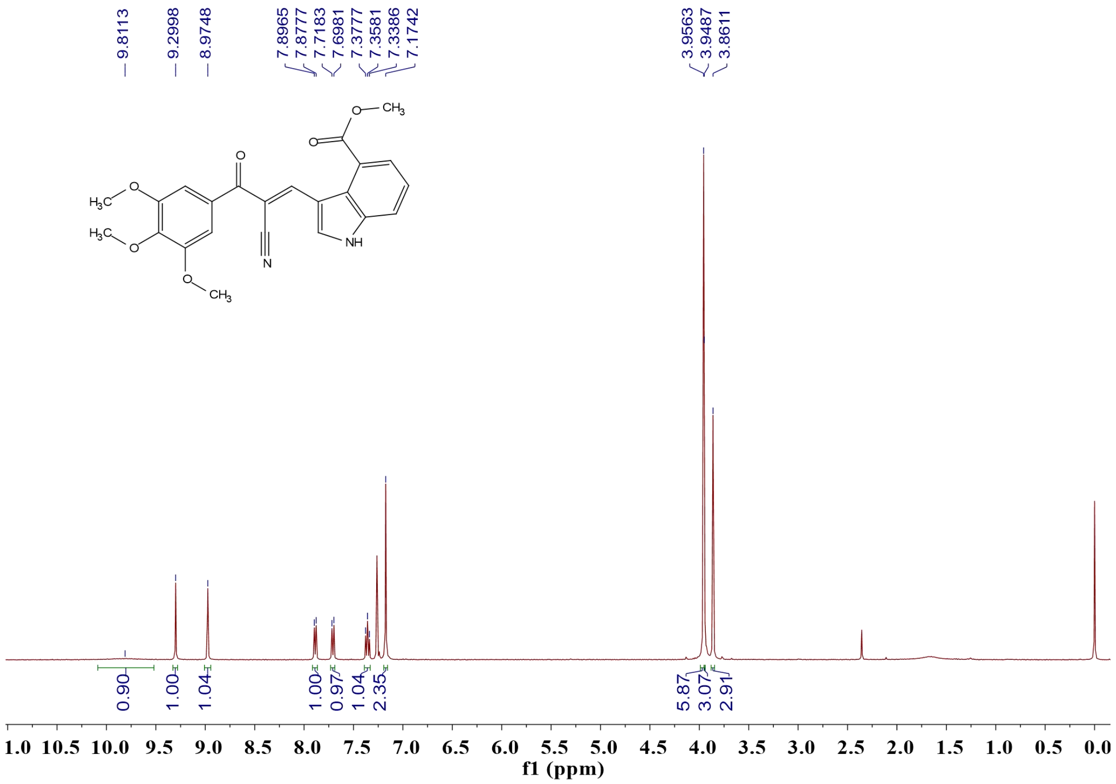

^{1}H NMR of **8d**

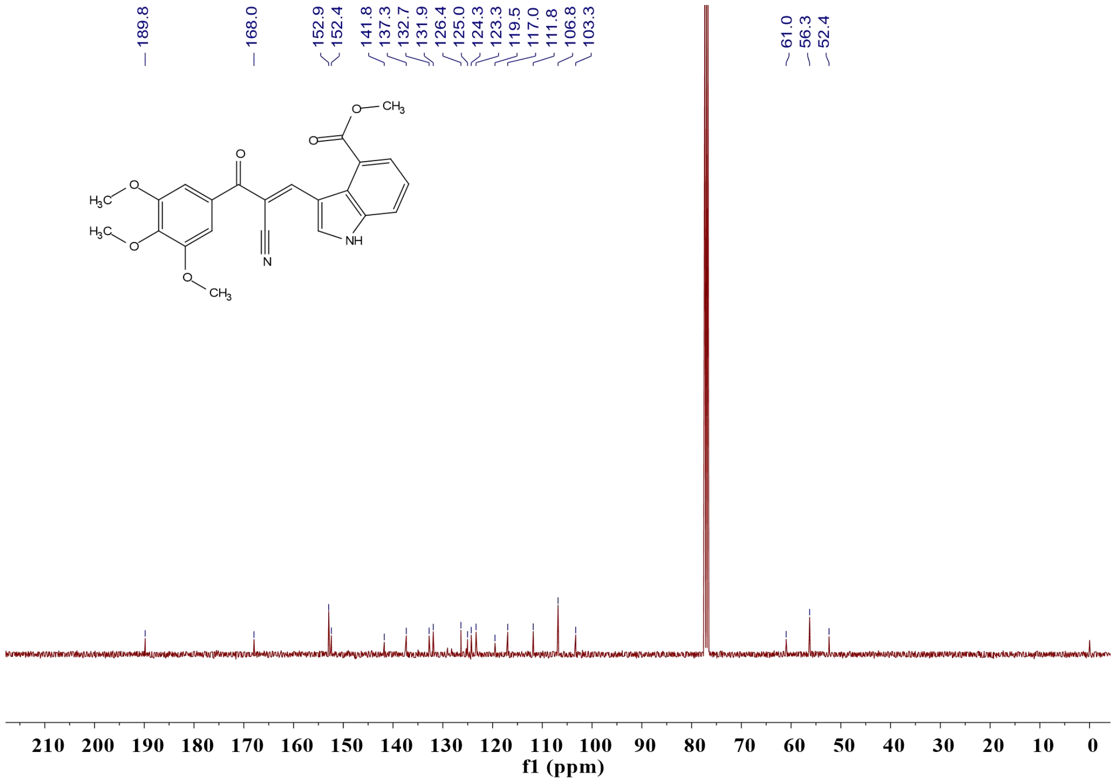

^{13}C NMR of **8d**

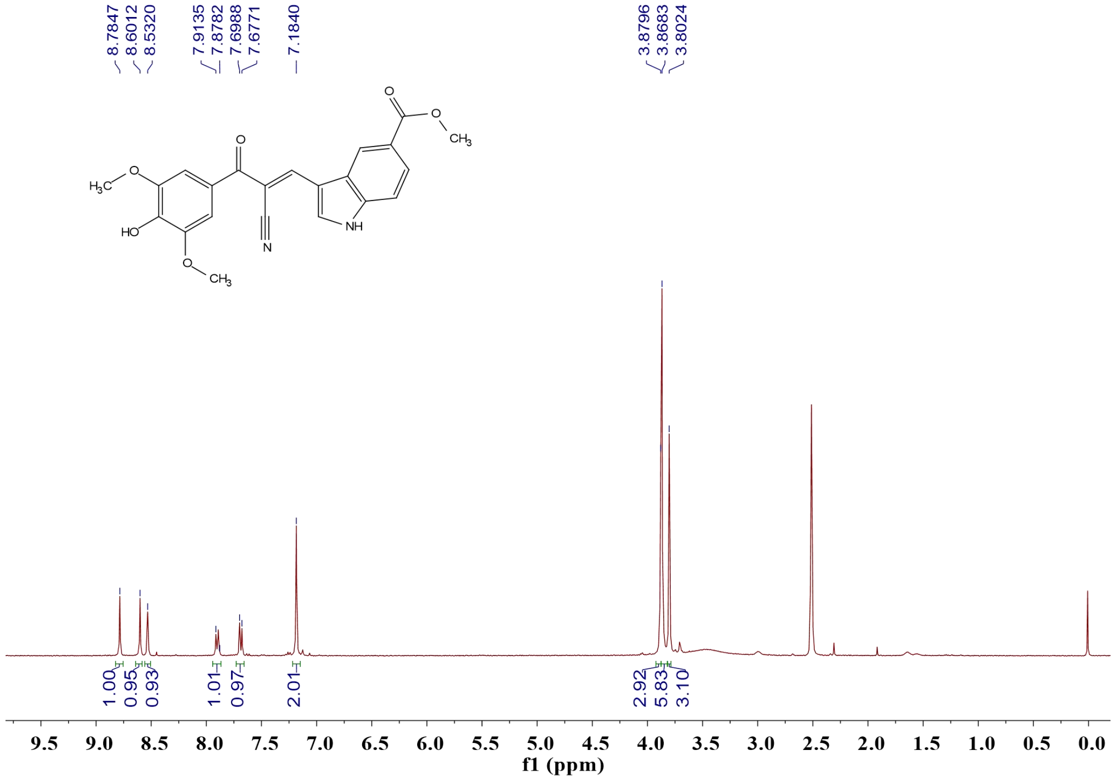

^{1}H NMR of **8e**

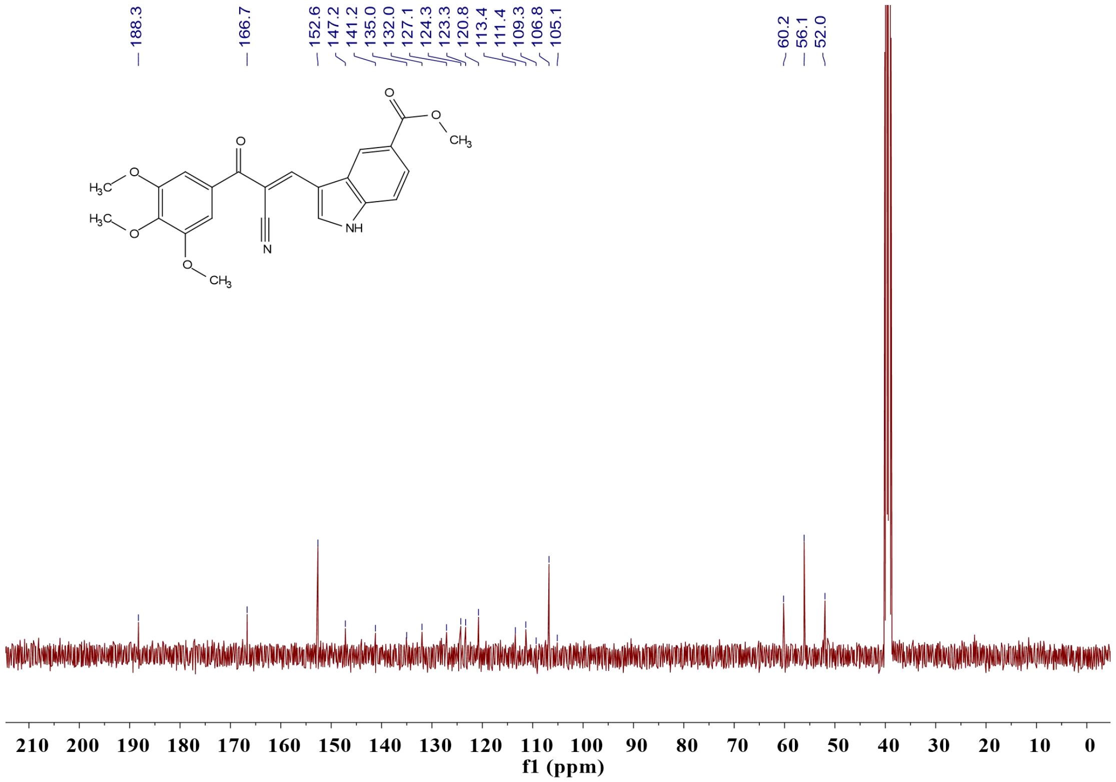

^{13}C NMR of **8e**

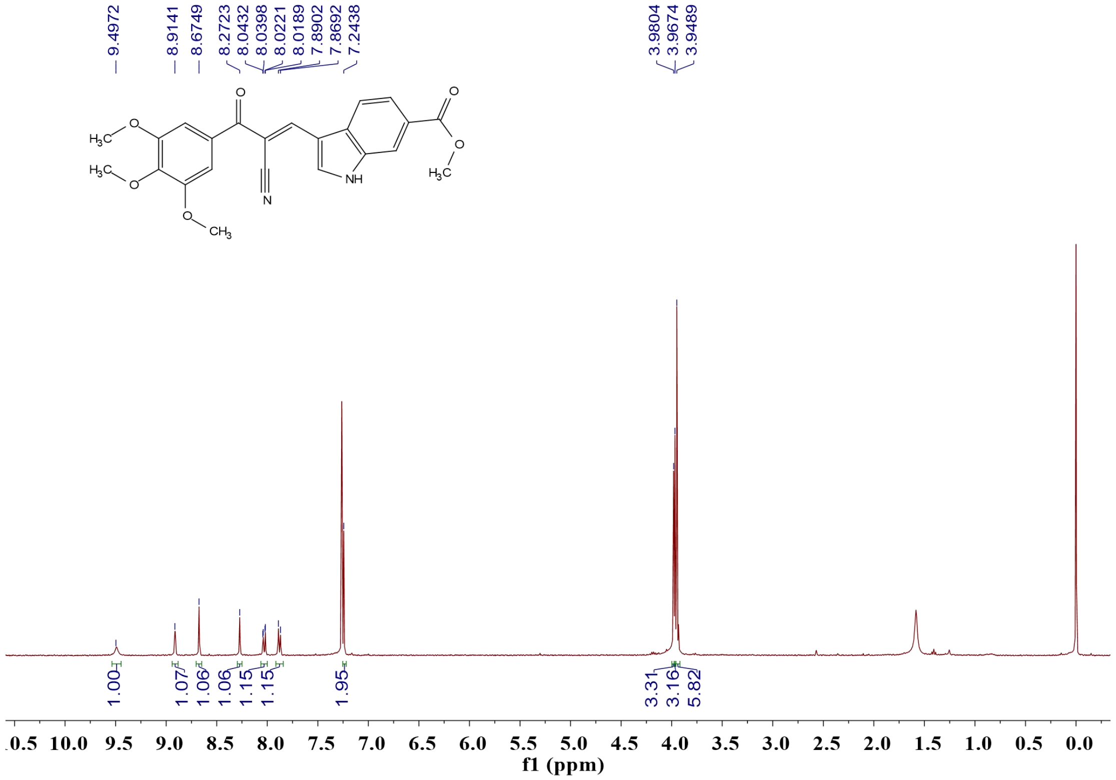

^{1}H NMR of **8f**

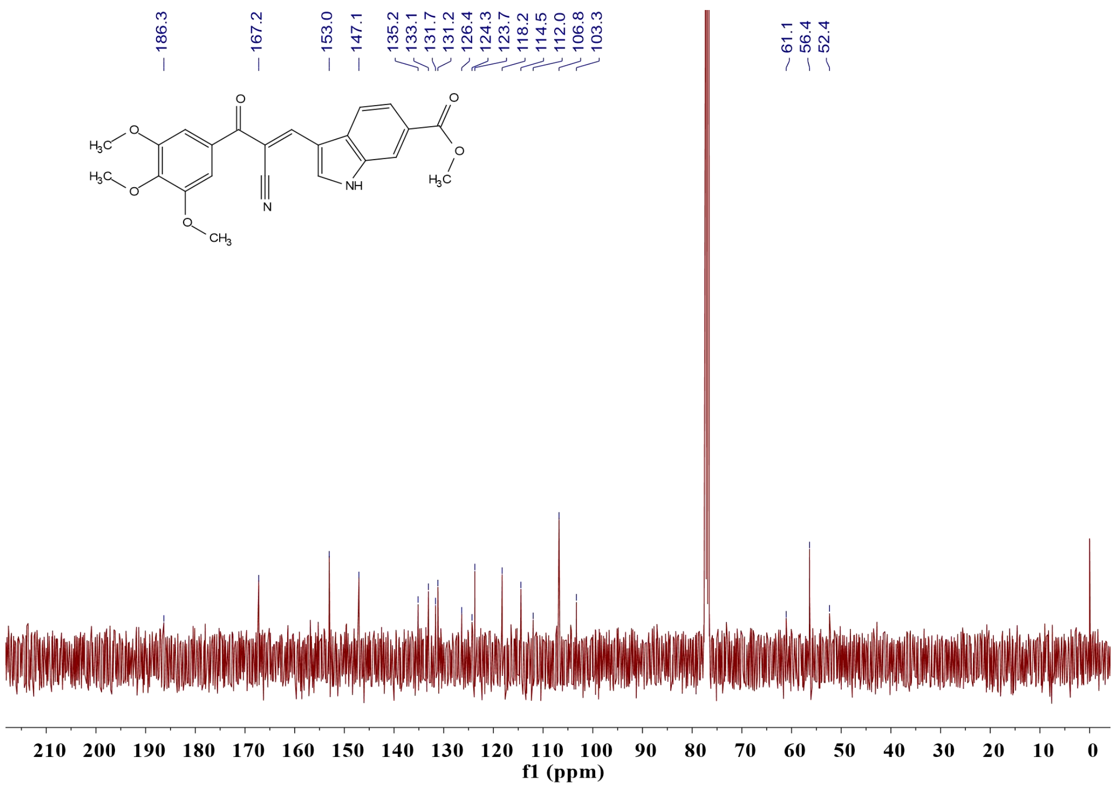

^{13}C NMR of **8f**

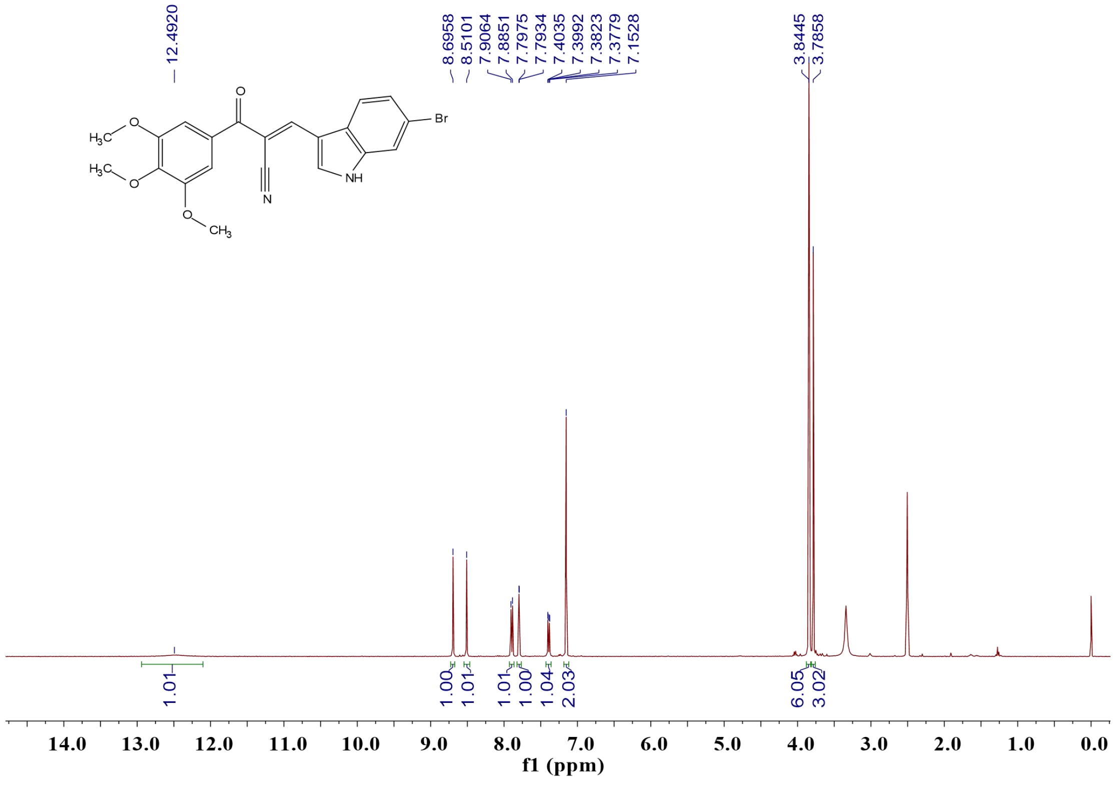

^{1}H NMR of **8g**

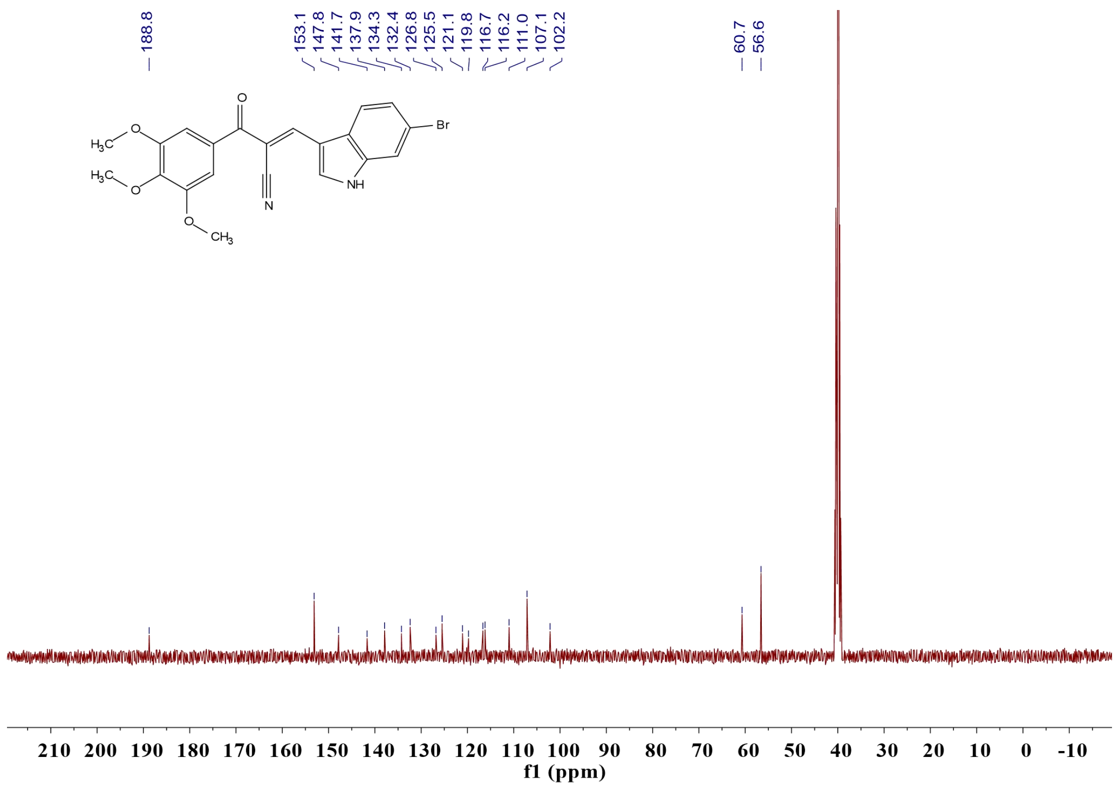

^{13}C NMR of **8g**

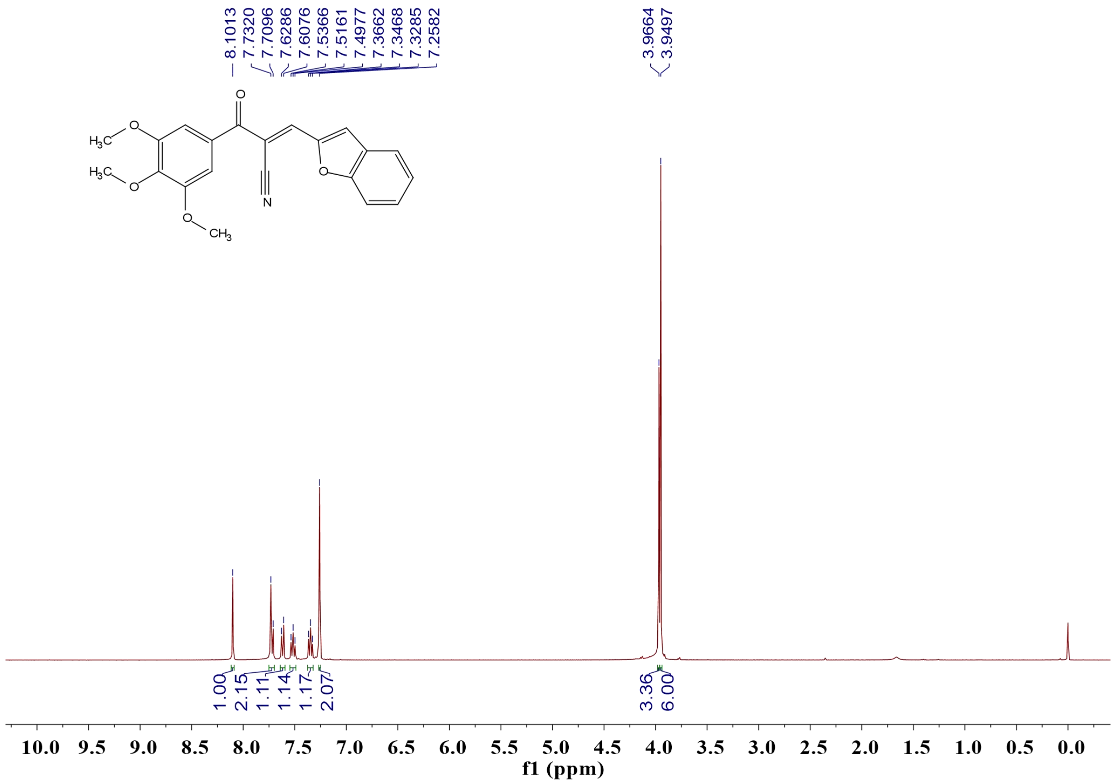

^{1}H NMR of **8h**

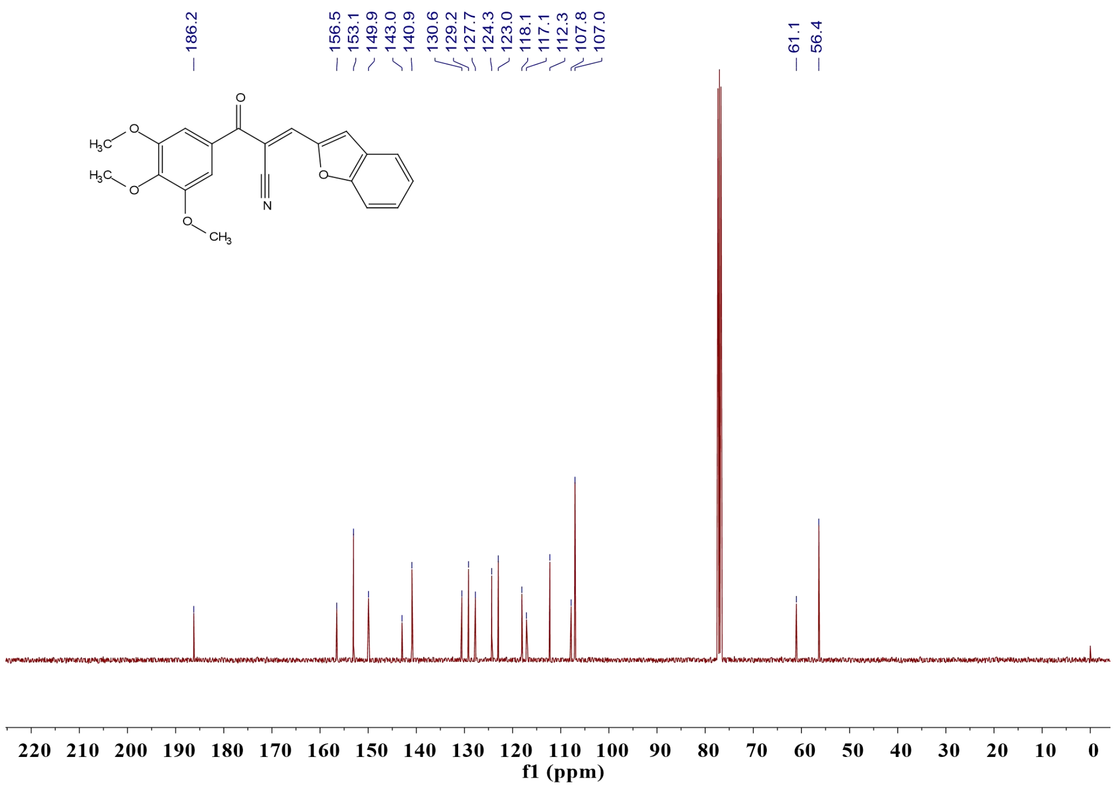

^{13}C NMR of **8h**

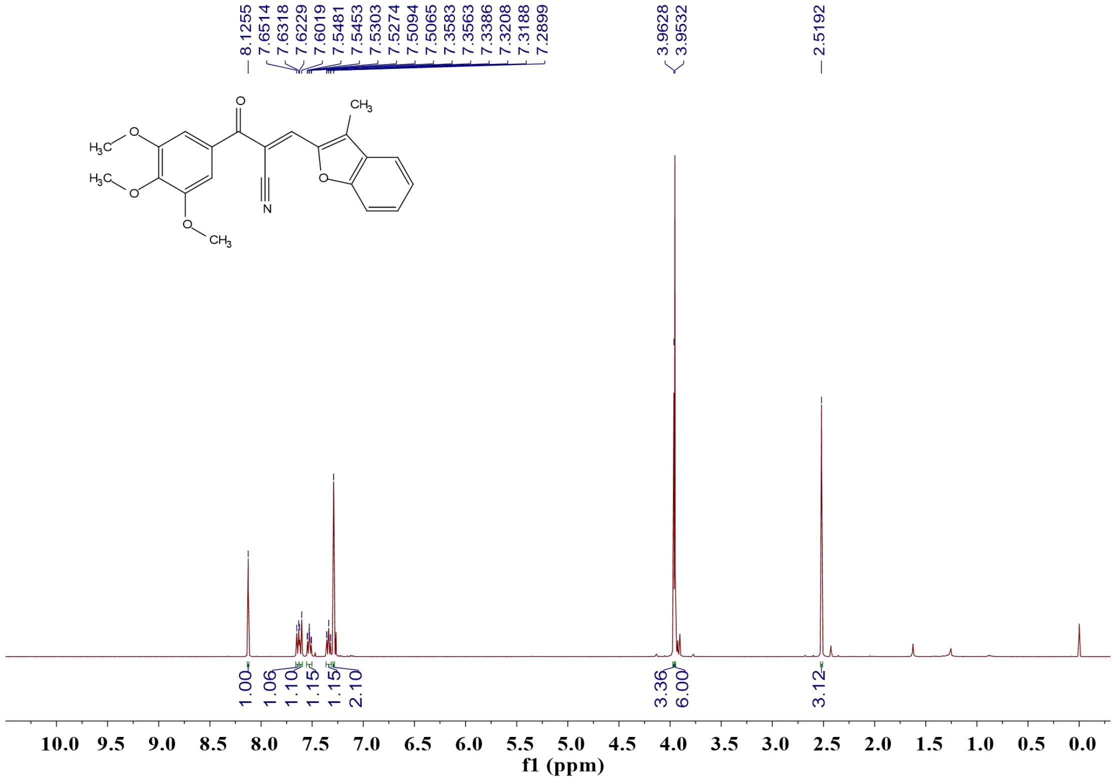

^{1}H NMR of **8i**

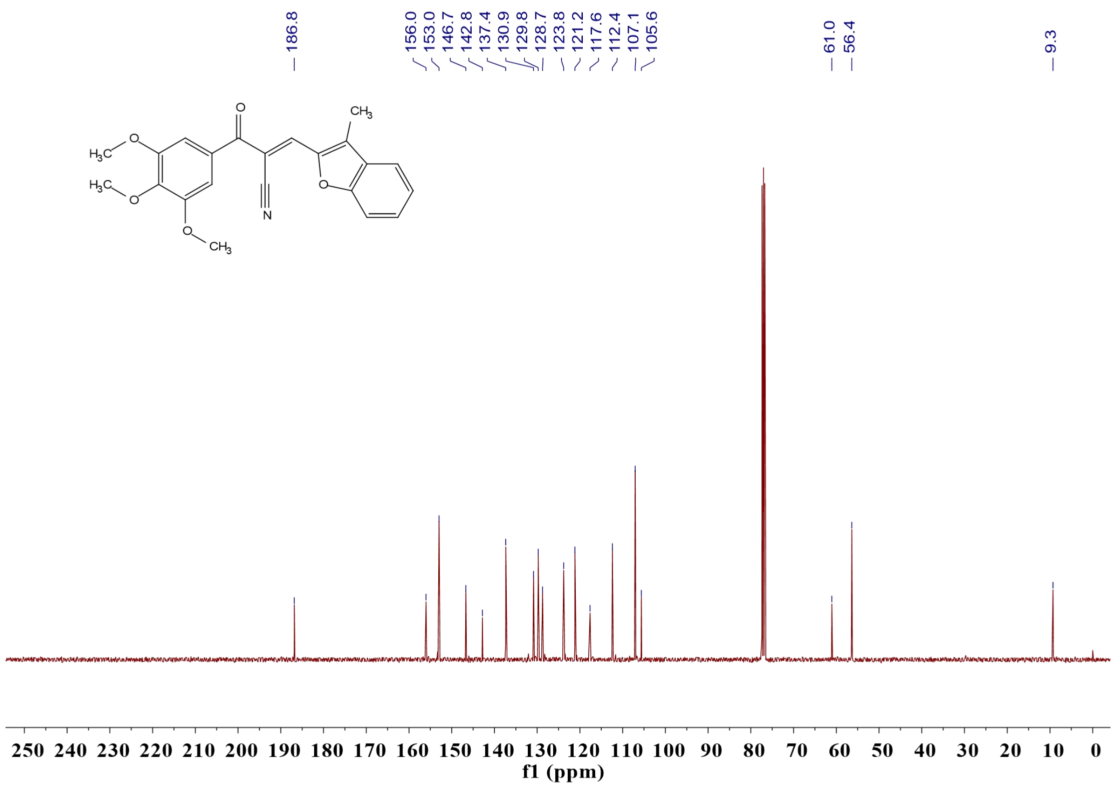

^{13}C NMR of **8i**

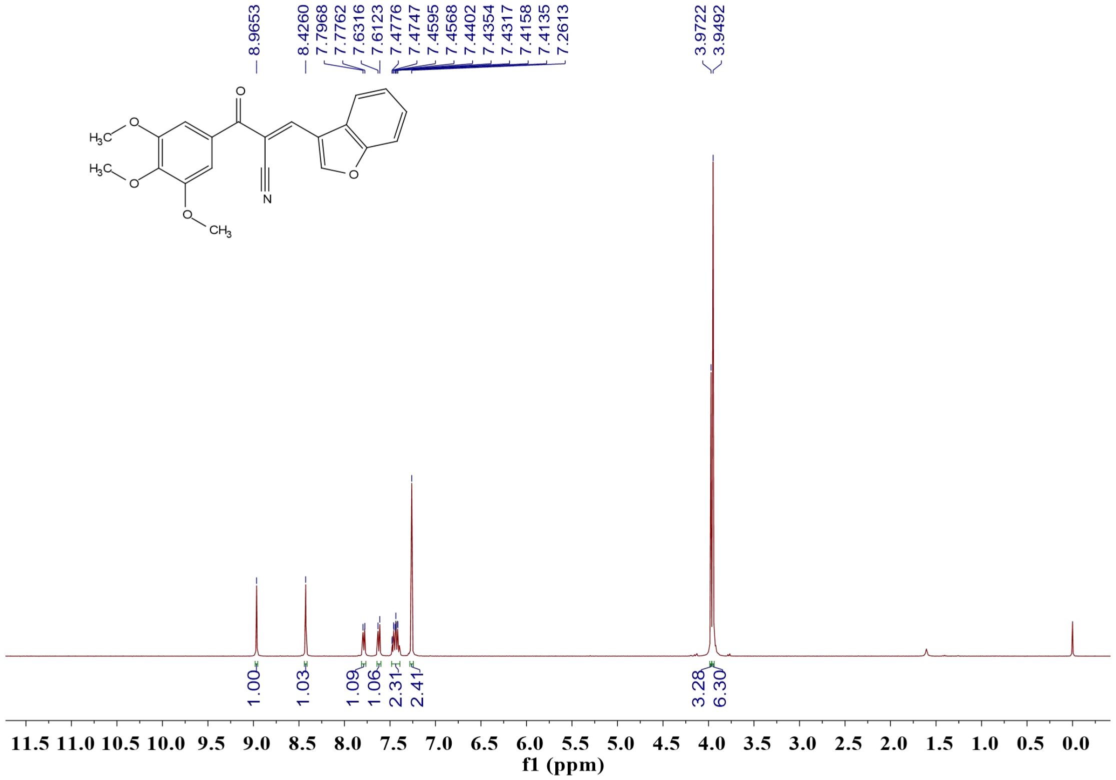

^{1}H NMR of **8j**

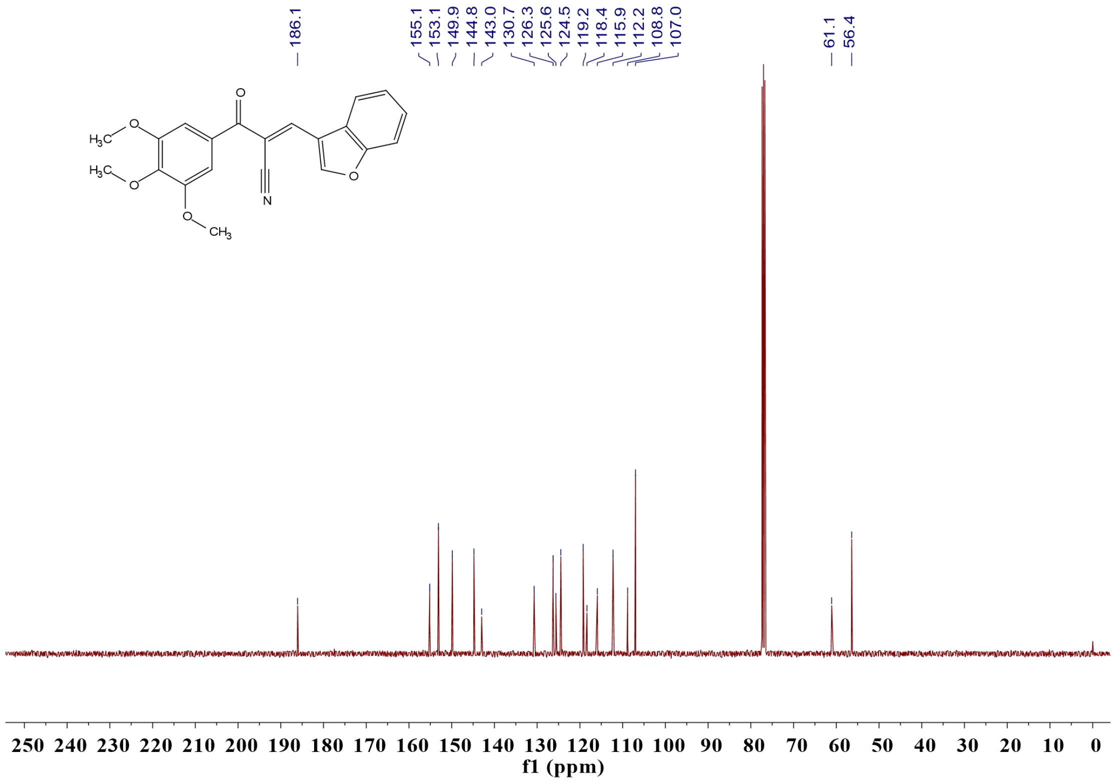

^{13}C NMR of **8j**

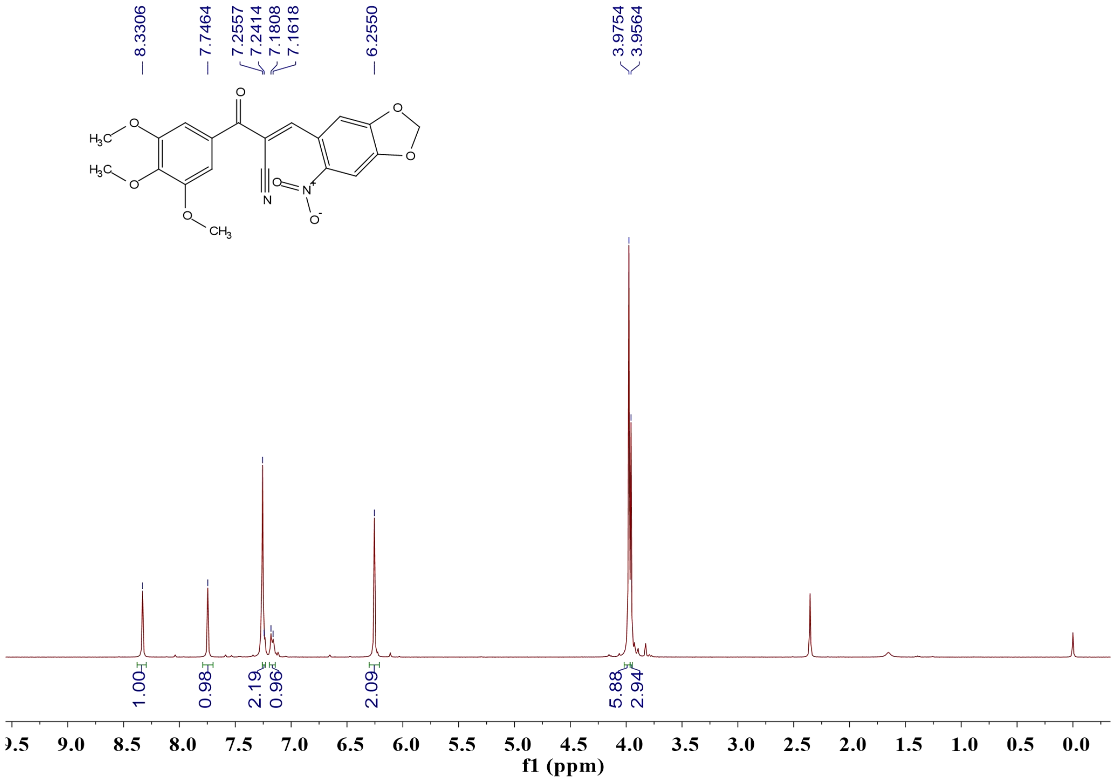

^{1}H NMR of **8k**

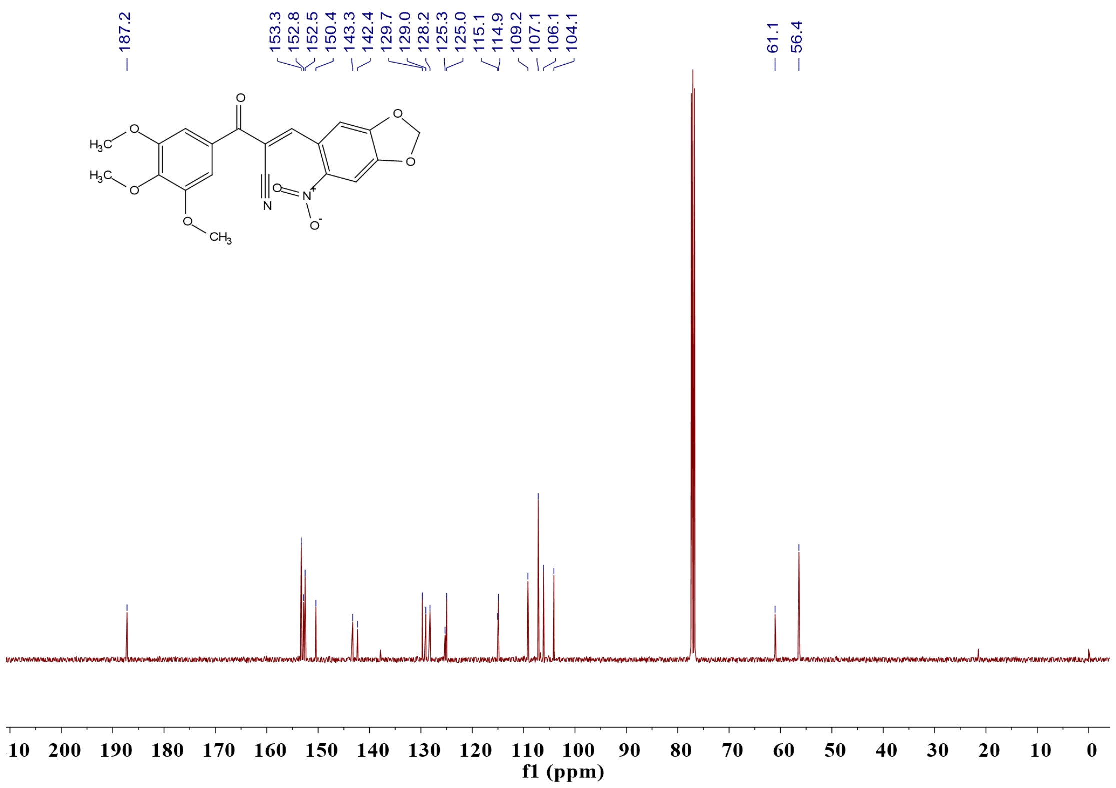
187.2
153.3
152.8
152.5
150.4
143.3
142.4
129.7
129.0
128.2
125.3
125.0
115.1
114.9
109.2
107.1
106.1
104.1
61.1
56.4
O
H_3C
N
CH_3
f1 (ppm)

^{13}C NMR of **8k**

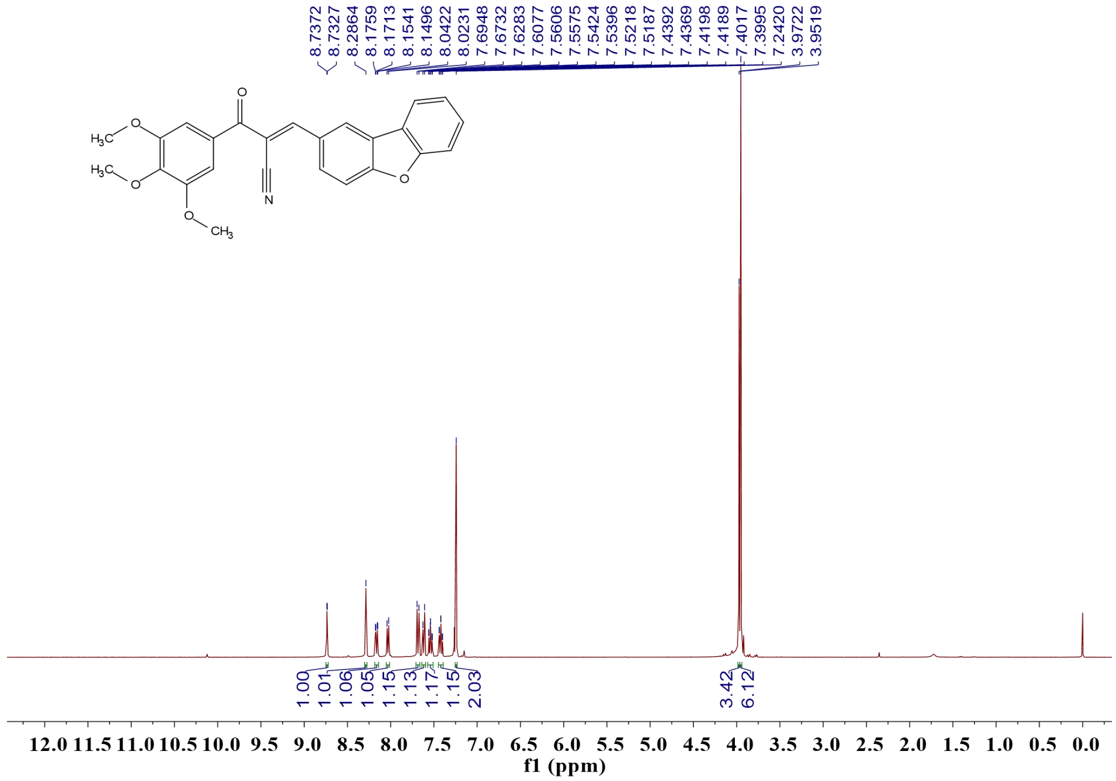
8.7372
8.7327
8.2864
8.1759
8.1713
8.1541
8.1496
8.0422
8.0231
7.6948
7.6732
7.6283
7.6077
7.5606
7.5575
7.5424
7.5396
7.5218
7.5187
7.4392
7.4369
7.4198
7.4189
7.4017
7.3995
7.2420
3.9722
3.9519
O
H_3C
N
CH_3
1.00
1.01
1.06
1.05
1.15
1.13
1.17
1.15
2.03
3.42
6.12
f1 (ppm)

^{1}H NMR of **8l**

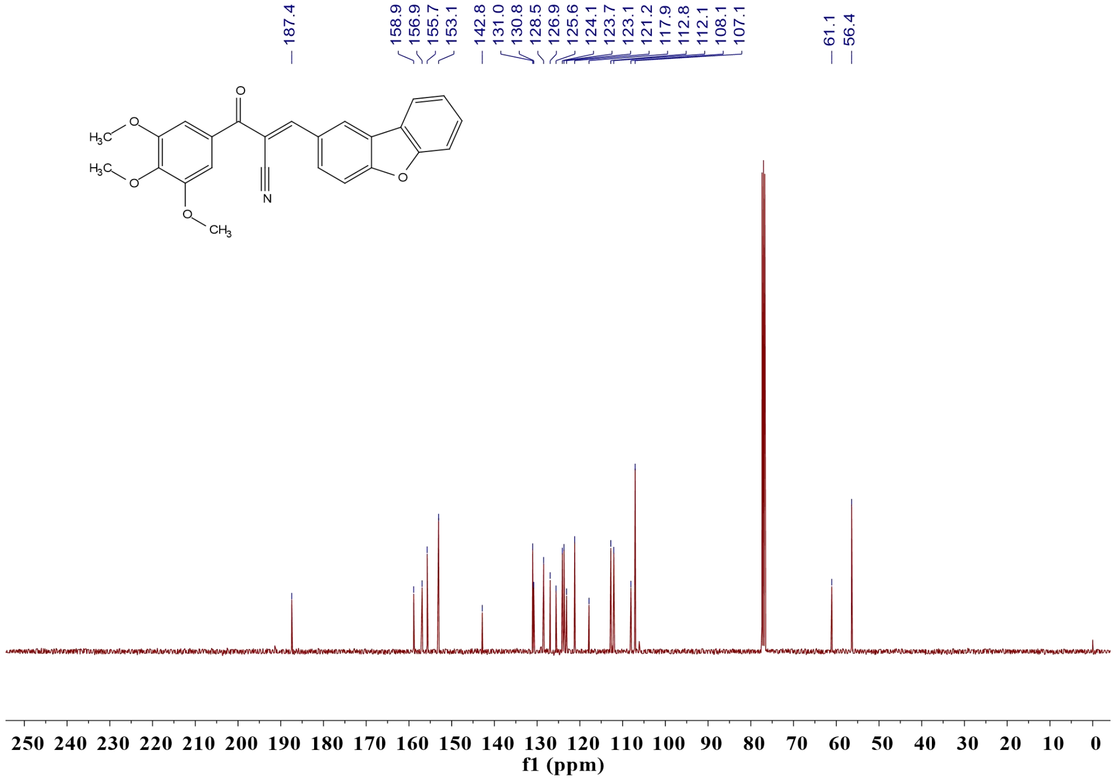

^{13}C NMR of **8l**

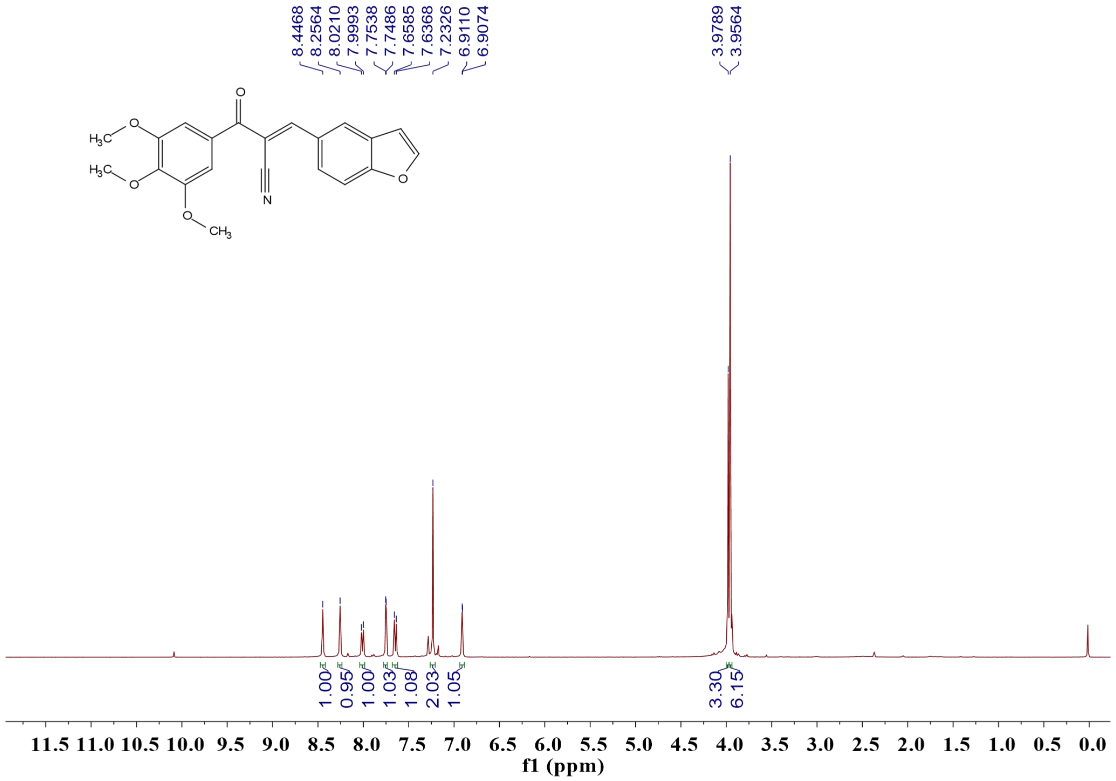

^{1}H NMR of **8m**

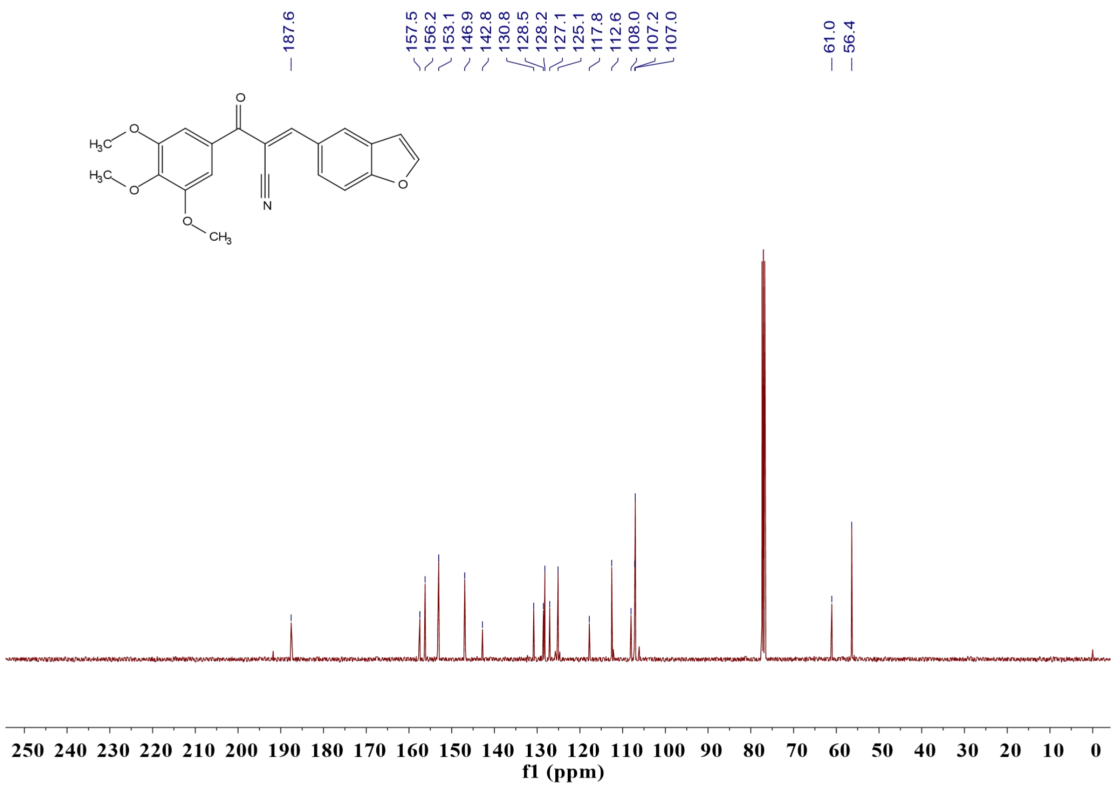

^{13}C NMR of **8m**

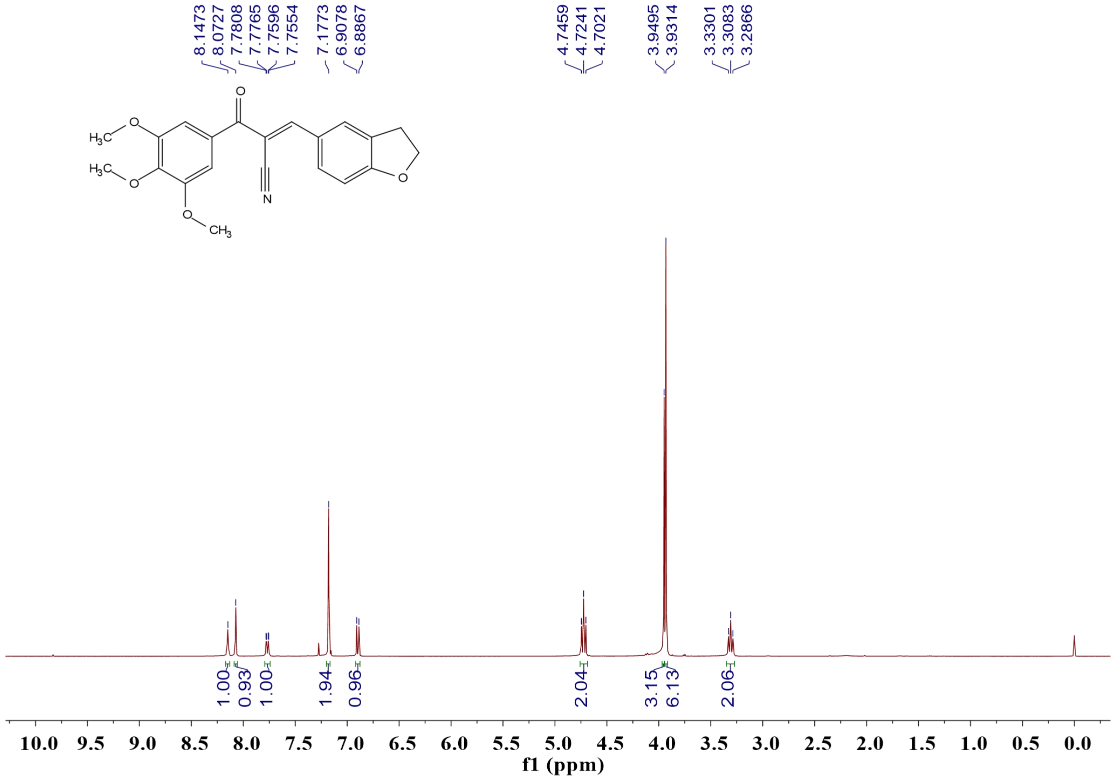

^{1}H NMR of **8n**

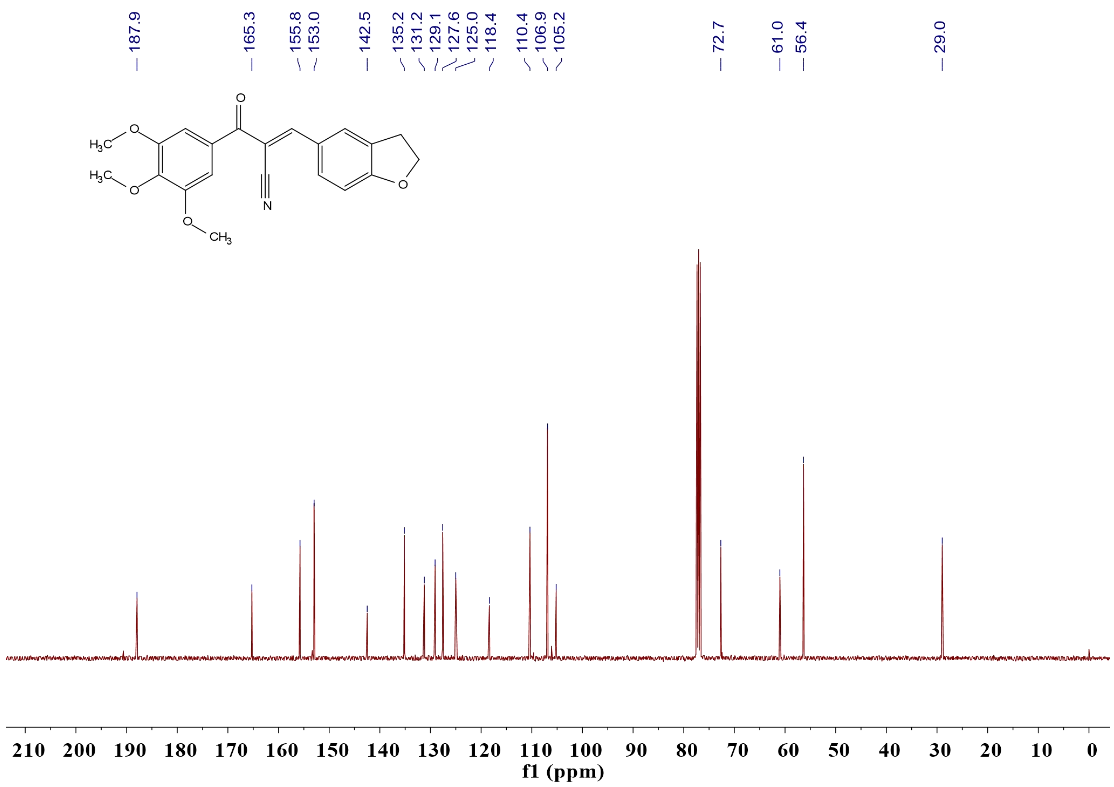

^{13}C NMR of **8n**

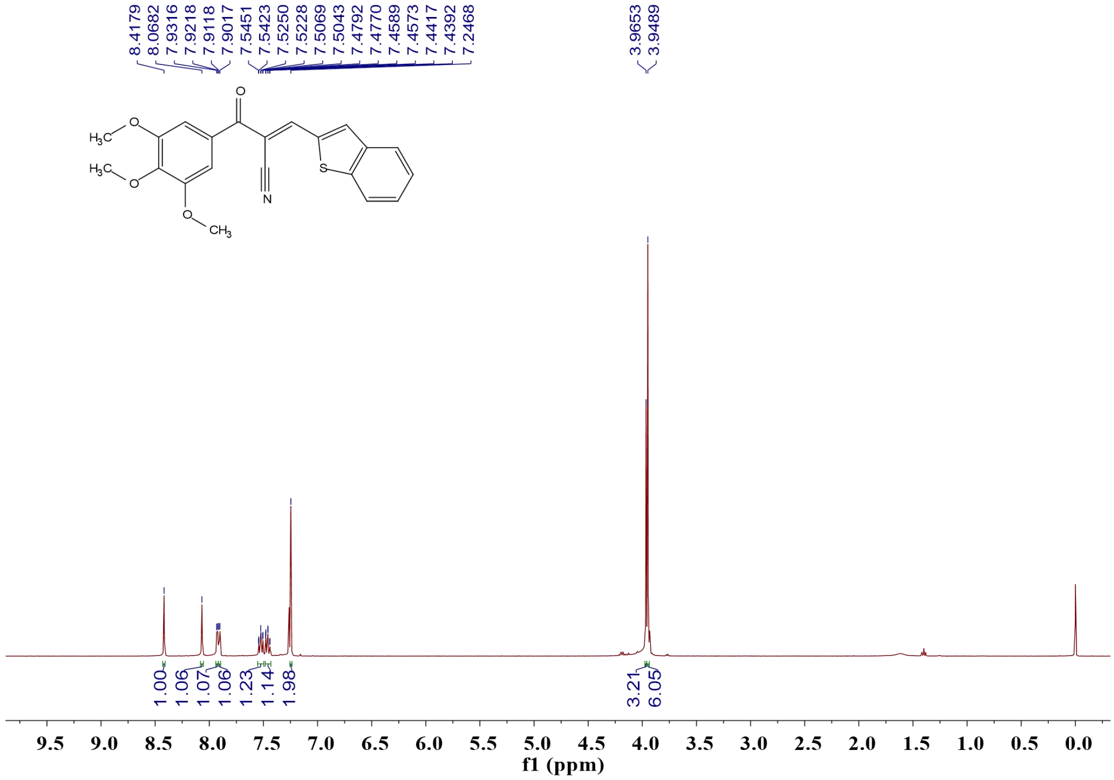

^{1}H NMR of **8o**

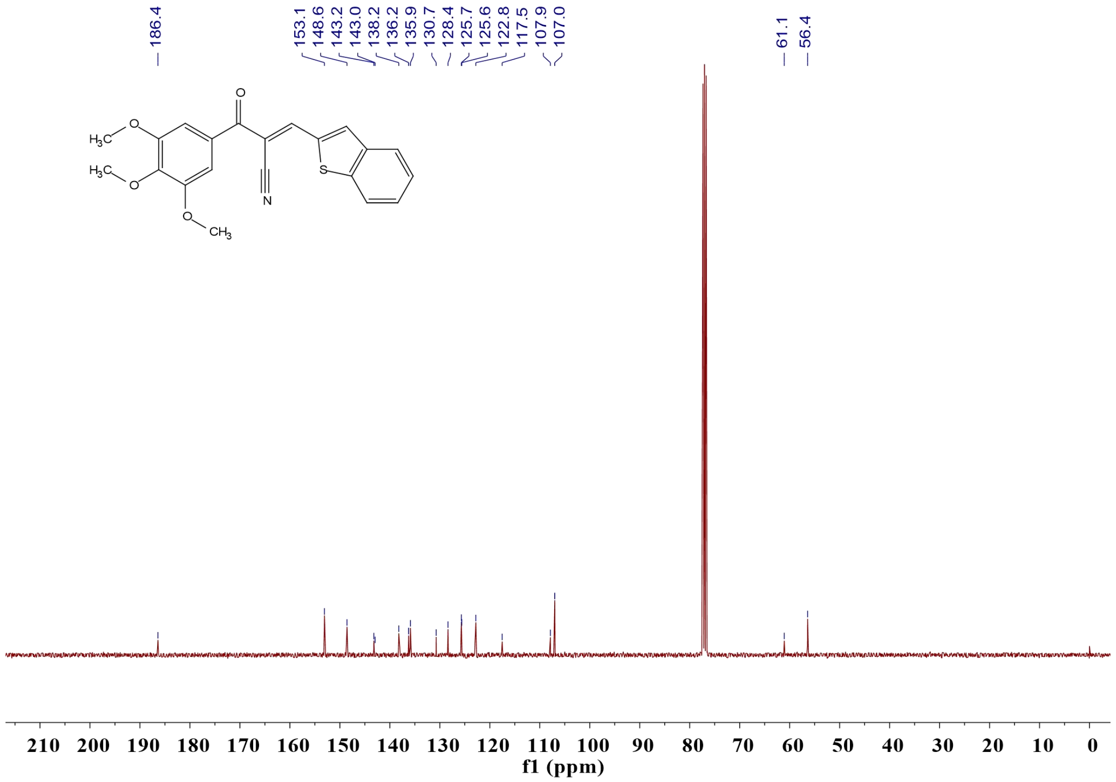

^{13}C NMR of **8o**

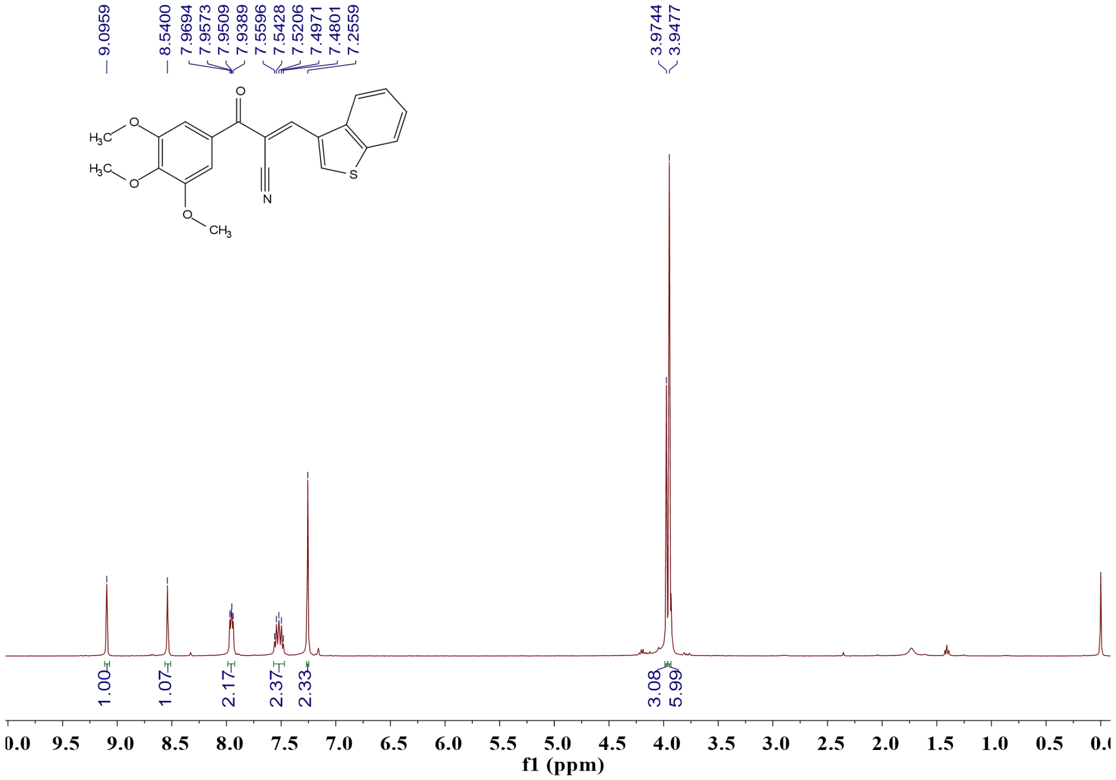

^{1}H NMR of **8p**

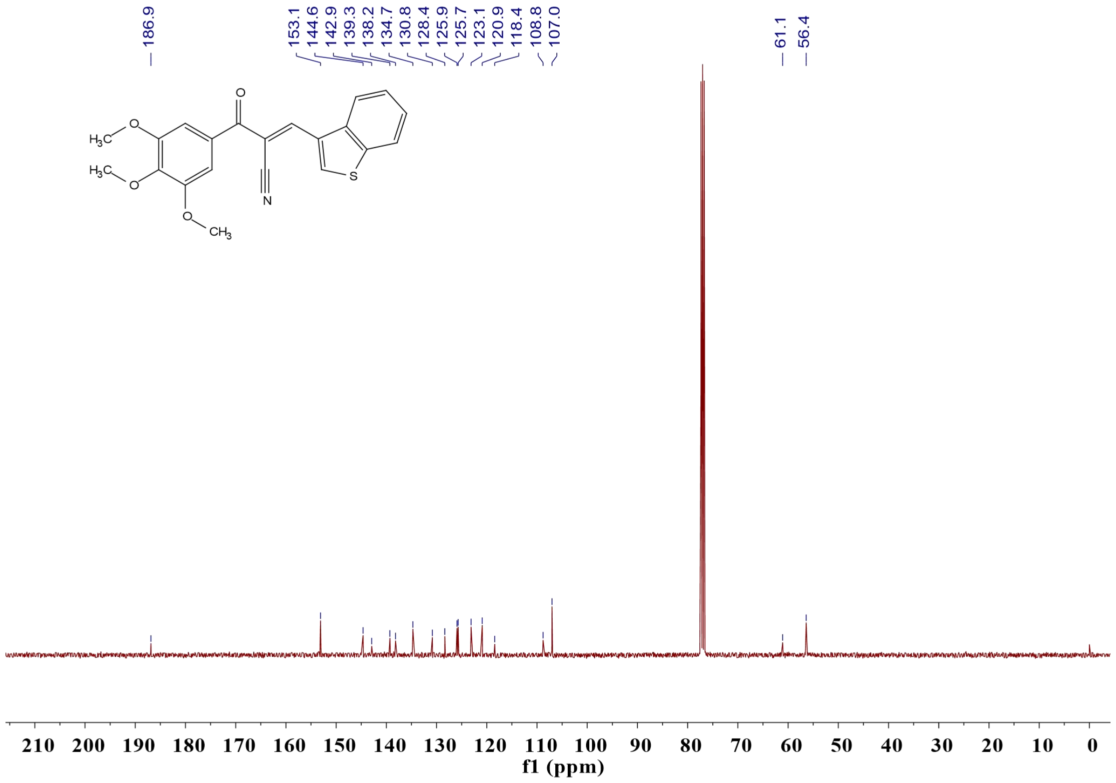

^{13}C NMR of **8p**

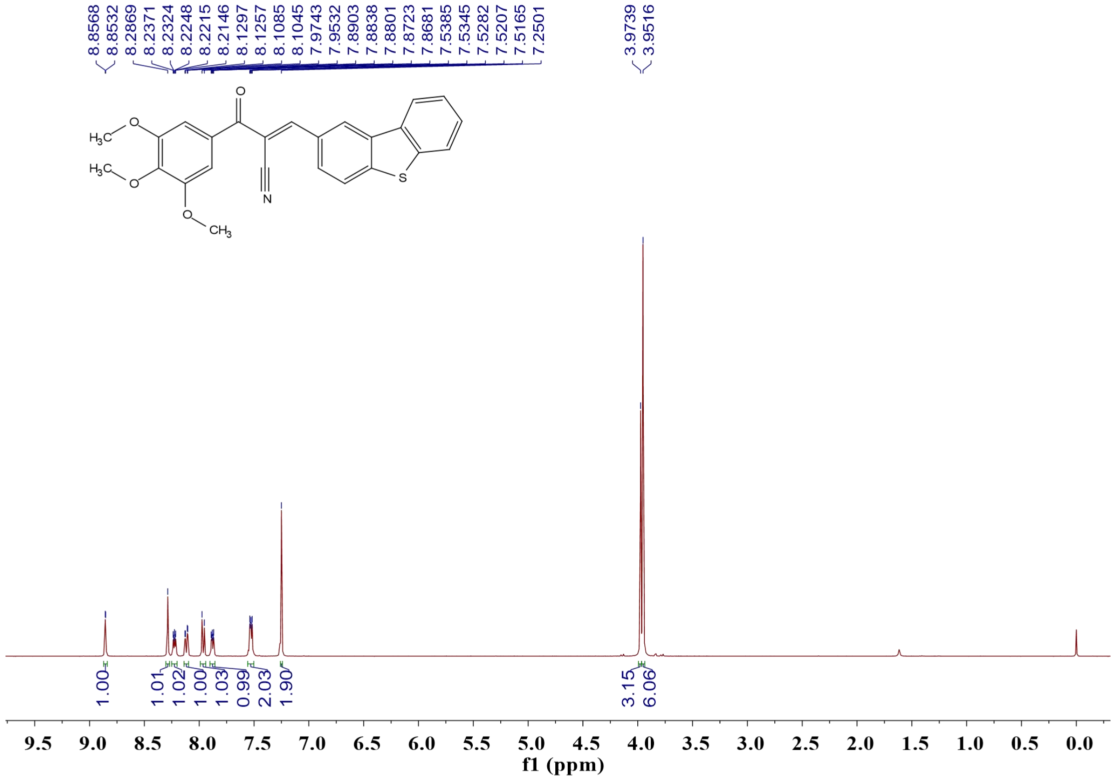

^{1}H NMR of **8q**

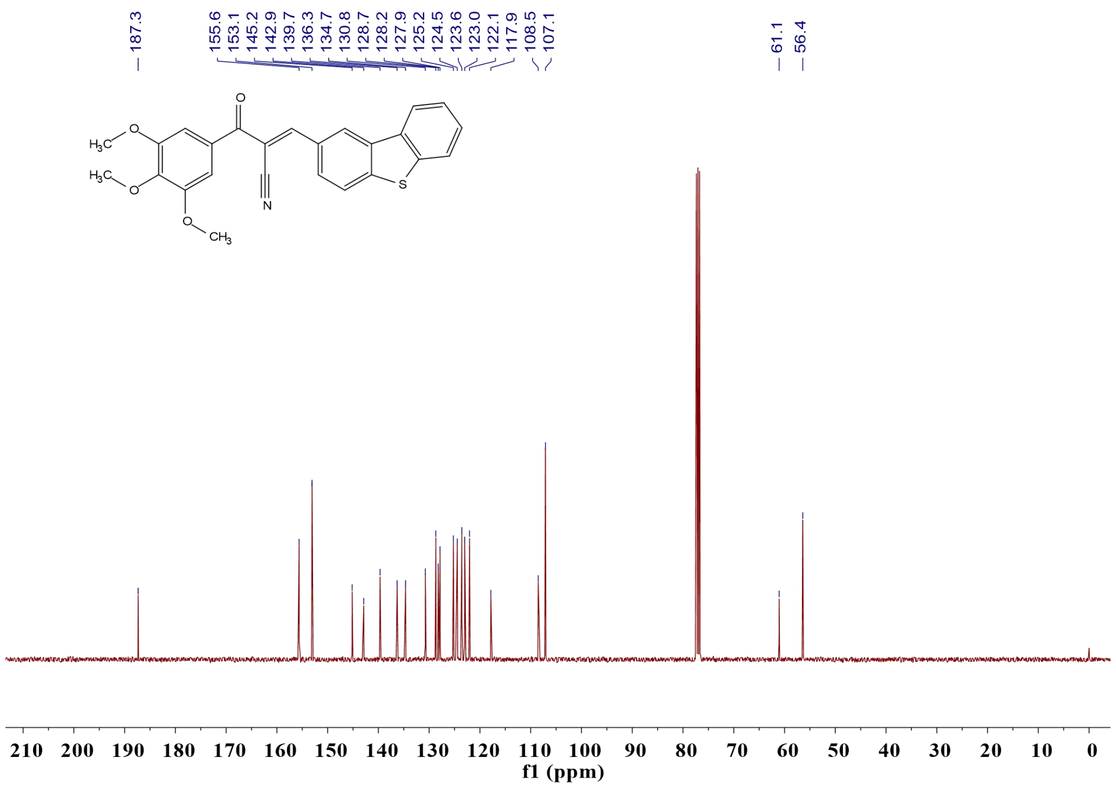

^{13}C NMR of **8q**

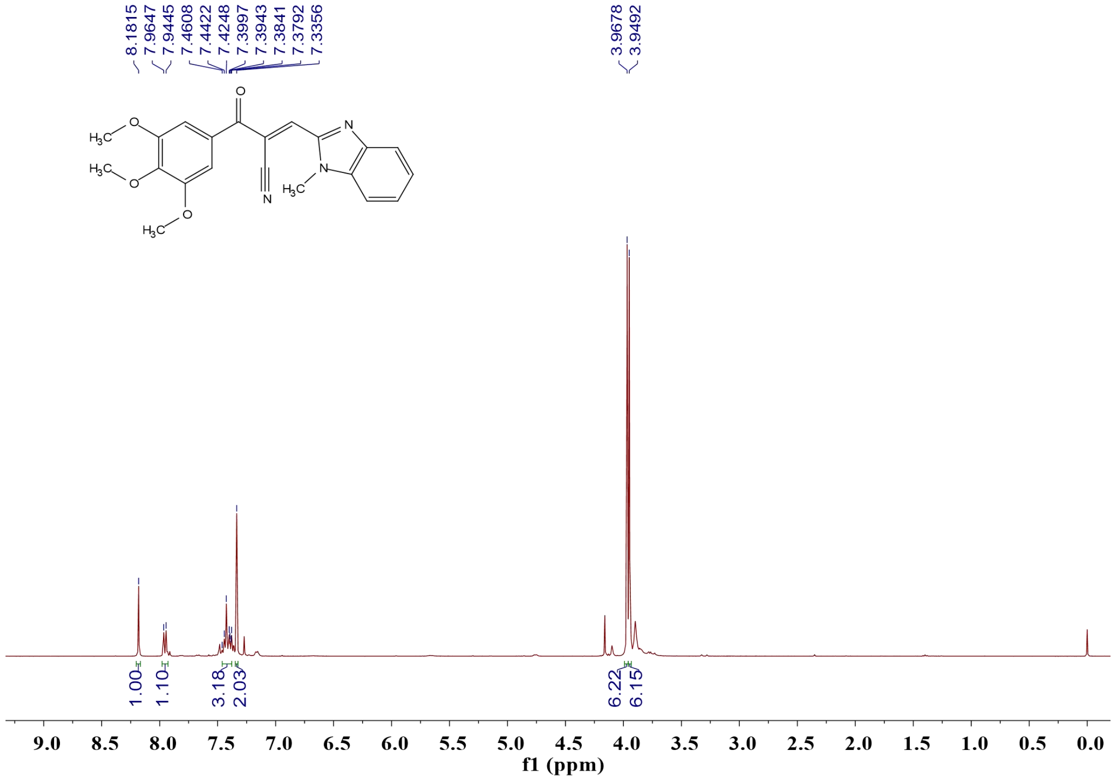

^{1}H NMR of **8r**

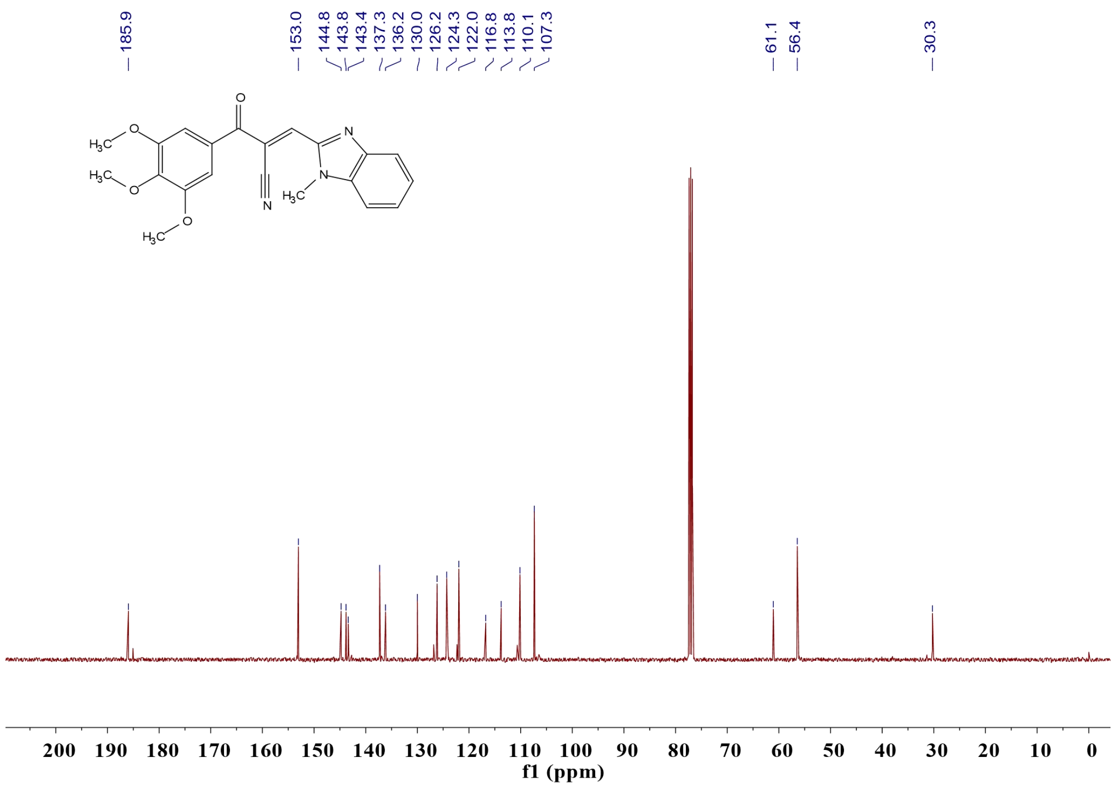

^{13}C NMR of **8r**

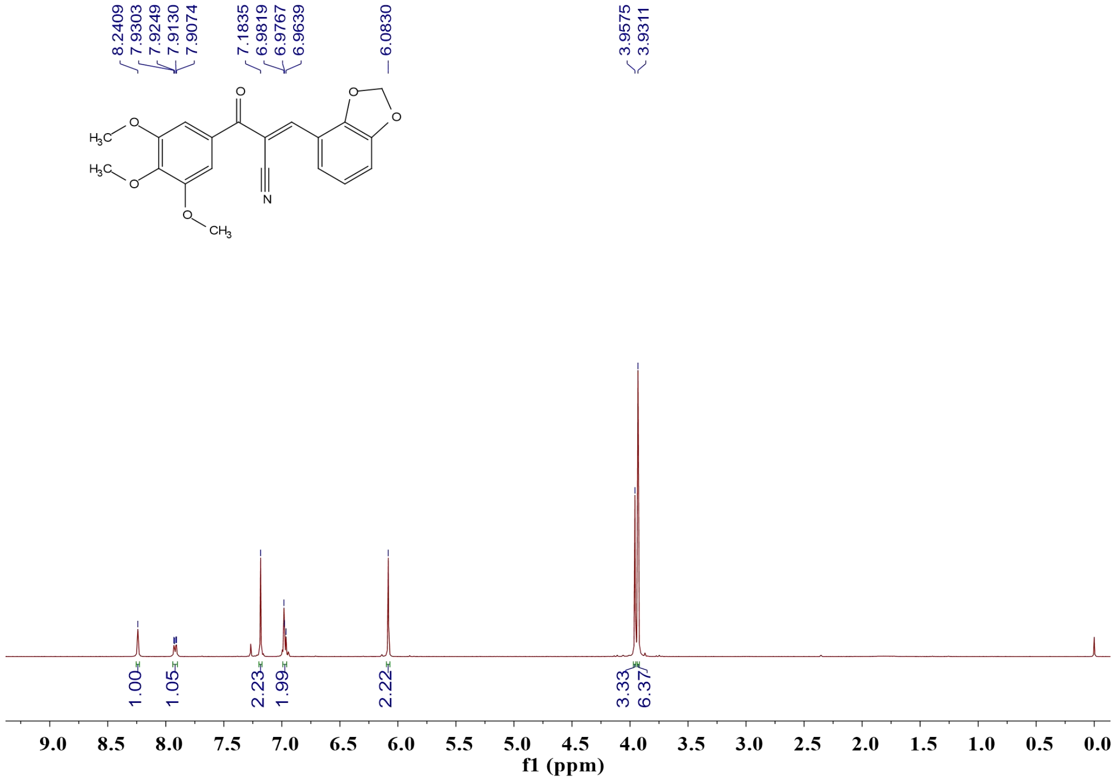

^{1}H NMR of **8s**

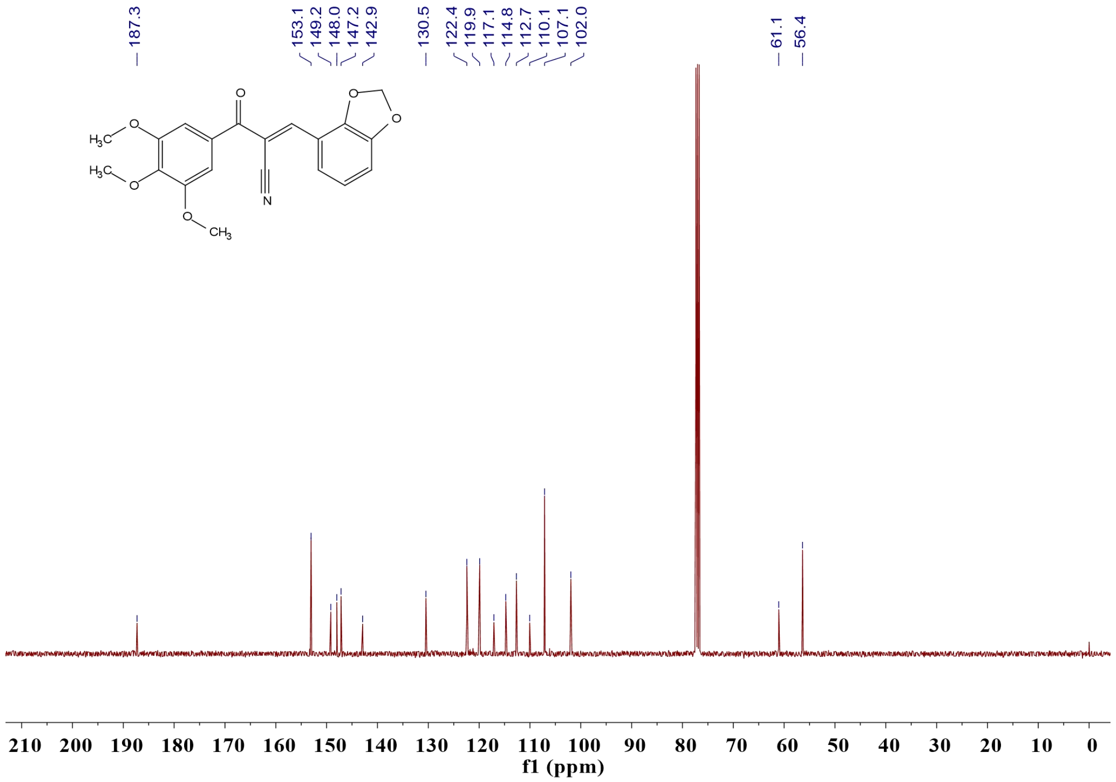

^{13}C NMR of **8s**

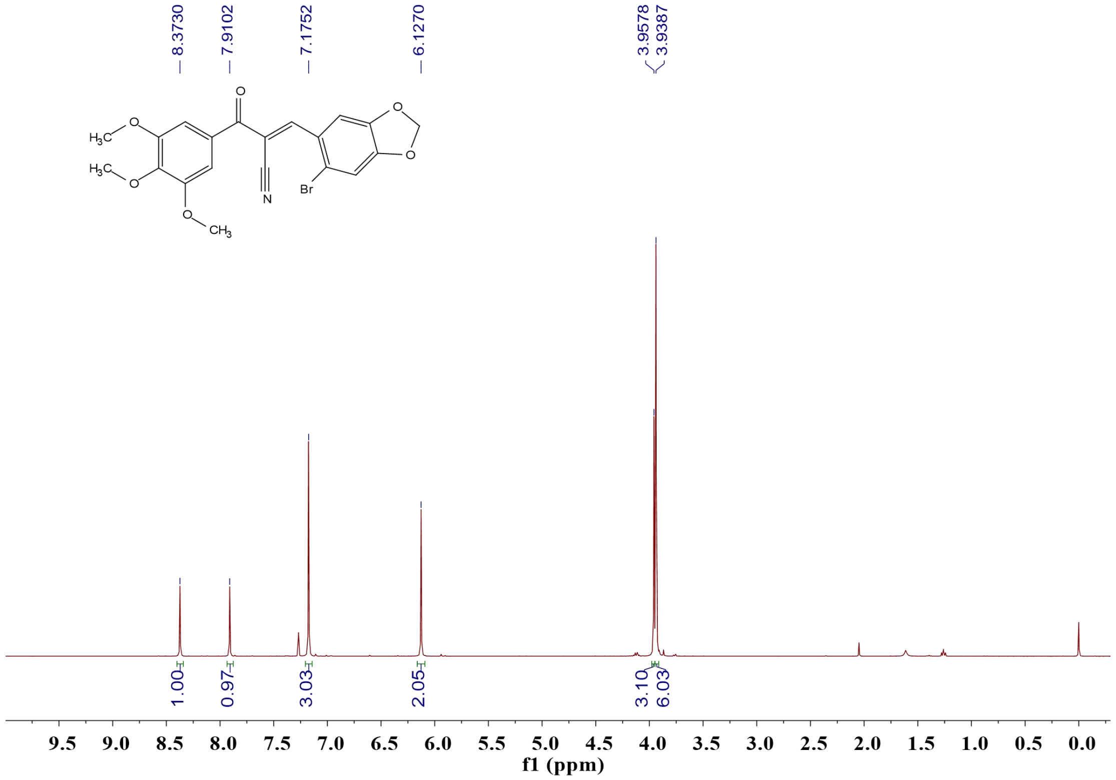

^{1}H NMR of **8t**

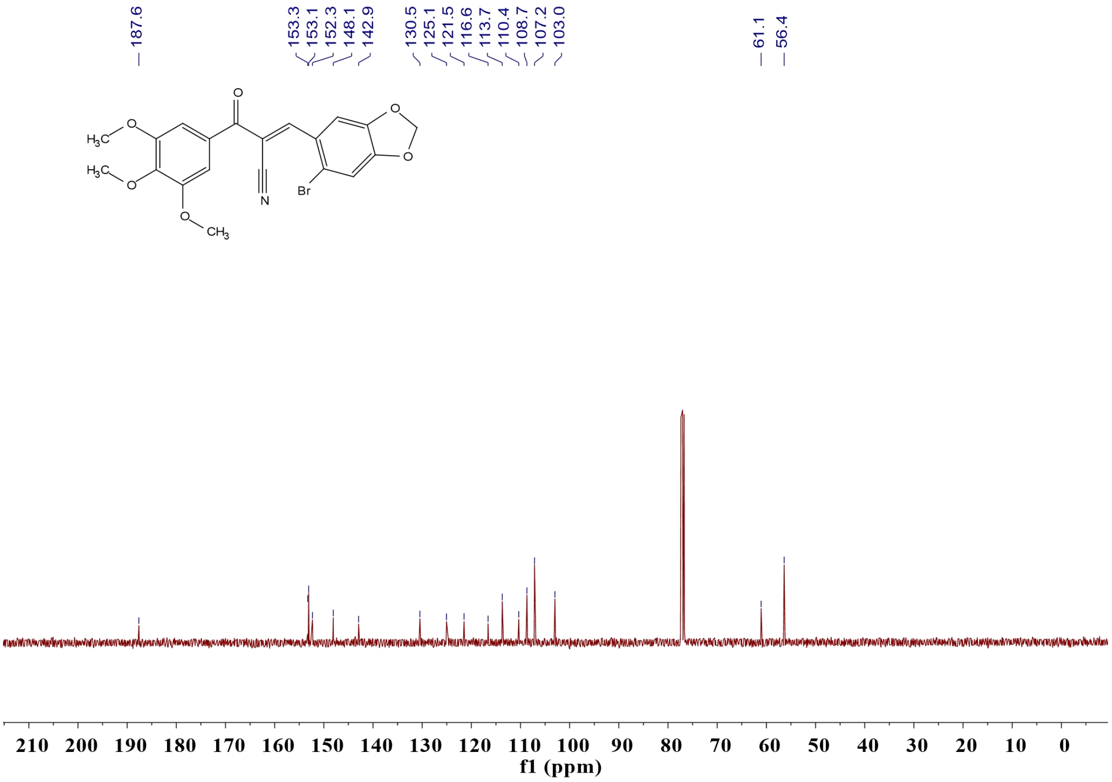

^{13}C NMR of **8t**

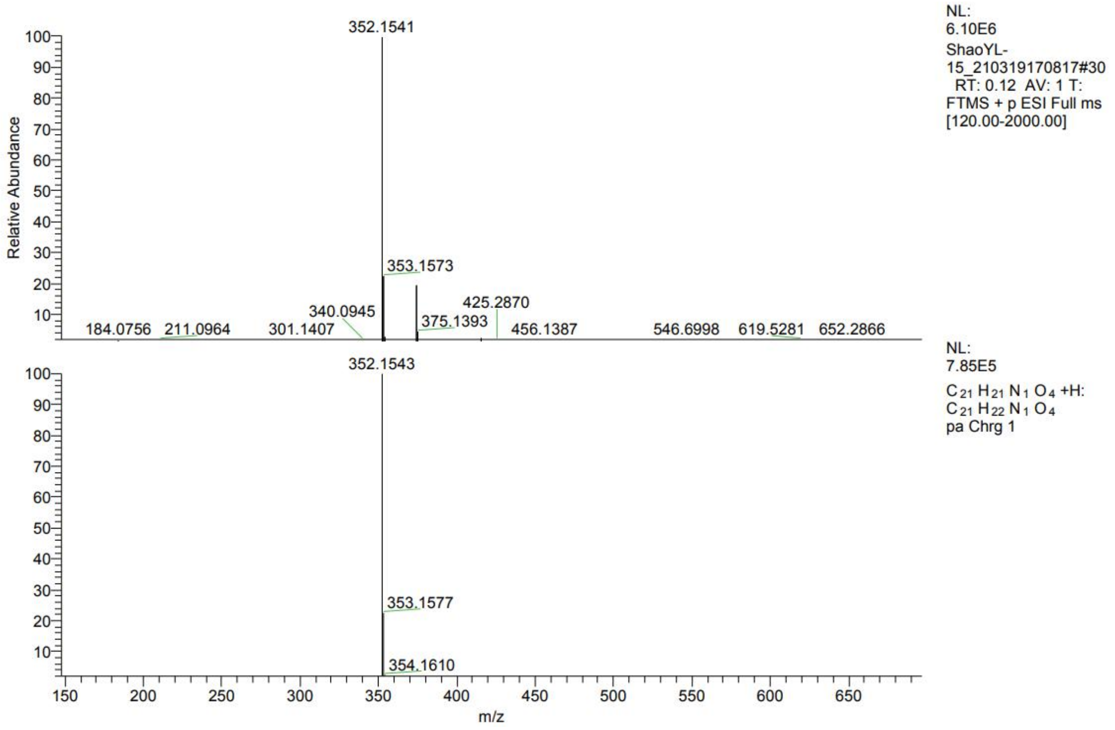

HRMS for **6b**

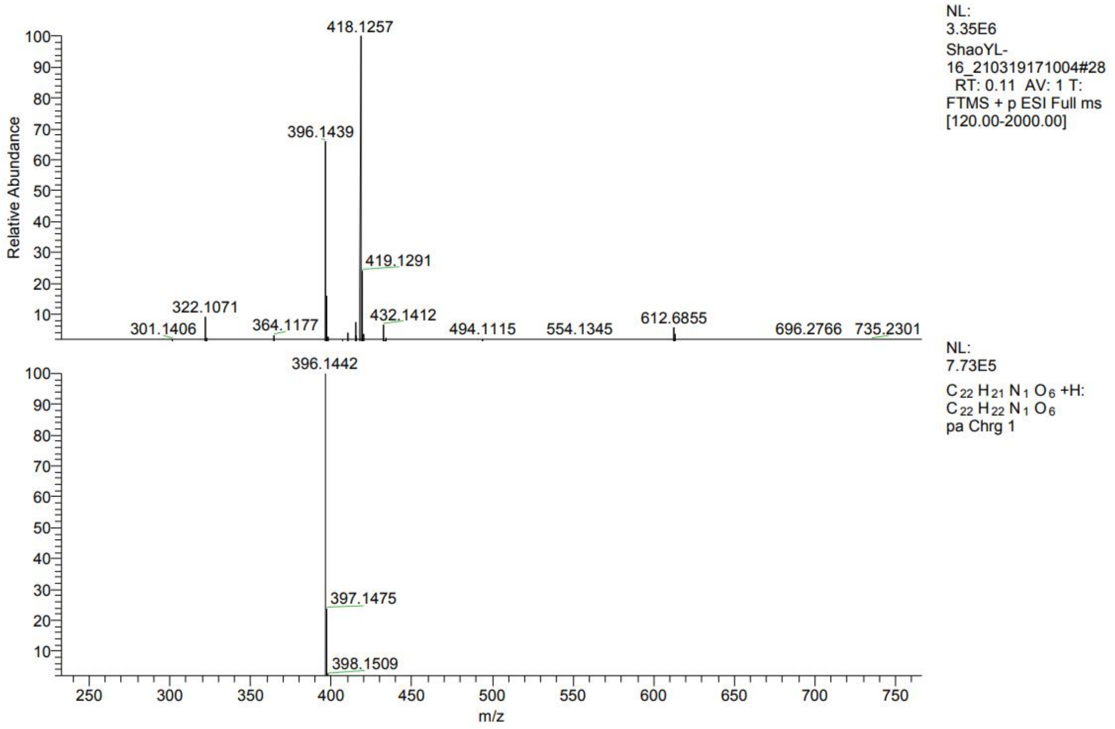

HRMS for **6d**

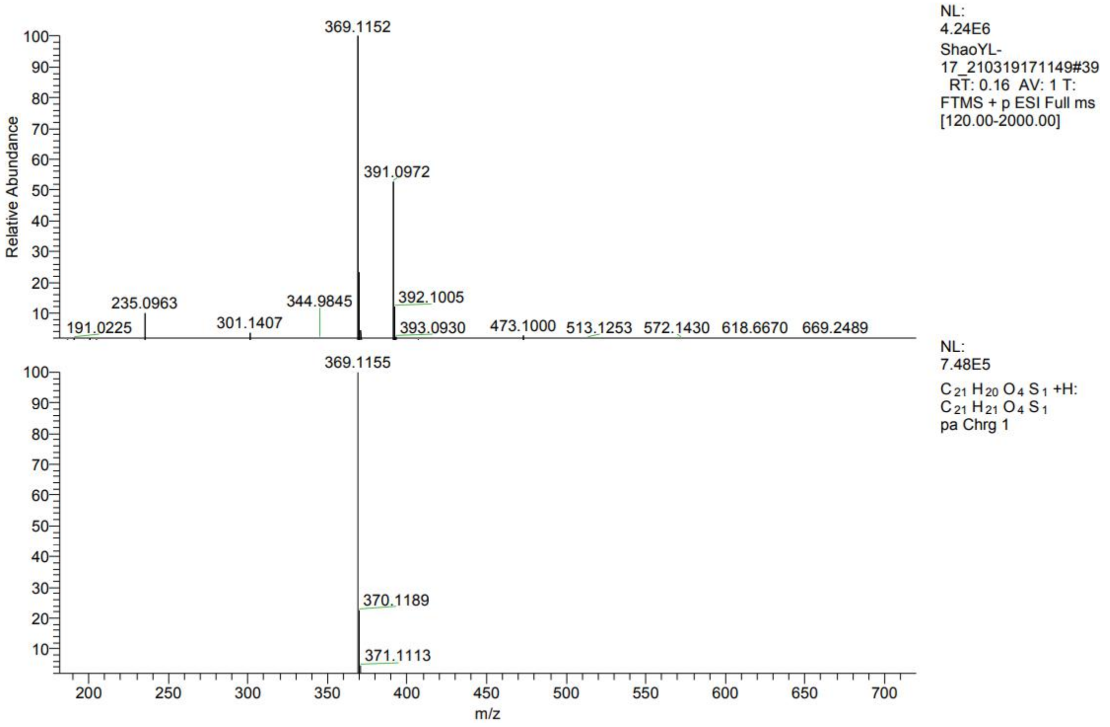

HRMS for **7o**

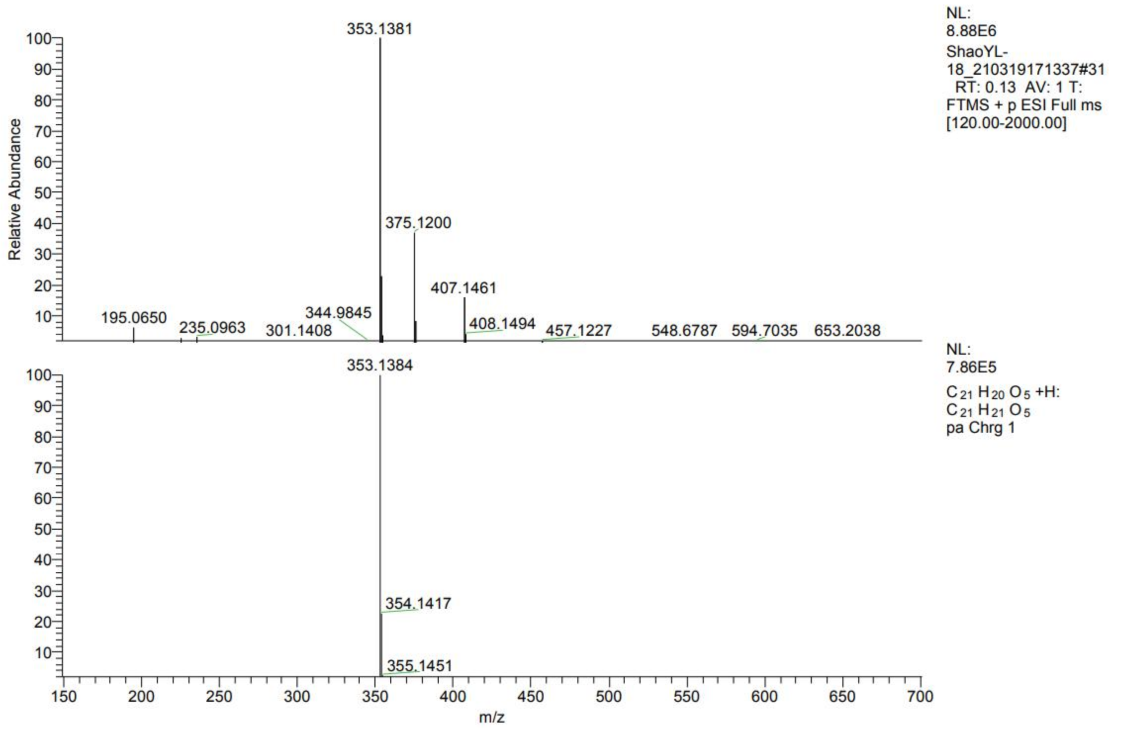

HRMS for **7m**

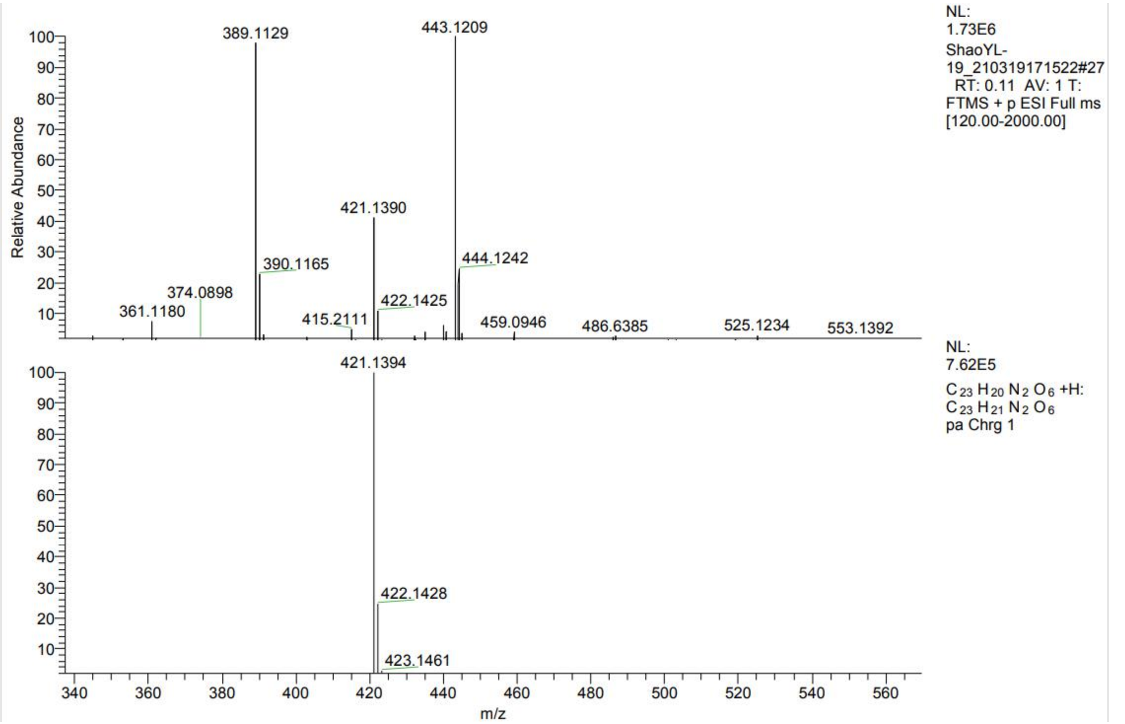

HRMS for **8d**

Reference

Chaitanya, M., Reddy, P., Kumar, N., Kumar, A., and Kumar, D. (2018). Synthesis and anticancer activity studies of indolylisoxazoline analogues. *Bioorganic & Medicinal Chemistry Letters* 28(17), 2842-2845. doi: 10.1016/j.bmcl.2018.07.035.

Karunakaran, J., Moorthy, N.D., Chowdhury, S.R., Iqbal, S., and Mohanakrishnan, A.K. (2019). Divergent Synthesis and Evaluation of the invitro Cytotoxicity Profiles of 3,4 - Ethylenedioxythiophenyl - 2 - propen - 1 - one Analogues. *ChemMedChem* 14(15), 1418-1448. doi: 10.1002/cmdc.201900225.

Koneni, V., Sashidhara, Ram, K., Modukuri, et al. (2014). Benzofuran-Chalcone Hybrids as Potential Multifunctional Agents against Alzheimer's Disease: Synthesis and in vivo Studies with TransgenicCaenorhabditis elegans. *ChemMedChem* 9(12), 2671-2684. doi: 10.1002/cmdc.201402291.

Narender, T., and Reddy, K.P. (2007). A Simple and Highly Efficient Method for the Synthesis of Chalcones by Using Borontrifluoride-etherate. *Tetrahedron Letters* 48(18), 3177–3180. doi: 10.1016/j.tetlet.2007.03.054.

Tanpure, R.P., Harkrider, A.R., Strecker, T.E., Hamel, E., Trawick, M.L., and Pinney, K.G. (2009). Application of the McMurry coupling reaction in the synthesis of tri- and tetra-arylethylene analogues as potential cancer chemotherapeutic agents. *Bioorganic & Medicinal Chemistry* 17(19), 6993-7001. doi: 10.1016/j.bmc.2009.08.011.

Wang, G., Peng, Z., Peng, S., Qiu, J., Li, Y., and Lan, Y. (2018). (E)-N-Aryl-2-oxo-2-(3,4,5-trimethoxyphenyl)acetohydrazonoyl cyanides as tubulin polymerization inhibitors: Structure-based bioisosterism design, synthesis, biological evaluation, molecular docking and in silico ADME prediction. *Bioorg Med Chem Lett* 28(20), 3350-3355. doi: 10.1016/j.bmcl.2018.09.004.

Wang, K.K., Wang, P., Ouyang, Q., Du, W., and Chen, Y.C. (2016). Substrate-Controlled Switchable Asymmetric Annulations to Access Polyheterocyclic Skeletons. *Chemical Communications* 52(74), 11104.

Yan, J., Chen, J., Zhang, S., Hu, J., Huang, L., and Li, X. (2016). Synthesis, Evaluation, and Mechanism Study of Novel Indole-Chalcone Derivatives Exerting Effective Antitumor Activity Through Microtubule Destabilization in Vitro and in Vivo. *Journal of Medicinal Chemistry* 59(11), 5264-5283. doi: 10.1021/acs.jmedchem.6b00021.
